# Silicon carbide formation from methane and silicon monoxide

**DOI:** 10.1038/s41598-020-79006-6

**Published:** 2020-12-11

**Authors:** Trygve Storm Aarnæs, Eli Ringdalen, Merete Tangstad

**Affiliations:** 1 Department of Materials Science and Engineering, Norwegian University of Science and Technology, Trondheim, Norway; 2 Metal Production and Processing, SINTEF Industry, Trondheim, Norway

**Keywords:** Chemistry, Materials science

## Abstract

Silicon carbide (SiC) formation plays an important role during the production of elemental silicon. SiC forms through a high temperature reaction between silicon monoxide gas (SiO) and carbon. Currently, the carbon sources are solids, however finding a way of substituting the solid carbon with methane could have several advantages. SiC formation was studied in argon, hydrogen and methane containing atmospheres at 1650 °C and 1750 °C. SiO gas was generated from pellets of a 1:2 molar ratio of SiC and silica (SiO_2_). The reactions were investigated through CO off-gas analysis in conjunction with measuring the weight change. After each experiment, the reaction products were examined in a scanning electron microscope with secondary electrons and through energy-dispersive X-ray spectroscopy. It was confirmed that SiC may form from SiO and methane. Increasing the methane content to 5% caused a significant increase in SiC formation. Furthermore, the SiC structure was also highly sensitive to the methane content that was used. In addition, the SiO producing reaction was affected by hydrogen. The hydrogen lead to an increased rate of SiO formation relative to what was seen in argon. The effect of hydrogen was most pronounced at 1750 °C which is right after the melting of silica.

## Introduction

Modern silicon production takes place in submerged arc furnaces (SAF). The items necessary to produce silicon are silica (SiO_2_), which is added as quartz, carbon that comes in the form of woodchips, charcoal and coal, and large amounts of electric energy^1^. It is convention to divide the SAF into two distinct regions, an upper and a lower region, separated by a temperature threshold. The two key intermediate species that form within the furnace are silicon carbide (SiC) and silicon monoxide gas (SiO). SiO forms in the bottom region of the furnace from reaction (1). SiO may then rise through the furnace. As it reaches the upper colder region, either it will react with carbon according to reaction (2), with CO according to reaction (3), or two SiO molecules may form a compound consisting of silica and silicon according to reaction (4).

1 $${\text{2SiO}}_{{2}} {\text{ + SiC = 3SiO(g) + CO(g)}}$$

2 $${\text{SiO(g) + 2C = SiC + CO(g)}}\quad \Delta {\text{H}}_{{{1200}^{ \circ } {\text{C}}}} = - {75}\,{\text{kJ}}^{{2}}$$

3 $${\text{3SiO(g) + CO(g) = 2SiO}}_{{2}} {\text{ + SiC}}$$

4 $${\text{2SiO(g) = SiO}}_{{2}} {\text{ + Si}}$$

Producing silicon is an energy intensive endeavor. Producing one metric ton requires 11–13 MWh of electric energy^3^. In addition to the electrical energy, a large amount of energy must be supplied in the form of carbon, which also acts as the reducing agent. As a result, the CO_2_ emissions of the process depend heavily on both how the electricity is produced and on the types of carbon used. Charcoal or other biocarbons are CO_2_ neutral in the sense that the CO_2_ released was initially captured when the biological matter formed. The primary purpose of the carbon is to react with the SiO gas which rises through the furnace, because SiO that makes it past the upper region without reacting will represent a large reduction in the furnace’s energy efficiency. The SiO reactivity of different carbon materials can be quantified, for example, through the SINTEF SiO reactivity test^4^.

Recent work by Li et al. investigated the effect hydrogen has on SiO, SiC and Si formation^5–8^. They found that hydrogen could result in a large improvement of the kinetics in the Si–O–C system. There were two main mechanisms suggested. Firstly, hydrogen can enhance mass transfer through improved diffusion. Secondly, the addition of hydrogen to the system allows small amounts of methane (CH_4_) to form which enables much faster gas phase mass transfer of carbon. It was also suggested that hydrogen could directly reduce SiO_2_ to SiO increasing the SiO pressure thus promoting the formation of SiC and Si.

**Figure 1:**
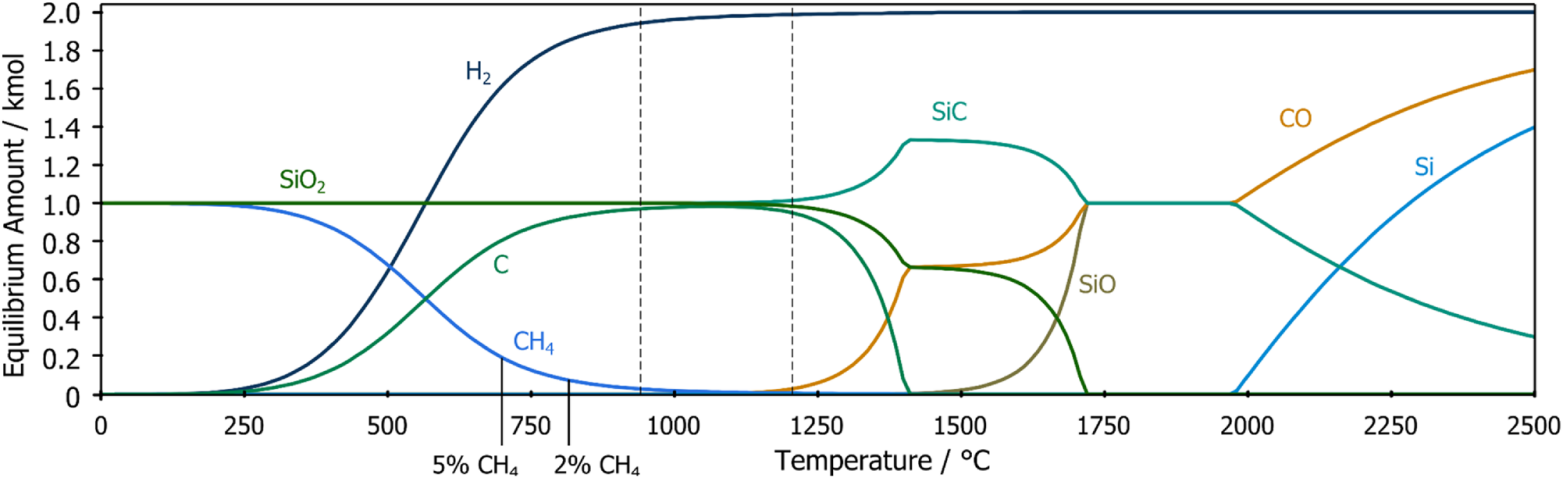
Equilibrium amounts of the main species of a system containing CH_4_ and SiO gas calculated in HSC9. Low temperatures favours CH_4_ while high temperatures favour SiO gas, and inbetween there is a temperature range where neither gas is favoured.

A carbon source that is currently not being used in silicon production is methane. Preliminary research by Monsen et al. suggests that it may have several advantages, such as a high SiO reactivity, and not contributing heat the upper furnace zone^9^. It has been documented that methane can act as the carbon source when forming SiC whiskers^10^. At high temperatures, methane may crack to hydrogen and carbon according to reaction (5). The left side of Fig. 1 shows how the equilibrium methane composition changes with temperature. On the right side of Fig. 1 we see how the equilibrium amount of SiO gas is affected by temperature. Because there is no overlap between the temperature region where SiO gas and where methane is thermodynamically stable, a reaction between these gasses will depend on how far away from equilibrium it is possible to operate. However, the rate of cracking depends not only on temperature but also on the surface it occurs at. Dalaker et al. measured the temperatures methane cracking began at for different oxides^11^.

5 $${\text{CH}}_{{4}} {\text{(g) = C + 2H}}_{{2}} {\text{(g)}}$$

The aim of this study is to examine the possibility of producing SiC from CH_4_ and SiO. As seen in Fig. 1 there is a large temperature range were neither SiO nor CH_4_ are thermodynamically stable. It is thus necessary to understand how methane is affected by operating at high temperatures. How high CH_4_ pressures should be used? How is the reaction between CH_4_ and SiO gas affected by temperature, and to what extent does thermal cracking of methane pose an issue? In conclusion, the study aims to determine if the reaction is possible at all, despite the large temperature spread between their respective stability domains. Additionally, because the SiO gas must be produced in situ the current study produces a prime opportunity to also look at how SiO formation from SiC/SiO_2_ pellets is influenced by the various process gasses.

## Experimental

The SiO used in each experiment was generated through reaction (1), from pellets mixed with a 1:2 molar ratio of SiC and SiO_2_. The SiO_2_ and SiC were first crushed to powders with a particle size of roughly 5 µm. Afterwards they were pelletized to produce pellets with a diameter of 1–1.5 mm. The pellets were then dried before being calcined at 1200 °C for 30 min.

**Figure 2:**
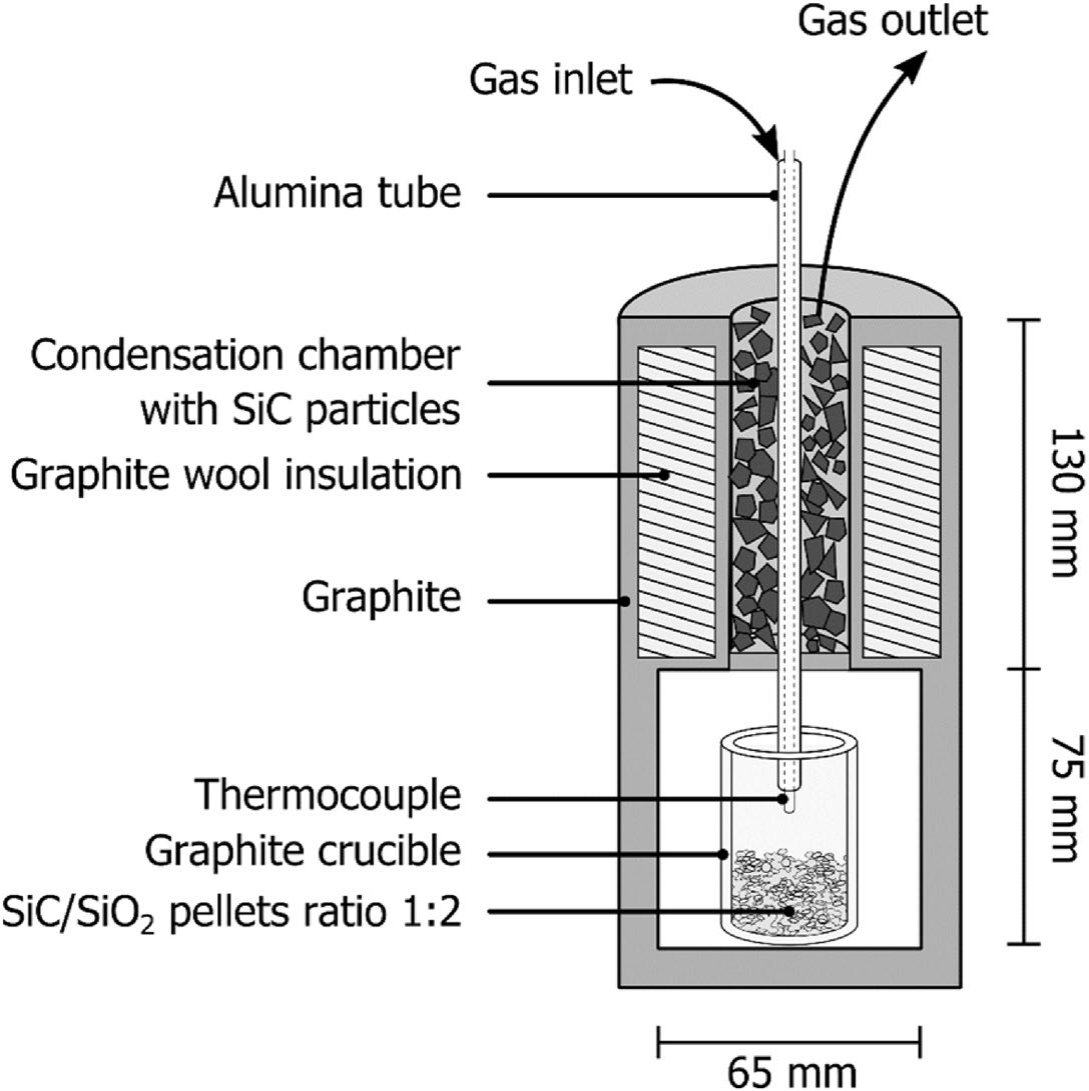
A schematic drawing of the setup used to investigate the reaction between SiO and CH_4_ in a CH_4_/H_2_ atmosphere.

A schematic overview of the experimental setup is shown in Fig. 2. It involves two sections; the lower section is the reaction chamber and the top section is the condensation chamber. The reaction chamber contains a graphite crucible containing 5 g of pellets. An alumina tube was positioned within the crucible and was used for process gas injection. To measure the temperature a type-C thermocouple was passed through the tube. The condensation chamber was a cylindrical section surrounded by graphite wool insulation and filled with SiC particles. An additional thermocouple was positioned next to the heating element and was used for temperature control. When ready, the setup was inserted into a resistance heated vertical tube furnace. While the off-gas went through a gas analyzer which determined its CO content.

**Figure 3:**
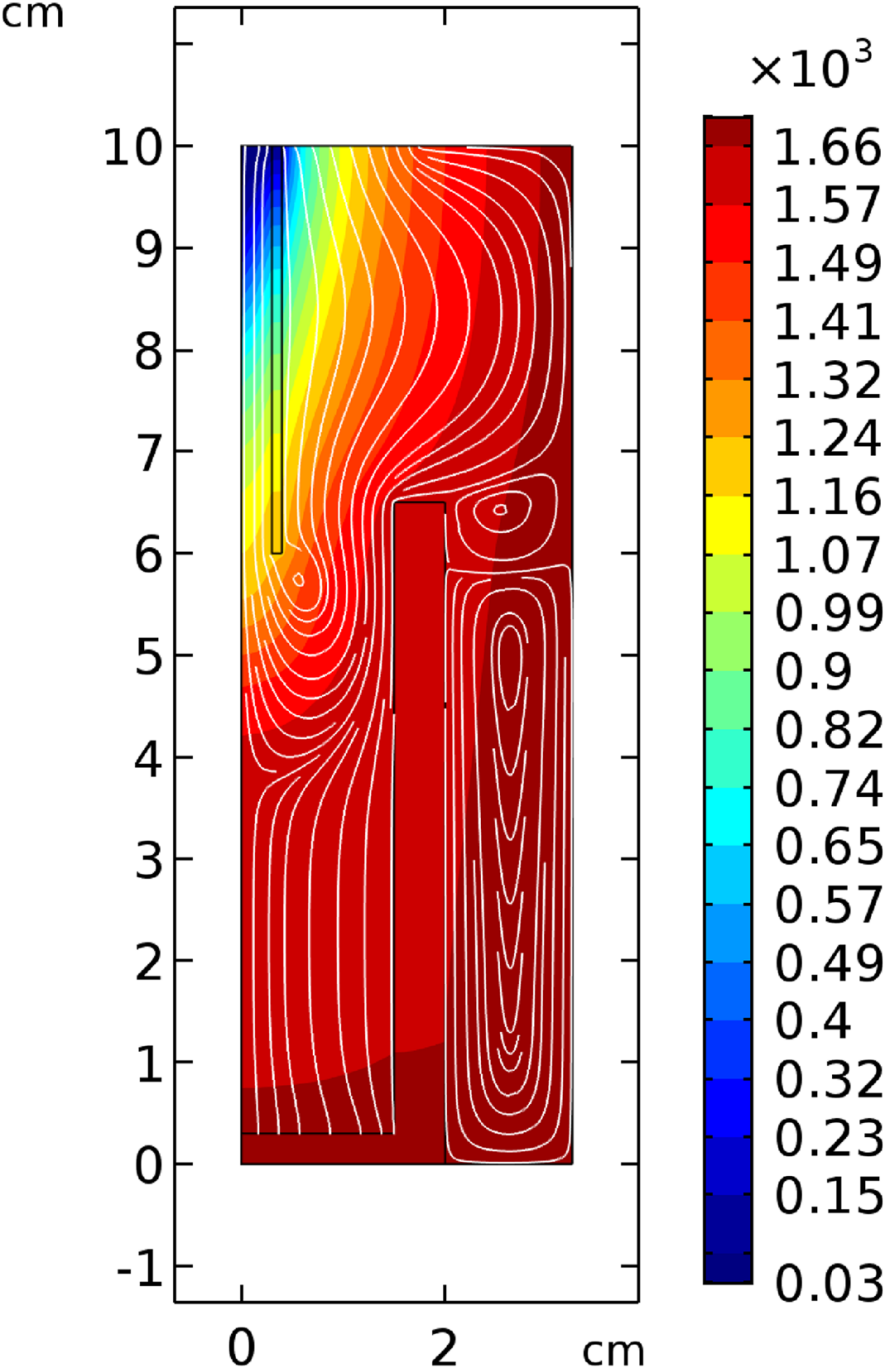
A COMSOL Multiphysics simulation of gas flow within the reaction chamber.

A COMSOL simulation done to get an overview of how the gas flows through the reaction chamber is shown in Fig. 3. It shows that when the gas from the raw material meets the process gas flow, it is pushed outwards and flows along the crucible surface. As a result, reaction products should mostly form on the crucible walls.

The weight of the crucibles containing the raw material was recorded before and after each experiment. The crucibles were then cut perpendicularly to obtain an interior cross section that could be examined in a scanning electron microscope (SEM) with secondary electrons, and through energy dispersive X-ray spectroscopy (EDS). Due to the nature of the samples the EDS spots often do not have a surface that is flat, perpendicular to the electron beam, or even continuous. As a result a large amount of EDS spots was done to minimize uncertainty. The EDS spectra and corresponding quantitative analysis are included as part of the supplementary information.

Experiments were performed at 1650 °C and at 1750 °C, below and above the melting point of SiO_2_. The process gasses that were used were Ar, and CH_4_/H_2_ mixtures with a CH_4_ content ranging from 0–5%. An overview of the experiments that were performed is displayed in Table 1.

**Table 1:** The experimental matrix, the number in each cell indicates the number of parallels.

	Pure Ar	%CH_4_ in a CH_4_/H_2_ gas mix		
		0%	2%	5%
1750 °C	3	2	4	2
1650 °C	2	2	4	2

## Results/discussion

### SiC formation

**Figure 4:**
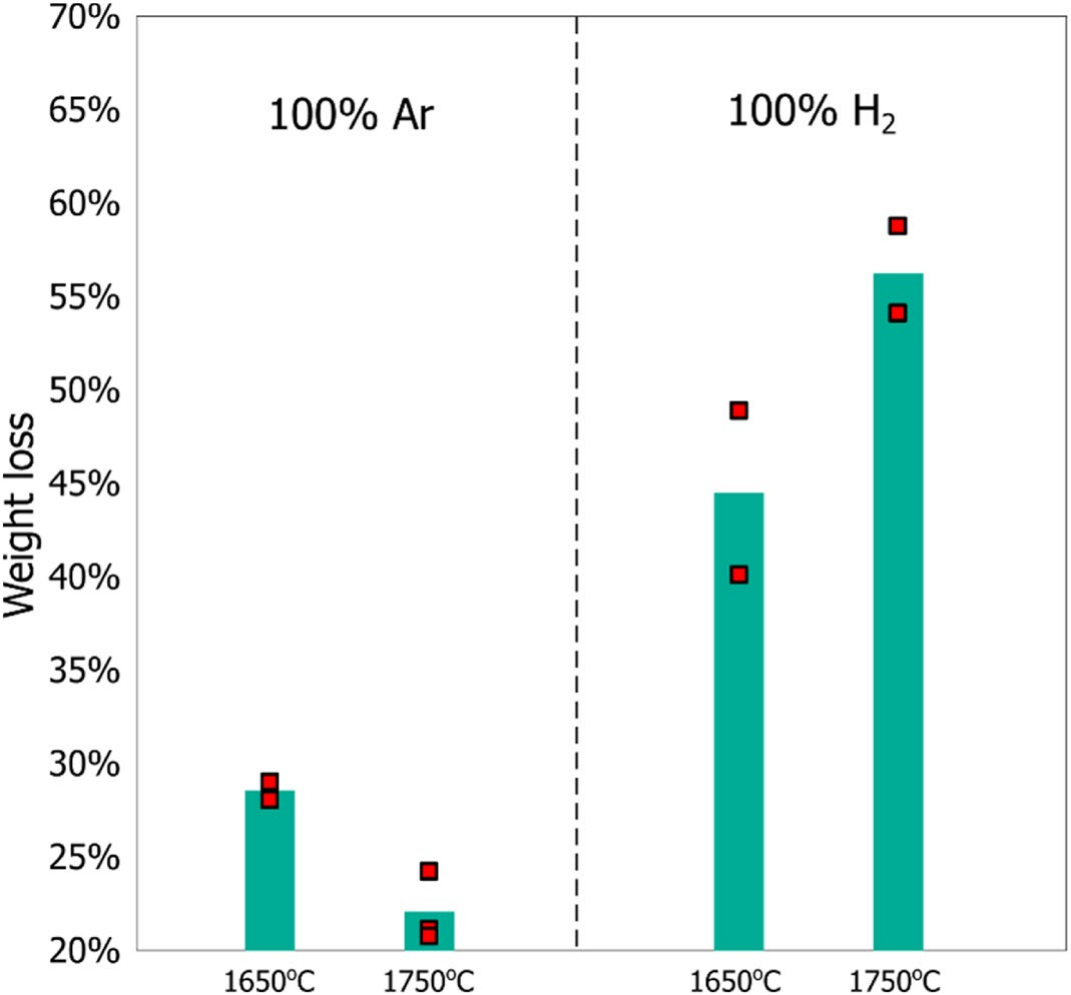
A comparison of the weight loss relative to starting weight of the raw material when using Ar and when using H_2_ as the process gas. The weight loss includes the crucible weight change and the raw material weight loss.

**Figure 5:**
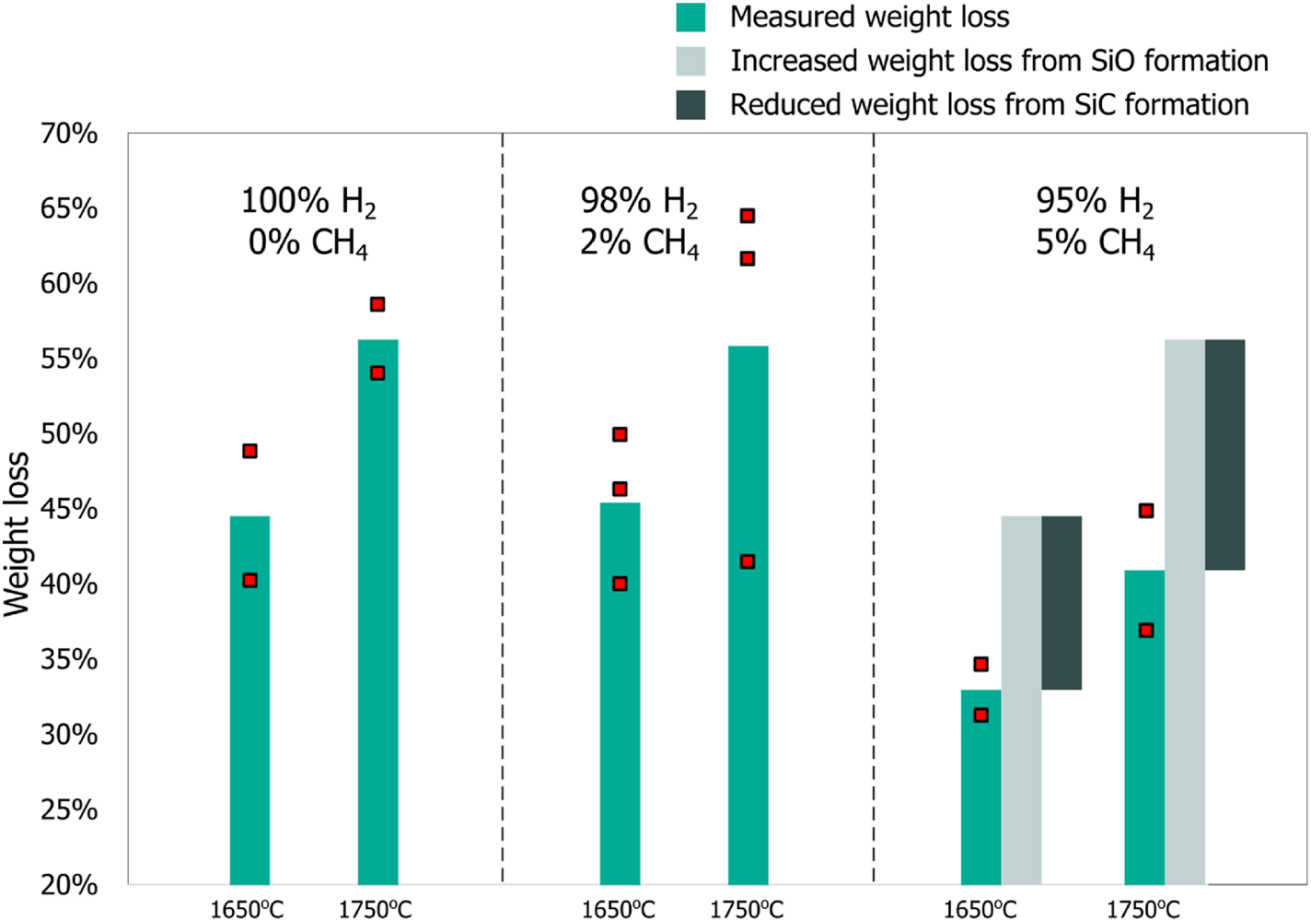
A comparison of weight loss relative to starting weight of the raw material at each temperature and process gas. The weight loss includes the crucible weight change and the raw material weight loss.

The weight change of the raw material and crucible observed during each experiment revealed that a substantial amount of SiC formed in CH_4_ containing atmospheres. The weight loss, displayed in Figs. 4 and 5, has two main contributions. The first is the raw material reacting according to reaction (1) forming SiO and CO, which may then escape the crucible and the reaction chamber. The second contribution is SiC formation, when SiO and CH_4_ reacts it may deposit SiC on the crucible according to reaction (6).

6 $${\text{2CH}}_{{4}} {\text{(g) + SiO(g) = SiC + CO(g) + 4H}}_{{2}} {\text{(g)}}\quad \Delta {\text{H}}_{{{12}00^{ \circ } {\text{C}}}} { = 1}0{5}\,{\text{kJ}}^{{2}}$$

Additional reactions that would affect the weight loss were also considered. One possibility is SiO and graphite from the crucible reacting to form a SiC layer according to reaction (2), which would reduce the weight loss. However, it was determined to be a small contribution because very small amounts of SiC that formed through this mechanism were observed when examining the crucibles in SEM. A second possibility is thermal cracking of CH_4_, according to reaction (5), which would result in a reduced weight loss. This mechanism was also deemed negligible, which was seen by performing experiments without raw material but with CH_4_, which did not result in a weight change.

Far higher weight loss was seen in H_2_ containing atmospheres than in Ar. Figure 4 shows the weight loss that was seen with Ar and H_2_ as the process gas. We see that, relative to Ar, pure H_2_ saw an increase in the reaction rate of reaction (1) of 57% at 1650 °C, and 161% increase at 1750 °C. Similar results to these have been reported by other researchers^7,10,12^. The most common mechanism used to explain it is the increased diffusion in H_2_, which speeds up mass transfer of SiO and CO which will affect all present reactions. Another possibility is that the presence of H_2_ and CH_4_ allows the reaction to proceed through different reaction steps, which allows for the much faster reaction rate.

Figure 5 shows that increasing the CH_4_ content to 5% CH_4_ led to a noticeable reduction in weight loss. This is a result of increased SiC formation, and therefore reduced weight loss due to the gasses depositing a SiC layer at the crucible surface. The amount of SiO that was transformed to SiC is calculated by comparing the weight loss at 0% CH_4_ to that at 5% CH_4_. The weight loss at 0% CH_4_ expresses the amount of SiO gas production. Shown as the light gray bar in Fig. 5. The difference in weight loss between 0 and 5% CH_4_ tells us the amount of SiC that was produced. Shown as the dark grey bar in Fig. 5. Performing the calculations reveals that at least 22% of the SiO was transformed to SiC. The amount of SiO that reacted was probably higher because the current experimental setup is only capable of quantifying the SiC which is deposited on the crucible. Additional SiC that forms on the lance, and likely other places too, is not included. Not to mention that the SiC was deposited firmly on the surface, indicating that it did not form homogeneously in the gas phase before sticking to the crucible. As a result, it should be possible to further increase the capture rate of SiO by taking measures to increase the available surface area.

Temperature also had a large effect on the weight loss caused by the reaction between SiO_2_ and SiC, which produced SiO gas, reaction (1). Increasing the temperature from 1650 to 1750 °C had opposite effects in Ar and in H_2_ containing atmospheres. In Ar the effect of increasing the temperature from 1650 to 1750 °C led to a reduction in weight loss and reaction rate. This can be explained by the melting of SiO_2_, which happens around 1710°C^2^. When SiO_2_ melts, it forms a viscous liquid that engulfs the SiC particles, effectively inhibiting gas bubble formation and escape from the SiC/ SiO_2_ interface. However, when using H_2_ containing atmospheres we see the opposite effect, an increased weight loss. Thus, hydrogen must somehow be improving mass transfer of SiO and CO from the SiC/SiO_2_ interface to the gas phase. Usually the effect of hydrogen is attributed to enhanced diffusion rates. Another possibility is that hydrogen may dissolve in the molten SiO_2_^13^, and the dissolved hydrogen produces an additional contribution participating in the formation of gas bubbles at the SiC/SiO_2_ interface.

**Figure 6:**
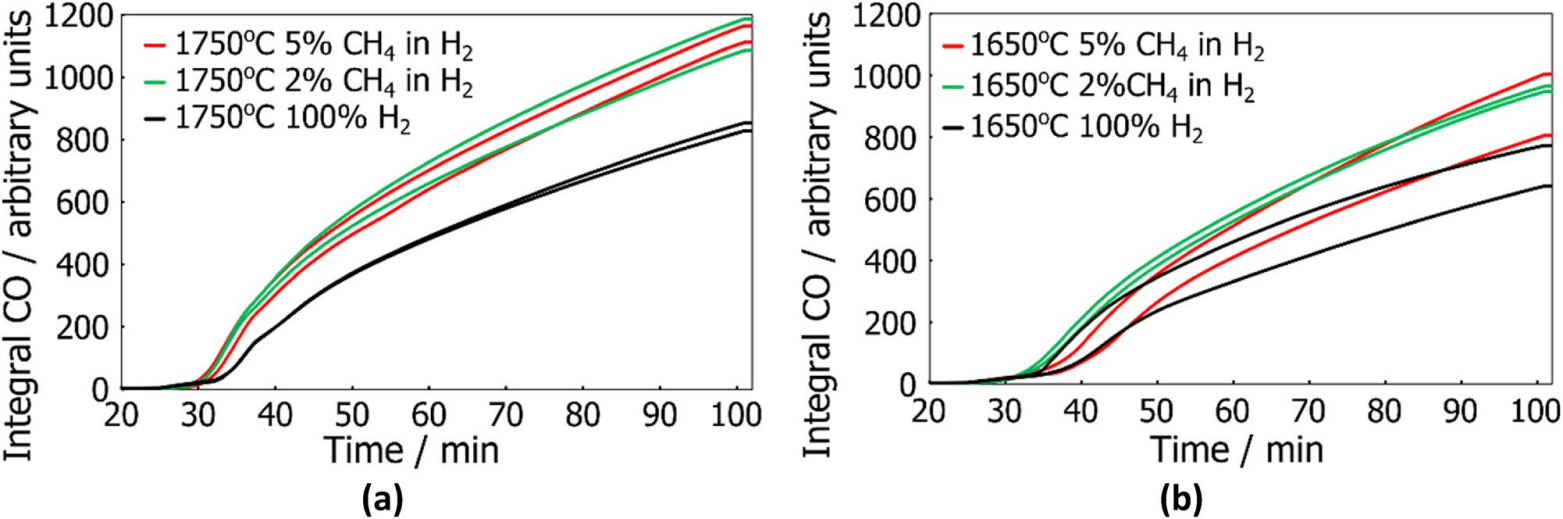
A comparison of the integral CO generated with different process gasses (**a**) at 1750 °C and (**b**) 1650 °C.

The CO content of the off-gas also points to CH_4_ reacting with SiO and producing SiC. The CO content of the off-gas is determined by two principal reactions. The first one is the formation of brown condensate, reaction (4). The second is SiC formation from CH_4_ and SiO, reaction (6). Figure 6 shows the integral CO amounts for the different process gasses at 1650 °C and at 1750 °C. In Fig. 6a we can see that at 1750 °C the CH_4_ containing process gasses generates the most CO and, by extension, the most SiC. We see the same effect at 1650 °C, which is shown in Fig. 6b, but the effect is a lot smaller. Thus, it points to CH_4_ having a more pronounced effect when it is used at higher temperatures.

### SiC structure

**Figure 7:**
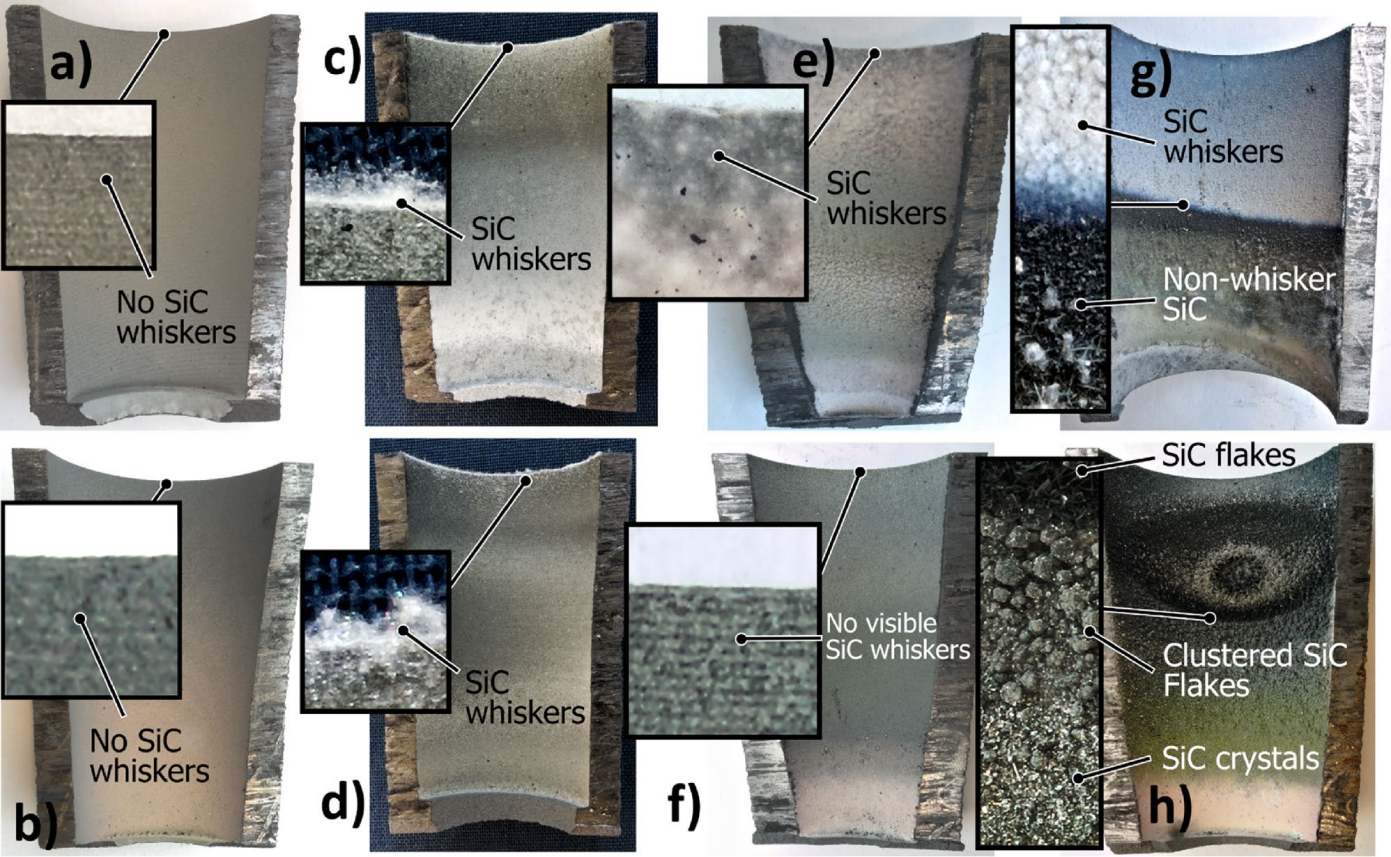
A comparison between crucible interiors after experiments **(a)** 1650 °C using Ar**, (b)** 1750 °C using Ar**, (c)** 1650 °C using pure H_2_, **(d)** 1750 °C using pure H_2_, **(e)** 1650 °C using 2% CH_4_ in H_2_, **(f)** 1750 °C using 2% CH_4_ in H_2_**, (g)** 1650 °C using 5% CH_4_ in H_2_ and **(h)** 1750 °C using 5% CH_4_ in H_2_.

**Figure 8:**
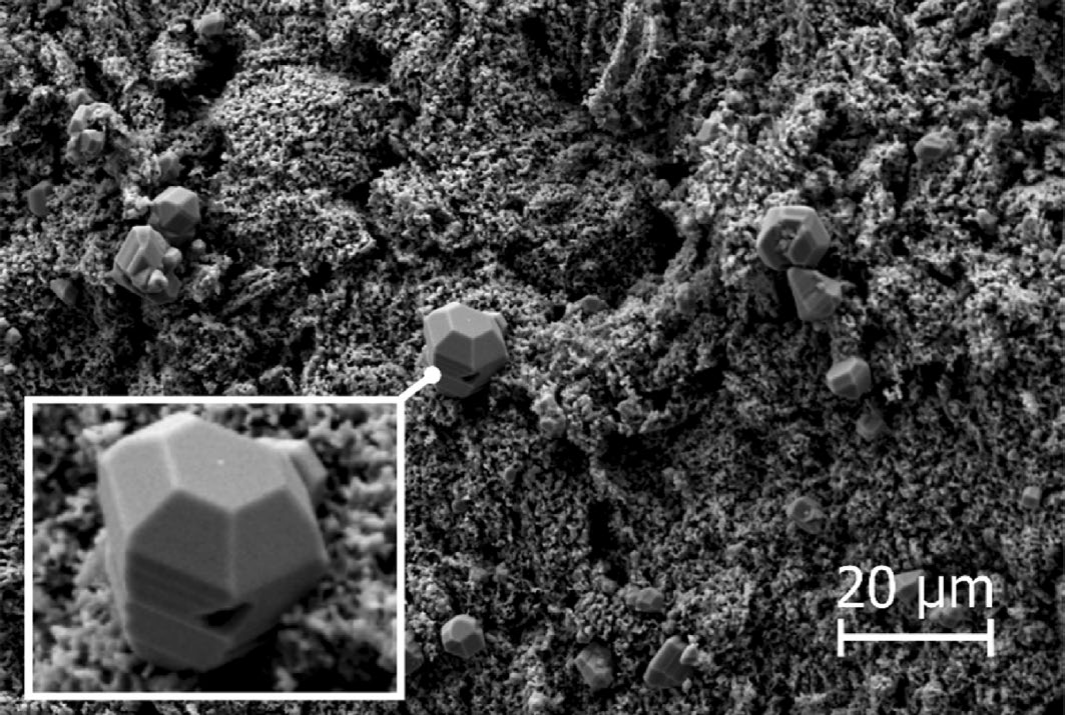
The SiC structure formed at 1750 °C using pure Ar as the process gas.

**Figure 9:**
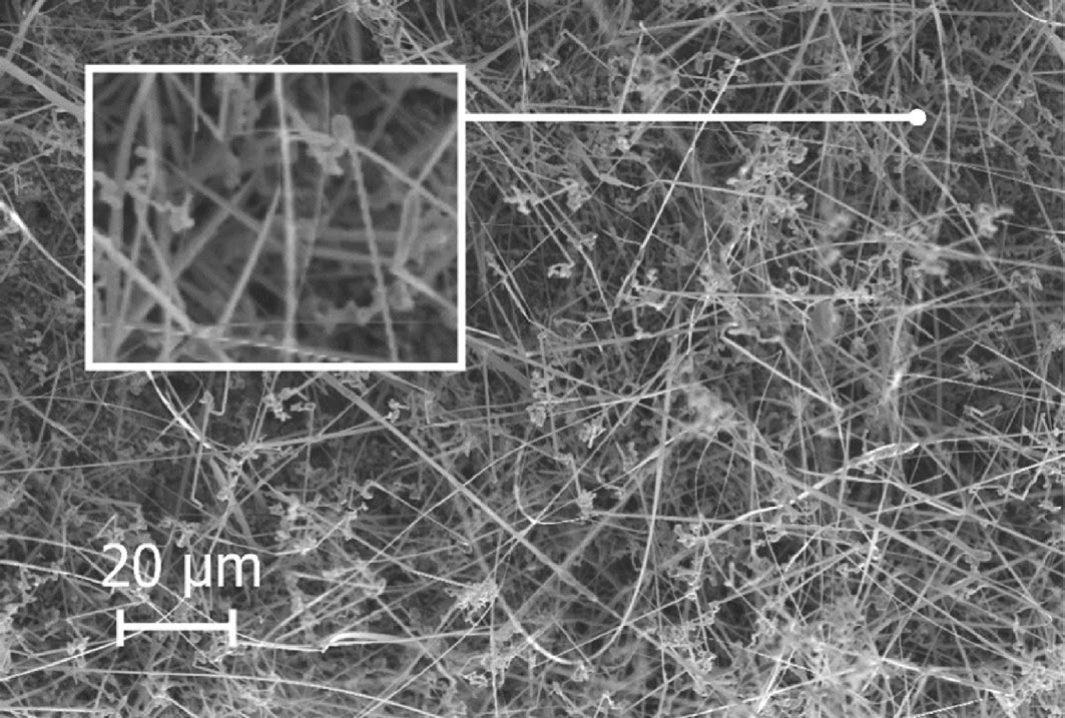
The SiC structure formed at 1650 °C using pure H_2_ as the process gas.

**Figure 10:**
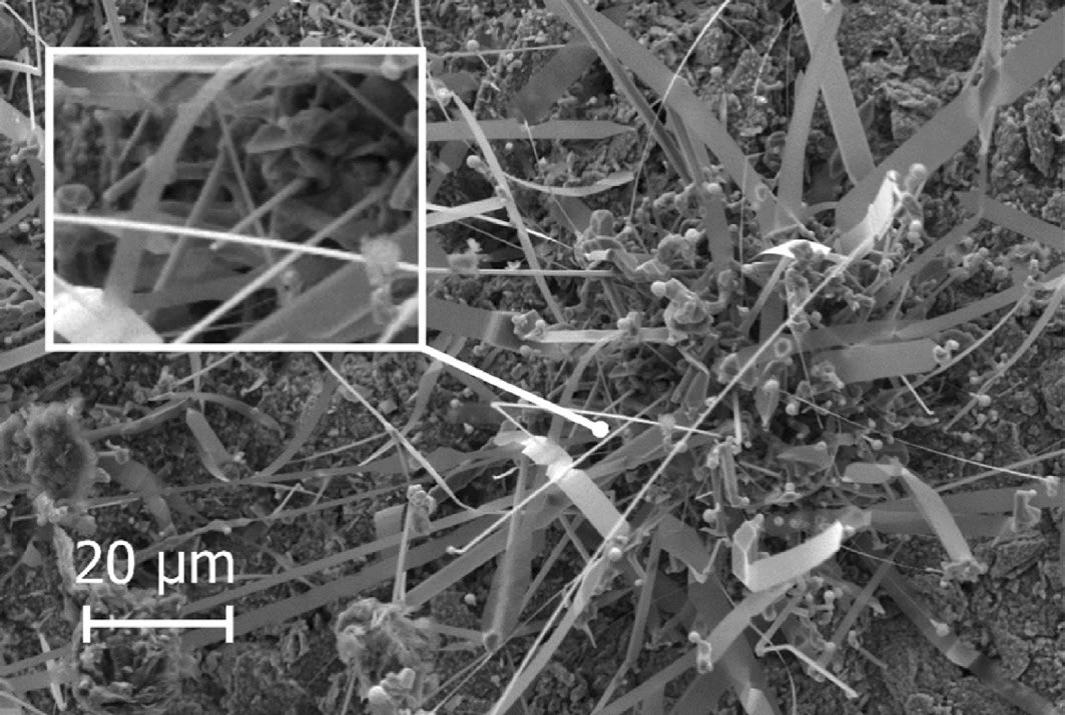
The SiC structure formed at 1750 °C using pure H_2_ as the process gas.

**Figure 11:**
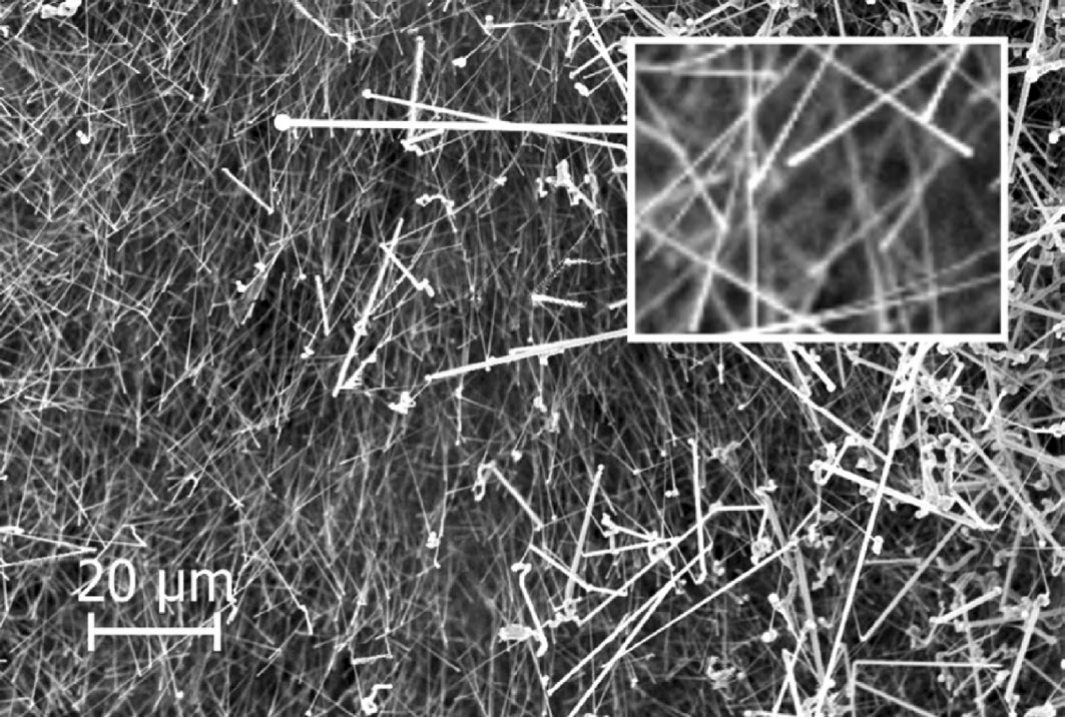
The SiC structure formed at 1650 °C using 2% CH_4_ in H_2_ as the process gas.

**Figure 12:**
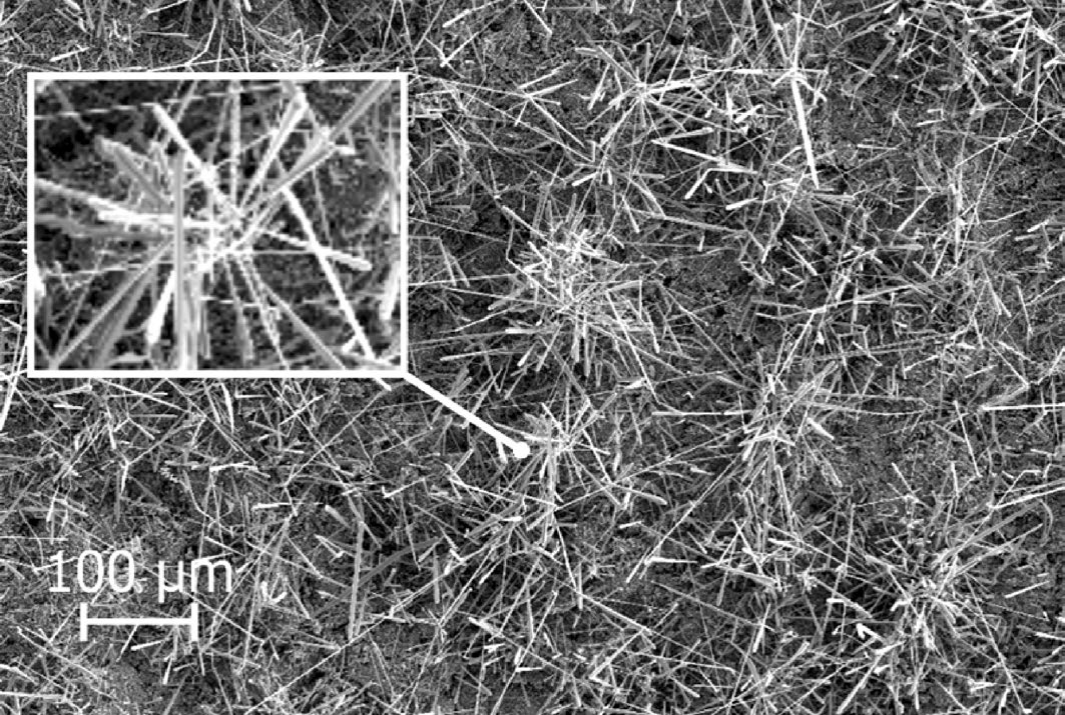
The type of SiC structure formed at 1750 °C using 2% CH_4_ in H_2_ as the process gas.

**Figure 13:**
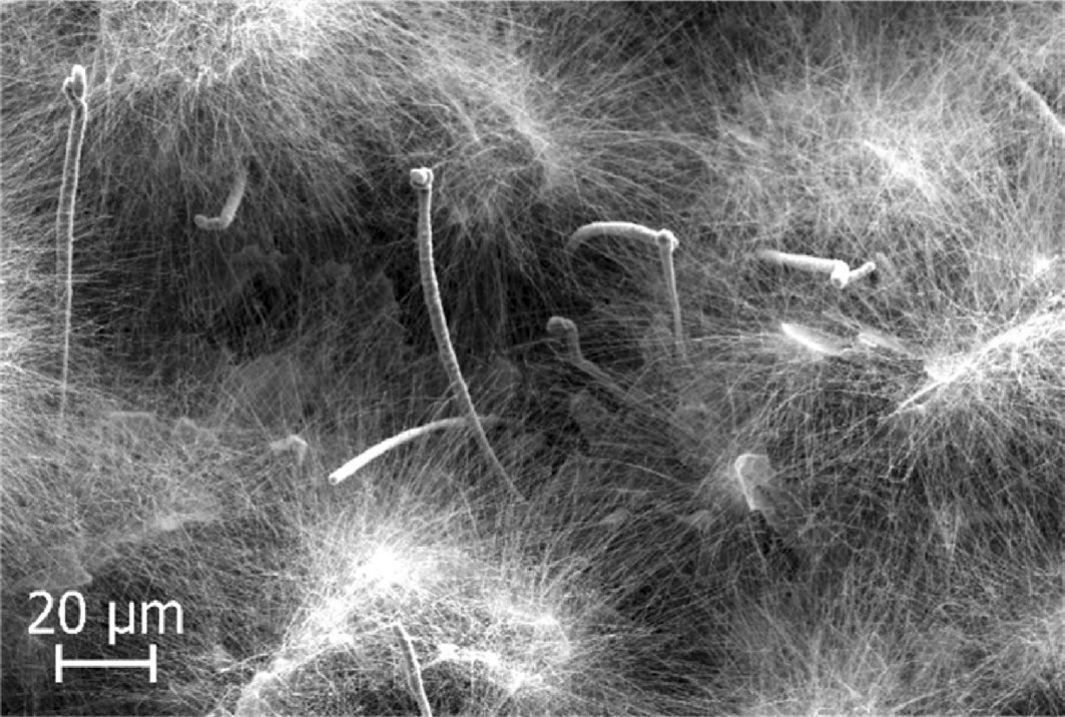
The SiC structure formed in the white region of the crucible at 1650 °C using 5% CH_4_ in H_2_ as the process gas.

**Figure 14:**
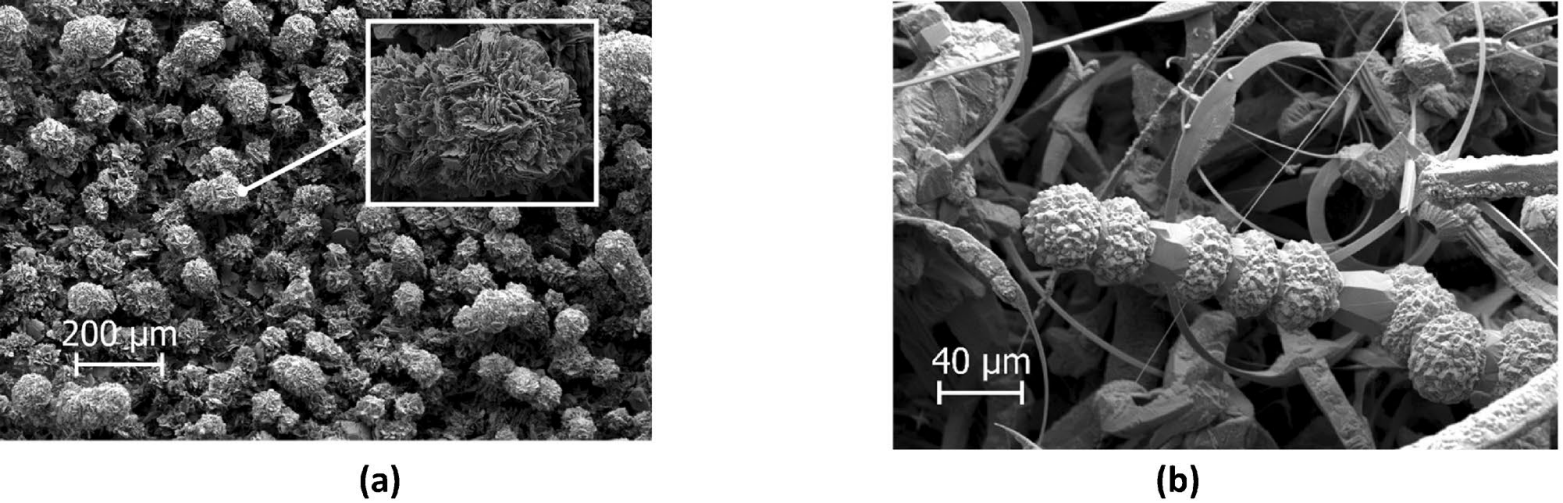
The SiC structures formed in the dark bottom region of the crucible at 1650 °C using 5% CH_4_ in H_2_ as the process gas, **(a)** is from the top of the dark region and **(b)** is a bit further down.

**Figure 15:**
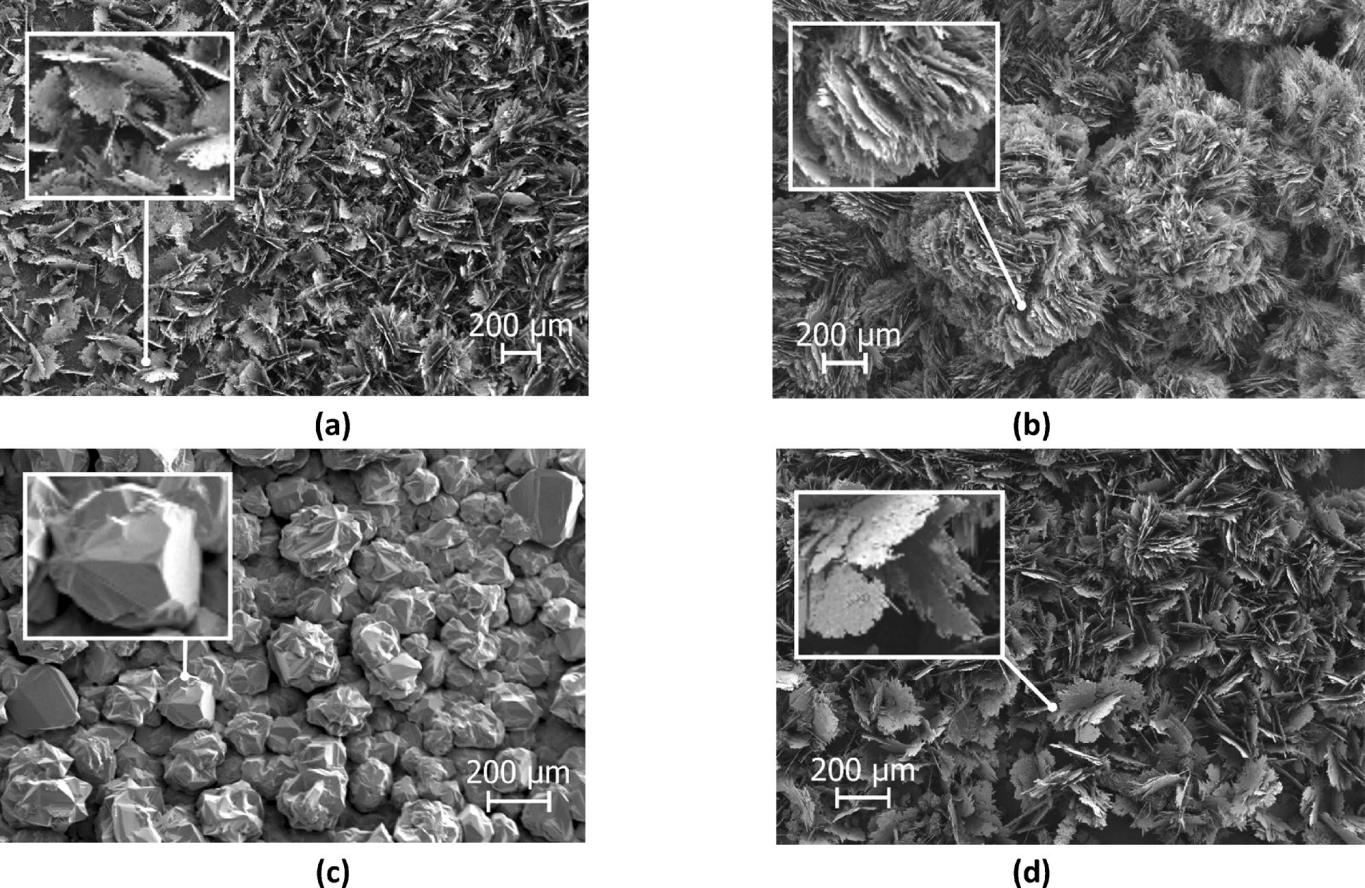
The SiC formed at 1750 °C using 5% CH_4_ in H_2_ as the process gas. **(a)** is from the top of the crucible, **(b)** is from around the middle and **(c)** is from close to the bottom. **(d)** is SiC formed on the alumina lance with the same structure as **(a)** and **(b)**.

Different SiC structures were observed in different gas atmospheres. Figure 7 shows a comparison of the SiC formed on the crucible surfaces. The SiC formed in Ar at 1750 °C is shown Fig. 8. We see a low amount of reaction product. There is a thin layer of SiC formed on the crucible, which was dotted with some larger SiC crystals. Changing the temperature did not have a large effect on the SiC structure or the amount of SiC. When pure Ar was replaced with pure H_2_, SiC whiskers, like those shown in Fig. 9, began forming at 1650 °C. Several researchers have observed similar behaviour^7,10^. The most common explanation is that H_2_ reacts with carbon that is present in the setup, as shown in reaction (5), to form a tiny amount of CH_4_ that enables gas phase mass transfer of carbon to the whisker tips. When the temperature was increased to 1750 °C less overall whisker formation occurred, and what did occur had a lower degree of curviness. The whiskers are shown in Fig. 10. When exchanging the pure H_2_ with H_2_ containing 2% CH_4_ there is a very large increase in whisker formation at 1650 °C. Which can be seen in both Figs. 7e and 11. The increased CH_4_ content allowed for much faster whisker growth. When increasing the temperature to 1750 °C, Fig. 7f shows a decrease in whisker formation to similar levels as in pure H_2_. Examining the crucible in SEM confirmed the presence of whiskers as shown in Fig. 12. Moving on to the highest CH_4_ content that was tested, which was 5%. At 1650 °C the crucible was divided into two regions, see Fig. 7g. The upper region that has a white colour experienced SiC whisker growth, while the bottom region experienced non-whisker SiC growth. Figure 13 shows an image of the upper white region, and Fig. 14a,b shows images of the darker bottom region. When the temperature was increased to 1750 °C SiC whiskers stopped forming. Nevertheless, there were still a large amount of SiC, as can be seen in Fig. 7h, and through the weight measurements discussed earlier. The top of the crucible, Fig. 15a, had flakes spread evenly on the crucible surface. Moving down there were still flakes but they, instead, formed as large clusters, Fig. 15b. Farther down it transitioned into larger SiC crystals with clearly visible crystal faces, Fig. 15c. However, there were no sudden changes, the transition happened gradually. Both the temperature and partial pressures of the various gasses varies with the height direction in the reaction chamber. The temperature appears to play a big role in allowing whisker growth. A type of whisker growth occurring in the Si–C–O–H system is oxide assisted growth, where an outer SiO_2_ layer guides the whisker while it is growing^14^**.** At 1750 °C the temperature is above the melting point of SiO_2_ which would effectively inhibit this growth mechanism.

Through examining the reaction products it was possible to confirm the formation of SiC from CH_4_ and SiO. Figure 15d is a SEM micrograph of the lance. The flaky structure covering it is comprised of SiC, which was determined by EDS. The lance was made from alumina, which means that if carbon is detected on the lance it must come from the gas phase. Additionally, the SiC only formed on the lance when a CH_4_ containing process gas was used which further corroborates the SiC having formed through reaction (6). Comparing the structure to the one from Fig. 15a which shows a SEM micrograph of the crucible from the same experiment shows that it is also covered in SiC. What’s more, is that the structure bears a striking resemblance to what formed on the lance. Indicating that the SiC forming within the crucible is produced through the same mechanism. An interesting finding considering that 5% CH_4_ is the equilibrium pressure of CH_4_ at around 700 °C, yet it is still able to partake in reactions far away from its equilibrium. Another interesting aspect of using CH_4_ to produce SiC is that the reaction is endothermic, while the usual reaction between carbon and SiO gas is exothermic. As a result, CH_4_ could be used to control the temperature in a silicon furnace, which would allow for increased control over SiO gas losses, and thus much greater energy efficiency.

## Conclusions

A reaction between SiO and CH_4_ has been confirmed. Using a process gas consisting of 5% CH_4_ in H_2_ allowed at least 22% of the SiO to be captured through a reaction with CH_4_. This is highly interesting as the temperature of the current experiments were roughly 1000 °C higher than equilibrium for CH_4_.

It was also seen that the reaction rate of the SiC/SiO_2_ pellets proceeds faster in hydrogen containing atmospheres. The effect was especially large after the melting point of SiO_2_. An increased reaction rate of 57% and 161% was calculated for 1650 °C and 1750 °C respectively.

The SiC structure that formed was highly dependent on both the temperature and what process gas was used. When Ar was used it grew as a dense SiC layer dotted with larger SiC crystals. While when H_2_ and low amounts of CH_4_ was used the SiC grew as thin whiskers, with the amount of whiskers increasing up to 2% CH_4_. However, increasing the temperature from 1650 to 1750 °C led to a very large reduction in whisker formation. At 5% CH_4_ in H_2_ a wide variety of different SiC structures, in addition to whiskers, was observed.

## Supplementary Information

Supplementary Information.
