# Supplementary material for: Silicon carbide formation from methane and silicon monoxide
Source: Sci Rep. 2020 Dec 11;10:21831. doi: 10.1038/s41598-020-79006-6 (PMC7733506; doi:10.1038/s41598-020-79006-6)
Supplement: Supplementary file 1 — Supplementary Information. [file 41598_2020_79006_MOESM1_ESM.pdf]

Silicon Carbide Formation from Methane and Silicon Monoxide

Trygve Storm Aarnæs*[1], Eli Ringdalen[2], Merete Tangstad[3]

[1,3]Department of Materials Science and Engineering, Norwegian University of Science and Technology, Trondheim, Norway

[2]Metal Production and Processing, SINTEF Industry, Trondheim, Norway

[1]trygve.s.aarnas@ntnu.no, [2]Eli.Ringdalen@sintef.no, [3]merete.tangstad@ntnu.no

Contents

Notes: 1
100% Ar at 1750^oC 2
100% H_2 at 1650^oC 17
100% H_2 at 1750^oC 32
2% CH_4 in H_2 at 1650^oC 47
2% CH_4 in H_2 at 1750^oC 60
5% CH_4 in H_2 at 1650^oC 103
5% CH_4 in H_2 at 1750^oC 110
5% CH_4 in H_2 at 1750^oC, SiC from the alumina gas lance 135

Notes:

The SEM was operated with an acceleration voltage of 10kV during this investigation. As a result the emission depth for characteristic x-rays will be around 1 – 1.5 µm, calculated by Castaing's formula.

Due to the nature of the samples, the EDS spots often does not have a flat and continuous surface that is parallel to the incoming electron beam, as such the uncertainty in each individual measurement is expected to be high. However, a large amount of spots was measured to remedy this.

100% Ar at 1750°C

Area 1

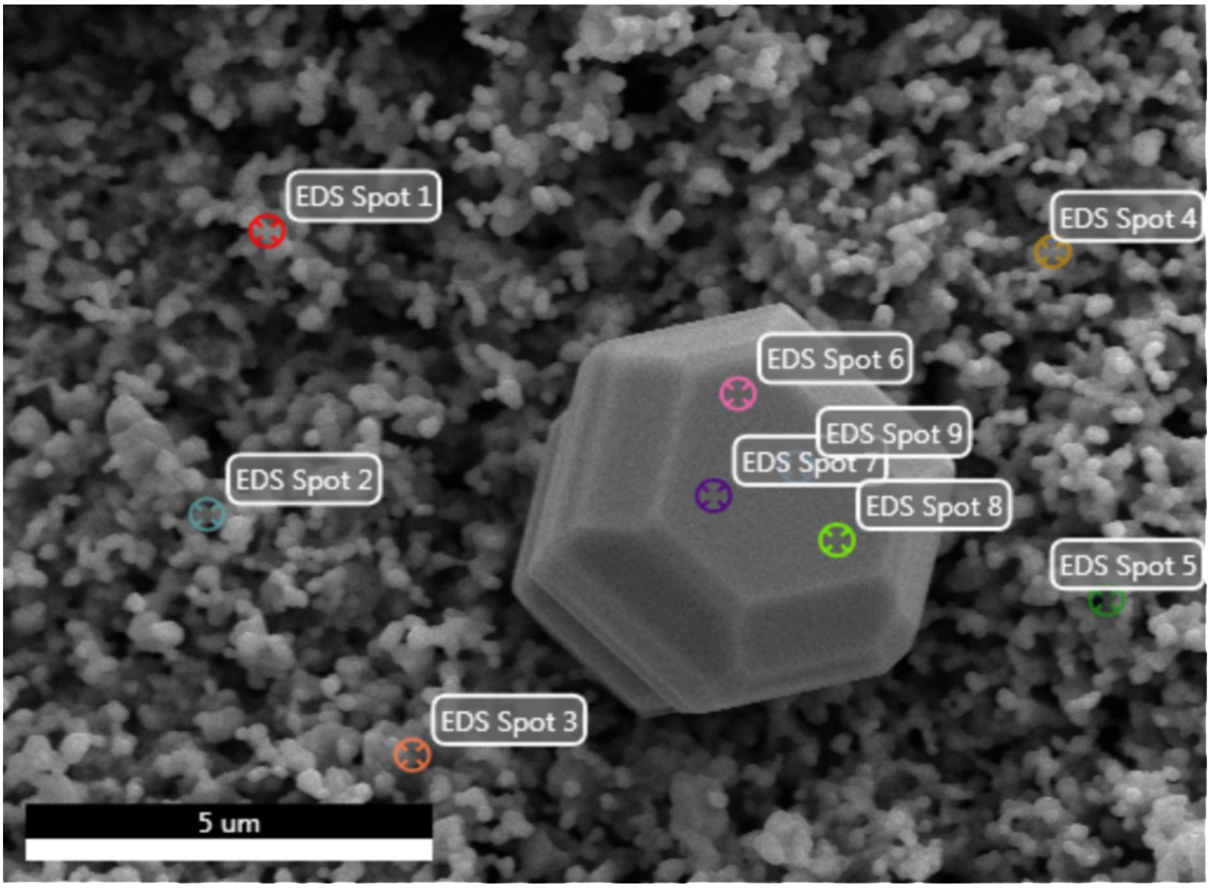

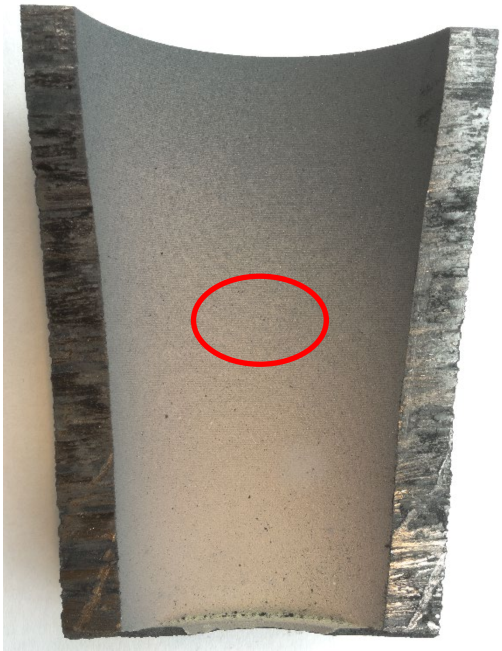

EDS Spot 1

kV: 10	Mag: 7431	Takeoff: 37.8	Live Time(s): 29.9	Amp Time(µs): 1.92	Resolution:(eV) 127.4

EDS Spot 1 - Det 1

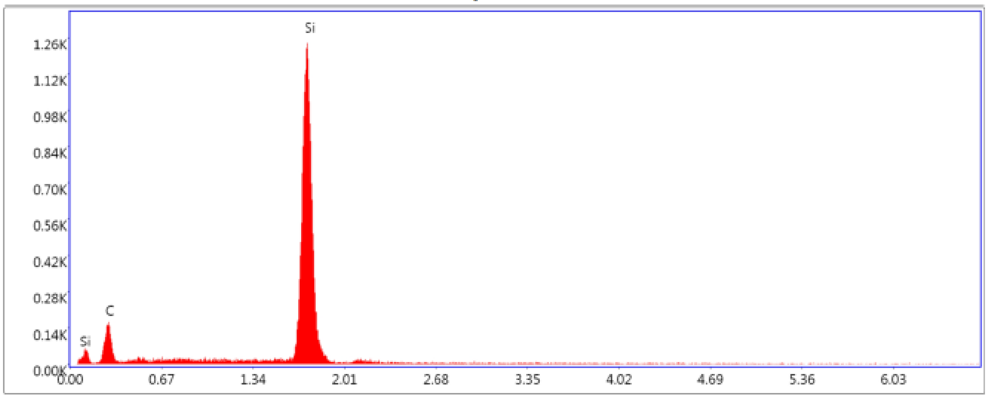

Lsec: 29.9 0 Cnts 0.000 keV Det: Octane Pro A

eZAF Smart Quant Results

Element	Weight %	Atomic %	Net Int.	Error %	Kratio	Z	A	F
C K	32.46	52.91	53.97	13.56	0.0610	1.1186	0.1680	1.0000
SiK	67.54	47.09	687.82	2.91	0.6321	0.9399	0.9956	1.0003

EDS Spot 2

kV: 10	Mag: 7431	Takeoff: 37.8	Live Time(s): 29.8	Amp Time(µs): 1.92	Resolution:(eV) 127.4

EDS Spot 2 - Det 1

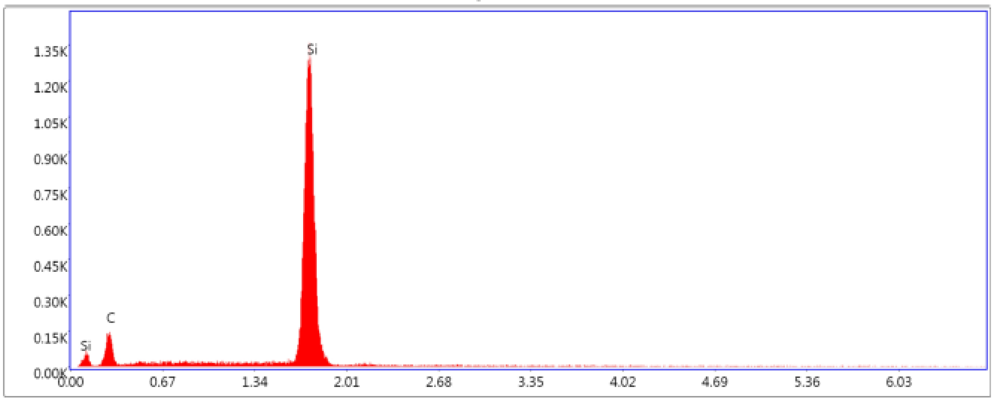

Lsec: 29.8 0 Cnts 0.000 keV Det: Octane Pro A

eZAF Smart Quant Results

Element	Weight %	Atomic %	Net Int.	Error %	Kratio	Z	A	F
C K	29.53	49.49	48.46	13.77	0.0535	1.1245	0.1611	1.0000
SiK	70.47	50.51	739.35	2.87	0.6637	0.9451	0.9960	1.0002

EDS Spot 3

kV: 10	Mag: 7431	Takeoff: 37.8	Live Time(s): 29.8	Amp Time(µs): 1.92	Resolution:(eV) 127.4

EDS Spot 3 - Det 1

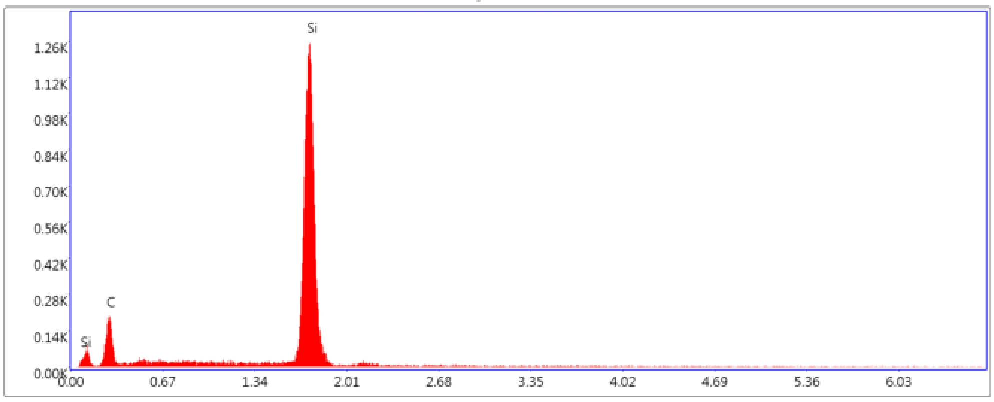

Lsec: 29.8 0 Cnts 0.000 keV Det: Octane Pro A

eZAF Smart Quant Results

Element	Weight %	Atomic %	Net Int.	Error %	Kratio	Z	A	F
C K	34.62	55.32	63.47	13.28	0.0669	1.1144	0.1735	1.0000
SiK	65.38	44.68	710.65	2.93	0.6092	0.9361	0.9953	1.0003

EDS Spot 4

kV: 10	Mag: 7431	Takeoff: 37.8	Live Time(s): 29.8	Amp Time(µs): 1.92	Resolution:(eV) 127.4

EDS Spot 4 - Det 1

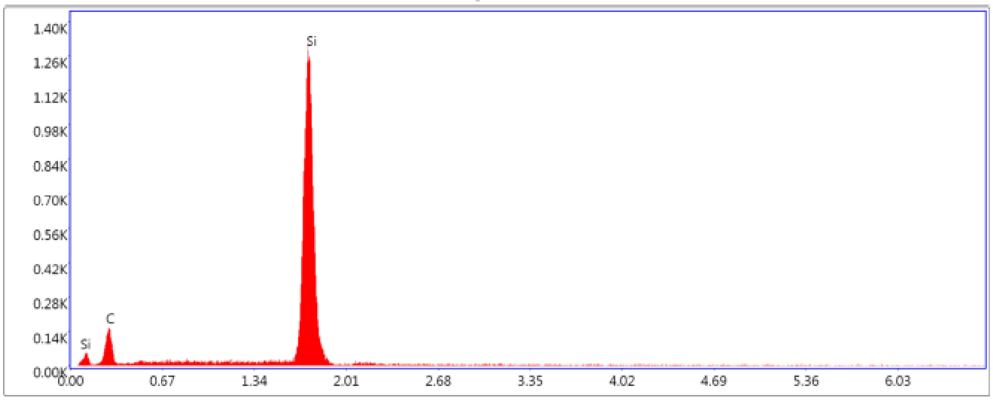

Lsec: 29.8 0 Cnts 0.000 keV Det: Octane Pro A

eZAF Smart Quant Results

Element	Weight %	Atomic %	Net Int.	Error %	Kratio	Z	A	F
C K	30.78	50.97	51.12	13.66	0.0566	1.1220	0.1640	1.0000
SiK	69.22	49.03	722.00	2.88	0.6503	0.9429	0.9958	1.0003

EDS Spot 5

kV: 10	Mag: 7431	Takeoff: 37.8	Live Time(s): 29.9	Amp Time(μs): 1.92	Resolution:(eV) 127.4

EDS Spot 5 - Det 1

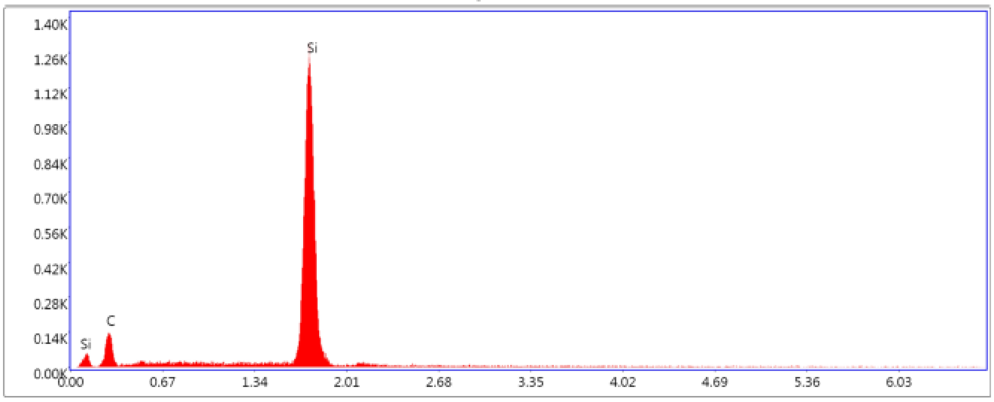

Lsec: 29.9   0 Cnts   0.000 keV   Det: Octane Pro A

eZAF Smart Quant Results

Element	Weight %	Atomic %	Net Int.	Error %	Kratio	Z	A	F
C K	30.44	50.58	47.70	13.78	0.0558	1.1227	0.1632	1.0000
SiK	69.56	49.42	687.83	2.92	0.6539	0.9435	0.9959	1.0003

EDS Spot 6

kV: 10	Mag: 7431	Takeoff: 37.8	Live Time(s): 29.8	Amp Time(μs): 1.92	Resolution:(eV) 127.4

EDS Spot 6 - Det 1

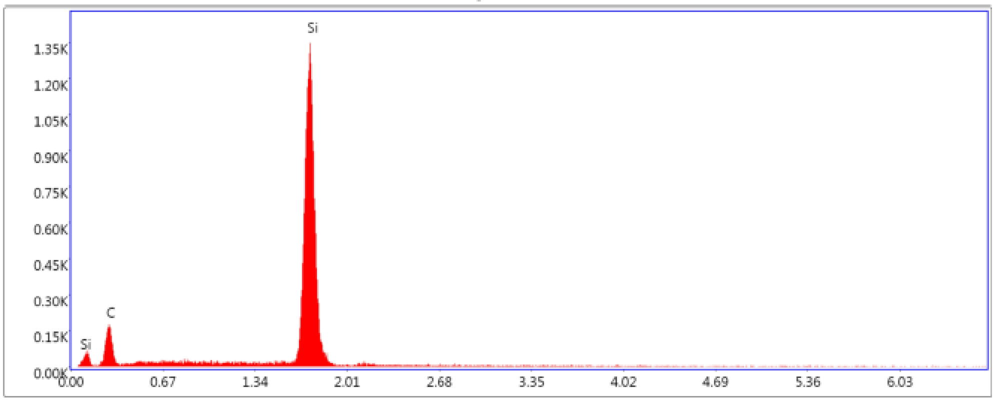

Lsec: 29.8   0 Cnts   0.000 keV   Det: Octane Pro A

eZAF Smart Quant Results

Element	Weight %	Atomic %	Net Int.	Error %	Kratio	Z	A	F
C K	32.95	53.47	58.35	13.42	0.0623	1.1177	0.1693	1.0000
SiK	67.05	46.53	721.96	2.91	0.6269	0.9390	0.9956	1.0003

EDS Spot 7

kV: 10	Mag: 7431	Takeoff: 37.8	Live Time(s): 29.9	Amp Time(µs): 1.92	Resolution:(eV) 127.4

EDS Spot 7 - Det 1

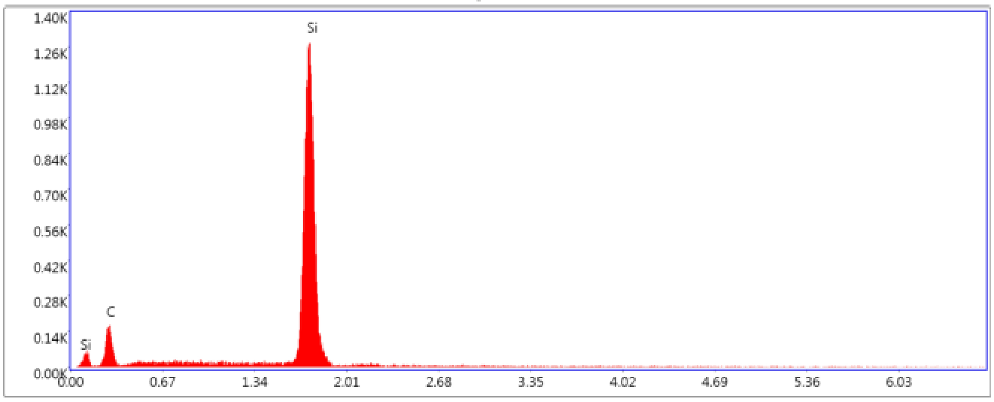

Lsec: 29.9 0 Cnts 0.000 keV Det: Octane Pro A

eZAF Smart Quant Results

Element	Weight %	Atomic %	Net Int.	Error %	Kratio	Z	A	F
C K	32.31	52.74	55.88	13.49	0.0606	1.1189	0.1677	1.0000
SiK	67.69	47.26	718.67	2.91	0.6337	0.9401	0.9956	1.0003

EDS Spot 8

kV: 10	Mag: 7431	Takeoff: 37.8	Live Time(s): 29.8	Amp Time(µs): 1.92	Resolution:(eV) 127.4

EDS Spot 8 - Det 1

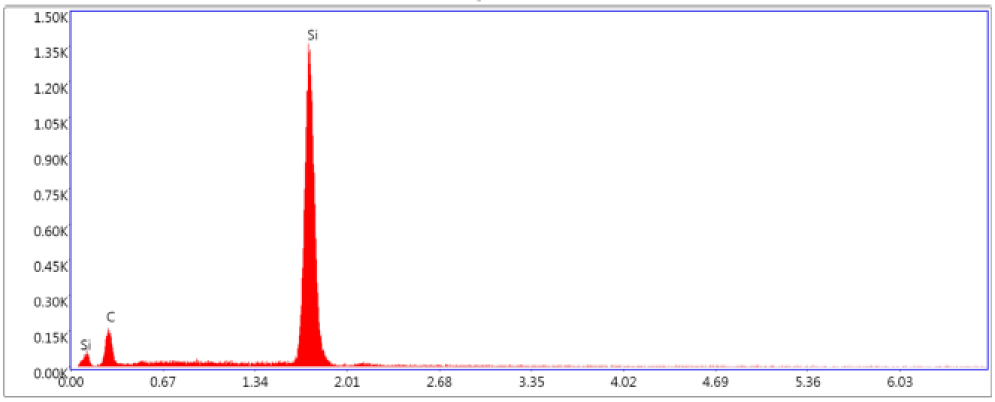

Lsec: 29.8 0 Cnts 0.000 keV Det: Octane Pro A

eZAF Smart Quant Results

Element	Weight %	Atomic %	Net Int.	Error %	Kratio	Z	A	F
C K	30.60	50.77	51.85	14.01	0.0562	1.1224	0.1635	1.0000
SiK	69.40	49.23	740.30	2.90	0.6522	0.9432	0.9959	1.0003

EDS Spot 9

kV: 10	Mag: 7431	Takeoff: 37.8	Live Time(s): 29.8	Amp Time(µs): 1.92	Resolution:(eV) 127.4

EDS Spot 9 - Det 1

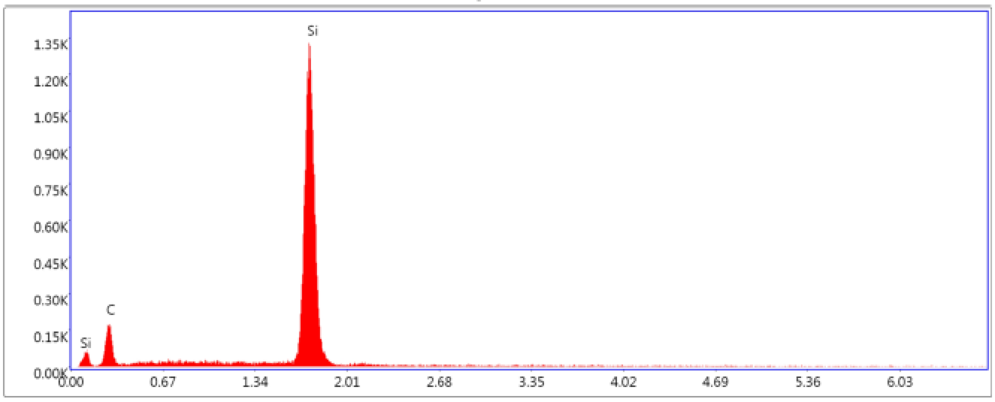

Lsec: 29.8 0 Cnts 0.000 keV Det: Octane Pro A

eZAF Smart Quant Results

Element	Weight %	Atomic %	Net Int.	Error %	Kratio	Z	A	F
C K	32.86	53.37	58.60	13.43	0.0621	1.1178	0.1690	1.0000
SiK	67.14	46.63	729.16	2.87	0.6279	0.9392	0.9956	1.0003

Area 2

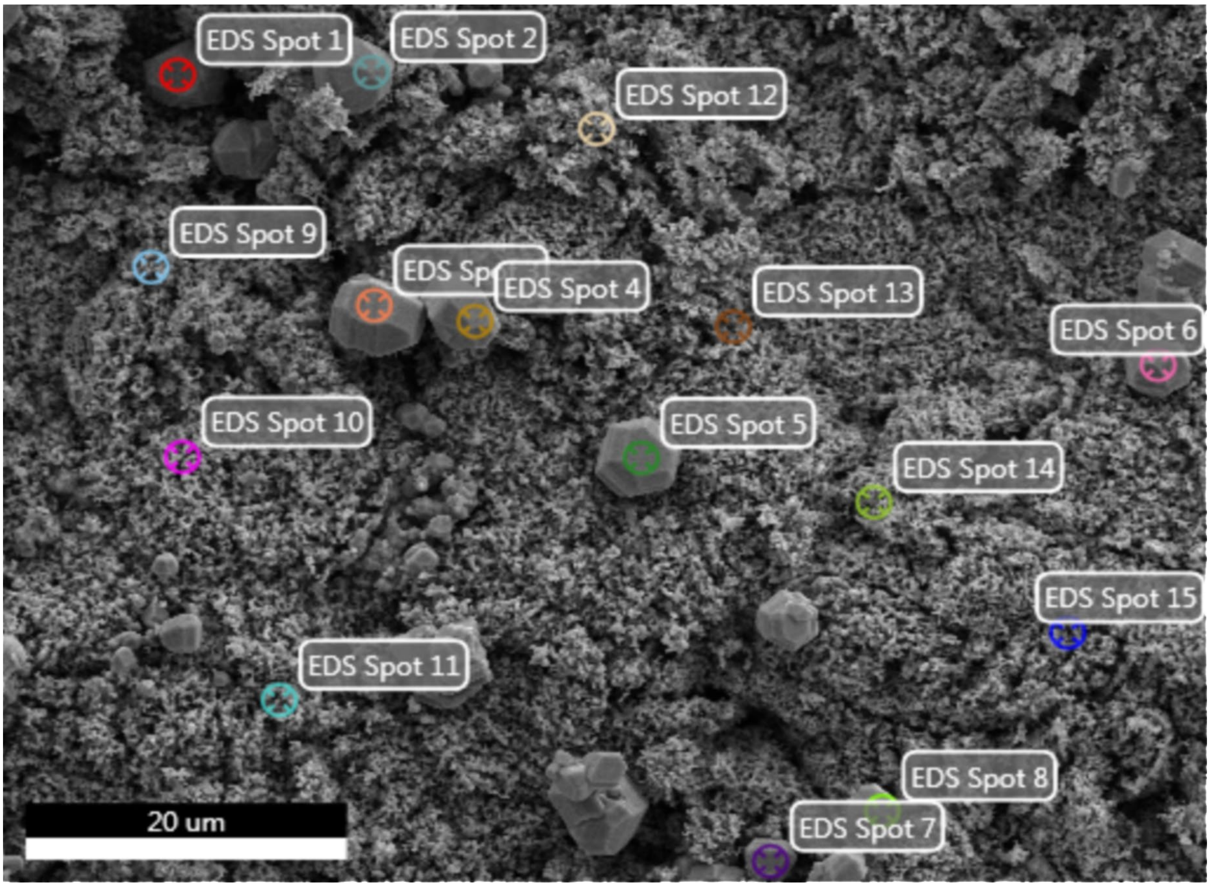
EDS Spot 1
EDS Spot 2
EDS Spot 12
EDS Spot 9
EDS Spot 4
EDS Spot 13
EDS Spot 6
EDS Spot 10
EDS Spot 5
EDS Spot 14
EDS Spot 15
EDS Spot 11
EDS Spot 8
EDS Spot 7
20 um

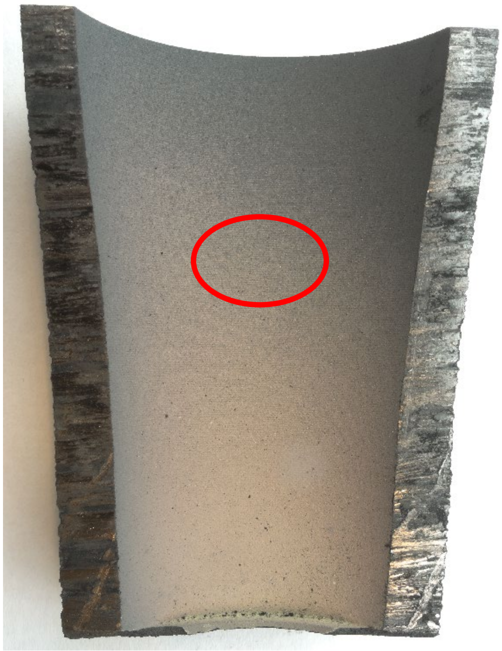

EDS Spot 1

kV: 10	Mag: 1467	Takeoff: 37.8	Live Time(s): 29.9	Amp Time(µs): 1.92	Resolution:(eV) 127.4

EDS Spot 1 - Det 1

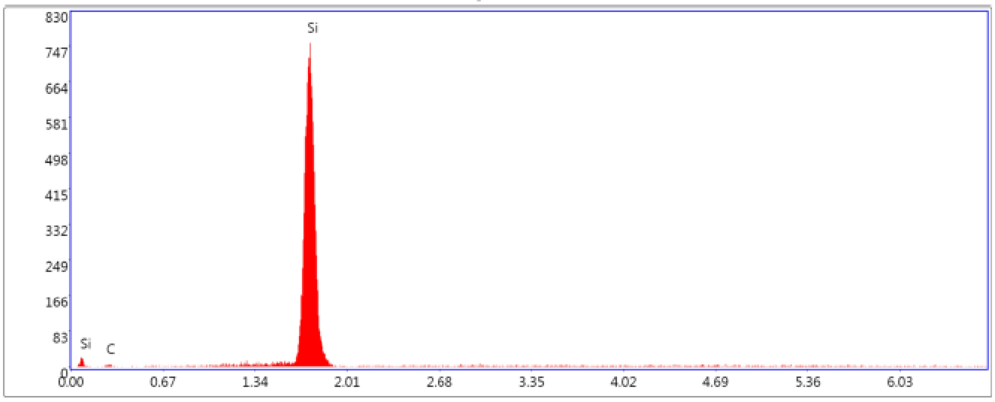

Lsec: 29.9 0 Cnts 0.000 keV Det: Octane Pro A

eZAF Smart Quant Results

Element	Weight %	Atomic %	Net Int.	Error %	Kratio	Z	A	F
C K	1.73	3.95	0.83	85.55	0.0024	1.1827	0.1155	1.0000
SiK	98.27	96.05	421.69	3.03	0.9792	0.9966	0.9998	1.0000

EDS Spot 2

kV: 10	Mag: 1467	Takeoff: 37.8	Live Time(s): 29.9	Amp Time(µs): 1.92	Resolution:(eV) 127.4

EDS Spot 2 - Det 1

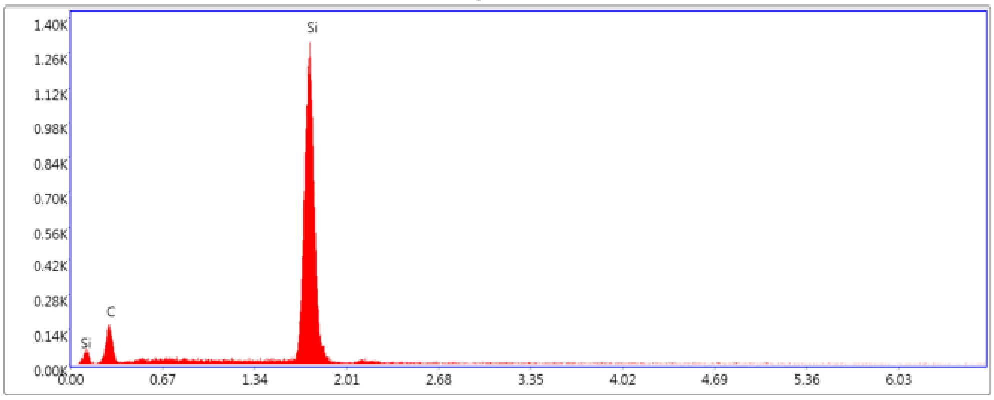

Lsec: 29.9 0 Cnts 0.000 keV Det: Octane Pro A

eZAF Smart Quant Results

Element	Weight %	Atomic %	Net Int.	Error %	Kratio	Z	A	F
C K	32.02	52.42	54.63	13.52	0.0599	1.1196	0.1669	1.0000
SiK	67.98	47.58	715.30	2.89	0.6371	0.9407	0.9957	1.0003

EDS Spot 3

kV: 10	Mag: 1467	Takeoff: 37.8	Live Time(s): 29.8	Amp Time(μs): 1.92	Resolution:(eV) 127.4

EDS Spot 3 - Det 1

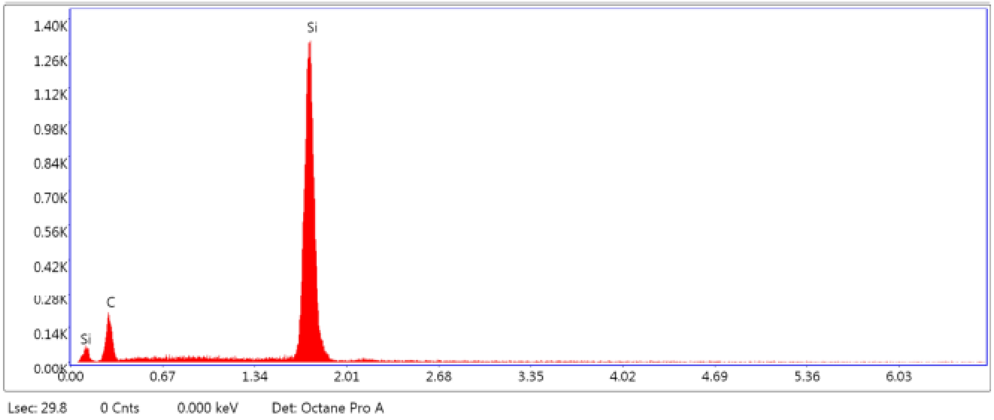

eZAF Smart Quant Results

Element	Weight %	Atomic %	Net Int.	Error %	Kratio	Z	A	F
C K	33.60	54.20	61.36	13.32	0.0641	1.1164	0.1709	1.0000
SiK	66.40	45.80	730.03	2.89	0.6200	0.9379	0.9955	1.0003

EDS Spot 4

kV: 10	Mag: 1467	Takeoff: 37.8	Live Time(s): 29.8	Amp Time(μs): 1.92	Resolution:(eV) 127.4

EDS Spot 4 - Det 1

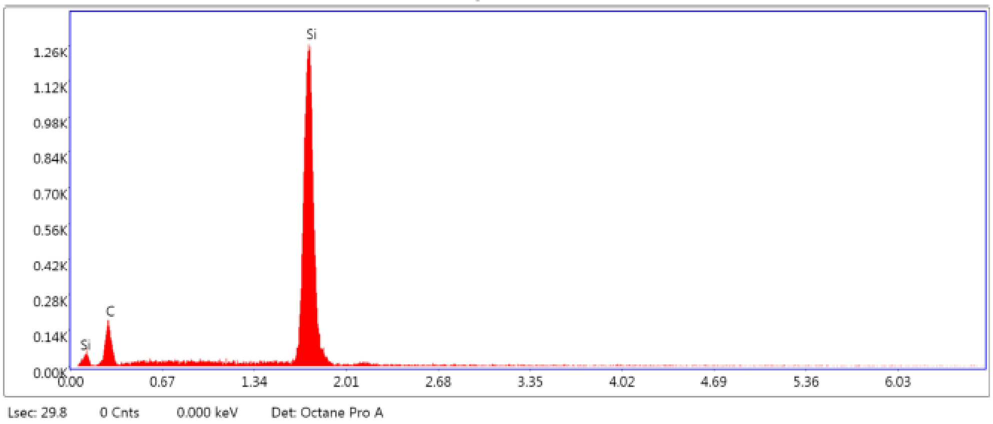

eZAF Smart Quant Results

Element	Weight %	Atomic %	Net Int.	Error %	Kratio	Z	A	F
C K	32.29	52.72	55.89	13.50	0.0606	1.1190	0.1676	1.0000
SiK	67.71	47.28	719.67	2.89	0.6339	0.9402	0.9956	1.0003

EDS Spot 5

kV: 10	Mag: 1467	Takeoff: 37.8	Live Time(s): 29.8	Amp Time(µs): 1.92	Resolution:(eV) 127.4

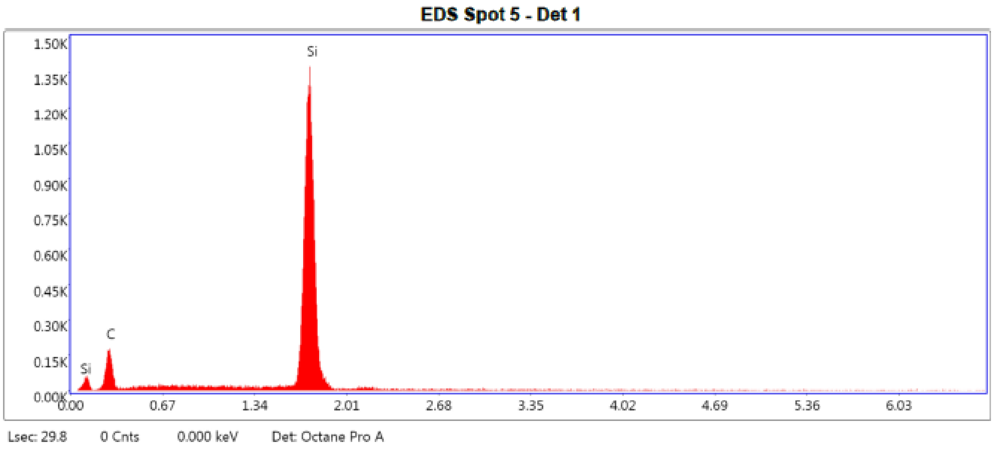

eZAF Smart Quant Results

Element	Weight %	Atomic %	Net Int.	Error %	Kratio	Z	A	F
C K	32.82	53.33	58.89	13.42	0.0620	1.1179	0.1689	1.0000
SiK	67.18	46.67	734.25	2.88	0.6283	0.9392	0.9956	1.0003

EDS Spot 6

kV: 10	Mag: 1467	Takeoff: 37.8	Live Time(s): 29.8	Amp Time(µs): 1.92	Resolution:(eV) 127.4

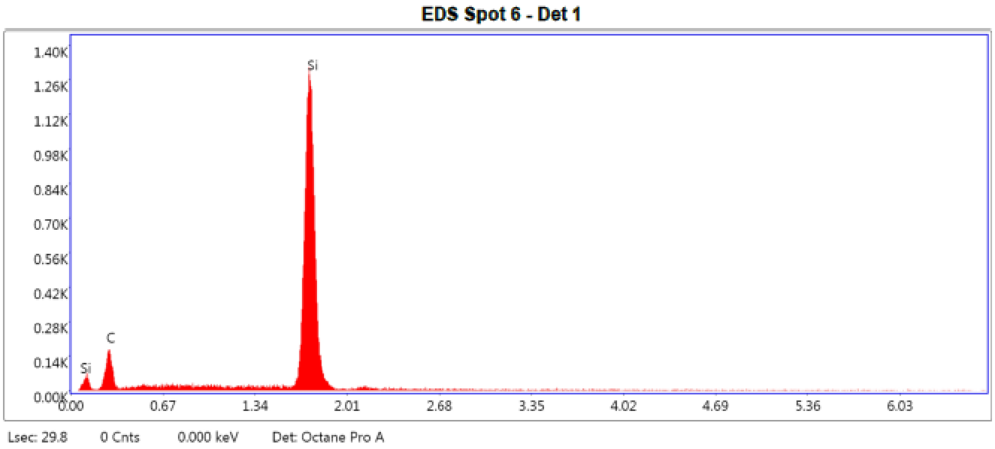

eZAF Smart Quant Results

Element	Weight %	Atomic %	Net Int.	Error %	Kratio	Z	A	F
C K	32.54	53.00	57.60	13.45	0.0612	1.1185	0.1682	1.0000
SiK	67.46	47.00	730.59	2.89	0.6313	0.9397	0.9956	1.0003

EDS Spot 7

kV: 10	Mag: 1467	Takeoff: 37.8	Live Time(s): 29.9	Amp Time(µs): 1.92	Resolution:(eV) 127.4

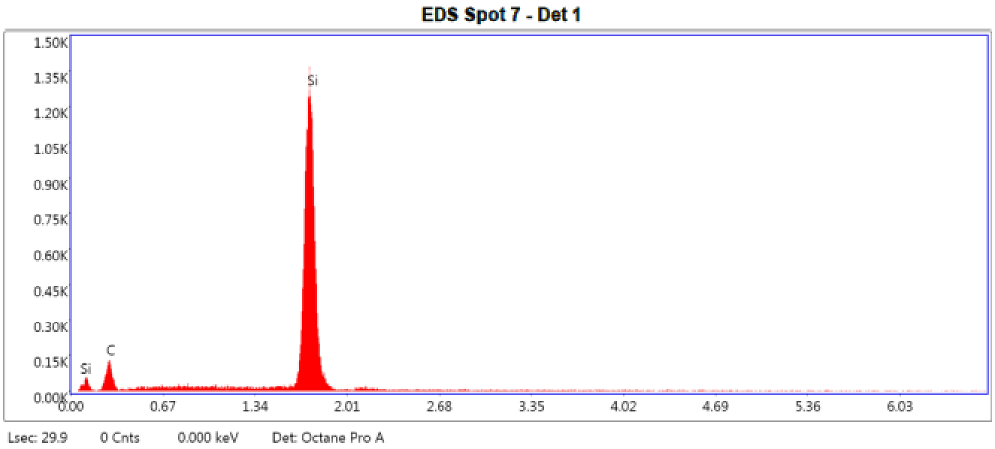

eZAF Smart Quant Results

Element	Weight %	Atomic %	Net Int.	Error %	Kratio	Z	A	F
C K	26.39	45.61	39.14	15.02	0.0461	1.1308	0.1543	1.0000
SiK	73.61	54.39	728.97	2.88	0.6975	0.9507	0.9964	1.0002

EDS Spot 8

kV: 10	Mag: 1467	Takeoff: 37.8	Live Time(s): 29.8	Amp Time(µs): 1.92	Resolution:(eV) 127.4

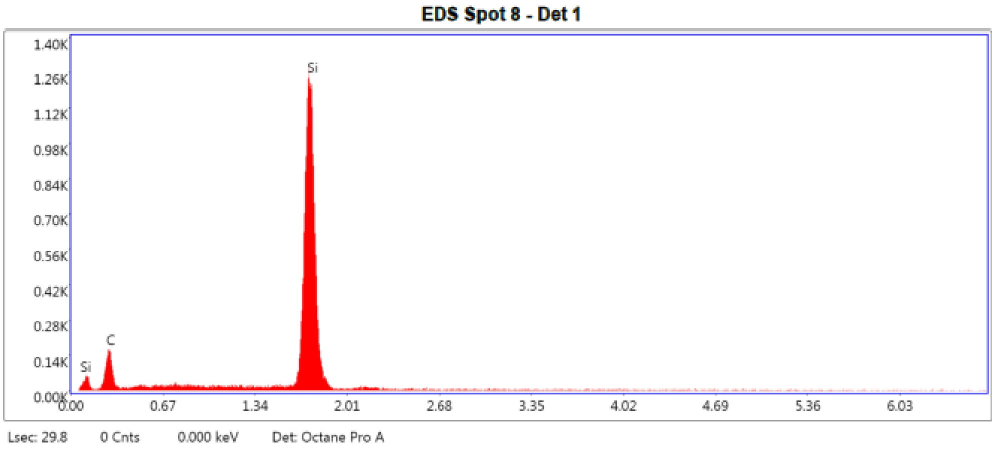

eZAF Smart Quant Results

Element	Weight %	Atomic %	Net Int.	Error %	Kratio	Z	A	F
C K	31.97	52.36	54.41	13.55	0.0597	1.1197	0.1668	1.0000
SiK	68.03	47.64	714.54	2.90	0.6376	0.9408	0.9957	1.0003

EDS Spot 9

kV: 10	Mag: 1467	Takeoff: 37.8	Live Time(s): 29.8	Amp Time(μs): 1.92	Resolution:(eV) 127.4

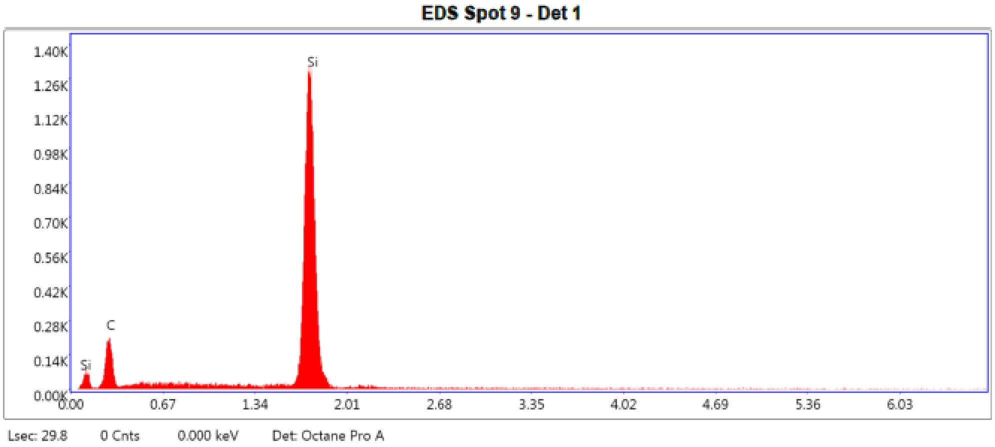

eZAF Smart Quant Results

Element	Weight %	Atomic %	Net Int.	Error %	Kratio	Z	A	F
C K	36.80	57.65	75.53	12.99	0.0732	1.1101	0.1794	1.0000
SiK	63.20	42.35	743.79	2.89	0.5863	0.9323	0.9950	1.0003

EDS Spot 10

kV: 10	Mag: 1467	Takeoff: 37.8	Live Time(s): 29.8	Amp Time(μs): 1.92	Resolution:(eV) 127.4

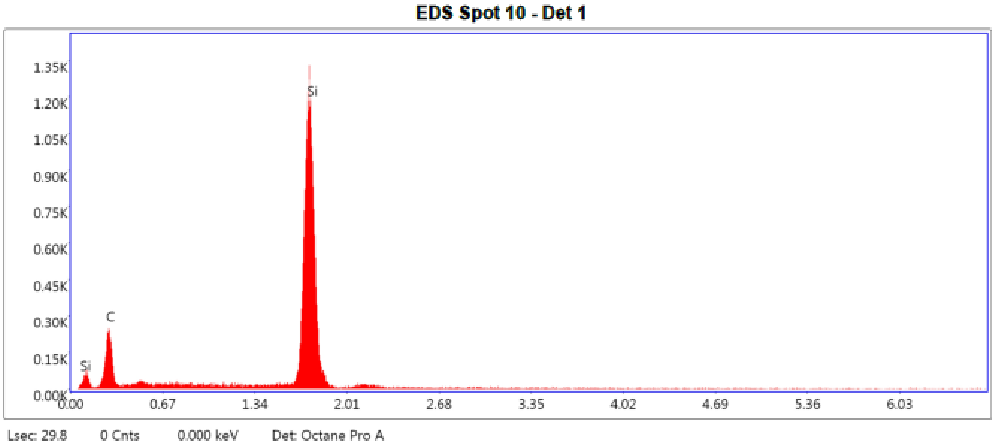

eZAF Smart Quant Results

Element	Weight %	Atomic %	Net Int.	Error %	Kratio	Z	A	F
C K	40.29	61.20	86.69	12.52	0.0842	1.1034	0.1894	1.0000
SiK	59.71	38.80	697.25	2.94	0.5505	0.9264	0.9946	1.0004

EDS Spot 11

kV: 10	Mag: 1467	Takeoff: 37.8	Live Time(s): 29.9	Amp Time(µs): 1.92	Resolution:(eV) 127.4

EDS Spot 11 - Det 1

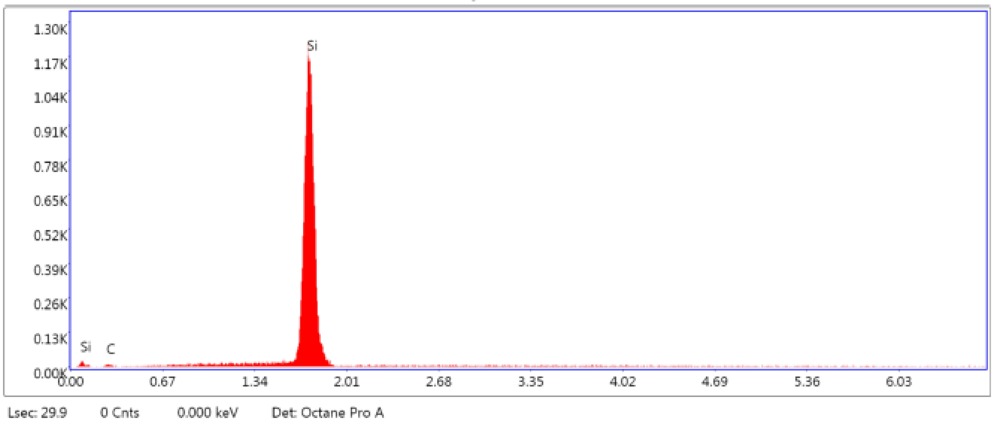

eZAF Smart Quant Results

Element	Weight %	Atomic %	Net Int.	Error %	Kratio	Z	A	F
C K	0.39	0.90	0.29	99.99	0.0005	1.1857	0.1139	1.0000
SiK	99.61	99.10	668.45	2.84	0.9953	0.9992	0.9999	1.0000

EDS Spot 12

kV: 10	Mag: 1467	Takeoff: 37.8	Live Time(s): 29.9	Amp Time(µs): 1.92	Resolution:(eV) 127.4

EDS Spot 12 - Det 1

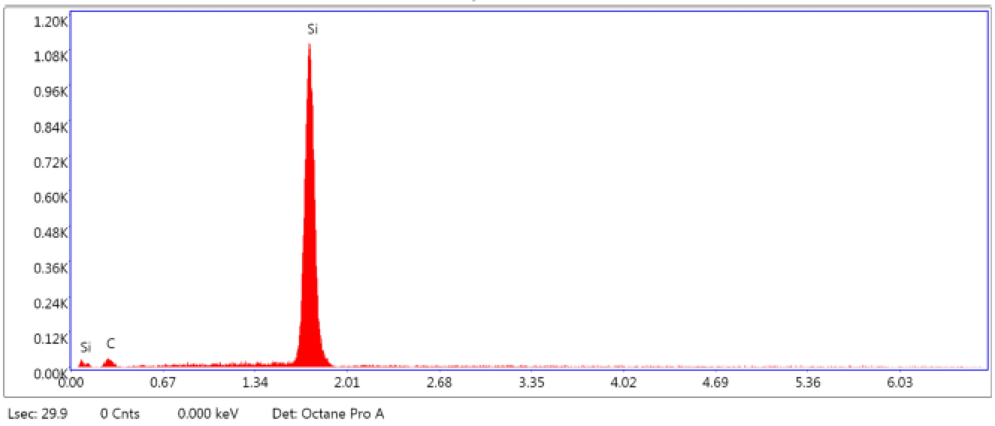

eZAF Smart Quant Results

Element	Weight %	Atomic %	Net Int.	Error %	Kratio	Z	A	F
C K	10.29	21.14	9.65	22.28	0.0152	1.1642	0.1266	1.0000
SiK	89.71	78.86	687.69	2.84	0.8783	0.9803	0.9986	1.0001

EDS Spot 13

kV: 10	Mag: 1467	Takeoff: 37.8	Live Time(s): 29.8	Amp Time(μs): 1.92	Resolution:(eV) 127.4

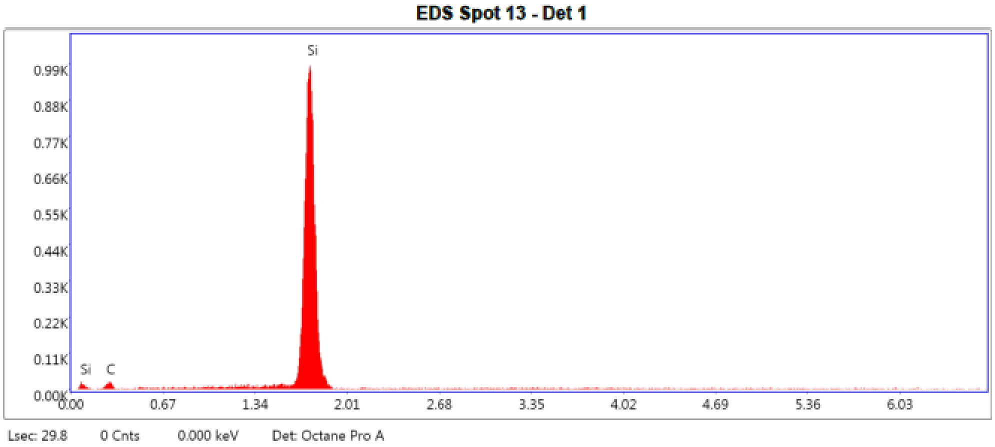

eZAF Smart Quant Results

Element	Weight %	Atomic %	Net Int.	Error %	Kratio	Z	A	F
C K	8.30	17.46	6.86	23.75	0.0120	1.1685	0.1239	1.0000
SiK	91.70	82.54	633.44	2.84	0.9014	0.9840	0.9989	1.0001

EDS Spot 14

kV: 10	Mag: 1467	Takeoff: 37.8	Live Time(s): 29.8	Amp Time(μs): 1.92	Resolution:(eV) 127.4

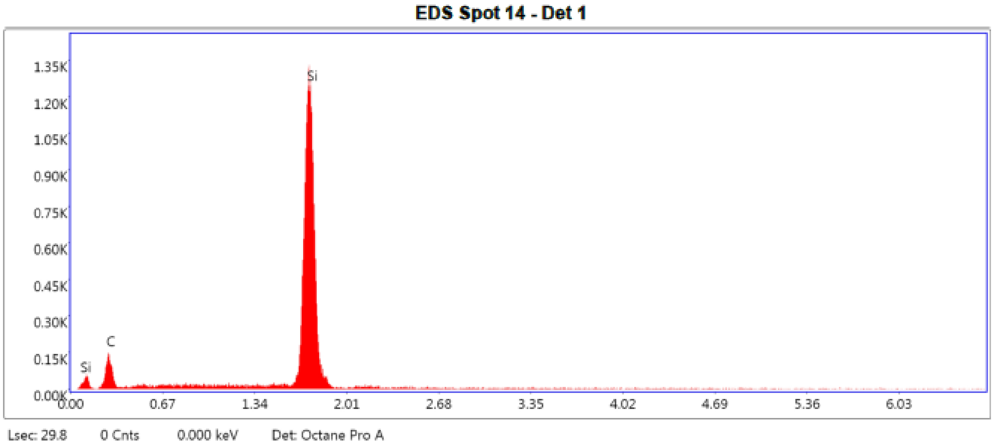

eZAF Smart Quant Results

Element	Weight %	Atomic %	Net Int.	Error %	Kratio	Z	A	F
C K	28.67	48.45	45.29	14.24	0.0514	1.1262	0.1592	1.0000
SiK	71.33	51.55	729.14	2.86	0.6729	0.9466	0.9961	1.0002

EDS Spot 15

kV: 10	Mag: 1467	Takeoff: 37.8	Live Time(s): 29.8	Amp Time(µs): 1.92	Resolution:(eV) 127.4

EDS Spot 15 - Det 1

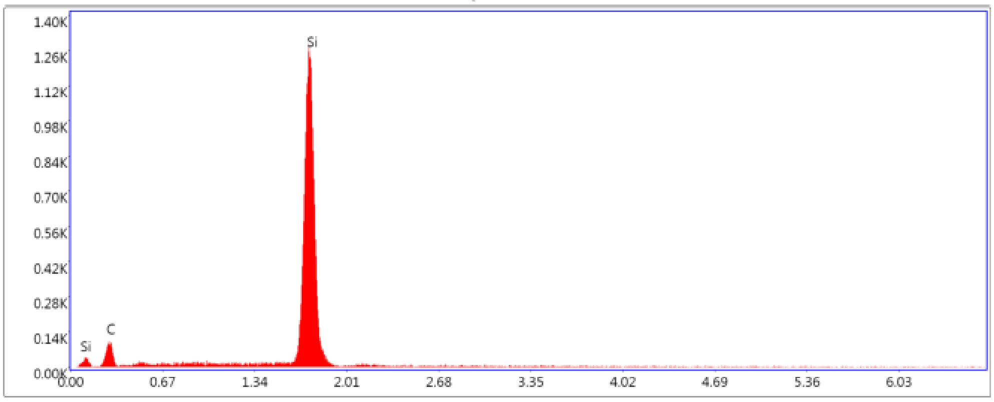

Lsec: 29.8 0 Cnts 0.000 keV Det: Octane Pro A

eZAF Smart Quant Results

Element	Weight %	Atomic %	Net Int.	Error %	Kratio	Z	A	F
C K	23.88	42.32	32.22	15.36	0.0405	1.1358	0.1493	1.0000
SiK	76.12	57.68	709.17	2.86	0.7247	0.9551	0.9968	1.0002

100% H_2 at 1650°C

Area 1

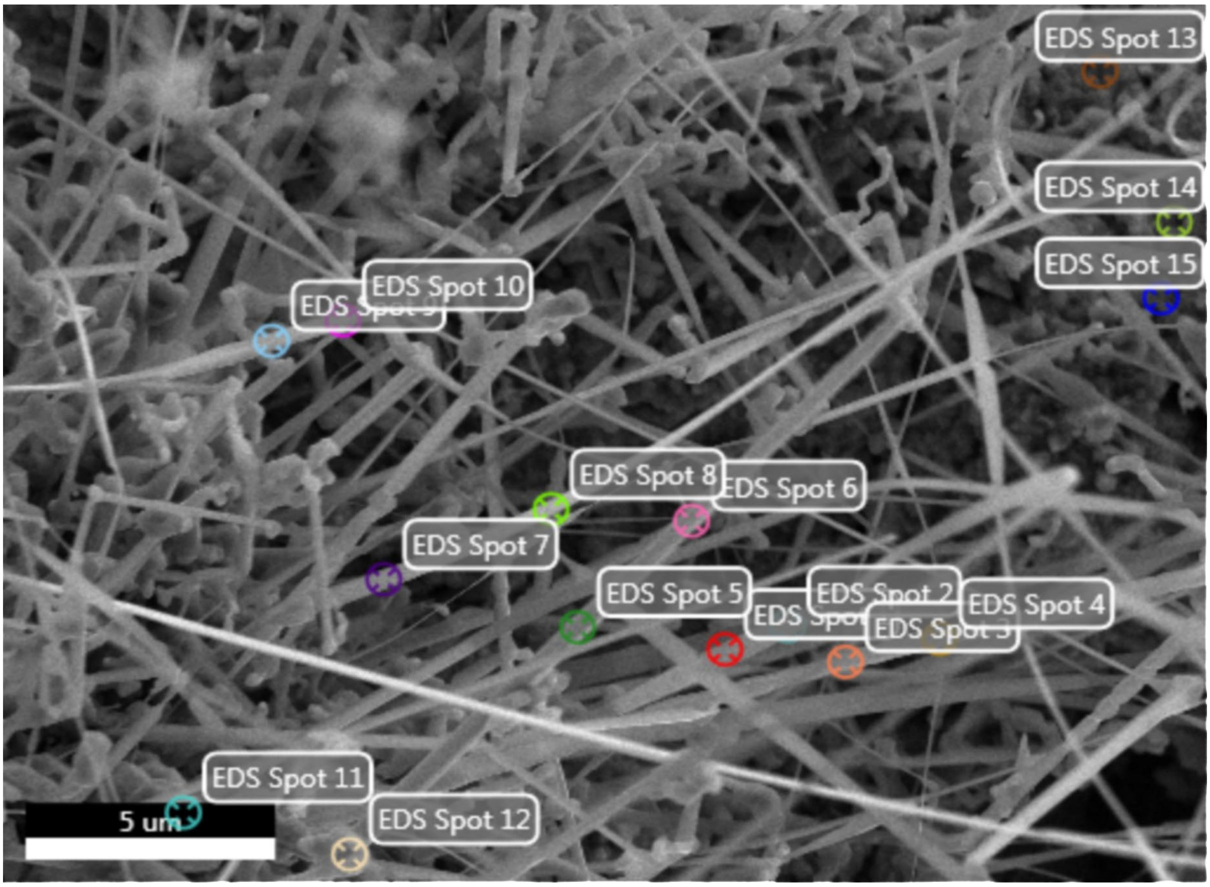

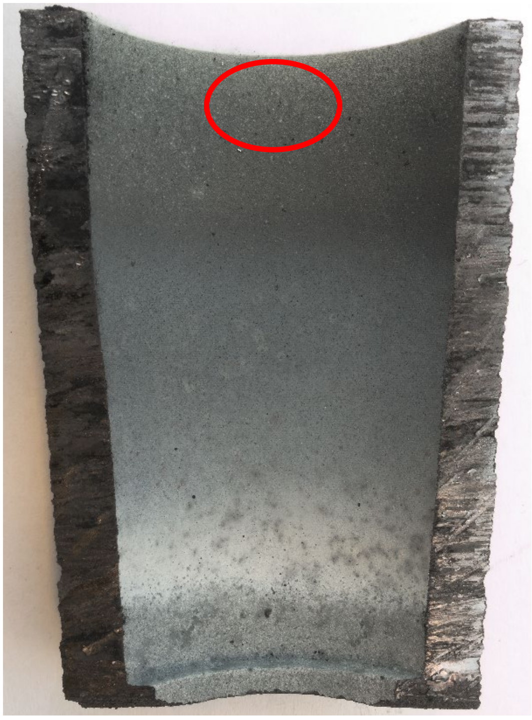

EDS Spot 1

kV: 10	Mag: 4558	Takeoff: 41	Live Time(s): 29.9	Amp Time(μs): 1.92	Resolution:(eV) 127.4

EDS Spot 1 - Det 1

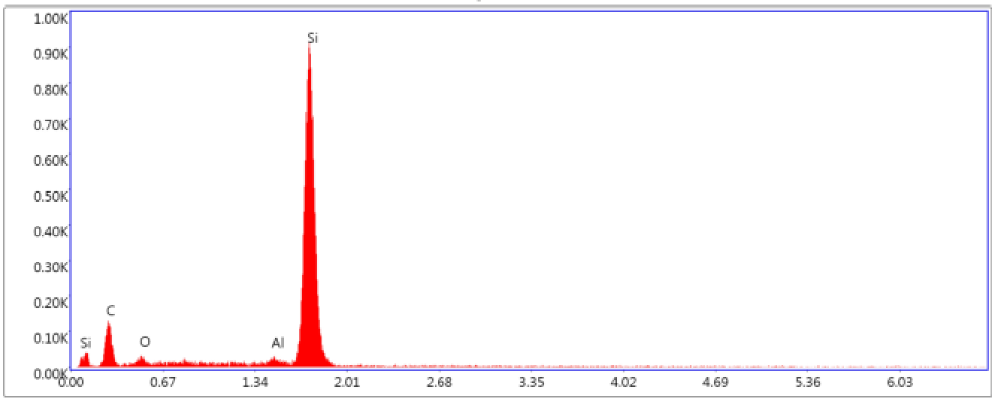

Lsec: 29.9 0 Cnts 0.000 keV Det: Octane Pro A

eZAF Smart Quant Results

Element	Weight %	Atomic %	Net Int.	Error %	Kratio	Z	A	F
C K	31.70	51.72	42.52	13.74	0.0634	1.1190	0.1786	1.0000
O K	1.13	1.39	5.13	37.98	0.0049	1.0584	0.4068	1.0000
AlK	1.37	0.99	11.07	17.97	0.0125	0.9229	0.9747	1.0152
SiK	65.79	45.90	495.26	3.11	0.6127	0.9402	0.9899	1.0003

EDS Spot 2

kV: 10	Mag: 4558	Takeoff: 41	Live Time(s): 29.9	Amp Time(μs): 1.92	Resolution:(eV) 127.4

EDS Spot 2 - Det 1

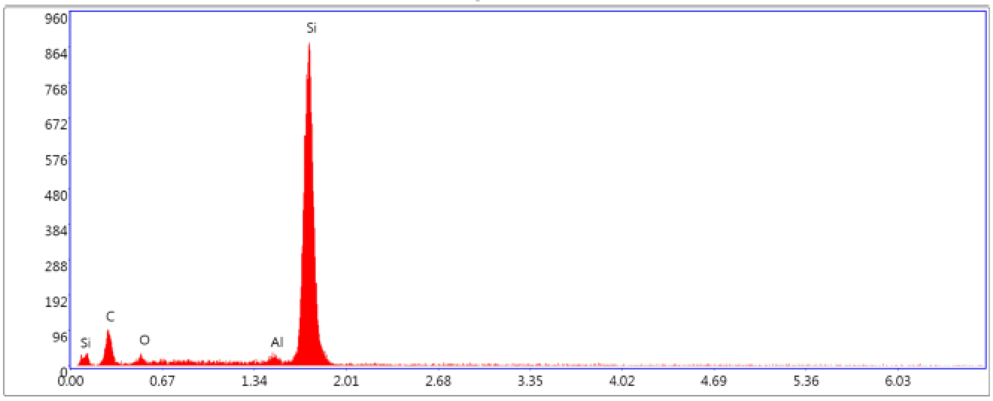

Lsec: 29.9 0 Cnts 0.000 keV Det: Octane Pro A

eZAF Smart Quant Results

Element	Weight %	Atomic %	Net Int.	Error %	Kratio	Z	A	F
C K	27.24	46.36	31.62	15.15	0.0516	1.1280	0.1679	1.0000
O K	1.13	1.44	4.82	38.35	0.0050	1.0670	0.4156	1.0000
AlK	1.78	1.35	13.30	14.62	0.0164	0.9307	0.9758	1.0152
SiK	69.85	50.84	483.91	3.13	0.6551	0.9481	0.9889	1.0002

EDS Spot 3

kV: 10	Mag: 4558	Takeoff: 41	Live Time(s): 29.8	Amp Time(µs): 1.92	Resolution:(eV) 127.4

EDS Spot 3 - Det 1

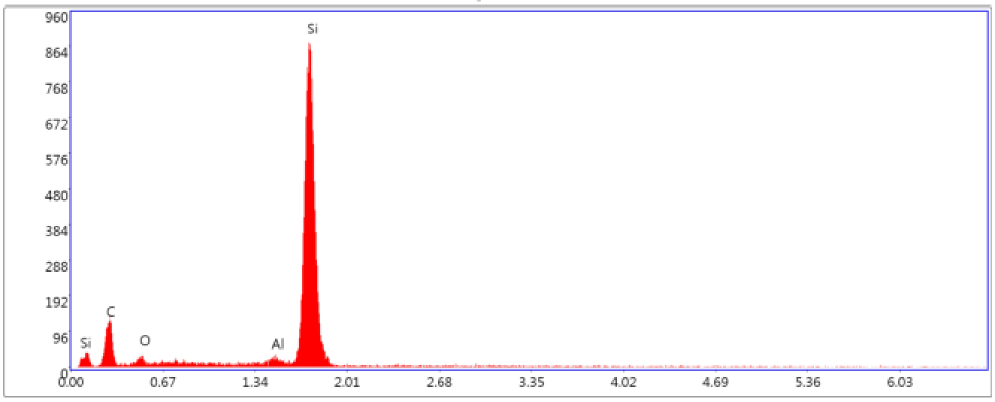

Lsec: 29.8 0 Cnts 0.000 keV Det: Octane Pro A

eZAF Smart Quant Results

Element	Weight %	Atomic %	Net Int.	Error %	Kratio	Z	A	F
C K	33.47	53.55	48.04	13.51	0.0690	1.1149	0.1850	1.0000
O K	1.68	2.02	7.83	25.21	0.0072	1.0544	0.4048	1.0000
AlK	1.78	1.26	14.81	13.55	0.0161	0.9193	0.9736	1.0148
SiK	63.08	43.16	489.53	3.15	0.5835	0.9365	0.9877	1.0003

EDS Spot 4

kV: 10	Mag: 4558	Takeoff: 41	Live Time(s): 29.8	Amp Time(µs): 1.92	Resolution:(eV) 127.4

EDS Spot 4 - Det 1

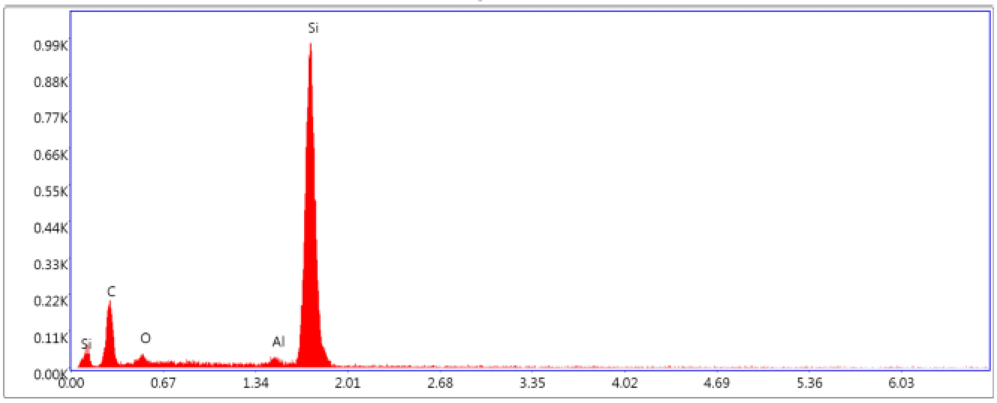

Lsec: 29.8 0 Cnts 0.000 keV Det: Octane Pro A

eZAF Smart Quant Results

Element	Weight %	Atomic %	Net Int.	Error %	Kratio	Z	A	F
C K	39.00	59.21	74.52	12.52	0.0874	1.1032	0.2032	1.0000
O K	2.31	2.64	12.75	19.98	0.0095	1.0431	0.3957	1.0000
AlK	1.34	0.90	13.47	15.50	0.0120	0.9092	0.9714	1.0147
SiK	57.36	37.25	539.30	3.11	0.5249	0.9261	0.9881	1.0004

EDS Spot 5

kV: 10	Mag: 4558	Takeoff: 41	Live Time(s): 29.9	Amp Time(µs): 1.92	Resolution:(eV) 127.4

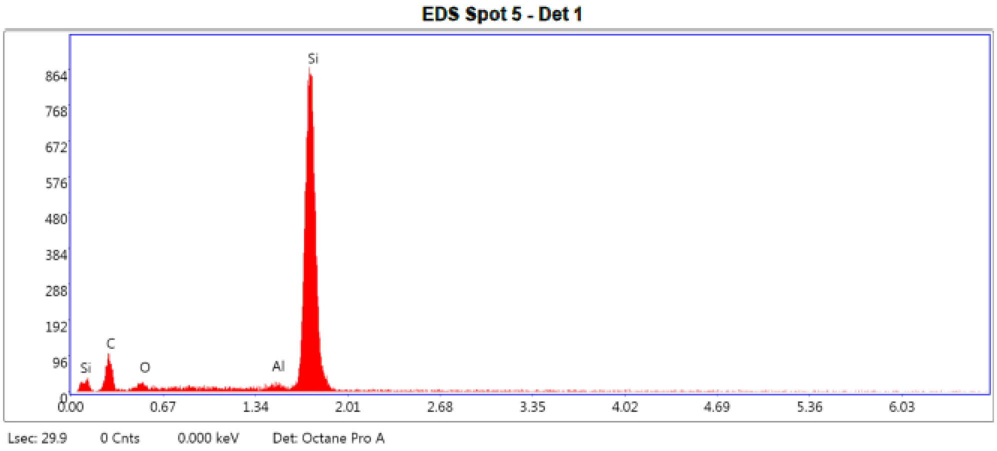

eZAF Smart Quant Results

Element	Weight %	Atomic %	Net Int.	Error %	Kratio	Z	A	F
C K	27.11	46.12	32.56	14.66	0.0514	1.1277	0.1681	1.0000
O K	1.46	1.87	6.46	25.76	0.0065	1.0668	0.4164	1.0000
AlK	1.25	0.95	9.64	17.09	0.0115	0.9304	0.9752	1.0154
SiK	70.18	51.06	503.34	3.08	0.6591	0.9479	0.9906	1.0002

EDS Spot 6

kV: 10	Mag: 4558	Takeoff: 41	Live Time(s): 29.8	Amp Time(µs): 1.92	Resolution:(eV) 127.4

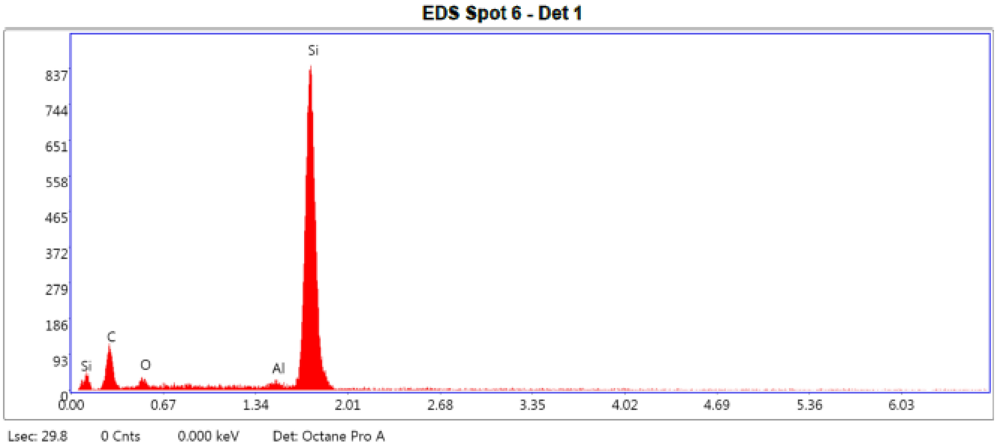

eZAF Smart Quant Results

Element	Weight %	Atomic %	Net Int.	Error %	Kratio	Z	A	F
C K	31.32	51.16	39.94	13.83	0.0626	1.1193	0.1785	1.0000
O K	1.54	1.89	6.68	30.64	0.0067	1.0586	0.4085	1.0000
AlK	1.29	0.94	9.94	18.46	0.0118	0.9231	0.9742	1.0152
SiK	65.85	46.00	471.47	3.13	0.6132	0.9404	0.9898	1.0003

EDS Spot 7

kV: 10	Mag: 4558	Takeoff: 41	Live Time(s): 29.9	Amp Time(µs): 1.92	Resolution:(eV) 127.4

EDS Spot 7 - Det 1

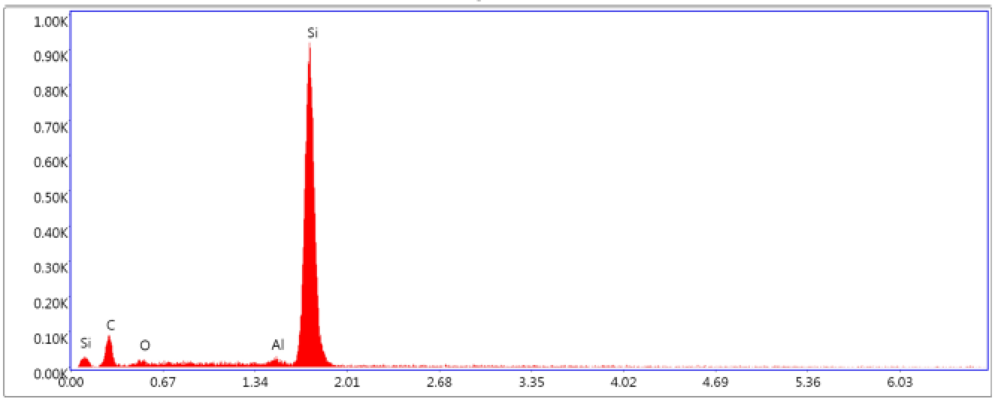

Lsec: 29.9 0 Cnts 0.000 keV Det: Octane Pro A

eZAF Smart Quant Results

Element	Weight %	Atomic %	Net Int.	Error %	Kratio	Z	A	F
C K	25.85	44.71	28.86	15.39	0.0479	1.1312	0.1638	1.0000
O K	0.74	0.96	3.13	70.26	0.0033	1.0702	0.4172	1.0000
AlK	1.41	1.08	10.35	17.95	0.0130	0.9335	0.9766	1.0155
SiK	72.00	53.25	492.60	3.10	0.6786	0.9510	0.9908	1.0002

EDS Spot 8

kV: 10	Mag: 4558	Takeoff: 41	Live Time(s): 29.9	Amp Time(µs): 1.92	Resolution:(eV) 127.4

EDS Spot 8 - Det 1

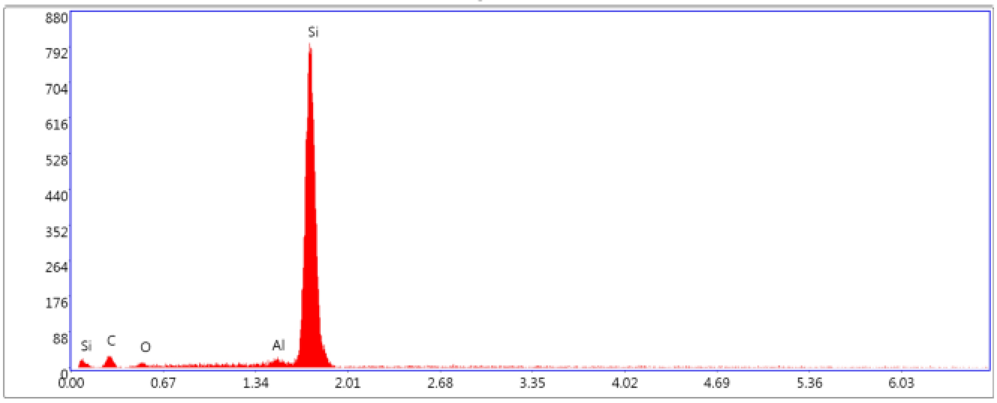

Lsec: 29.9 0 Cnts 0.000 keV Det: Octane Pro A

eZAF Smart Quant Results

Element	Weight %	Atomic %	Net Int.	Error %	Kratio	Z	A	F
C K	11.96	24.02	8.54	22.66	0.0191	1.1606	0.1375	1.0000
O K	0.39	0.59	1.35	77.44	0.0019	1.0986	0.4453	1.0000
AlK	2.19	1.96	12.35	14.35	0.0209	0.9589	0.9804	1.0156
SiK	85.46	73.43	445.93	3.12	0.8266	0.9770	0.9899	1.0001

EDS Spot 9

kV: 10	Mag: 4558	Takeoff: 41	Live Time(s): 29.8	Amp Time(µs): 1.92	Resolution:(eV) 127.4

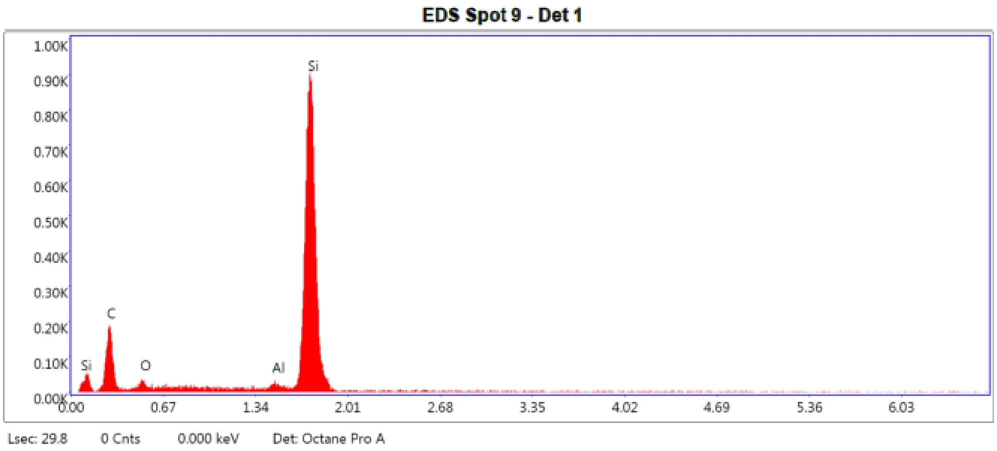

eZAF Smart Quant Results

Element	Weight %	Atomic %	Net Int.	Error %	Kratio	Z	A	F
C K	38.82	59.32	67.63	12.89	0.0856	1.1047	0.1997	1.0000
O K	1.35	1.55	6.86	30.72	0.0055	1.0445	0.3937	1.0000
AlK	1.08	0.74	10.13	19.80	0.0097	0.9105	0.9727	1.0150
SiK	58.75	38.40	513.56	3.10	0.5394	0.9275	0.9899	1.0004

EDS Spot 10

kV: 10	Mag: 4558	Takeoff: 41	Live Time(s): 29.9	Amp Time(µs): 1.92	Resolution:(eV) 127.4

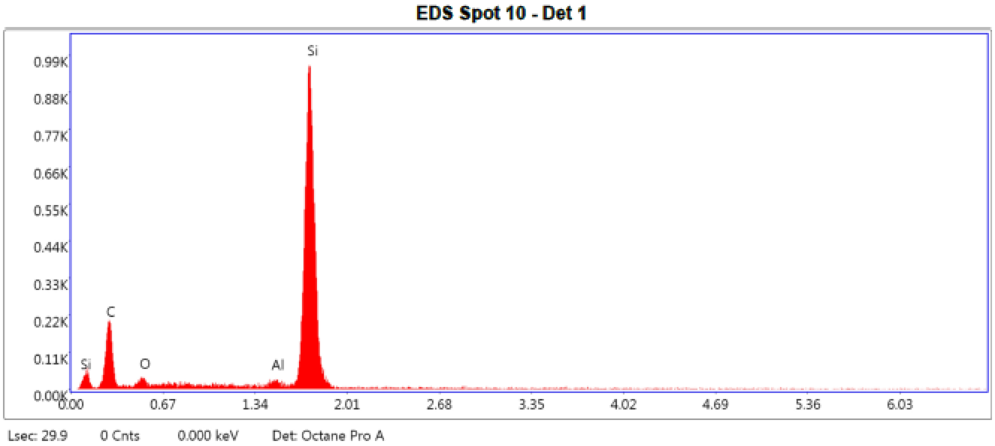

eZAF Smart Quant Results

Element	Weight %	Atomic %	Net Int.	Error %	Kratio	Z	A	F
C K	40.43	60.82	76.57	12.45	0.0918	1.1013	0.2061	1.0000
O K	1.72	1.94	9.17	24.77	0.0070	1.0412	0.3918	1.0000
AlK	1.30	0.87	12.78	15.41	0.0116	0.9075	0.9719	1.0148
SiK	56.55	36.38	519.84	3.11	0.5171	0.9244	0.9886	1.0004

EDS Spot 11

kV: 10	Mag: 4558	Takeoff: 41	Live Time(s): 29.8	Amp Time(μs): 1.92	Resolution:(eV) 127.4

EDS Spot 11 - Det 1

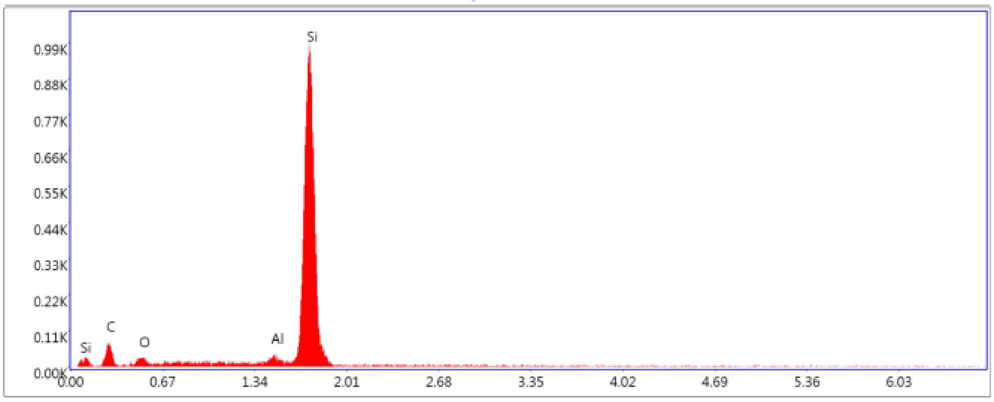

Lsec: 29.8 0 Cnts 0.000 keV Det: Octane Pro A

eZAF Smart Quant Results

Element	Weight %	Atomic %	Net Int.	Error %	Kratio	Z	A	F
C K	19.97	36.50	21.82	16.09	0.0350	1.1423	0.1532	1.0000
O K	1.48	2.03	6.74	30.35	0.0069	1.0809	0.4312	1.0000
AlK	1.99	1.62	15.37	14.05	0.0187	0.9431	0.9770	1.0153
SiK	76.56	59.85	547.27	3.08	0.7274	0.9608	0.9887	1.0002

EDS Spot 12

kV: 10	Mag: 4558	Takeoff: 41	Live Time(s): 29.9	Amp Time(μs): 1.92	Resolution:(eV) 127.4

EDS Spot 12 - Det 1

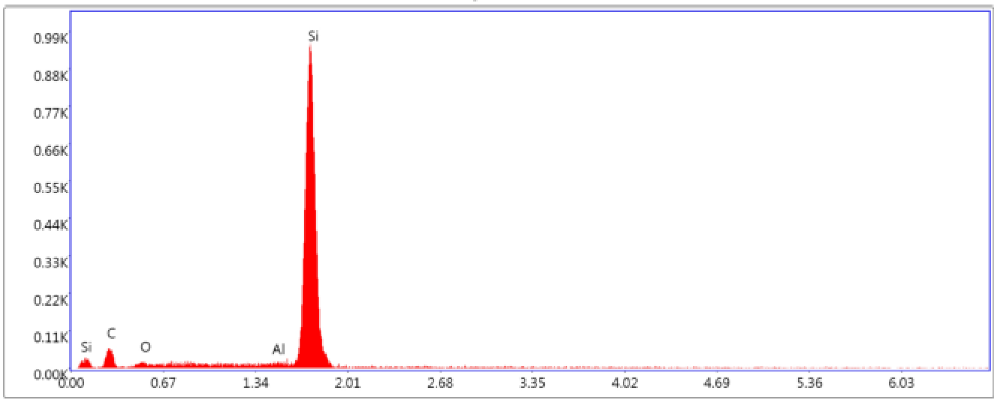

Lsec: 29.9 0 Cnts 0.000 keV Det: Octane Pro A

eZAF Smart Quant Results

Element	Weight %	Atomic %	Net Int.	Error %	Kratio	Z	A	F
C K	19.01	35.32	18.68	17.90	0.0325	1.1455	0.1494	1.0000
O K	0.45	0.63	1.90	74.47	0.0021	1.0840	0.4301	1.0000
AlK	1.12	0.93	8.01	22.99	0.0106	0.9459	0.9785	1.0159
SiK	79.42	63.12	526.14	3.02	0.7600	0.9637	0.9930	1.0001

EDS Spot 13

kV: 10	Mag: 4558	Takeoff: 41	Live Time(s): 29.9	Amp Time(μs): 1.92	Resolution:(eV) 127.4

EDS Spot 13 - Det 1

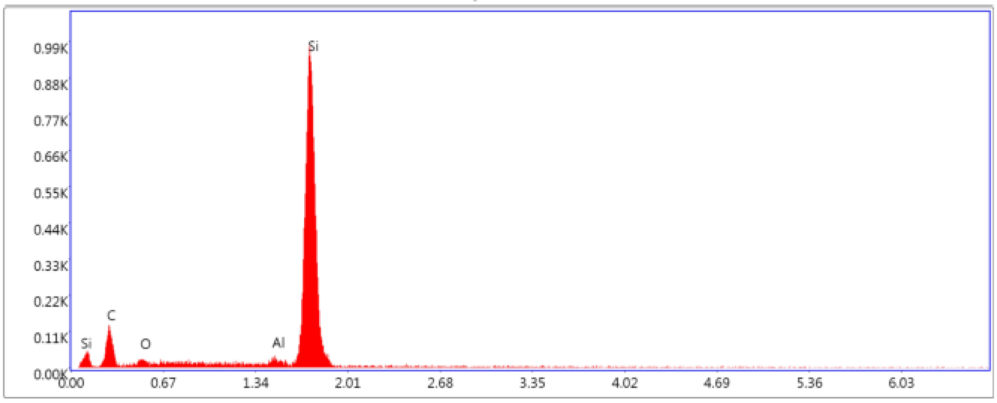

Lsec: 29.9 0 Cnts 0.000 keV Det: Octane Pro A

eZAF Smart Quant Results

Element	Weight %	Atomic %	Net Int.	Error %	Kratio	Z	A	F
C K	29.78	49.55	40.53	14.24	0.0579	1.1232	0.1731	1.0000
O K	0.82	1.03	3.94	68.71	0.0036	1.0624	0.4097	1.0000
AlK	1.31	0.97	11.09	18.96	0.0120	0.9266	0.9755	1.0153
SiK	68.09	48.45	537.23	3.07	0.6369	0.9439	0.9906	1.0003

EDS Spot 14

kV: 10	Mag: 4558	Takeoff: 41	Live Time(s): 29.9	Amp Time(μs): 1.92	Resolution:(eV) 127.4

EDS Spot 14 - Det 1

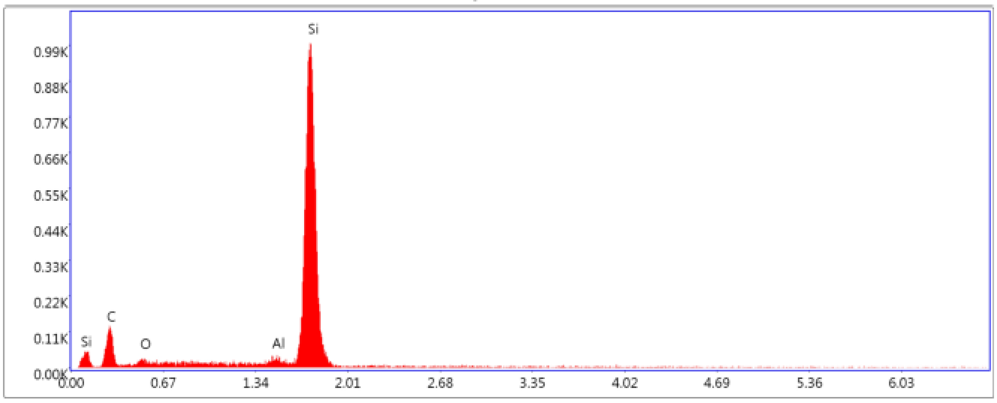

Lsec: 29.9 0 Cnts 0.000 keV Det: Octane Pro A

eZAF Smart Quant Results

Element	Weight %	Atomic %	Net Int.	Error %	Kratio	Z	A	F
C K	29.51	49.14	42.42	13.83	0.0574	1.1235	0.1732	1.0000
O K	1.12	1.40	5.67	37.33	0.0049	1.0627	0.4111	1.0000
AlK	1.65	1.22	14.73	15.27	0.0151	0.9267	0.9752	1.0151
SiK	67.73	48.24	563.36	3.07	0.6327	0.9441	0.9891	1.0003

EDS Spot 15

kV: 10	Mag: 4558	Takeoff: 41	Live Time(s): 29.9	Amp Time(µs): 1.92	Resolution:(eV) 127.4

EDS Spot 15 - Det 1

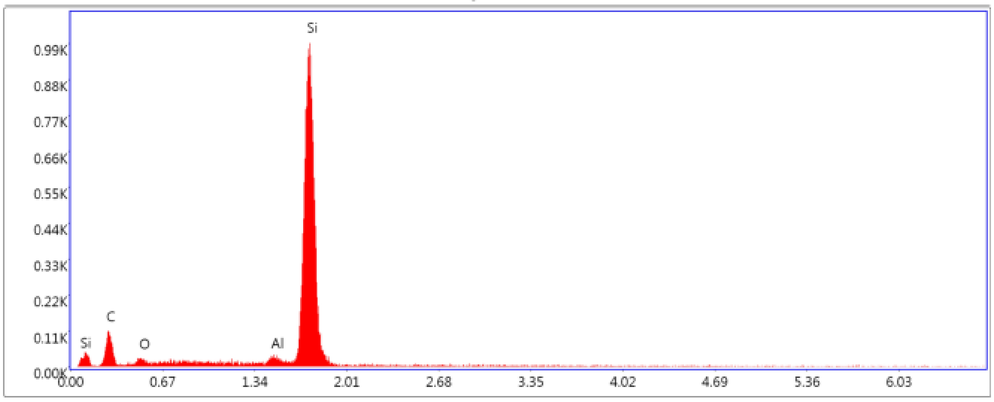

Lsec: 29.9 0 Cnts 0.000 keV Det: Octane Pro A

eZAF Smart Quant Results

Element	Weight %	Atomic %	Net Int.	Error %	Kratio	Z	A	F
C K	26.91	46.02	34.91	14.99	0.0506	1.1290	0.1666	1.0000
O K	0.87	1.11	4.15	54.92	0.0038	1.0680	0.4156	1.0000
AlK	1.72	1.31	14.49	15.41	0.0159	0.9315	0.9762	1.0152
SiK	70.50	51.56	550.46	3.07	0.6622	0.9490	0.9894	1.0002

Area 2

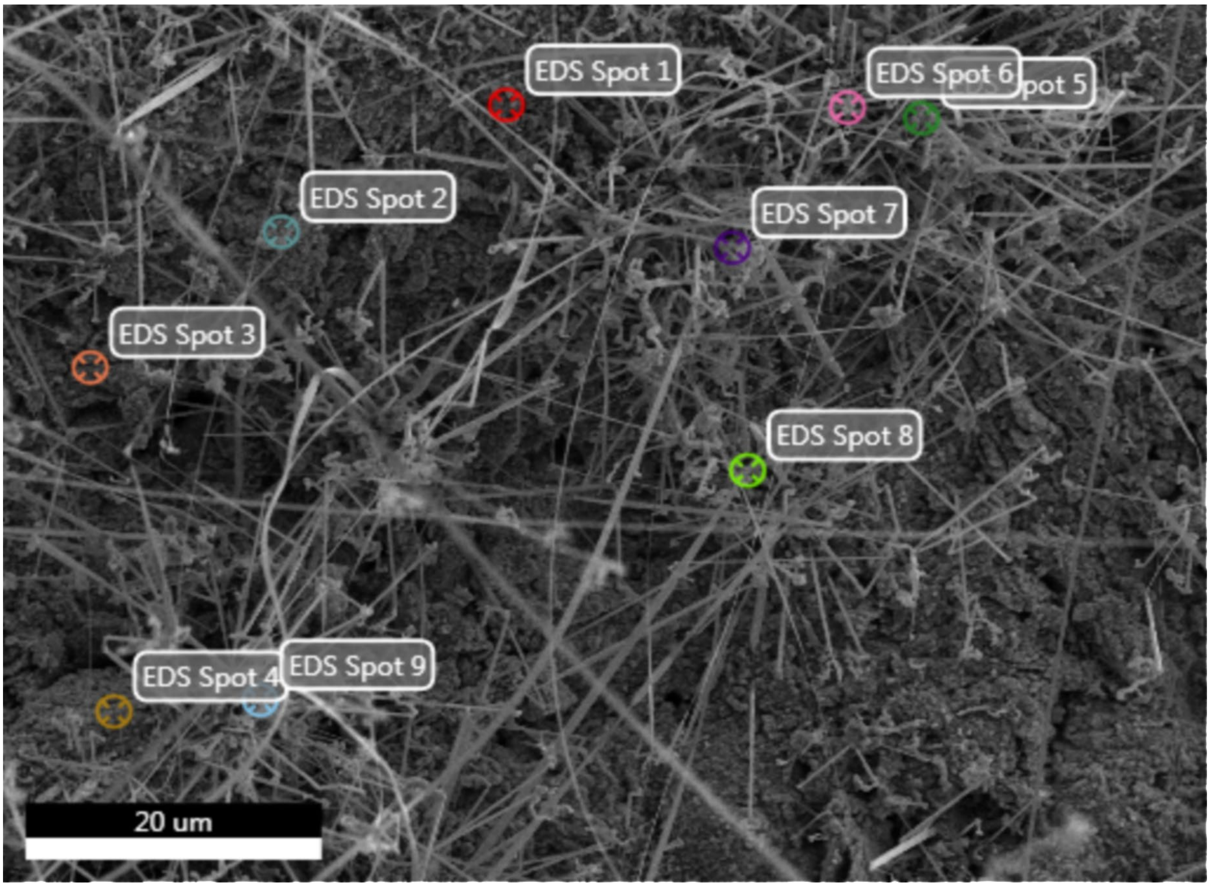
EDS Spot 1
EDS Spot 6
pot 5
EDS Spot 2
EDS Spot 7
EDS Spot 3
EDS Spot 8
EDS Spot 4
EDS Spot 9
20 um

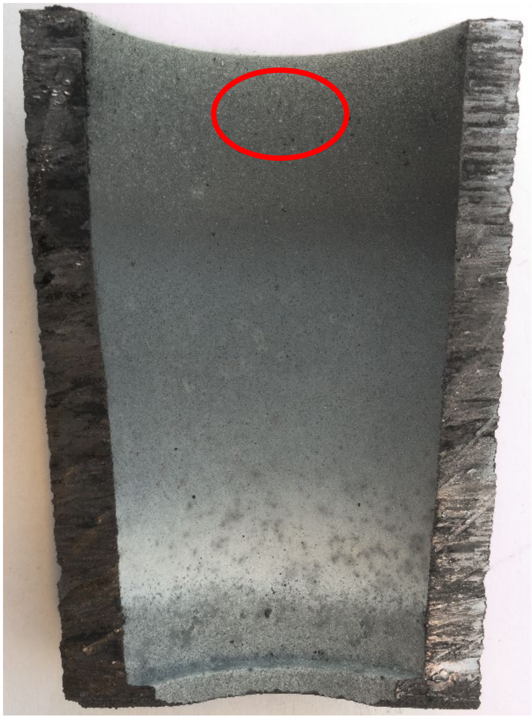

EDS Spot 1

kV: 10	Mag: 1350	Takeoff: 41	Live Time(s): 29.9	Amp Time(µs): 1.92	Resolution:(eV) 127.4

EDS Spot 1 - Det 1

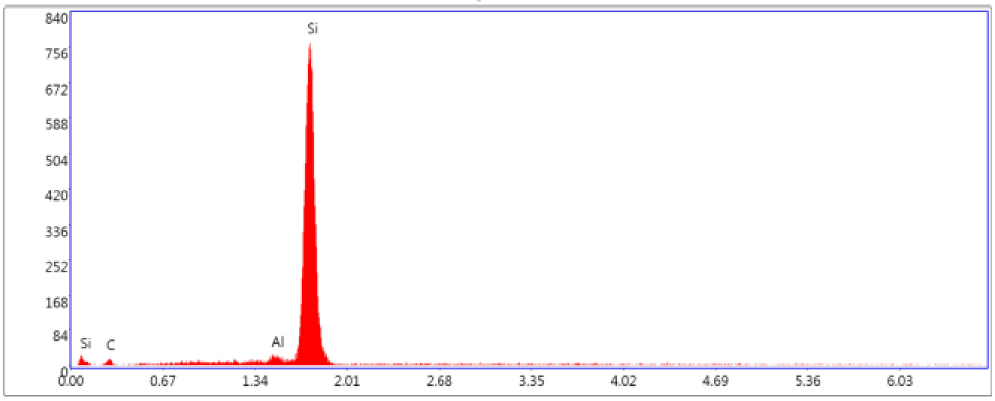

Lsec: 29.9 0 Cnts 0.000 keV Det: Octane Pro A

eZAF Smart Quant Results

Element	Weight %	Atomic %	Net Int.	Error %	Kratio	Z	A	F
C K	3.76	8.37	2.16	65.45	0.0056	1.1789	0.1253	1.0000
AlK	2.52	2.49	12.57	14.19	0.0245	0.9747	0.9829	1.0157
SiK	93.72	89.14	432.08	3.16	0.9216	0.9932	0.9901	1.0000

EDS Spot 2

kV: 10	Mag: 1350	Takeoff: 41	Live Time(s): 29.8	Amp Time(µs): 1.92	Resolution:(eV) 127.4

EDS Spot 2 - Det 1

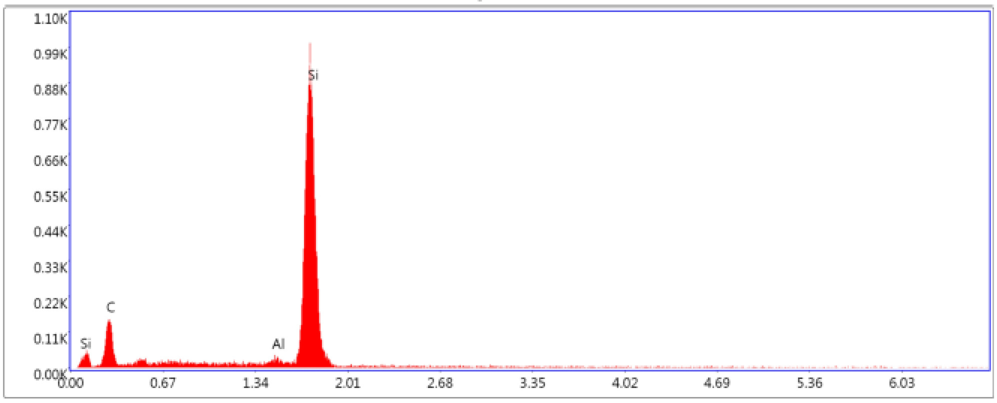

Lsec: 29.8 0 Cnts 0.000 keV Det: Octane Pro A

eZAF Smart Quant Results

Element	Weight %	Atomic %	Net Int.	Error %	Kratio	Z	A	F
C K	35.09	55.81	53.28	13.36	0.0723	1.1137	0.1852	1.0000
AlK	1.36	0.97	12.05	18.49	0.0124	0.9183	0.9755	1.0152
SiK	63.55	43.23	522.72	3.09	0.5889	0.9355	0.9905	1.0003

EDS Spot 3

kV: 10	Mag: 1350	Takeoff: 41	Live Time(s): 29.9	Amp Time(µs): 1.92	Resolution:(eV) 127.4

EDS Spot 3 - Det 1

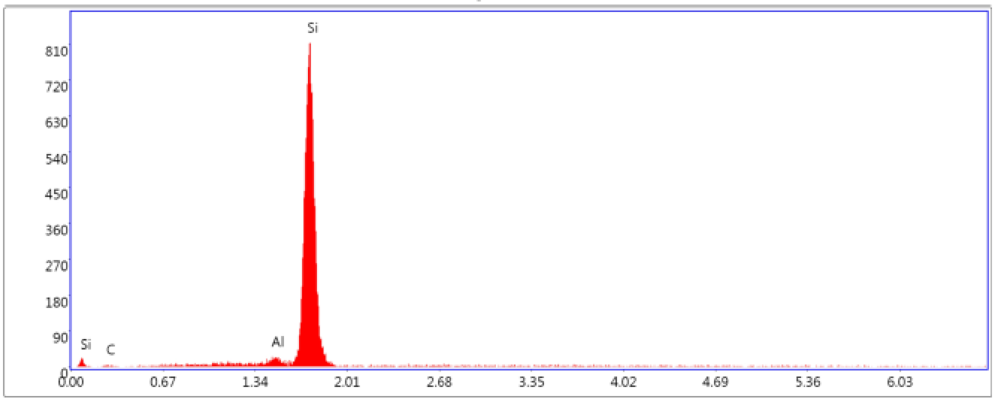

Lsec: 29.9 0 Cnts 0.000 keV Det: Octane Pro A

eZAF Smart Quant Results

Element	Weight %	Atomic %	Net Int.	Error %	Kratio	Z	A	F
C K	0.40	0.93	0.22	99.99	0.0006	1.1864	0.1211	1.0000
AlK	3.05	3.15	15.25	11.43	0.0299	0.9812	0.9838	1.0156
SiK	96.55	95.92	444.55	3.11	0.9542	0.9998	0.9885	1.0000

EDS Spot 4

kV: 10	Mag: 1350	Takeoff: 41	Live Time(s): 29.8	Amp Time(µs): 1.92	Resolution:(eV) 127.4

EDS Spot 4 - Det 1

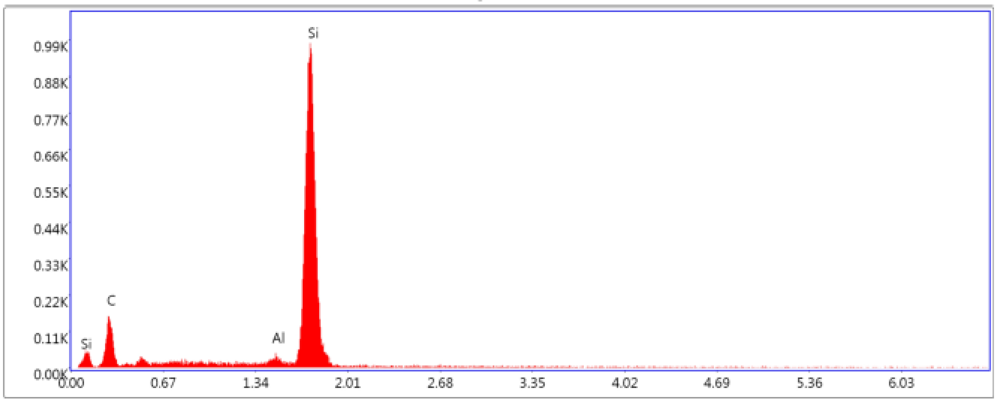

Lsec: 29.8 0 Cnts 0.000 keV Det: Octane Pro A

eZAF Smart Quant Results

Element	Weight %	Atomic %	Net Int.	Error %	Kratio	Z	A	F
C K	34.53	55.20	53.89	13.34	0.0707	1.1149	0.1838	1.0000
AlK	1.66	1.18	15.18	15.00	0.0151	0.9193	0.9757	1.0150
SiK	63.81	43.62	542.76	3.08	0.5914	0.9365	0.9895	1.0003

EDS Spot 5

kV: 10	Mag: 1350	Takeoff: 41	Live Time(s): 29.9	Amp Time(μs): 1.92	Resolution:(eV) 127.4

EDS Spot 5 - Det 1

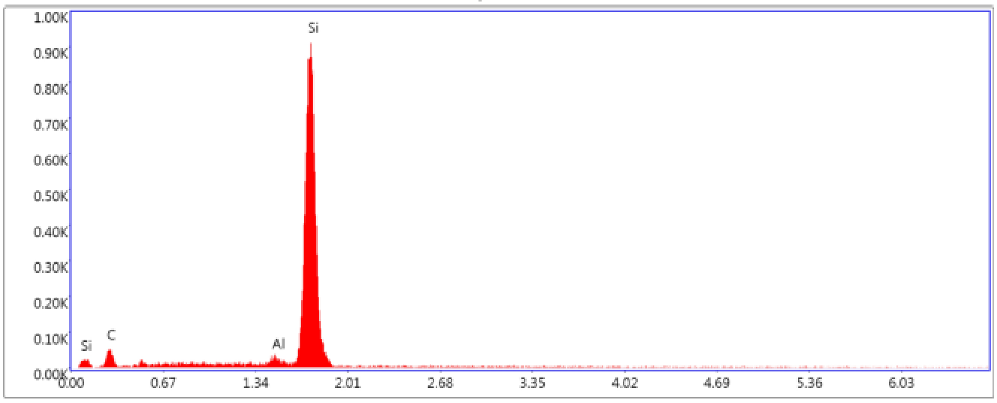

Lsec: 29.9 0 Cnts 0.000 keV Det: Octane Pro A

eZAF Smart Quant Results

Element	Weight %	Atomic %	Net Int.	Error %	Kratio	Z	A	F
C K	16.67	31.85	15.15	18.55	0.0278	1.1512	0.1447	1.0000
AlK	2.03	1.73	13.85	14.17	0.0193	0.9508	0.9798	1.0155
SiK	81.29	66.42	512.41	3.09	0.7800	0.9687	0.9902	1.0001

EDS Spot 6

kV: 10	Mag: 1350	Takeoff: 41	Live Time(s): 29.9	Amp Time(μs): 1.92	Resolution:(eV) 127.4

EDS Spot 6 - Det 1

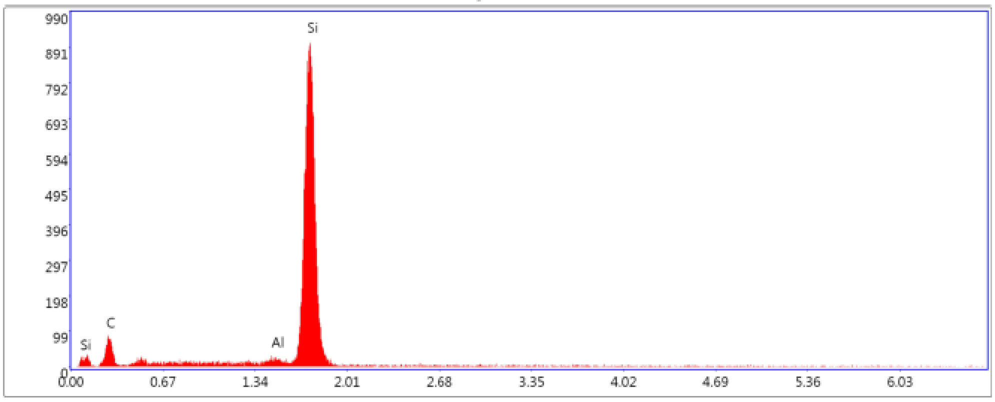

Lsec: 29.9 0 Cnts 0.000 keV Det: Octane Pro A

eZAF Smart Quant Results

Element	Weight %	Atomic %	Net Int.	Error %	Kratio	Z	A	F
C K	24.31	42.87	26.88	15.55	0.0439	1.1353	0.1592	1.0000
AlK	1.52	1.20	11.44	16.63	0.0142	0.9370	0.9780	1.0155
SiK	74.17	55.94	517.50	3.08	0.7018	0.9546	0.9912	1.0002

EDS Spot 7

kV: 10	Mag: 1350	Takeoff: 41	Live Time(s): 29.8	Amp Time(µs): 1.92	Resolution:(eV) 127.4

EDS Spot 7 - Det 1

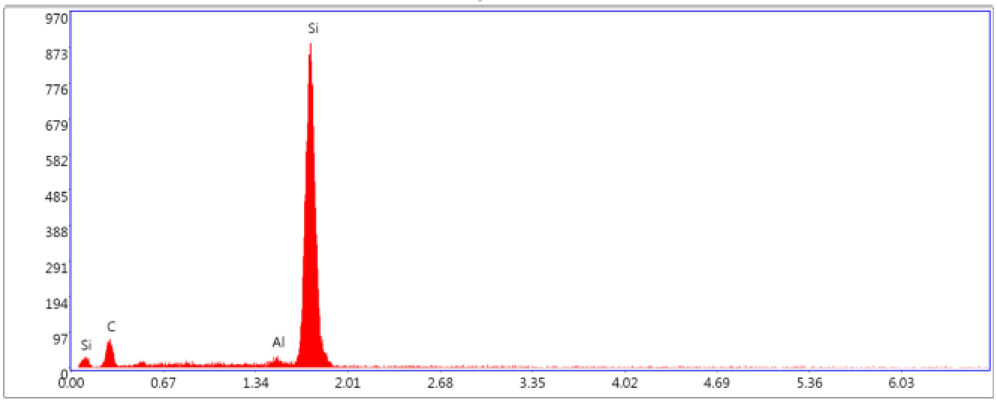

Lsec: 29.8 0 Cnts 0.000 keV Det: Octane Pro A

eZAF Smart Quant Results

Element	Weight %	Atomic %	Net Int.	Error %	Kratio	Z	A	F
C K	25.35	44.24	27.61	15.49	0.0464	1.1332	0.1616	1.0000
AlK	1.82	1.41	13.25	14.16	0.0169	0.9352	0.9778	1.0153
SiK	72.83	54.35	492.48	3.09	0.6869	0.9528	0.9900	1.0002

EDS Spot 8

kV: 10	Mag: 1350	Takeoff: 41	Live Time(s): 29.9	Amp Time(µs): 1.92	Resolution:(eV) 127.4

EDS Spot 8 - Det 1

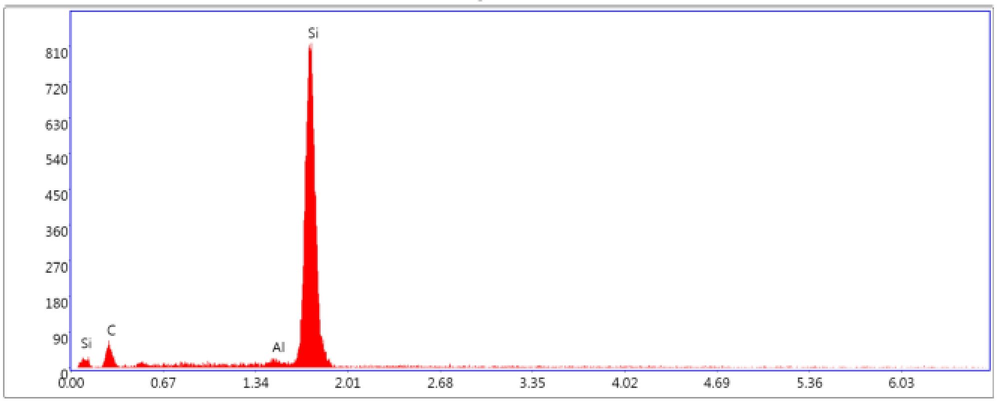

Lsec: 29.9 0 Cnts 0.000 keV Det: Octane Pro A

eZAF Smart Quant Results

Element	Weight %	Atomic %	Net Int.	Error %	Kratio	Z	A	F
C K	20.73	37.92	18.82	17.03	0.0360	1.1427	0.1521	1.0000
AlK	1.64	1.34	10.62	17.10	0.0154	0.9434	0.9788	1.0156
SiK	77.63	60.74	465.80	3.12	0.7396	0.9611	0.9912	1.0002

EDS Spot 9

kV: 10	Mag: 1350	Takeoff: 41	Live Time(s): 29.9	Amp Time(μs): 1.92	Resolution:(eV) 127.4

EDS Spot 9 - Det 1

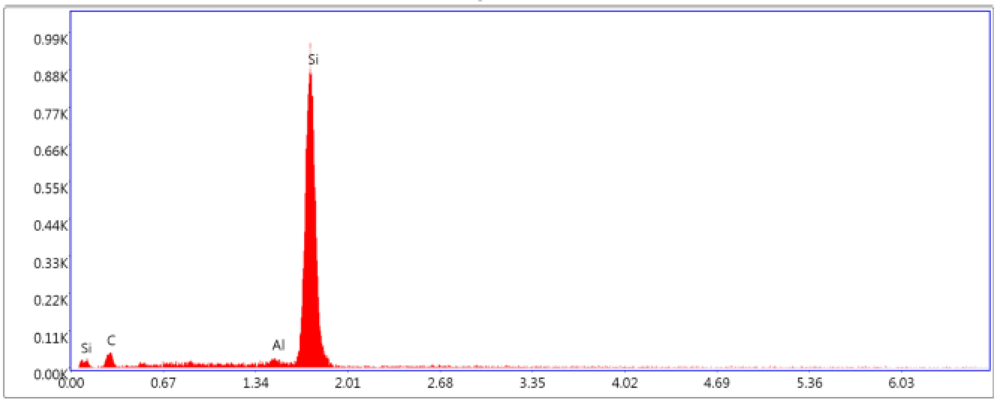

Lsec: 29.9 0 Cnts 0.000 keV Det: Octane Pro A

eZAF Smart Quant Results

Element	Weight %	Atomic %	Net Int.	Error %	Kratio	Z	A	F
C K	14.57	28.49	12.56	19.13	0.0238	1.1556	0.1412	1.0000
AlK	2.07	1.80	13.73	14.52	0.0197	0.9546	0.9803	1.0156
SiK	83.36	69.71	511.38	3.06	0.8031	0.9726	0.9904	1.0001

100% H_2 at 1750°C

Area 1

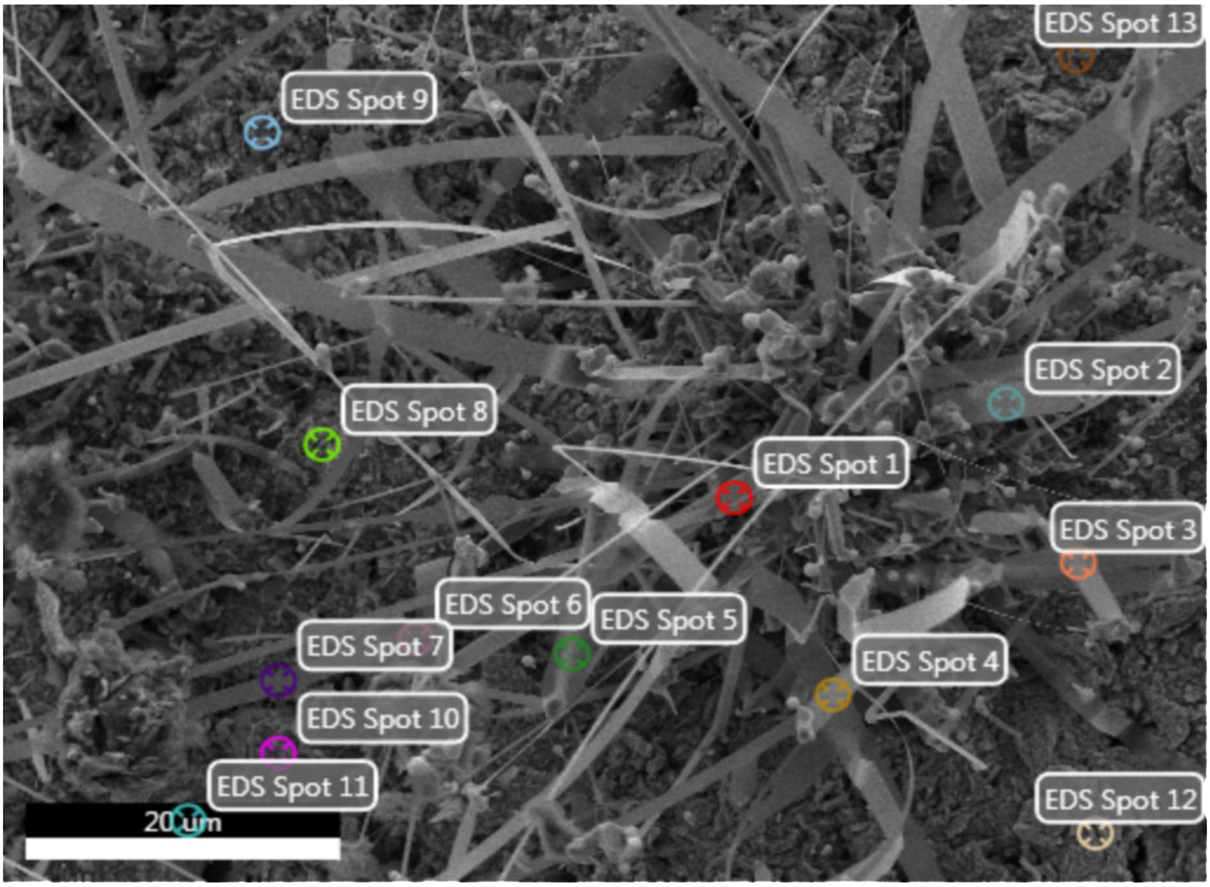

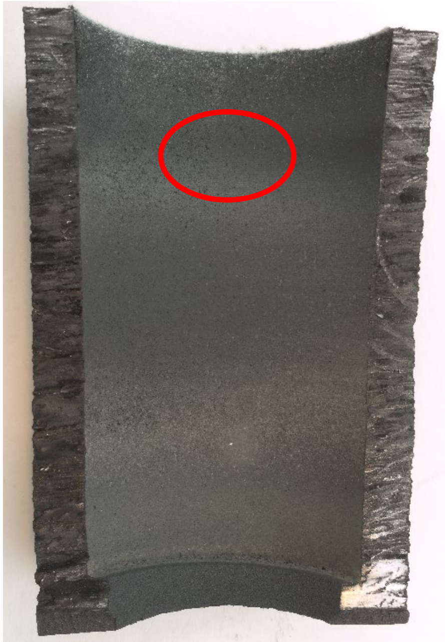

EDS Spot 1

kV: 10	Mag: 1437	Takeoff: 40.6	Live Time(s): 29.9	Amp Time(µs): 1.92	Resolution:(eV) 127.4

EDS Spot 1 - Det 1

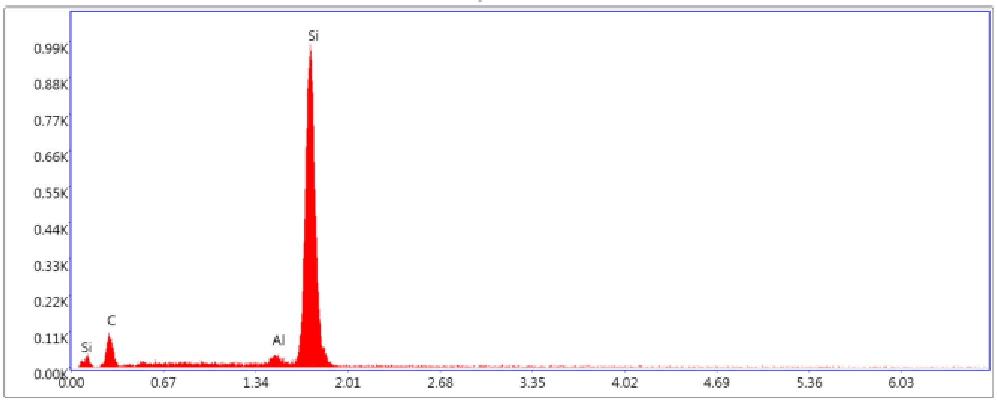

Lsec: 29.9 0 Cnts 0.000 keV Det: Octane Pro A

eZAF Smart Quant Results

Element	Weight %	Atomic %	Net Int.	Error %	Kratio	Z	A	F
C K	27.01	46.36	33.85	15.07	0.0501	1.1300	0.1641	1.0000
AlK	2.36	1.80	19.47	11.91	0.0218	0.9325	0.9773	1.0150
SiK	70.63	51.84	541.18	3.09	0.6629	0.9499	0.9877	1.0002

EDS Spot 2

kV: 10	Mag: 1437	Takeoff: 40.6	Live Time(s): 29.9	Amp Time(µs): 1.92	Resolution:(eV) 127.4

EDS Spot 2 - Det 1

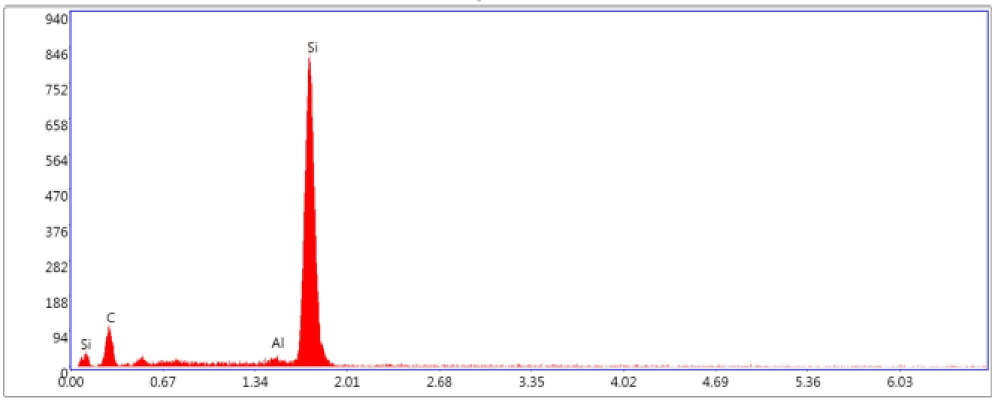

Lsec: 29.9 0 Cnts 0.000 keV Det: Octane Pro A

eZAF Smart Quant Results

Element	Weight %	Atomic %	Net Int.	Error %	Kratio	Z	A	F
C K	31.36	51.63	37.25	13.99	0.0612	1.1212	0.1740	1.0000
AlK	1.70	1.24	12.51	15.86	0.0155	0.9248	0.9762	1.0152
SiK	66.94	47.13	459.03	3.15	0.6245	0.9421	0.9896	1.0003

EDS Spot 3

kV: 10	Mag: 1437	Takeoff: 40.6	Live Time(s): 29.8	Amp Time(µs): 1.92	Resolution:(eV) 127.4

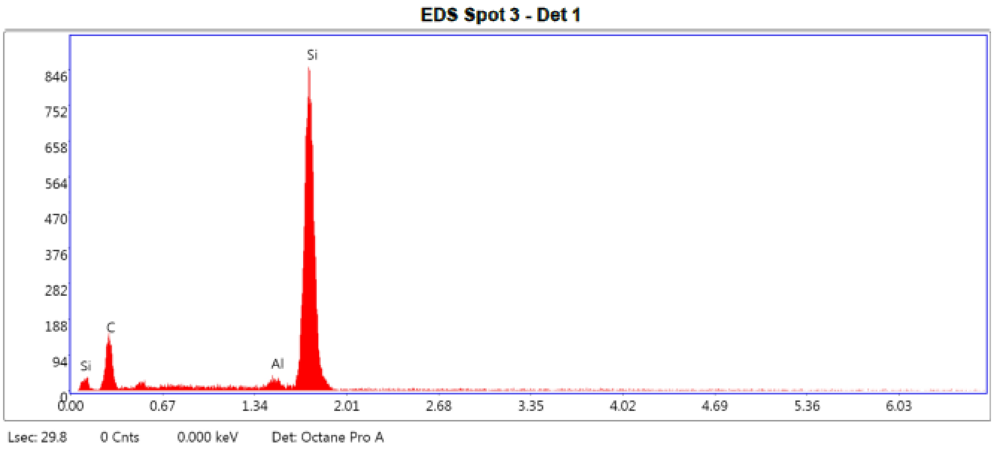

eZAF Smart Quant Results

Element	Weight %	Atomic %	Net Int.	Error %	Kratio	Z	A	F
C K	37.11	57.95	53.55	13.26	0.0782	1.1099	0.1899	1.0000
AlK	2.04	1.42	16.74	11.80	0.0185	0.9150	0.9749	1.0147
SiK	60.85	40.63	463.39	3.17	0.5602	0.9321	0.9876	1.0003

EDS Spot 4

kV: 10	Mag: 1437	Takeoff: 40.6	Live Time(s): 30	Amp Time(µs): 1.92	Resolution:(eV) 127.4

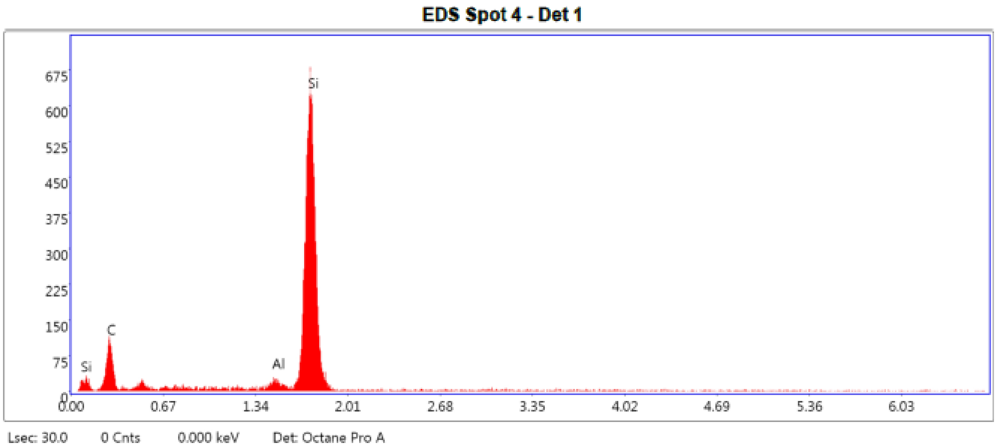

eZAF Smart Quant Results

Element	Weight %	Atomic %	Net Int.	Error %	Kratio	Z	A	F
C K	36.06	56.84	38.77	13.76	0.0749	1.1119	0.1869	1.0000
AlK	2.02	1.42	12.55	13.44	0.0183	0.9168	0.9752	1.0148
SiK	61.92	41.74	357.18	3.30	0.5713	0.9339	0.9878	1.0003

EDS Spot 5

kV: 10	Mag: 1437	Takeoff: 40.6	Live Time(s): 29.9	Amp Time(µs): 1.92	Resolution:(eV) 127.4

EDS Spot 5 - Det 1

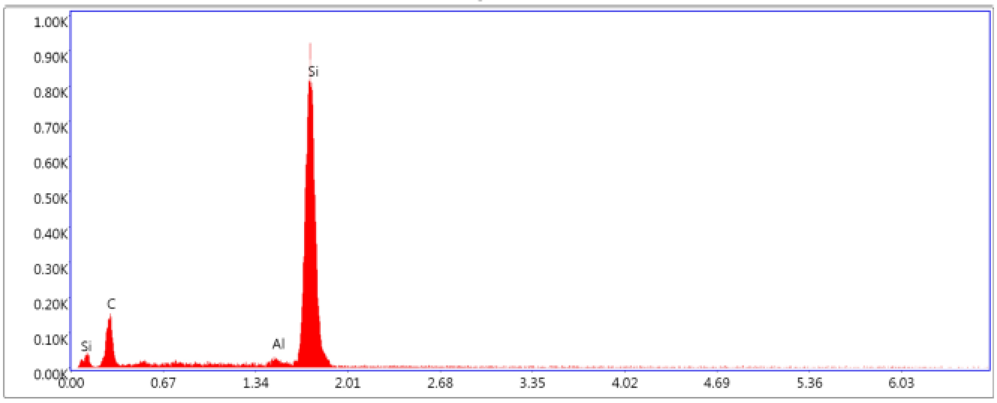

Lsec: 29.9 0 Cnts 0.000 keV Det: Octane Pro A

eZAF Smart Quant Results

Element	Weight %	Atomic %	Net Int.	Error %	Kratio	Z	A	F
C K	36.79	57.63	52.75	13.33	0.0771	1.1104	0.1887	1.0000
AlK	1.44	1.00	11.77	15.92	0.0130	0.9154	0.9749	1.0151
SiK	61.77	41.37	471.58	3.13	0.5703	0.9325	0.9899	1.0003

EDS Spot 6

kV: 10	Mag: 1437	Takeoff: 40.6	Live Time(s): 29.8	Amp Time(µs): 1.92	Resolution:(eV) 127.4

EDS Spot 6 - Det 1

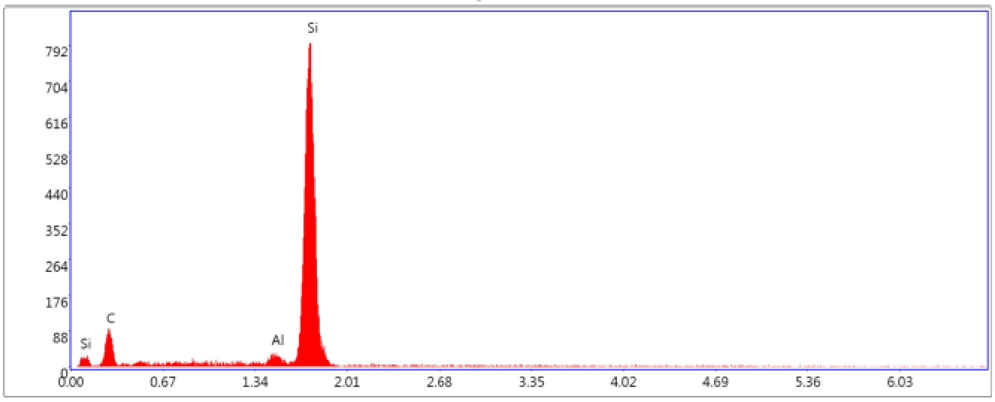

Lsec: 29.8 0 Cnts 0.000 keV Det: Octane Pro A

eZAF Smart Quant Results

Element	Weight %	Atomic %	Net Int.	Error %	Kratio	Z	A	F
C K	29.73	49.70	33.23	14.58	0.0570	1.1246	0.1703	1.0000
AlK	2.24	1.67	15.92	12.66	0.0206	0.9277	0.9766	1.0149
SiK	68.02	48.63	447.64	3.18	0.6353	0.9451	0.9878	1.0003

EDS Spot 7

kV: 10	Mag: 1437	Takeoff: 40.6	Live Time(s): 29.9	Amp Time(µs): 1.92	Resolution:(eV) 127.4

EDS Spot 7 - Det 1

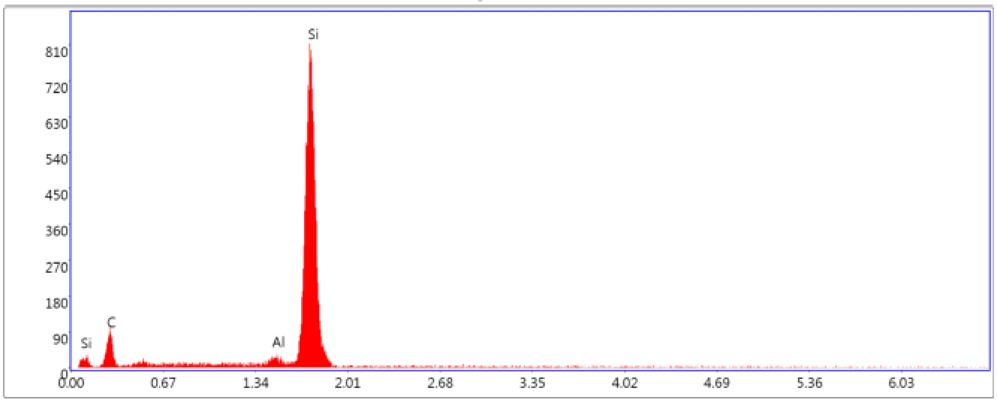

Lsec: 29.9 0 Cnts 0.000 keV Det: Octane Pro A

eZAF Smart Quant Results

Element	Weight %	Atomic %	Net Int.	Error %	Kratio	Z	A	F
C K	28.23	47.88	29.78	15.33	0.0531	1.1275	0.1667	1.0000
AlK	2.08	1.57	14.24	13.60	0.0192	0.9303	0.9770	1.0151
SiK	69.69	50.55	442.69	3.17	0.6532	0.9477	0.9885	1.0002

EDS Spot 8

kV: 10	Mag: 1437	Takeoff: 40.6	Live Time(s): 29.9	Amp Time(µs): 1.92	Resolution:(eV) 127.4

EDS Spot 8 - Det 1

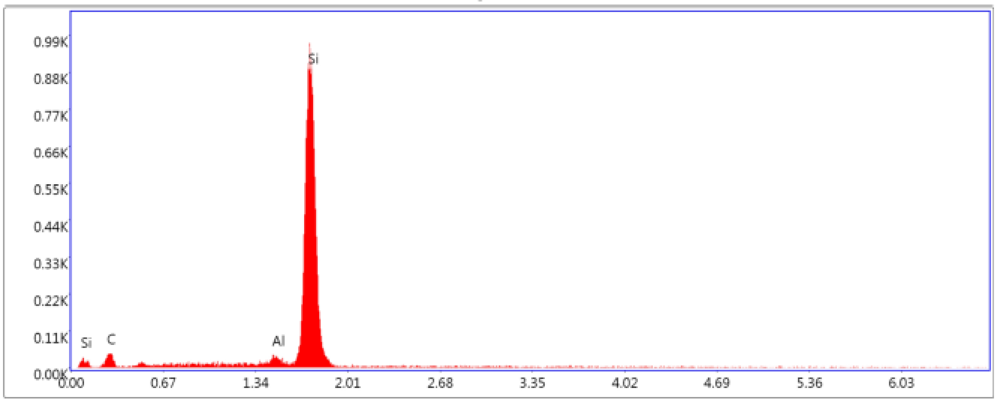

Lsec: 29.9 0 Cnts 0.000 keV Det: Octane Pro A

eZAF Smart Quant Results

Element	Weight %	Atomic %	Net Int.	Error %	Kratio	Z	A	F
C K	14.57	28.48	12.98	20.10	0.0236	1.1557	0.1402	1.0000
AlK	2.39	2.08	16.48	12.51	0.0227	0.9547	0.9802	1.0154
SiK	83.05	69.44	530.87	3.08	0.7991	0.9727	0.9891	1.0001

EDS Spot 9

kV: 10	Mag: 1437	Takeoff: 40.6	Live Time(s): 29.8	Amp Time(µs): 1.92	Resolution:(eV) 127.4

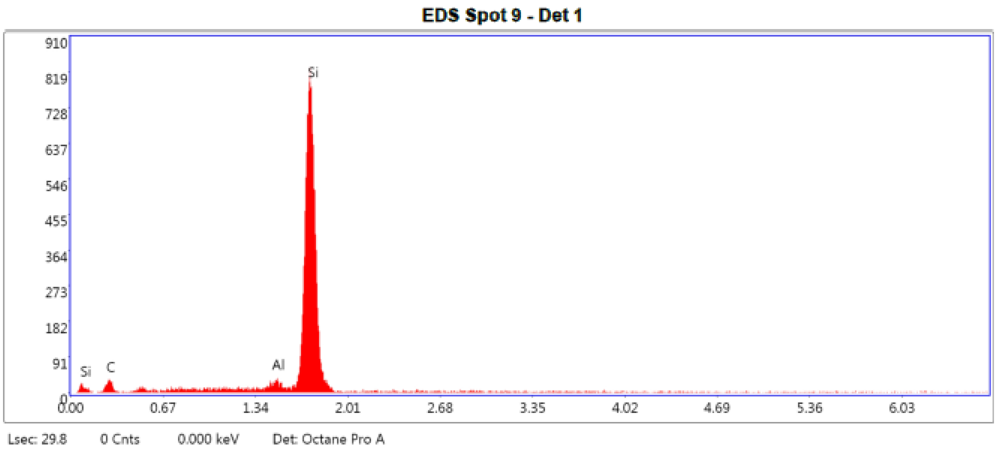

eZAF Smart Quant Results

Element	Weight %	Atomic %	Net Int.	Error %	Kratio	Z	A	F
C K	11.54	23.34	8.28	23.02	0.0182	1.1622	0.1355	1.0000
AlK	2.90	2.62	16.75	11.73	0.0278	0.9603	0.9810	1.0153
SiK	85.56	74.04	455.25	3.16	0.8268	0.9784	0.9875	1.0001

EDS Spot 10

kV: 10	Mag: 1437	Takeoff: 40.6	Live Time(s): 30	Amp Time(µs): 1.92	Resolution:(eV) 127.4

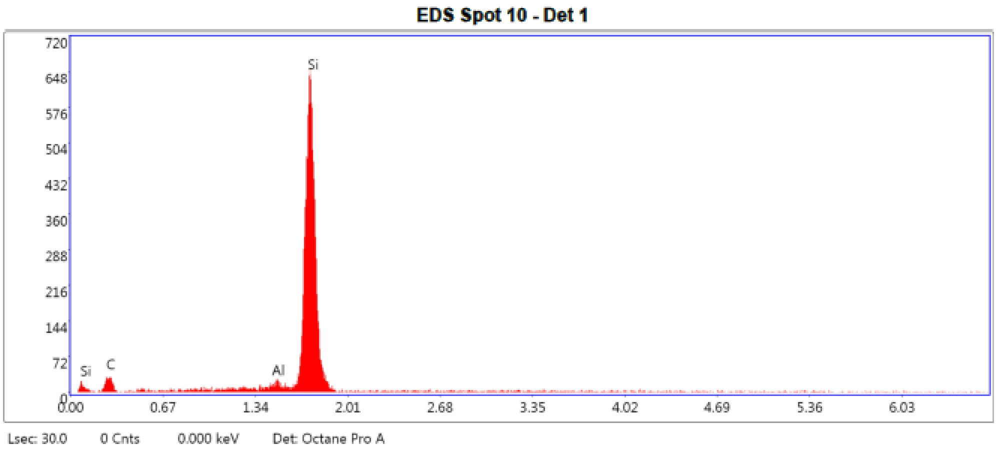

eZAF Smart Quant Results

Element	Weight %	Atomic %	Net Int.	Error %	Kratio	Z	A	F
C K	17.42	33.00	11.02	19.30	0.0291	1.1498	0.1451	1.0000
AlK	2.71	2.28	12.82	13.48	0.0256	0.9496	0.9796	1.0152
SiK	79.88	64.72	349.50	3.28	0.7633	0.9675	0.9875	1.0001

EDS Spot 11

kV: 10	Mag: 1437	Takeoff: 40.6	Live Time(s): 29.8	Amp Time(µs): 1.92	Resolution:(eV) 127.4

EDS Spot 11 - Det 1

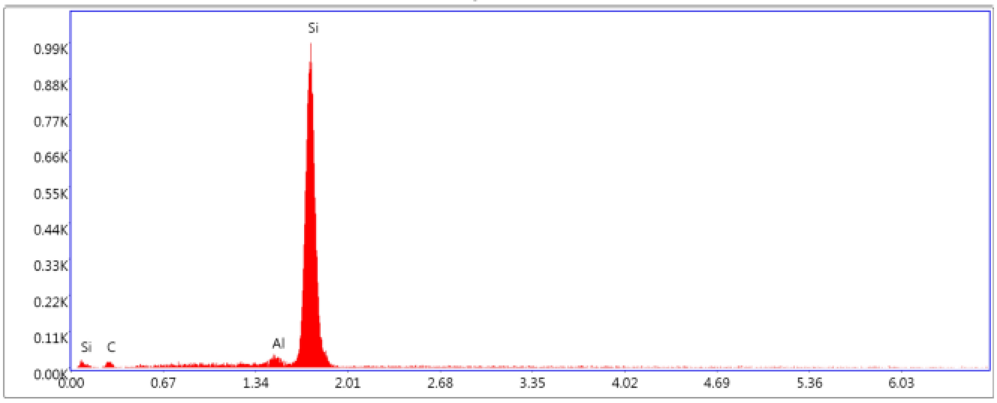

Lsec: 29.8 0 Cnts 0.000 keV Det: Octane Pro A

eZAF Smart Quant Results

Element	Weight %	Atomic %	Net Int.	Error %	Kratio	Z	A	F
C K	4.39	9.68	3.12	48.47	0.0065	1.1777	0.1253	1.0000
AlK	3.20	3.14	19.84	10.96	0.0311	0.9737	0.9827	1.0154
SiK	92.41	87.17	527.26	3.08	0.9052	0.9921	0.9873	1.0000

EDS Spot 12

kV: 10	Mag: 1437	Takeoff: 40.6	Live Time(s): 29.9	Amp Time(µs): 1.92	Resolution:(eV) 127.4

EDS Spot 12 - Det 1

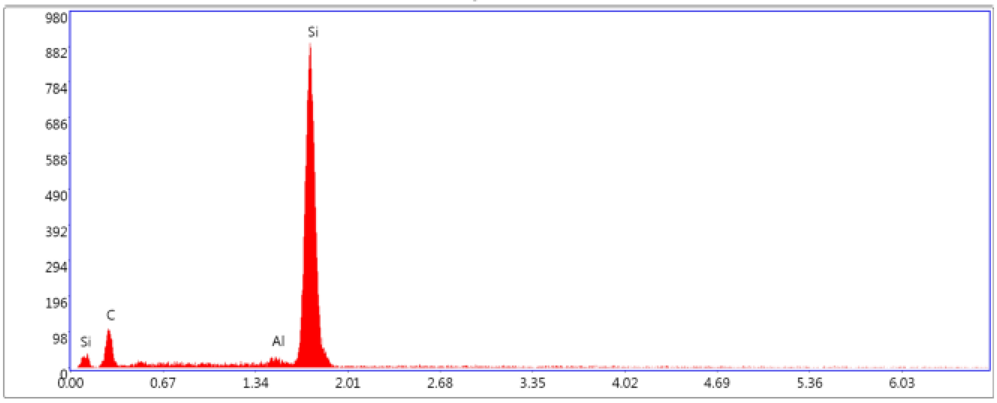

Lsec: 29.9 0 Cnts 0.000 keV Det: Octane Pro A

eZAF Smart Quant Results

Element	Weight %	Atomic %	Net Int.	Error %	Kratio	Z	A	F
C K	30.28	50.36	37.49	14.01	0.0584	1.1234	0.1715	1.0000
AlK	1.98	1.47	15.48	13.40	0.0182	0.9267	0.9765	1.0150
SiK	67.73	48.17	490.83	3.11	0.6325	0.9441	0.9887	1.0003

EDS Spot 13

kV: 10	Mag: 1437	Takeoff: 40.6	Live Time(s): 29.8	Amp Time(μs): 1.92	Resolution:(eV) 127.4

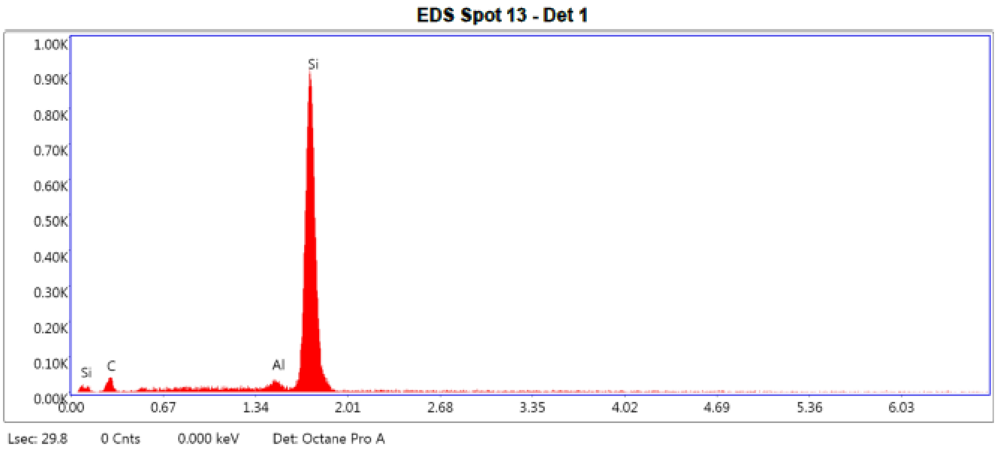

eZAF Smart Quant Results

Element	Weight %	Atomic %	Net Int.	Error %	Kratio	Z	A	F
C K	13.06	25.97	10.65	22.14	0.0209	1.1589	0.1378	1.0000
AlK	2.55	2.26	16.43	12.48	0.0243	0.9574	0.9806	1.0154
SiK	84.39	71.77	502.30	3.11	0.8140	0.9755	0.9887	1.0001

Area 2

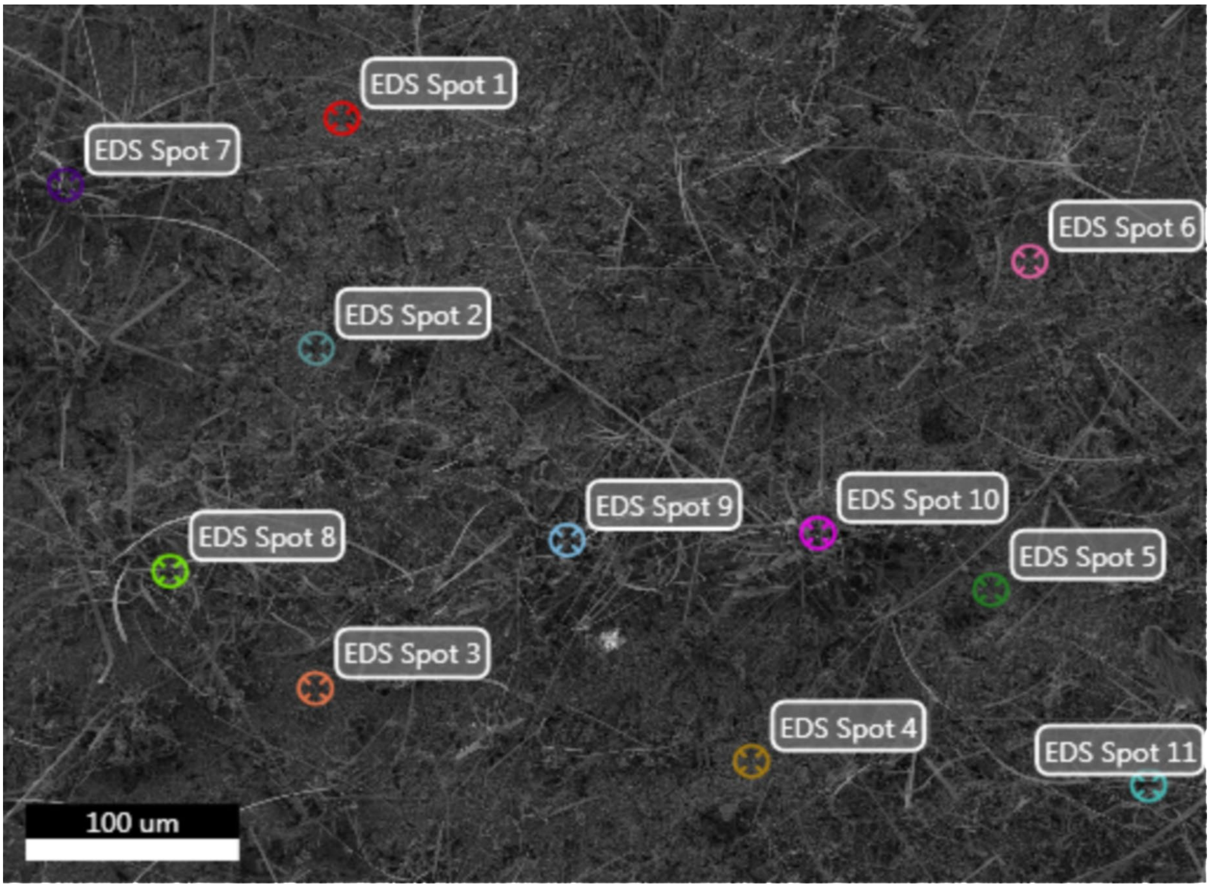
EDS Spot 1
EDS Spot 7
EDS Spot 6
EDS Spot 2
EDS Spot 8
EDS Spot 9
EDS Spot 10
EDS Spot 5
EDS Spot 3
EDS Spot 4
EDS Spot 11
100 um

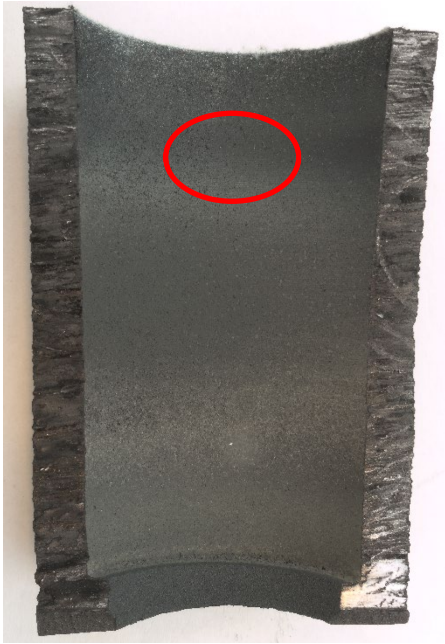

EDS Spot 1

kV: 10	Mag: 195	Takeoff: 40.7	Live Time(s): 30	Amp Time(µs): 1.92	Resolution:(eV) 127.4

EDS Spot 1 - Det 1

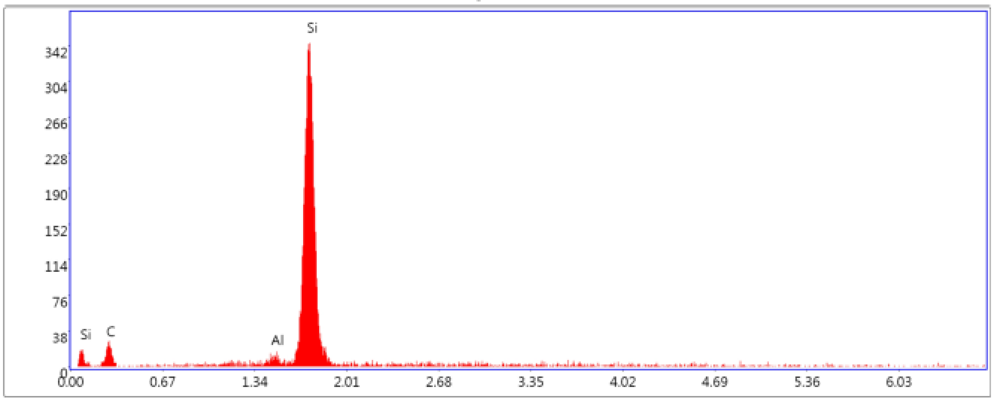

eZAF Smart Quant Results

Element	Weight %	Atomic %	Net Int.	Error %	Kratio	Z	A	F
C K	19.56	36.22	7.26	23.32	0.0334	1.1453	0.1492	1.0000
AlK	2.61	2.15	7.04	21.16	0.0245	0.9457	0.9791	1.0151
SiK	77.83	61.63	194.28	3.74	0.7407	0.9634	0.9877	1.0002

EDS Spot 2

kV: 10	Mag: 195	Takeoff: 40.7	Live Time(s): 29.8	Amp Time(µs): 1.92	Resolution:(eV) 127.4

EDS Spot 2 - Det 1

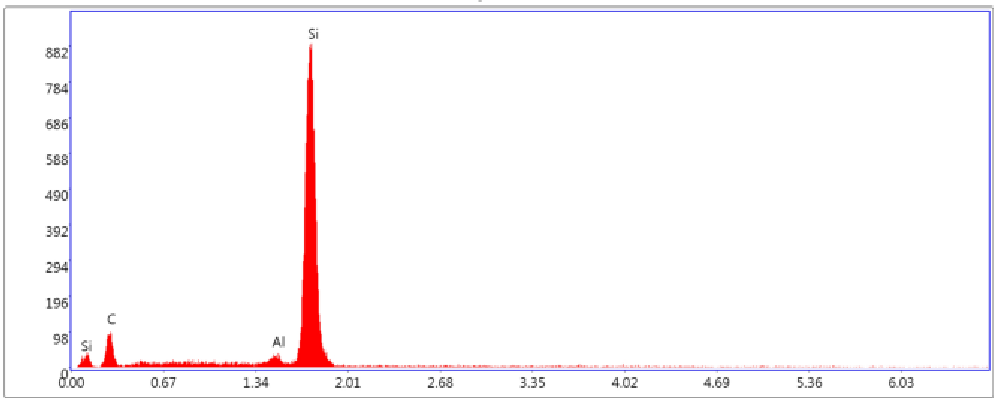

eZAF Smart Quant Results

Element	Weight %	Atomic %	Net Int.	Error %	Kratio	Z	A	F
C K	27.86	47.42	33.49	14.66	0.0522	1.1283	0.1662	1.0000
AlK	2.29	1.73	17.90	12.44	0.0211	0.9310	0.9771	1.0150
SiK	69.85	50.85	506.72	3.12	0.6547	0.9484	0.9878	1.0002

EDS Spot 3

kV: 10	Mag: 195	Takeoff: 40.7	Live Time(s): 29.9	Amp Time(µs): 1.92	Resolution:(eV) 127.4

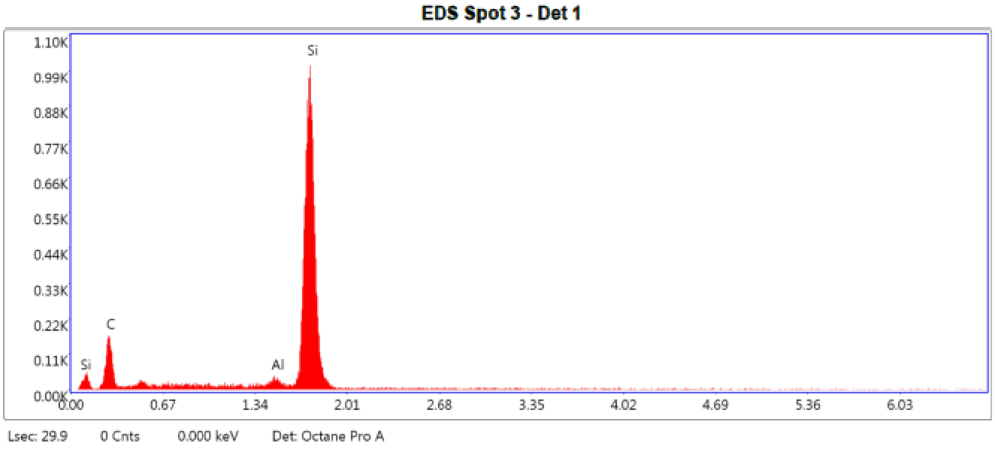

eZAF Smart Quant Results

Element	Weight %	Atomic %	Net Int.	Error %	Kratio	Z	A	F
C K	35.17	55.90	55.60	13.30	0.0723	1.1136	0.1845	1.0000
AlK	1.82	1.28	16.79	13.58	0.0165	0.9182	0.9754	1.0149
SiK	63.01	42.82	541.62	3.09	0.5828	0.9354	0.9887	1.0003

EDS Spot 4

kV: 10	Mag: 195	Takeoff: 40.7	Live Time(s): 29.9	Amp Time(µs): 1.92	Resolution:(eV) 127.4

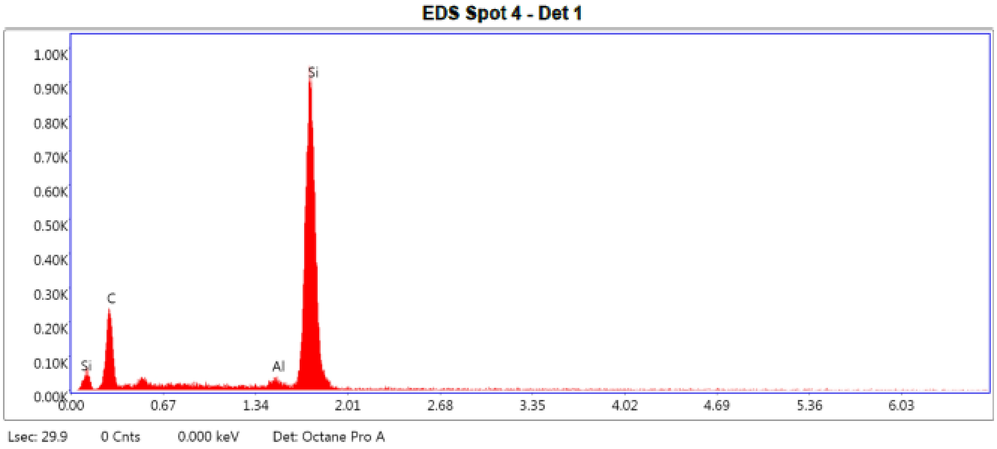

eZAF Smart Quant Results

Element	Weight %	Atomic %	Net Int.	Error %	Kratio	Z	A	F
C K	43.88	64.62	87.82	12.04	0.1019	1.0969	0.2117	1.0000
AlK	1.55	1.02	15.81	13.87	0.0139	0.9037	0.9734	1.0146
SiK	54.56	34.36	517.07	3.12	0.4970	0.9205	0.9887	1.0005

EDS Spot 5

kV: 10	Mag: 195	Takeoff: 40.7	Live Time(s): 29.8	Amp Time(µs): 1.92	Resolution:(eV) 127.4

EDS Spot 5 - Det 1

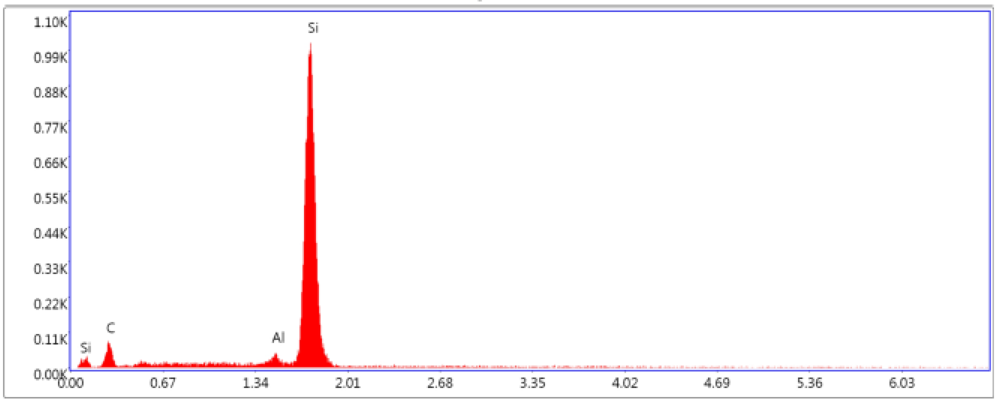

Lsec: 29.8 0 Cnts 0.000 keV Det: Octane Pro A

eZAF Smart Quant Results

Element	Weight %	Atomic %	Net Int.	Error %	Kratio	Z	A	F
C K	21.20	38.59	23.75	15.93	0.0369	1.1418	0.1523	1.0000
AlK	2.33	1.89	18.58	11.79	0.0218	0.9427	0.9787	1.0152
SiK	76.47	59.52	564.79	3.05	0.7260	0.9604	0.9885	1.0002

EDS Spot 6

kV: 10	Mag: 195	Takeoff: 40.7	Live Time(s): 29.8	Amp Time(µs): 1.92	Resolution:(eV) 127.4

EDS Spot 6 - Det 1

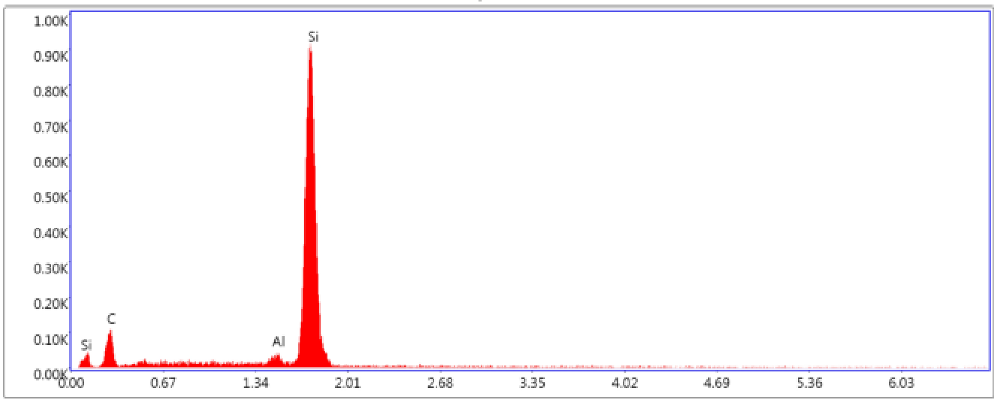

Lsec: 29.8 0 Cnts 0.000 keV Det: Octane Pro A

eZAF Smart Quant Results

Element	Weight %	Atomic %	Net Int.	Error %	Kratio	Z	A	F
C K	28.96	48.77	36.06	14.54	0.0550	1.1261	0.1686	1.0000
AlK	2.03	1.52	16.20	12.84	0.0187	0.9290	0.9768	1.0151
SiK	69.02	49.71	511.59	3.10	0.6461	0.9464	0.9887	1.0002

EDS Spot 7

kV: 10	Mag: 195	Takeoff: 40.7	Live Time(s): 29.9	Amp Time(µs): 1.92	Resolution:(eV) 127.4

EDS Spot 7 - Det 1

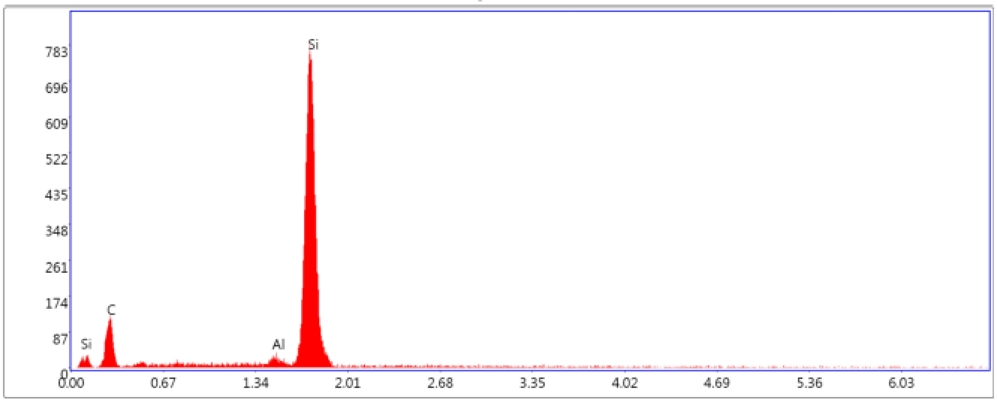

eZAF Smart Quant Results

Element	Weight %	Atomic %	Net Int.	Error %	Kratio	Z	A	F
C K	36.00	56.78	46.27	13.54	0.0747	1.1120	0.1867	1.0000
AlK	1.69	1.18	12.52	13.86	0.0153	0.9168	0.9752	1.0150
SiK	62.32	42.04	430.39	3.18	0.5757	0.9340	0.9891	1.0003

EDS Spot 8

kV: 10	Mag: 195	Takeoff: 40.7	Live Time(s): 29.9	Amp Time(µs): 1.92	Resolution:(eV) 127.4

EDS Spot 8 - Det 1

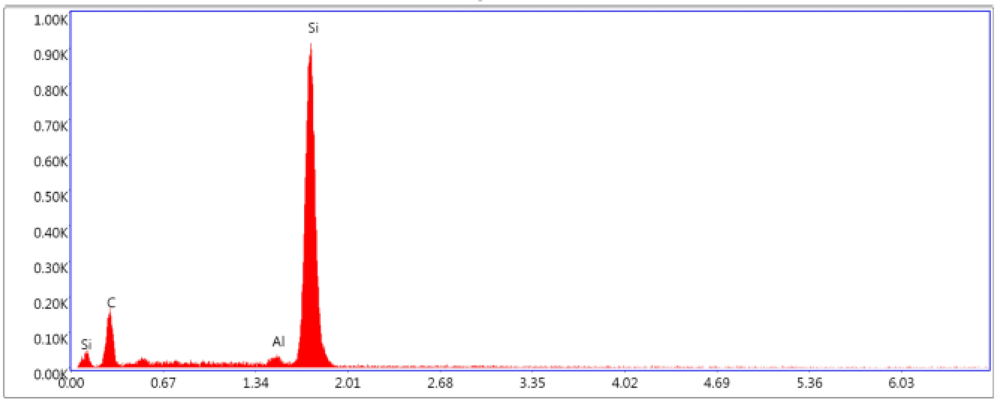

eZAF Smart Quant Results

Element	Weight %	Atomic %	Net Int.	Error %	Kratio	Z	A	F
C K	37.25	58.10	59.53	13.16	0.0786	1.1095	0.1903	1.0000
AlK	1.63	1.13	14.72	13.86	0.0147	0.9147	0.9749	1.0149
SiK	61.13	40.77	515.03	3.08	0.5634	0.9318	0.9892	1.0003

EDS Spot 9

kV: 10	Mag: 195	Takeoff: 40.7	Live Time(s): 29.8	Amp Time(µs): 1.92	Resolution:(eV) 127.4

EDS Spot 9 - Det 1

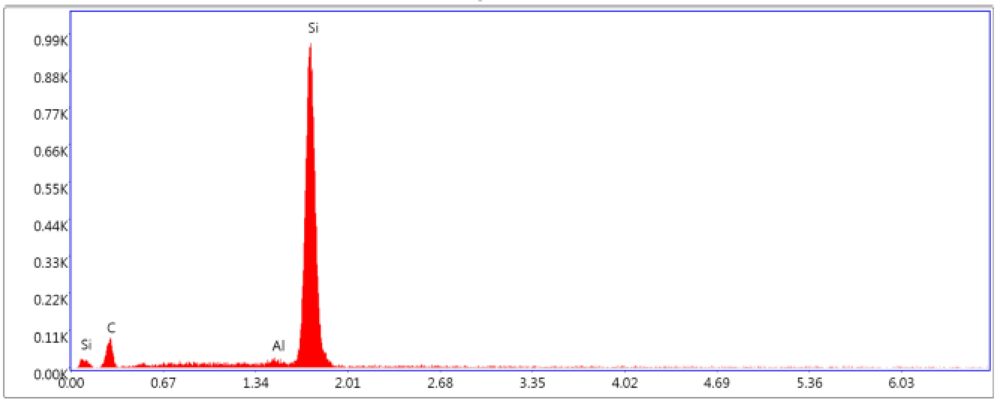

Lsec: 29.8 0 Cnts 0.000 keV Det: Octane Pro A

eZAF Smart Quant Results

Element	Weight %	Atomic %	Net Int.	Error %	Kratio	Z	A	F
C K	23.93	42.36	26.70	15.64	0.0428	1.1360	0.1574	1.0000
AlK	1.47	1.16	11.31	16.87	0.0137	0.9377	0.9779	1.0156
SiK	74.60	56.48	532.34	3.04	0.7065	0.9553	0.9914	1.0002

EDS Spot 10

kV: 10	Mag: 195	Takeoff: 40.7	Live Time(s): 29.9	Amp Time(µs): 1.92	Resolution:(eV) 127.4

EDS Spot 10 - Det 1

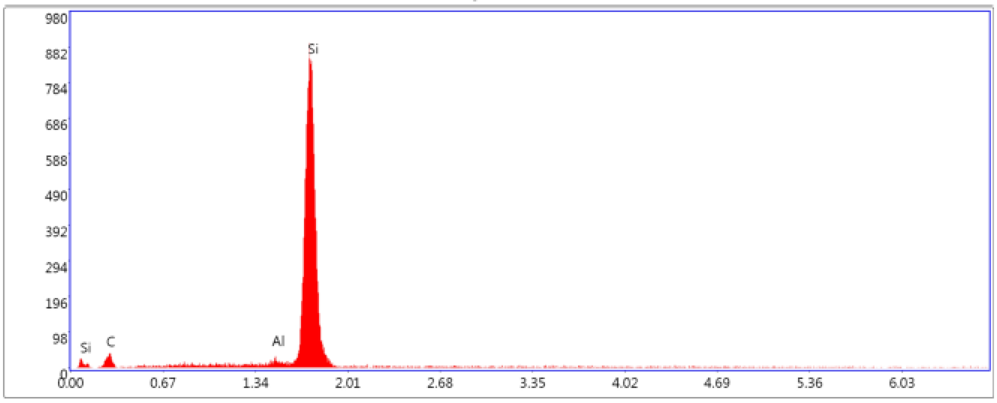

Lsec: 29.9 0 Cnts 0.000 keV Det: Octane Pro A

eZAF Smart Quant Results

Element	Weight %	Atomic %	Net Int.	Error %	Kratio	Z	A	F
C K	13.01	25.89	10.09	22.27	0.0207	1.1588	0.1376	1.0000
AlK	1.68	1.49	10.27	17.01	0.0160	0.9574	0.9805	1.0158
SiK	85.32	72.62	484.72	3.07	0.8256	0.9754	0.9920	1.0001

EDS Spot 11

kV: 10	Mag: 195	Takeoff: 40.7	Live Time(s): 29.9	Amp Time(µs): 1.92	Resolution:(eV) 127.4

EDS Spot 11 - Det 1

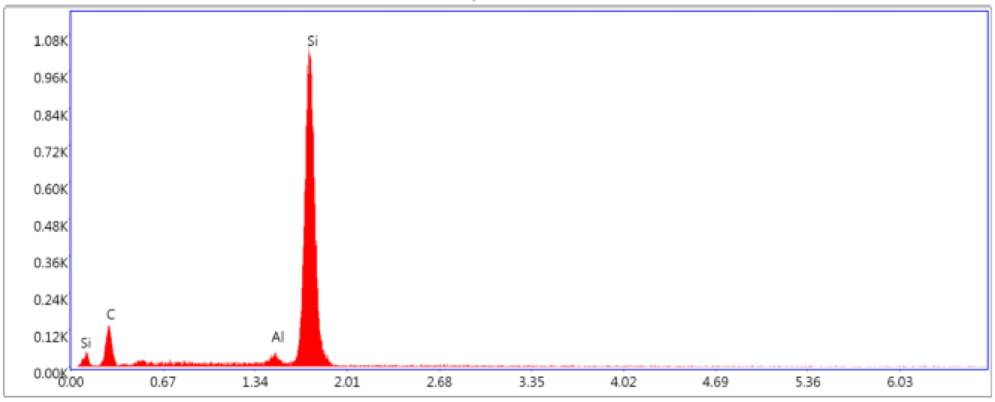

Lsec: 29.9 0 Cnts 0.000 keV Det: Octane Pro A

eZAF Smart Quant Results

Element	Weight %	Atomic %	Net Int.	Error %	Kratio	Z	A	F
C K	30.37	50.46	44.35	13.75	0.0587	1.1233	0.1720	1.0000
AlK	2.10	1.55	19.25	12.24	0.0193	0.9266	0.9765	1.0150
SiK	67.54	47.99	575.22	3.07	0.6303	0.9440	0.9882	1.0003

2% CH_4 in H_2 at 1650°C

Area 1

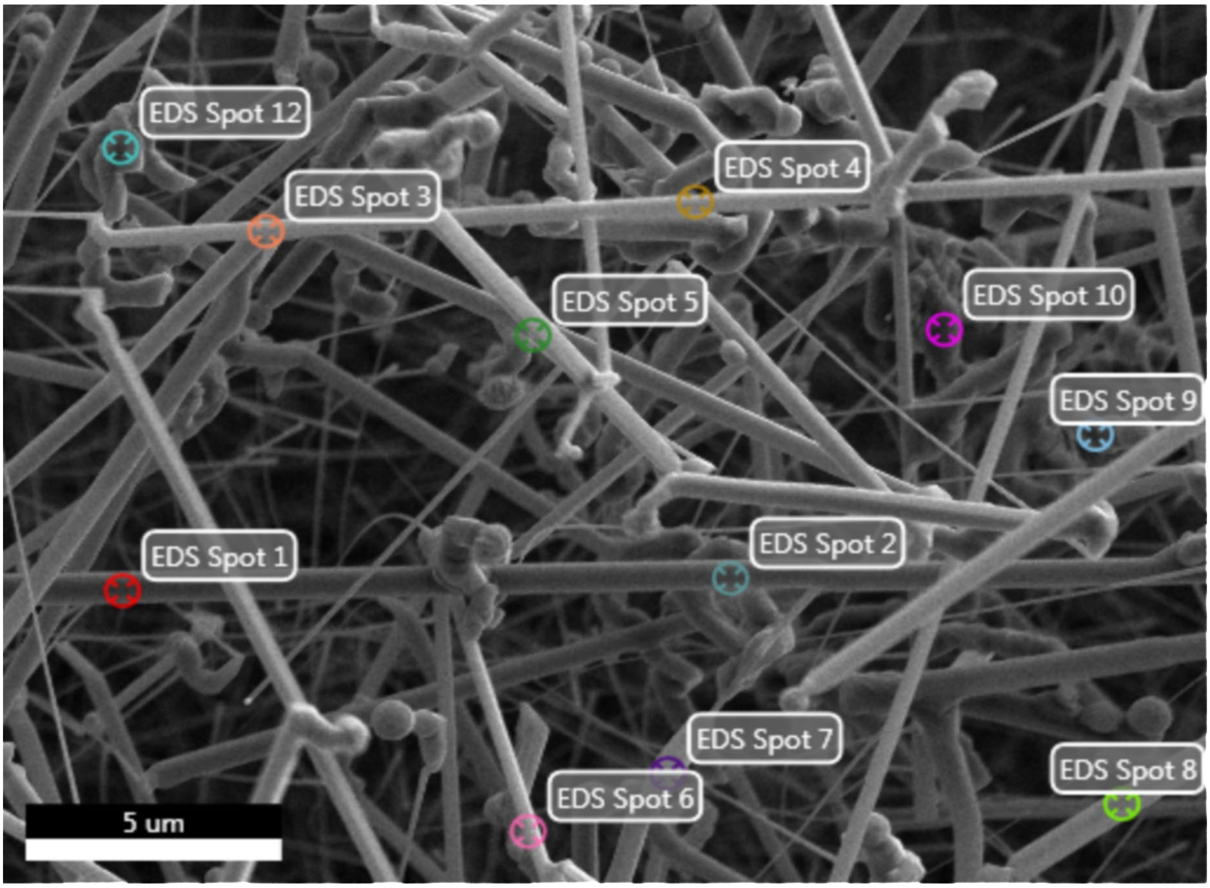

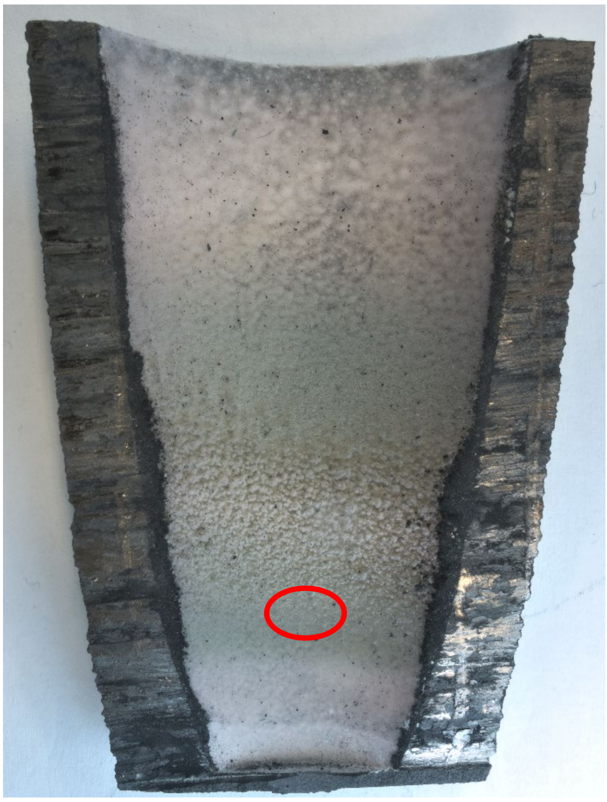

EDS Spot 1

kV: 10 Mag: 4672 Takeoff: 47.7 Live Time(s): 30 Amp Time(µs): 1.92 Resolution:(eV) 127.4

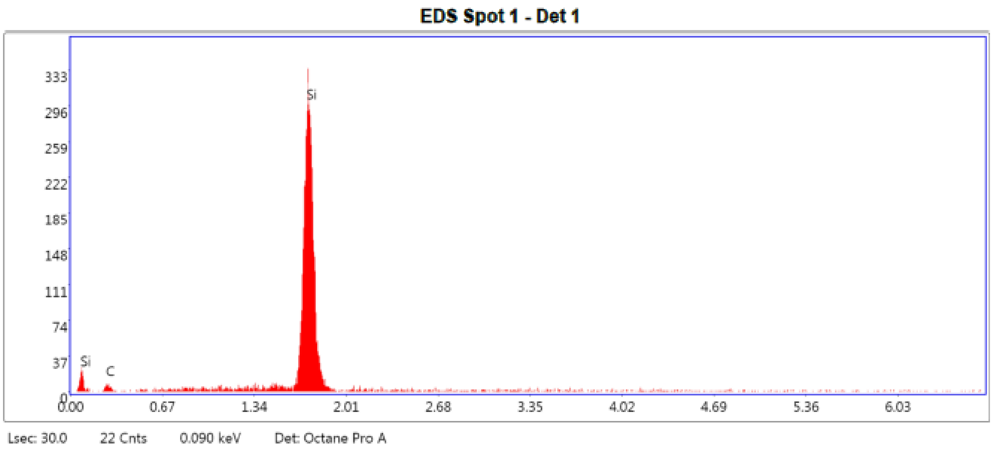

eZAF Smart Quant Results

Element	Weight %	Atomic %	Net Int.	Error %	Kratio	Z	A	F
C K	5.20	11.37	1.32	80.51	0.0085	1.1751	0.1398	1.0000
SiK	94.80	88.63	171.04	3.68	0.9379	0.9899	0.9994	1.0000

EDS Spot 2

kV: 10 Mag: 4672 Takeoff: 47.7 Live Time(s): 29.8 Amp Time(µs): 1.92 Resolution:(eV) 127.4

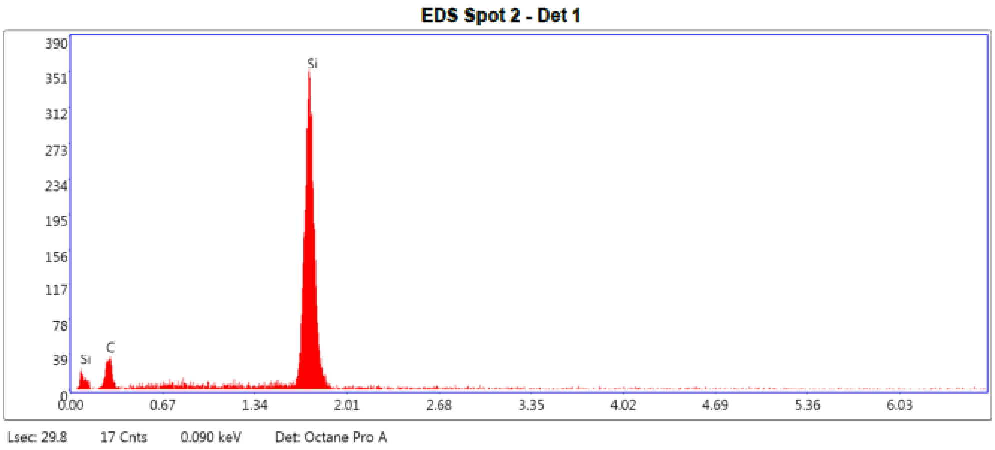

eZAF Smart Quant Results

Element	Weight %	Atomic %	Net Int.	Error %	Kratio	Z	A	F
C K	25.41	44.34	11.45	18.94	0.0511	1.1327	0.1775	1.0000
SiK	74.59	55.66	186.77	3.69	0.7083	0.9524	0.9971	1.0002

EDS Spot 3

kV: 10	Mag: 4672	Takeoff: 47.7	Live Time(s): 30	Amp Time(µs): 1.92	Resolution:(eV) 127.4

EDS Spot 3 - Det 1

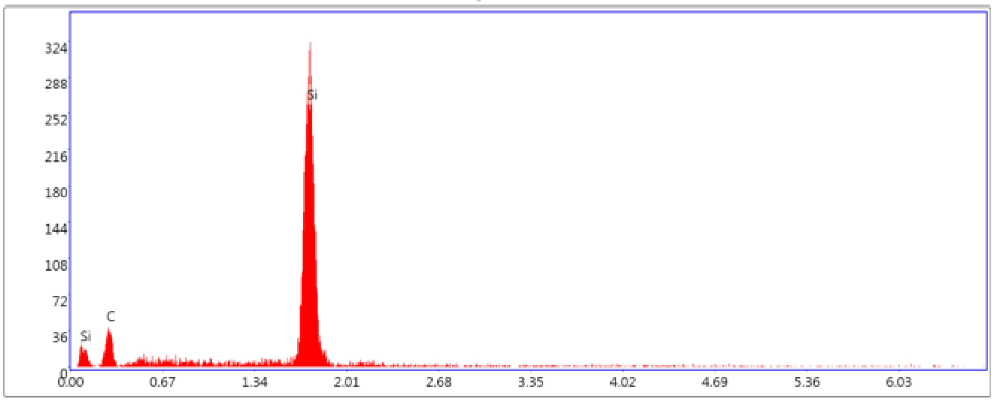

Lsec: 30.0 20 Cnts 0.090 keV Det: Octane Pro A

eZAF Smart Quant Results

Element	Weight %	Atomic %	Net Int.	Error %	Kratio	Z	A	F
C K	28.21	47.89	12.62	18.60	0.0586	1.1271	0.1842	1.0000
SiK	71.79	52.11	171.85	3.81	0.6782	0.9474	0.9968	1.0002

EDS Spot 4

kV: 10	Mag: 4672	Takeoff: 47.7	Live Time(s): 30	Amp Time(µs): 1.92	Resolution:(eV) 127.4

EDS Spot 4 - Det 1

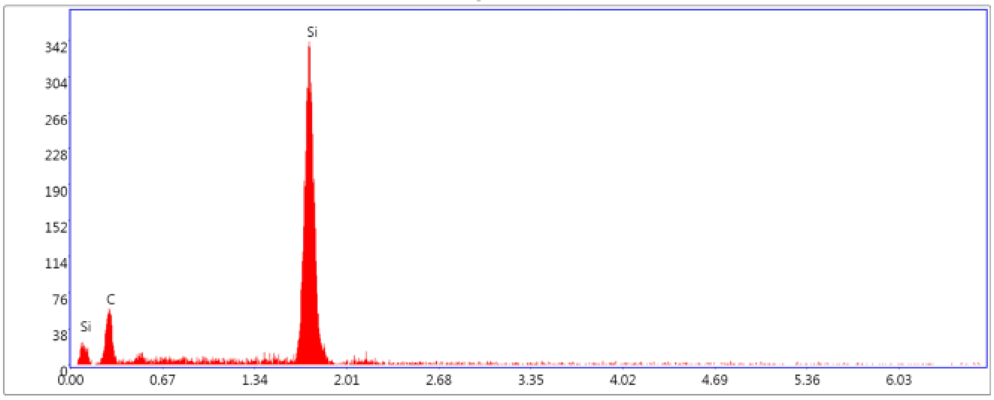

Lsec: 30.0 18 Cnts 0.090 keV Det: Octane Pro A

eZAF Smart Quant Results

Element	Weight %	Atomic %	Net Int.	Error %	Kratio	Z	A	F
C K	35.29	56.05	20.15	15.71	0.0800	1.1131	0.2037	1.0000
SiK	64.71	43.95	178.64	3.79	0.6027	0.9349	0.9961	1.0003

EDS Spot 5

kV: 10	Mag: 4672	Takeoff: 47.7	Live Time(s): 30	Amp Time(µs): 1.92	Resolution:(eV) 127.4

EDS Spot 5 - Det 1

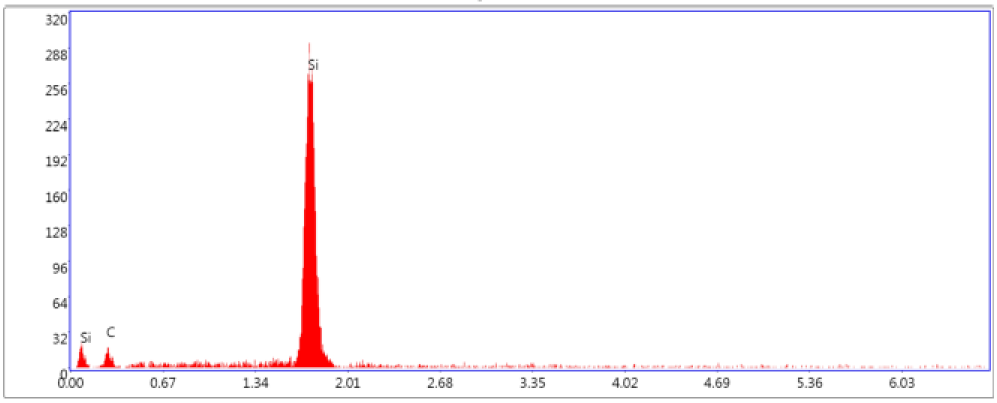

Lsec: 30.0 21 Cnts 0.090 keV Det: Octane Pro A

eZAF Smart Quant Results

Element	Weight %	Atomic %	Net Int.	Error %	Kratio	Z	A	F
C K	14.62	28.60	4.27	29.15	0.0262	1.1550	0.1553	1.0000
SiK	85.38	71.40	158.62	3.80	0.8288	0.9721	0.9983	1.0001

EDS Spot 6

kV: 10	Mag: 4672	Takeoff: 47.7	Live Time(s): 29.7	Amp Time(µs): 1.92	Resolution:(eV) 127.4

EDS Spot 6 - Det 1

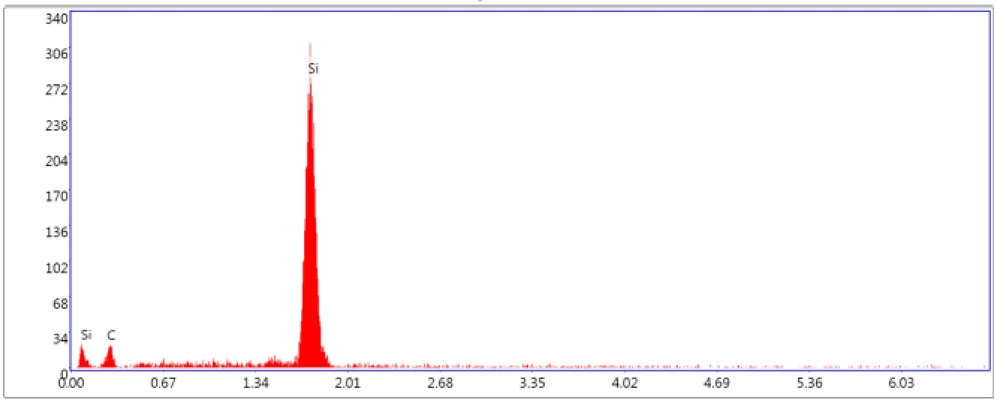

Lsec: 29.7 16 Cnts 0.090 keV Det: Octane Pro A

eZAF Smart Quant Results

Element	Weight %	Atomic %	Net Int.	Error %	Kratio	Z	A	F
C K	19.35	35.93	6.30	23.71	0.0364	1.1452	0.1643	1.0000
SiK	80.65	64.07	157.76	3.81	0.7754	0.9634	0.9978	1.0001

EDS Spot 7

kV: 10	Mag: 4672	Takeoff: 47.7	Live Time(s): 30	Amp Time(μs): 1.92	Resolution:(eV) 127.4

EDS Spot 7 - Det 1

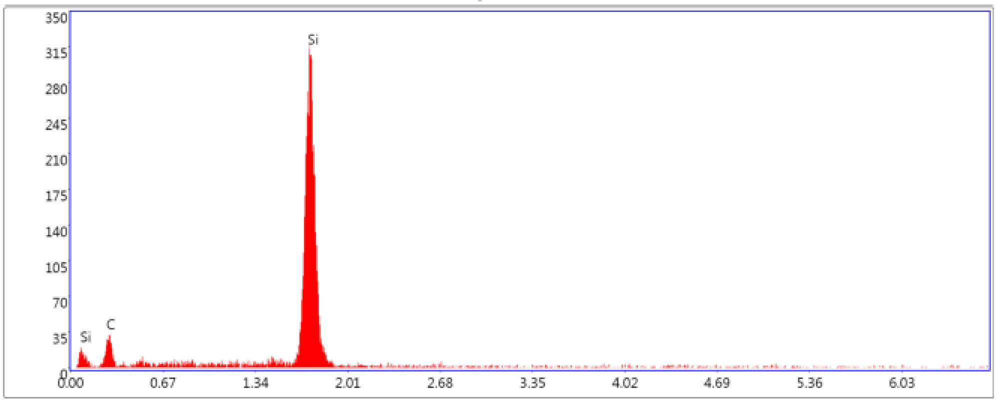

Lsec: 30.0 12 Cnts 0.090 keV Det: Octane Pro A

eZAF Smart Quant Results

Element	Weight %	Atomic %	Net Int.	Error %	Kratio	Z	A	F
C K	25.02	43.82	9.84	19.41	0.0501	1.1335	0.1766	1.0000
SiK	74.98	56.18	164.67	3.73	0.7126	0.9531	0.9972	1.0002

EDS Spot 8

kV: 10	Mag: 4672	Takeoff: 47.7	Live Time(s): 30	Amp Time(μs): 1.92	Resolution:(eV) 127.4

EDS Spot 8 - Det 1

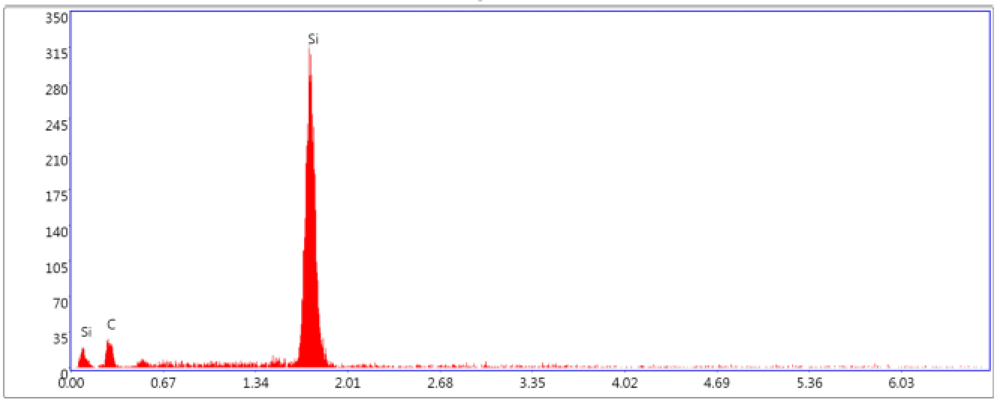

Lsec: 30.0 18 Cnts 0.090 keV Det: Octane Pro A

eZAF Smart Quant Results

Element	Weight %	Atomic %	Net Int.	Error %	Kratio	Z	A	F
C K	23.79	42.20	9.03	19.67	0.0470	1.1360	0.1738	1.0000
SiK	76.21	57.80	164.26	3.79	0.7261	0.9553	0.9973	1.0002

EDS Spot 9

kV: 10	Mag: 4672	Takeoff: 47.7	Live Time(s): 30	Amp Time(µs): 1.92	Resolution:(eV) 127.4

EDS Spot 9 - Det 1

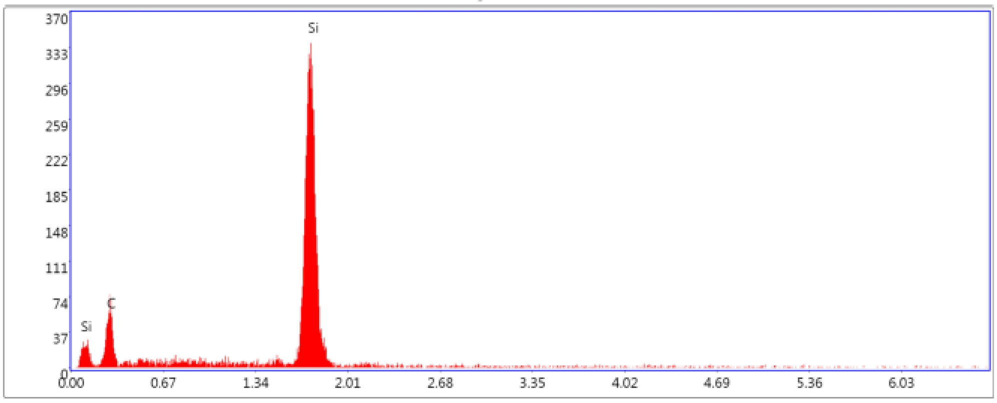

Lsec: 30.0 21 Cnts 0.090 keV Det: Octane Pro A

eZAF Smart Quant Results

Element	Weight %	Atomic %	Net Int.	Error %	Kratio	Z	A	F
C K	37.60	58.49	24.14	14.47	0.0879	1.1085	0.2109	1.0000
SiK	62.40	41.51	186.95	3.64	0.5785	0.9309	0.9958	1.0003

EDS Spot 10

kV: 10	Mag: 4672	Takeoff: 47.7	Live Time(s): 30	Amp Time(µs): 1.92	Resolution:(eV) 127.4

EDS Spot 10 - Det 1

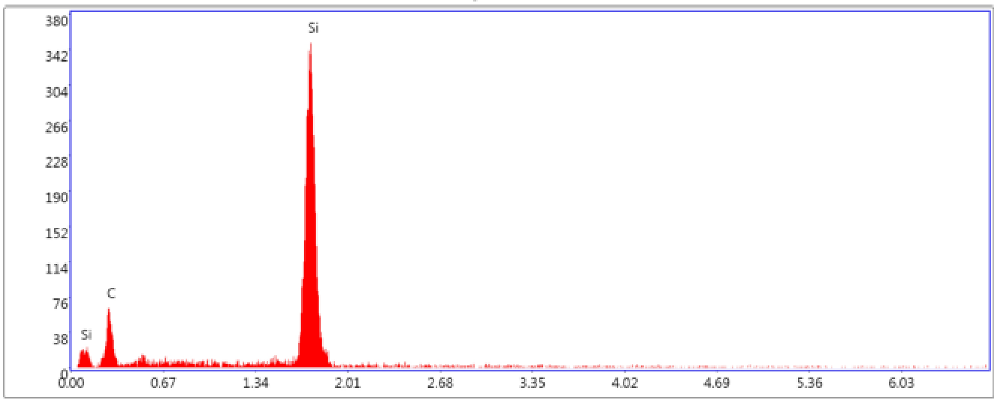

Lsec: 30.0 20 Cnts 0.090 keV Det: Octane Pro A

eZAF Smart Quant Results

Element	Weight %	Atomic %	Net Int.	Error %	Kratio	Z	A	F
C K	35.71	56.51	21.55	15.03	0.0814	1.1122	0.2049	1.0000
SiK	64.29	43.49	186.28	3.68	0.5982	0.9342	0.9960	1.0003

EDS Spot 12

kV: 10	Mag: 4672	Takeoff: 47.7	Live Time(s): 29.7	Amp Time(µs): 1.92	Resolution:(eV) 127.4

EDS Spot 12 - Det 1

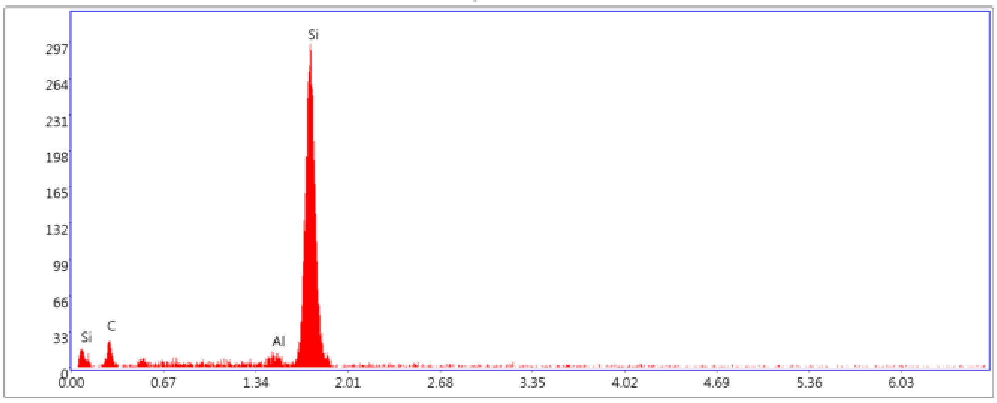

Lsec: 29.7 16 Cnts 0.090 keV Det: Octane Pro A

eZAF Smart Quant Results

Element	Weight %	Atomic %	Net Int.	Error %	Kratio	Z	A	F
C K	19.13	35.58	6.67	23.50	0.0362	1.1463	0.1650	1.0000
AlK	3.07	2.54	6.90	17.71	0.0290	0.9465	0.9816	1.0149
SiK	77.80	61.87	160.77	3.89	0.7410	0.9643	0.9876	1.0001

Area 2

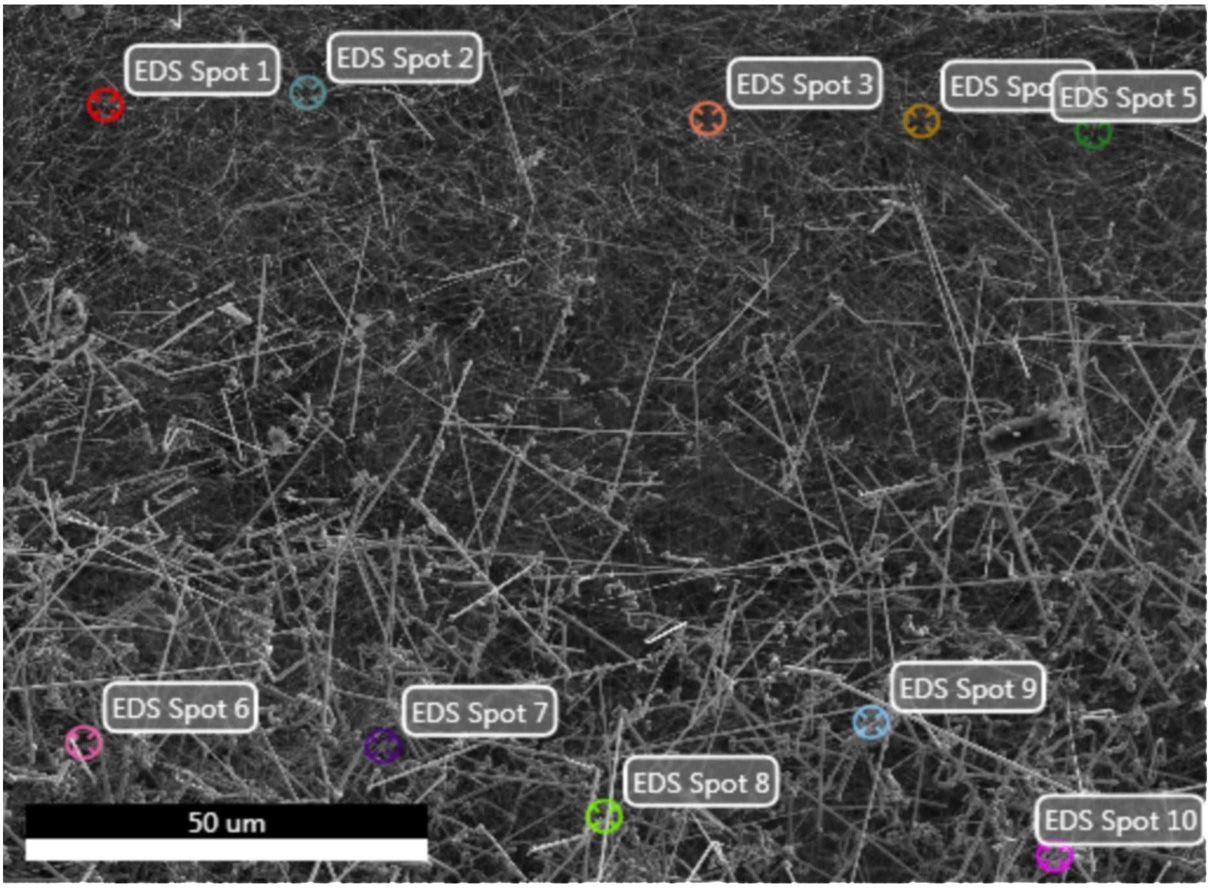
EDS Spot 1
EDS Spot 2
EDS Spot 3
EDS Spo
EDS Spot 5
EDS Spot 6
EDS Spot 7
EDS Spot 8
EDS Spot 9
EDS Spot 10
50 um

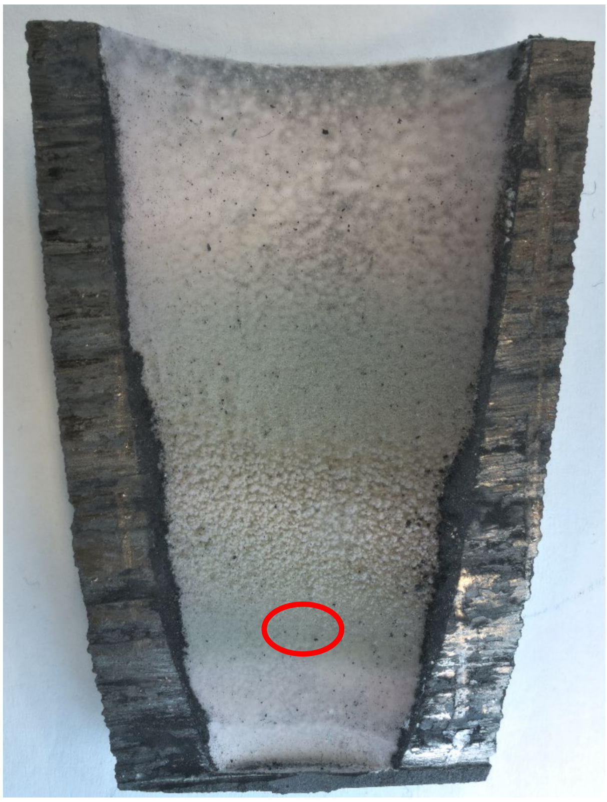

EDS Spot 1

kV: 10 Mag: 734 Takeoff: 47.7 Live Time(s): 30 Amp Time(µs): 1.92 Resolution:(eV) 127.4

EDS Spot 1 - Det 1

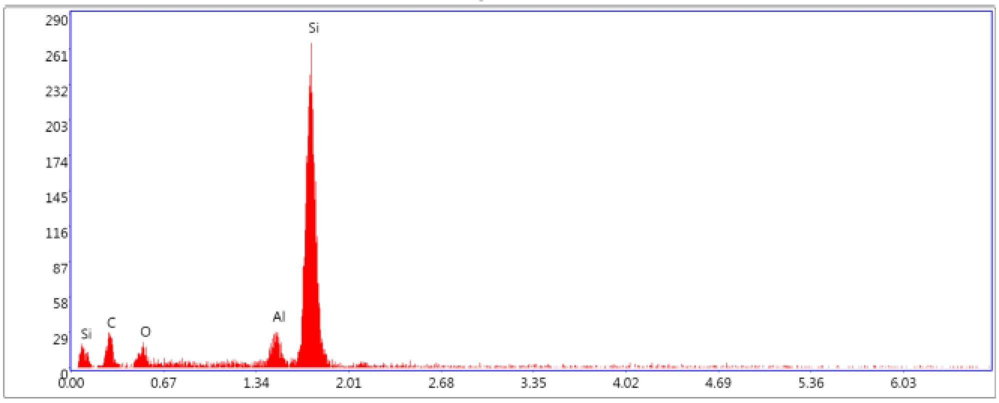

Lsec: 30.0 17 Cnts 0.090 keV Det: Octane Pro A

eZAF Smart Quant Results

Element	Weight %	Atomic %	Net Int.	Error %	Kratio	Z	A	F
C K	23.02	40.12	8.80	19.78	0.0478	1.1337	0.1833	1.0000
O K	4.09	5.35	5.84	25.54	0.0205	1.0725	0.4663	1.0000
AlK	6.67	5.17	14.65	11.34	0.0617	0.9354	0.9762	1.0129
SiK	66.22	49.35	132.79	4.25	0.6136	0.9530	0.9722	1.0002

EDS Spot 2

kV: 10 Mag: 734 Takeoff: 47.7 Live Time(s): 30 Amp Time(µs): 1.92 Resolution:(eV) 127.4

EDS Spot 2 - Det 1

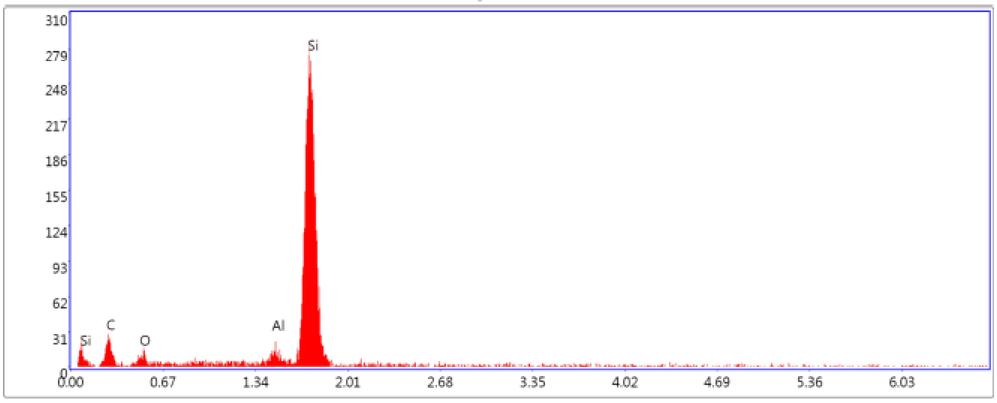

Lsec: 30.0 20 Cnts 0.090 keV Det: Octane Pro A

eZAF Smart Quant Results

Element	Weight %	Atomic %	Net Int.	Error %	Kratio	Z	A	F
C K	22.73	40.14	8.87	19.66	0.0459	1.1356	0.1776	1.0000
O K	2.49	3.30	3.70	32.87	0.0123	1.0744	0.4611	1.0000
AlK	3.26	2.56	7.57	17.24	0.0303	0.9372	0.9778	1.0145
SiK	71.51	54.00	153.02	3.96	0.6724	0.9548	0.9846	1.0002

EDS Spot 3

kV: 10	Mag: 734	Takeoff: 47.7	Live Time(s): 29.7	Amp Time(μs): 1.92	Resolution:(eV) 127.4

EDS Spot 3 - Det 1

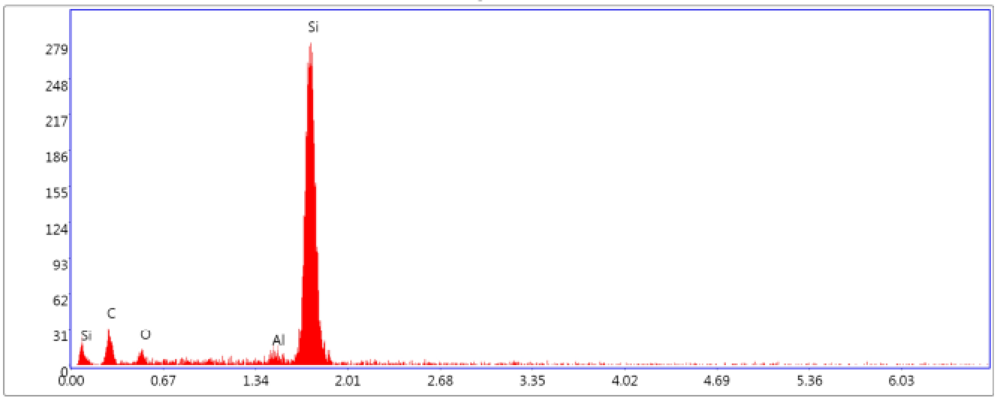

Lsec: 29.7 19 Cnts 0.090 keV Det: Octane Pro A

eZAF Smart Quant Results

Element	Weight %	Atomic %	Net Int.	Error %	Kratio	Z	A	F
C K	21.85	39.07	8.36	20.10	0.0433	1.1380	0.1740	1.0000
O K	1.89	2.53	2.81	48.21	0.0094	1.0768	0.4610	1.0000
AlK	2.45	1.95	5.69	22.83	0.0228	0.9394	0.9786	1.0150
SiK	73.82	56.45	158.63	3.91	0.6979	0.9570	0.9879	1.0002

EDS Spot 4

kV: 10	Mag: 734	Takeoff: 47.7	Live Time(s): 30	Amp Time(μs): 1.92	Resolution:(eV) 127.4

EDS Spot 4 - Det 1

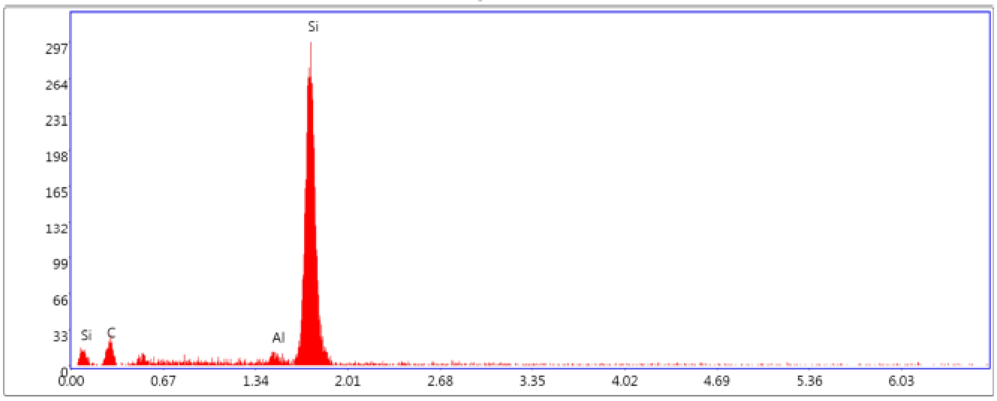

Lsec: 30.0 13 Cnts 0.090 keV Det: Octane Pro A

eZAF Smart Quant Results

Element	Weight %	Atomic %	Net Int.	Error %	Kratio	Z	A	F
C K	21.80	39.43	7.83	20.28	0.0423	1.1406	0.1702	1.0000
AlK	2.51	2.02	5.62	21.16	0.0235	0.9416	0.9810	1.0151
SiK	75.70	58.56	156.46	3.86	0.7184	0.9593	0.9892	1.0002

EDS Spot 5

kV: 10	Mag: 734	Takeoff: 47.7	Live Time(s): 30	Amp Time(µs): 1.92	Resolution:(eV) 127.4

EDS Spot 5 - Det 1

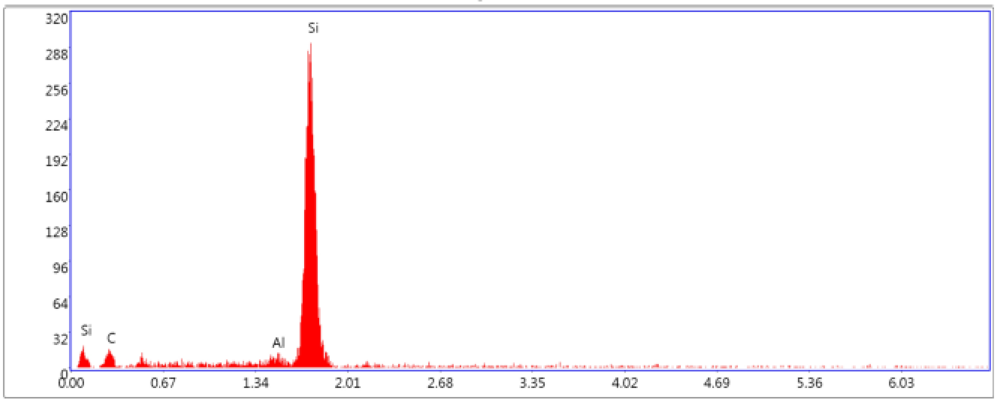

Lsec: 30.0   17 Cnts   0.090 keV   Det: Octane Pro A

eZAF Smart Quant Results

Element	Weight %	Atomic %	Net Int.	Error %	Kratio	Z	A	F
C K	16.21	31.12	5.09	24.41	0.0297	1.1523	0.1590	1.0000
AlK	2.77	2.37	5.81	21.37	0.0263	0.9518	0.9822	1.0152
SiK	81.02	66.52	156.71	3.90	0.7771	0.9697	0.9889	1.0001

EDS Spot 6

kV: 10	Mag: 734	Takeoff: 47.7	Live Time(s): 29.8	Amp Time(µs): 1.92	Resolution:(eV) 127.4

EDS Spot 6 - Det 1

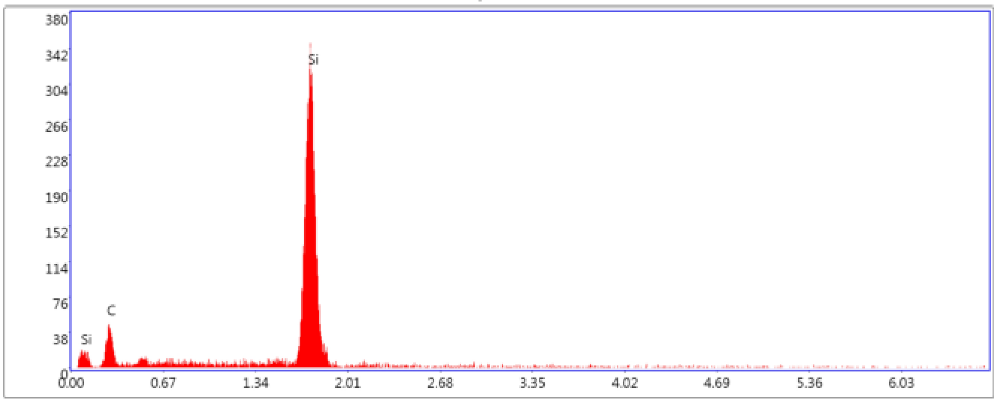

Lsec: 29.8   13 Cnts   0.090 keV   Det: Octane Pro A

eZAF Smart Quant Results

Element	Weight %	Atomic %	Net Int.	Error %	Kratio	Z	A	F
C K	30.36	50.48	15.40	16.48	0.0646	1.1228	0.1895	1.0000
SiK	69.64	49.52	183.62	3.68	0.6552	0.9436	0.9966	1.0003

EDS Spot 7

kV: 10	Mag: 734	Takeoff: 47.7	Live Time(s): 30	Amp Time(µs): 1.92	Resolution:(eV) 127.4

EDS Spot 7 - Det 1

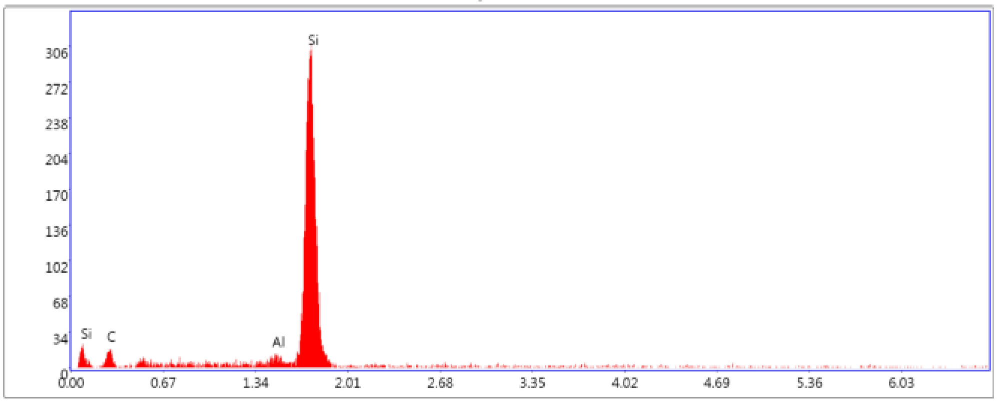

Lsec: 30.0 23 Cnts 0.090 keV Det: Octane Pro A

eZAF Smart Quant Results

Element	Weight %	Atomic %	Net Int.	Error %	Kratio	Z	A	F
C K	15.28	29.64	5.03	24.61	0.0278	1.1543	0.1573	1.0000
AlK	2.97	2.56	6.60	18.55	0.0282	0.9535	0.9824	1.0151
SiK	81.75	67.80	167.25	3.81	0.7850	0.9714	0.9884	1.0001

EDS Spot 8

kV: 10	Mag: 734	Takeoff: 47.7	Live Time(s): 30	Amp Time(µs): 1.92	Resolution:(eV) 127.4

EDS Spot 8 - Det 1

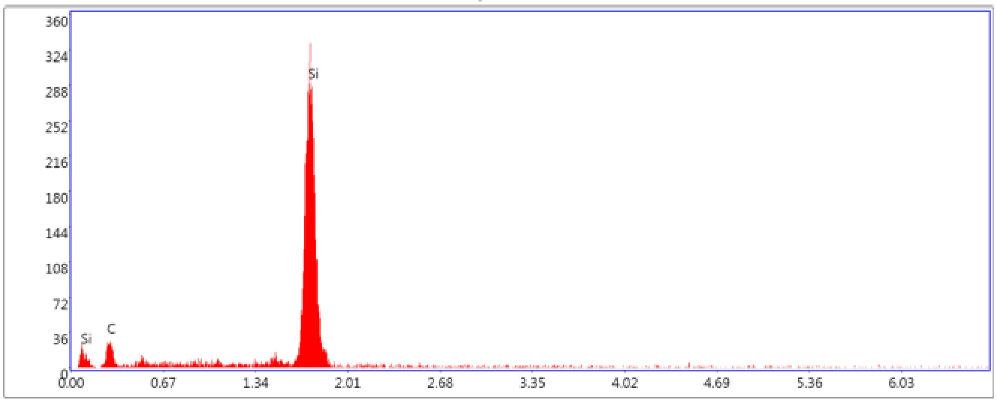

Lsec: 30.0 18 Cnts 0.090 keV Det: Octane Pro A

eZAF Smart Quant Results

Element	Weight %	Atomic %	Net Int.	Error %	Kratio	Z	A	F
C K	22.46	40.39	8.51	19.95	0.0437	1.1387	0.1707	1.0000
SiK	77.54	59.61	169.88	3.76	0.7407	0.9577	0.9975	1.0002

EDS Spot 9

kV: 10	Mag: 734	Takeoff: 47.7	Live Time(s): 30	Amp Time(µs): 1.92	Resolution:(eV) 127.4

EDS Spot 9 - Det 1

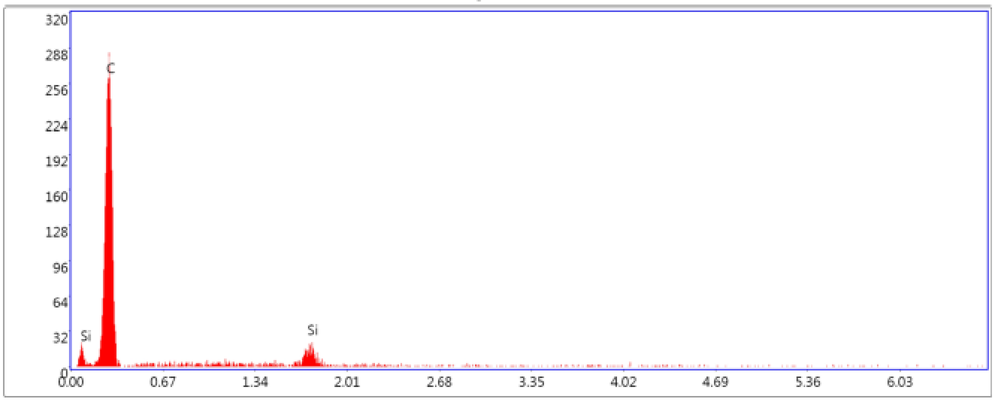

Lsec: 30.0 17 Cnts 0.090 keV Det: Octane Pro A

eZAF Smart Quant Results

Element	Weight %	Atomic %	Net Int.	Error %	Kratio	Z	A	F
C K	93.50	97.11	108.46	5.90	0.7407	1.0102	0.7842	1.0000
SiK	6.50	2.89	9.39	13.90	0.0545	0.8430	0.9909	1.0041

EDS Spot 10

kV: 10	Mag: 734	Takeoff: 47.7	Live Time(s): 30	Amp Time(µs): 1.92	Resolution:(eV) 127.4

EDS Spot 10 - Det 1

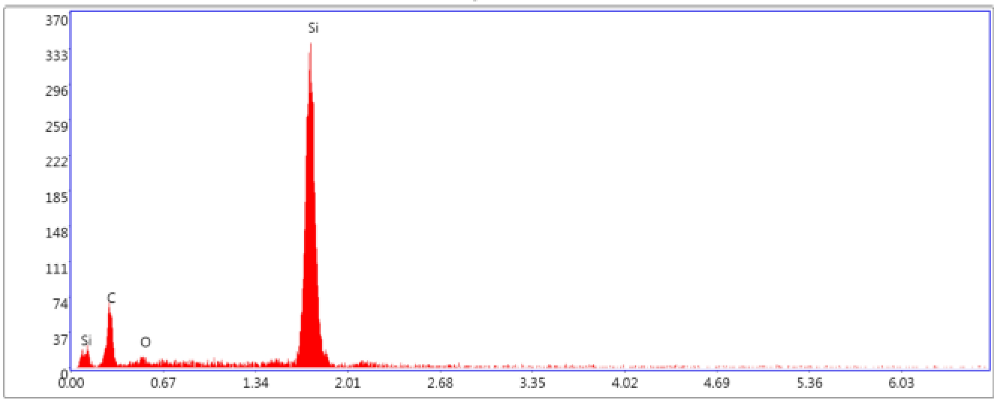

Lsec: 30.0 20 Cnts 0.090 keV Det: Octane Pro A

eZAF Smart Quant Results

Element	Weight %	Atomic %	Net Int.	Error %	Kratio	Z	A	F
C K	35.29	55.51	21.42	15.14	0.0818	1.1107	0.2087	1.0000
O K	1.89	2.23	3.49	33.55	0.0086	1.0503	0.4333	1.0000
SiK	62.82	42.26	179.58	3.76	0.5829	0.9328	0.9945	1.0003

2% CH_4 in H_2 at 1750°C

Area 1

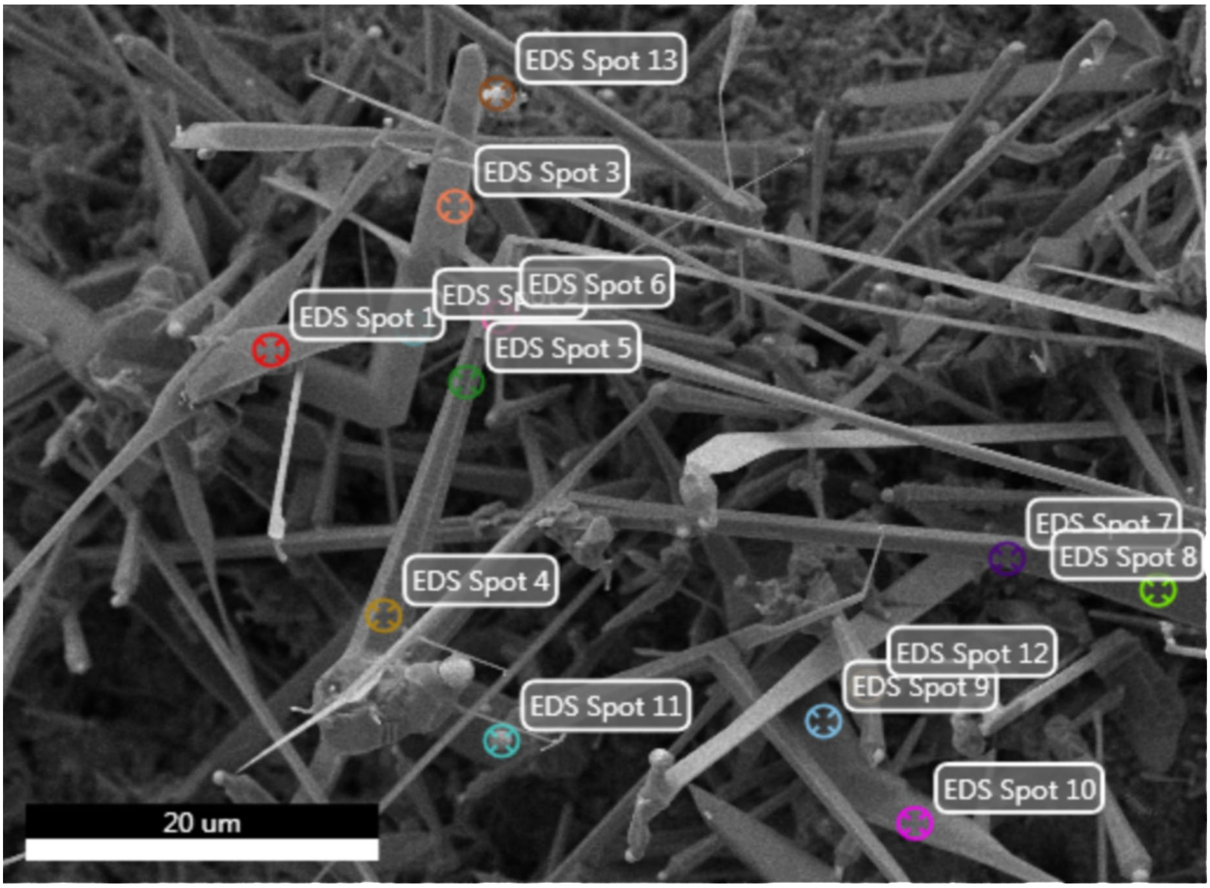

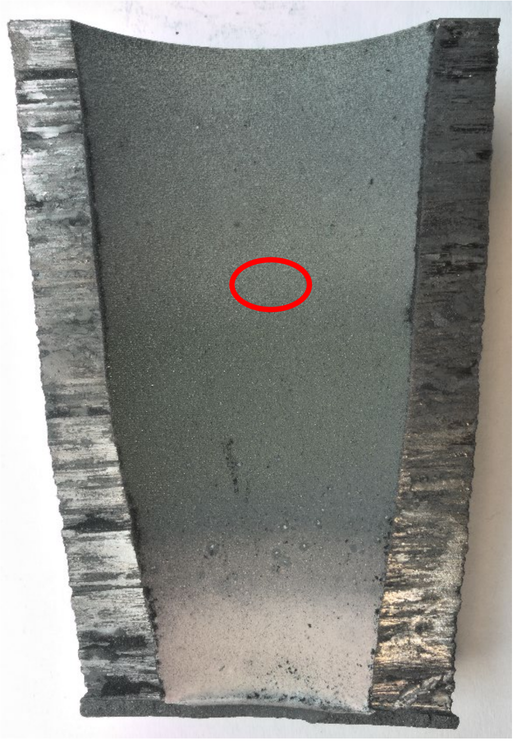

EDS Spot 1

kV: 10	Mag: 1612	Takeoff: 33.9	Live Time(s): 29.8	Amp Time(µs): 1.92	Resolution:(eV) 127.4

EDS Spot 1 - Det 1

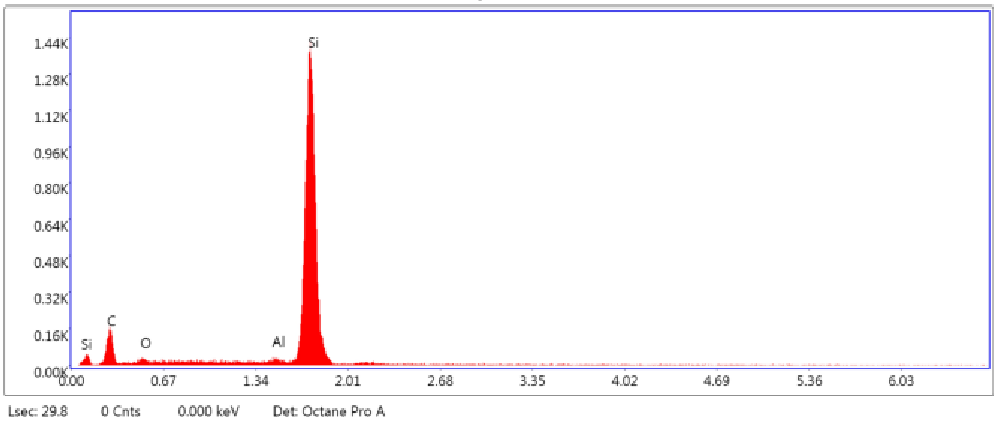

eZAF Smart Quant Results

Element	Weight %	Atomic %	Net Int.	Error %	Kratio	Z	A	F
C K	30.87	50.84	52.07	13.77	0.0536	1.1209	0.1548	1.0000
O K	0.87	1.08	5.29	45.78	0.0034	1.0602	0.3650	1.0000
AlK	0.77	0.57	9.52	26.26	0.0070	0.9245	0.9710	1.0156
SiK	67.48	47.52	779.11	2.90	0.6303	0.9419	0.9911	1.0003

EDS Spot 2

kV: 10	Mag: 1612	Takeoff: 33.9	Live Time(s): 29.9	Amp Time(µs): 1.92	Resolution:(eV) 127.4

EDS Spot 2 - Det 1

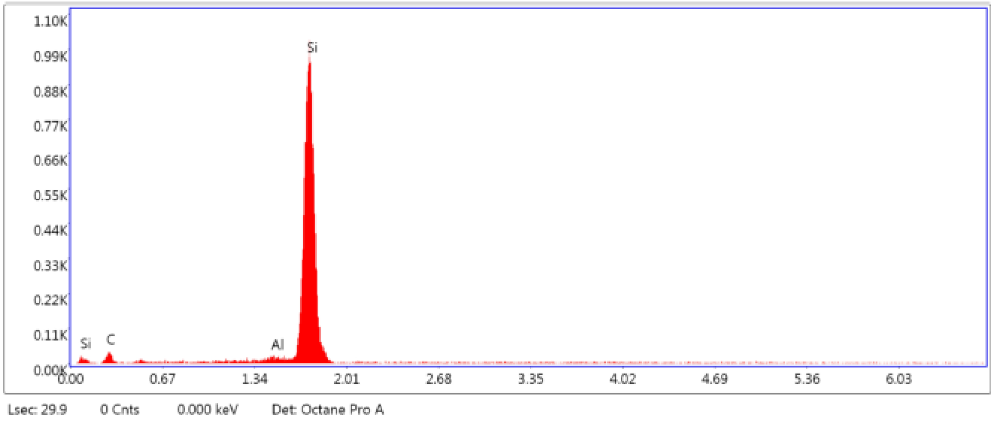

eZAF Smart Quant Results

Element	Weight %	Atomic %	Net Int.	Error %	Kratio	Z	A	F
C K	13.03	25.93	9.86	20.11	0.0184	1.1589	0.1221	1.0000
AlK	2.39	2.11	16.85	11.42	0.0227	0.9575	0.9775	1.0155
SiK	84.58	71.96	554.01	3.04	0.8150	0.9755	0.9876	1.0001

EDS Spot 3

kV: 10	Mag: 1612	Takeoff: 33.9	Live Time(s): 29.9	Amp Time(µs): 1.92	Resolution:(eV) 127.4

EDS Spot 3 - Det 1

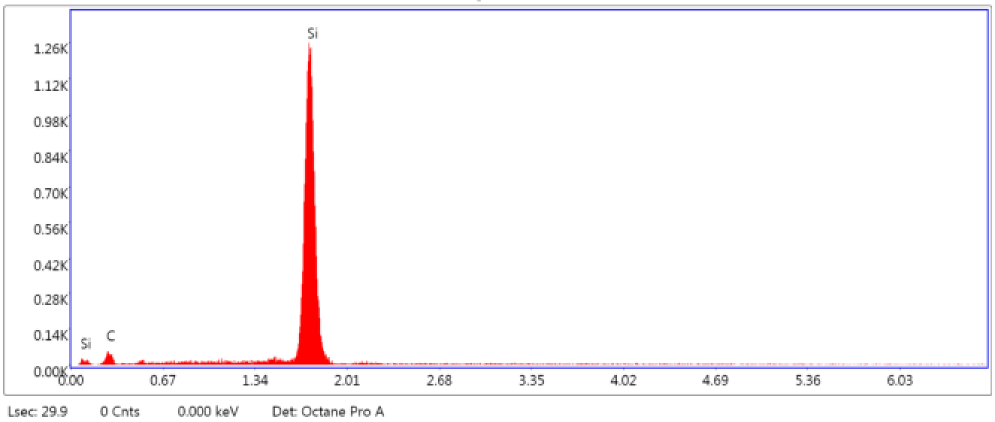

eZAF Smart Quant Results

Element	Weight %	Atomic %	Net Int.	Error %	Kratio	Z	A	F
C K	14.49	28.38	13.90	19.02	0.0207	1.1553	0.1236	1.0000
SiK	85.51	71.62	709.13	2.84	0.8298	0.9724	0.9978	1.0001

EDS Spot 4

kV: 10	Mag: 1612	Takeoff: 33.9	Live Time(s): 29.8	Amp Time(µs): 1.92	Resolution:(eV) 127.4

EDS Spot 4 - Det 1

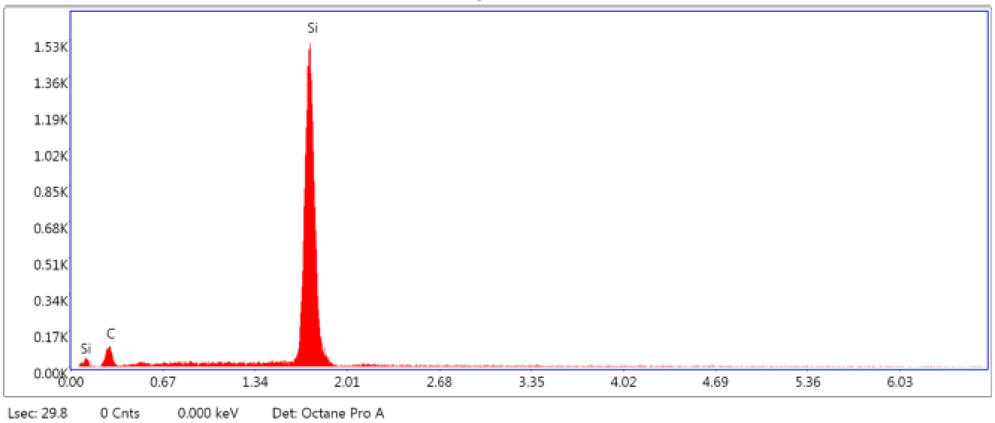

eZAF Smart Quant Results

Element	Weight %	Atomic %	Net Int.	Error %	Kratio	Z	A	F
C K	20.91	38.20	27.69	16.52	0.0319	1.1419	0.1337	1.0000
SiK	79.09	61.80	836.08	2.80	0.7574	0.9605	0.9969	1.0002

EDS Spot 5

kV: 10	Mag: 1612	Takeoff: 33.9	Live Time(s): 29.9	Amp Time(µs): 1.92	Resolution:(eV) 127.4

EDS Spot 5 - Det 1

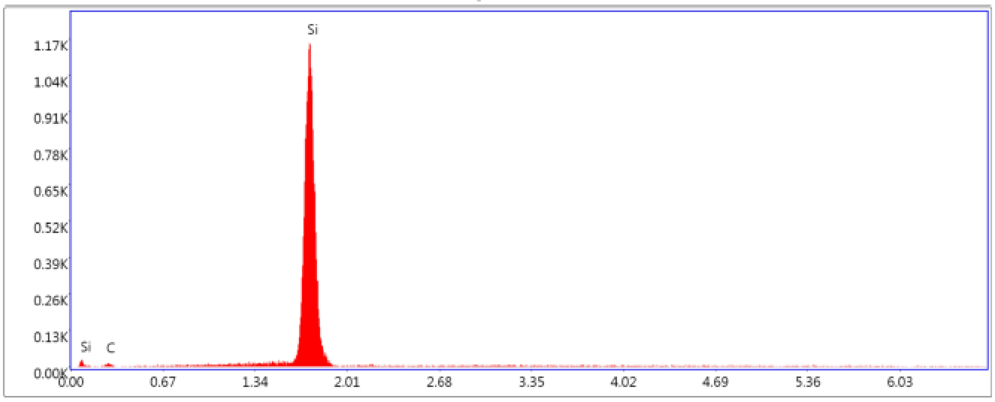

Lsec: 29.9 0 Cnts 0.000 keV Det: Octane Pro A

eZAF Smart Quant Results

Element	Weight %	Atomic %	Net Int.	Error %	Kratio	Z	A	F
C K	2.54	5.74	1.71	78.55	0.0032	1.1810	0.1084	1.0000
SiK	97.46	94.26	650.30	2.84	0.9695	0.9951	0.9996	1.0000

EDS Spot 6

kV: 10	Mag: 1612	Takeoff: 33.9	Live Time(s): 29.8	Amp Time(µs): 1.92	Resolution:(eV) 127.4

EDS Spot 6 - Det 1

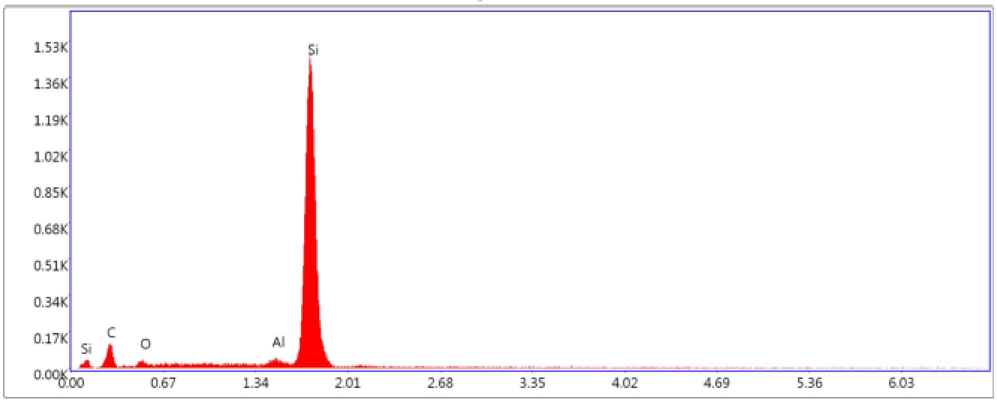

Lsec: 29.8 0 Cnts 0.000 keV Det: Octane Pro A

eZAF Smart Quant Results

Element	Weight %	Atomic %	Net Int.	Error %	Kratio	Z	A	F
C K	23.30	41.29	34.65	15.40	0.0371	1.1362	0.1400	1.0000
O K	0.94	1.25	5.82	45.33	0.0039	1.0750	0.3801	1.0000
AlK	1.85	1.46	22.26	12.60	0.0171	0.9378	0.9730	1.0153
SiK	73.91	56.00	829.40	2.90	0.6973	0.9554	0.9874	1.0002

EDS Spot 7

kV: 10	Mag: 1612	Takeoff: 33.9	Live Time(s): 29.8	Amp Time(µs): 1.92	Resolution:(eV) 127.4

EDS Spot 7 - Det 1

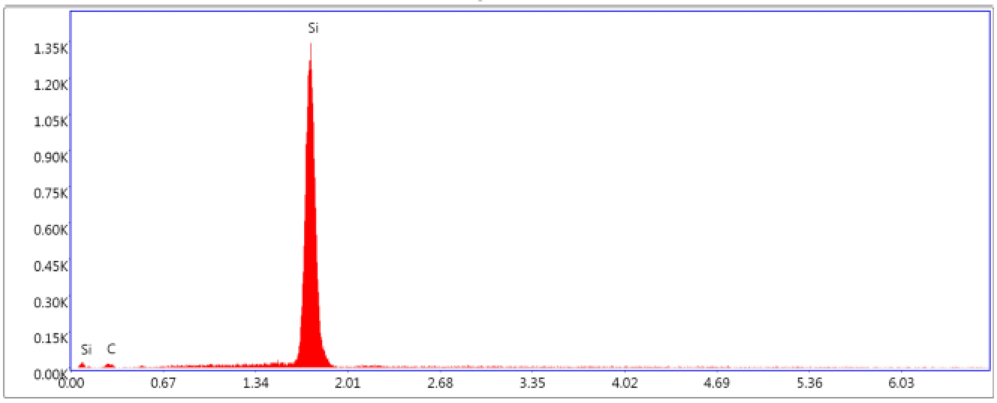

Lsec: 29.8 0 Cnts 0.000 keV Det: Octane Pro A

eZAF Smart Quant Results

Element	Weight %	Atomic %	Net Int.	Error %	Kratio	Z	A	F
C K	3.48	7.78	2.71	54.15	0.0045	1.1789	0.1095	1.0000
SiK	96.52	92.22	736.15	2.81	0.9582	0.9932	0.9995	1.0000

EDS Spot 8

kV: 10	Mag: 1612	Takeoff: 33.9	Live Time(s): 29.9	Amp Time(µs): 1.92	Resolution:(eV) 127.4

EDS Spot 8 - Det 1

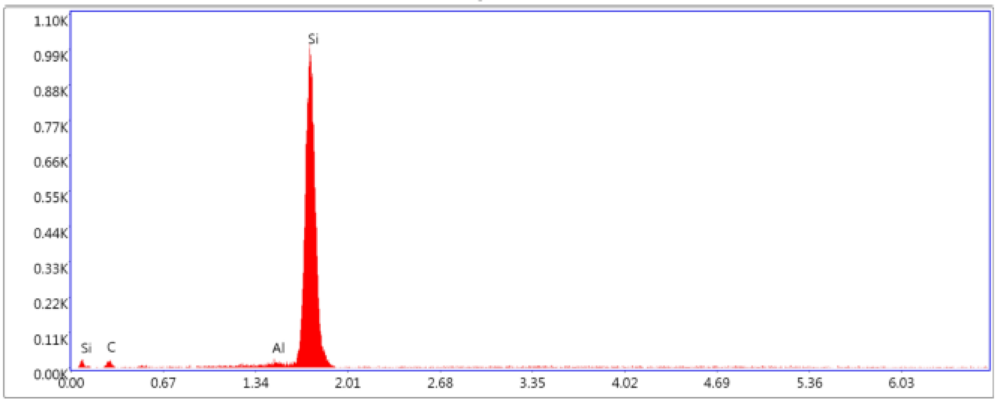

Lsec: 29.9 0 Cnts 0.000 keV Det: Octane Pro A

eZAF Smart Quant Results

Element	Weight %	Atomic %	Net Int.	Error %	Kratio	Z	A	F
C K	8.39	17.62	6.29	24.08	0.0114	1.1687	0.1158	1.0000
AlK	2.12	1.99	15.71	10.39	0.0204	0.9660	0.9787	1.0158
SiK	89.49	80.40	614.13	2.95	0.8715	0.9842	0.9894	1.0001

EDS Spot 9

kV: 10	Mag: 1612	Takeoff: 33.9	Live Time(s): 29.7	Amp Time(µs): 1.92	Resolution:(eV) 127.4

EDS Spot 9 - Det 1

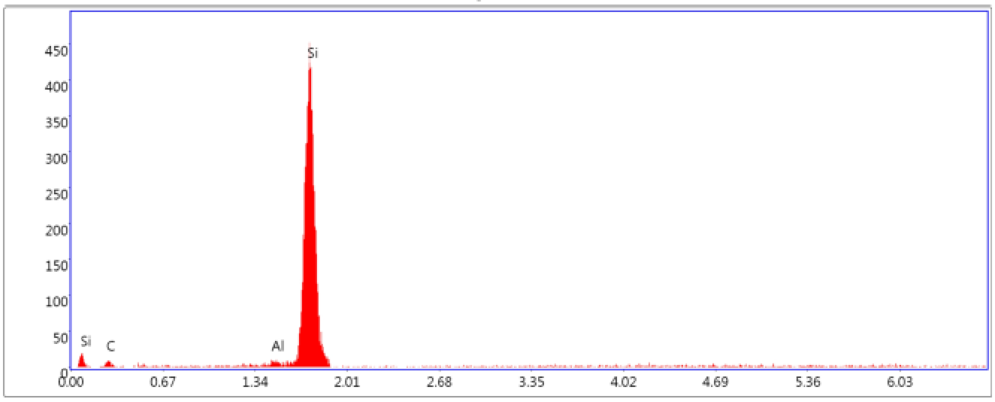

Lsec: 29.7 0 Cnts 0.000 keV Det: Octane Pro A

eZAF Smart Quant Results

Element	Weight %	Atomic %	Net Int.	Error %	Kratio	Z	A	F
C K	9.29	19.30	2.73	28.10	0.0127	1.1668	0.1170	1.0000
AlK	1.92	1.78	5.52	16.47	0.0184	0.9643	0.9784	1.0158
SiK	88.79	78.92	236.78	3.43	0.8637	0.9825	0.9902	1.0001

EDS Spot 10

kV: 10	Mag: 1612	Takeoff: 33.9	Live Time(s): 29.8	Amp Time(µs): 1.92	Resolution:(eV) 127.4

EDS Spot 10 - Det 1

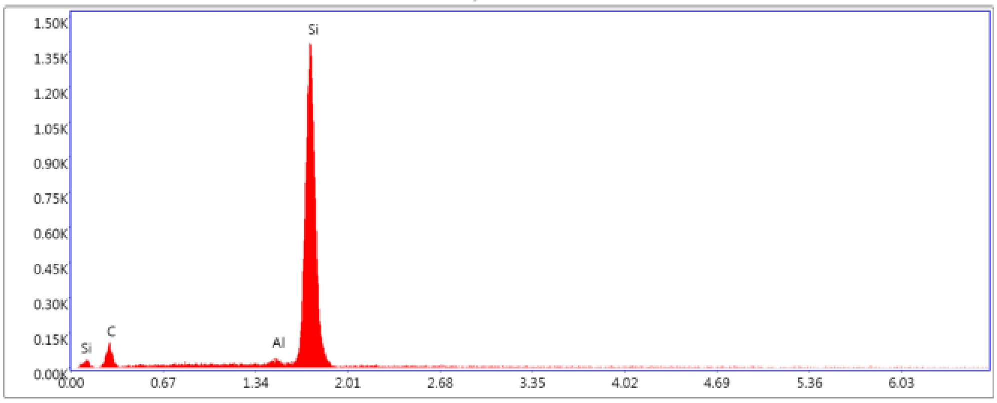

Lsec: 29.8 0 Cnts 0.000 keV Det: Octane Pro A

eZAF Smart Quant Results

Element	Weight %	Atomic %	Net Int.	Error %	Kratio	Z	A	F
C K	23.01	41.11	30.74	15.60	0.0361	1.1379	0.1378	1.0000
AlK	1.51	1.20	16.66	15.48	0.0141	0.9393	0.9746	1.0156
SiK	75.48	57.69	775.33	2.92	0.7151	0.9570	0.9699	1.0002

EDS Spot 11

kV: 10	Mag: 1612	Takeoff: 33.9	Live Time(s): 29.9	Amp Time(μs): 1.92	Resolution:(eV) 127.4

EDS Spot 11 - Det 1

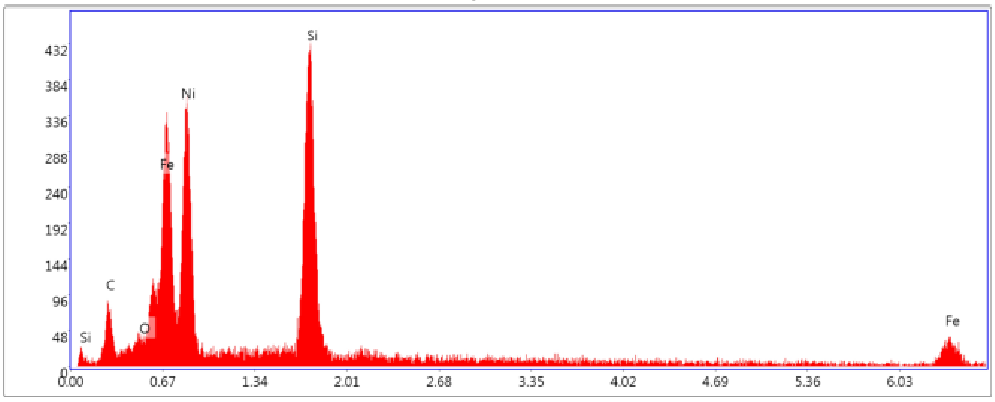

Lsec: 29.9   0 Cnts   0.000 keV   Det: Octane Pro A

eZAF Smart Quant Results

Element	Weight %	Atomic %	Net Int.	Error %	Kratio	Z	A	F
C K	11.43	30.84	24.46	16.77	0.0317	1.2696	0.2183	1.0000
O K	1.59	3.21	12.12	22.97	0.0097	1.2052	0.5084	1.0000
FeL	24.08	13.97	94.04	4.67	0.2060	0.8994	0.9511	1.0000
NiL	34.28	18.93	121.18	6.56	0.2363	0.9109	0.7568	1.0000
SiK	28.63	33.05	245.98	5.70	0.2502	1.0789	0.8089	1.0014

EDS Spot 12

kV: 10	Mag: 1612	Takeoff: 33.9	Live Time(s): 29.9	Amp Time(μs): 1.92	Resolution:(eV) 127.4

EDS Spot 12 - Det 1

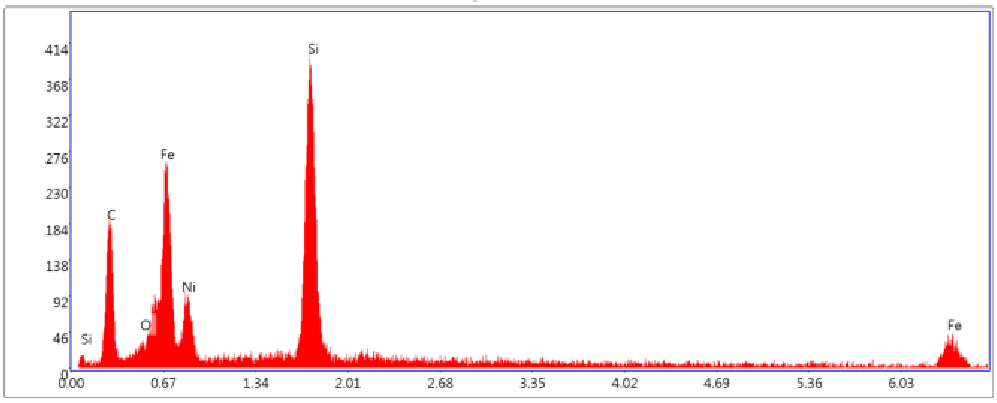

Lsec: 29.9   0 Cnts   0.000 keV   Det: Octane Pro A

eZAF Smart Quant Results

Element	Weight %	Atomic %	Net Int.	Error %	Kratio	Z	A	F
C K	33.68	61.27	72.39	12.24	0.1033	1.1747	0.2610	1.0000
O K	1.99	2.72	10.54	24.33	0.0093	1.1132	0.4191	1.0000
FeL	26.81	10.49	78.51	5.75	0.1895	0.8302	0.8513	1.0000
NiL	9.02	3.36	25.82	12.60	0.0555	0.8403	0.7324	1.0000
SiK	28.49	22.16	222.75	5.02	0.2497	0.9930	0.8813	1.0016

EDS Spot 13

kV: 10	Mag: 1612	Takeoff: 33.9	Live Time(s): 29.8	Amp Time(µs): 1.92	Resolution:(eV) 127.4

EDS Spot 13 - Det 1

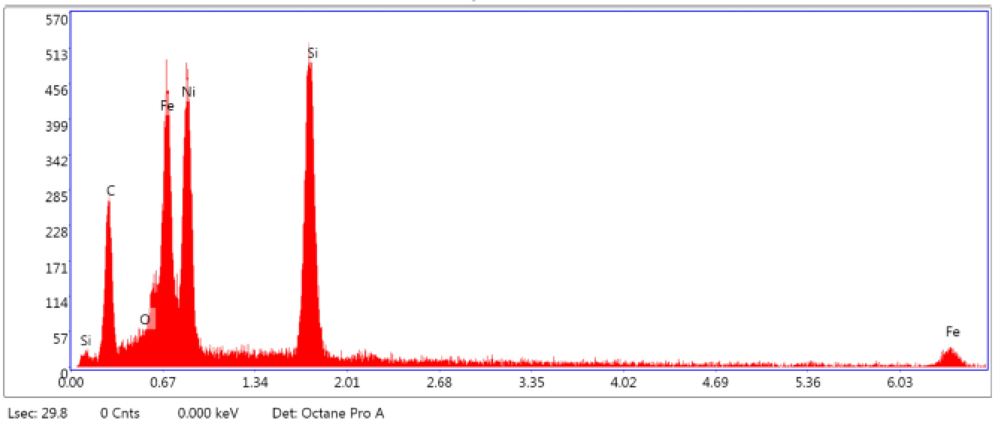

eZAF Smart Quant Results

Element	Weight %	Atomic %	Net Int.	Error %	Kratio	Z	A	F
C K	25.74	54.88	99.88	11.84	0.0832	1.2198	0.2651	1.0000
O K	1.72	2.75	17.66	18.80	0.0091	1.1573	0.4581	1.0000
FeL	21.76	9.98	120.42	4.93	0.1698	0.8634	0.9039	1.0000
NiL	29.22	12.75	156.20	6.16	0.1961	0.8743	0.7676	1.0000
SiK	21.56	19.65	282.65	5.39	0.1851	1.0346	0.8289	1.0016

Area 2

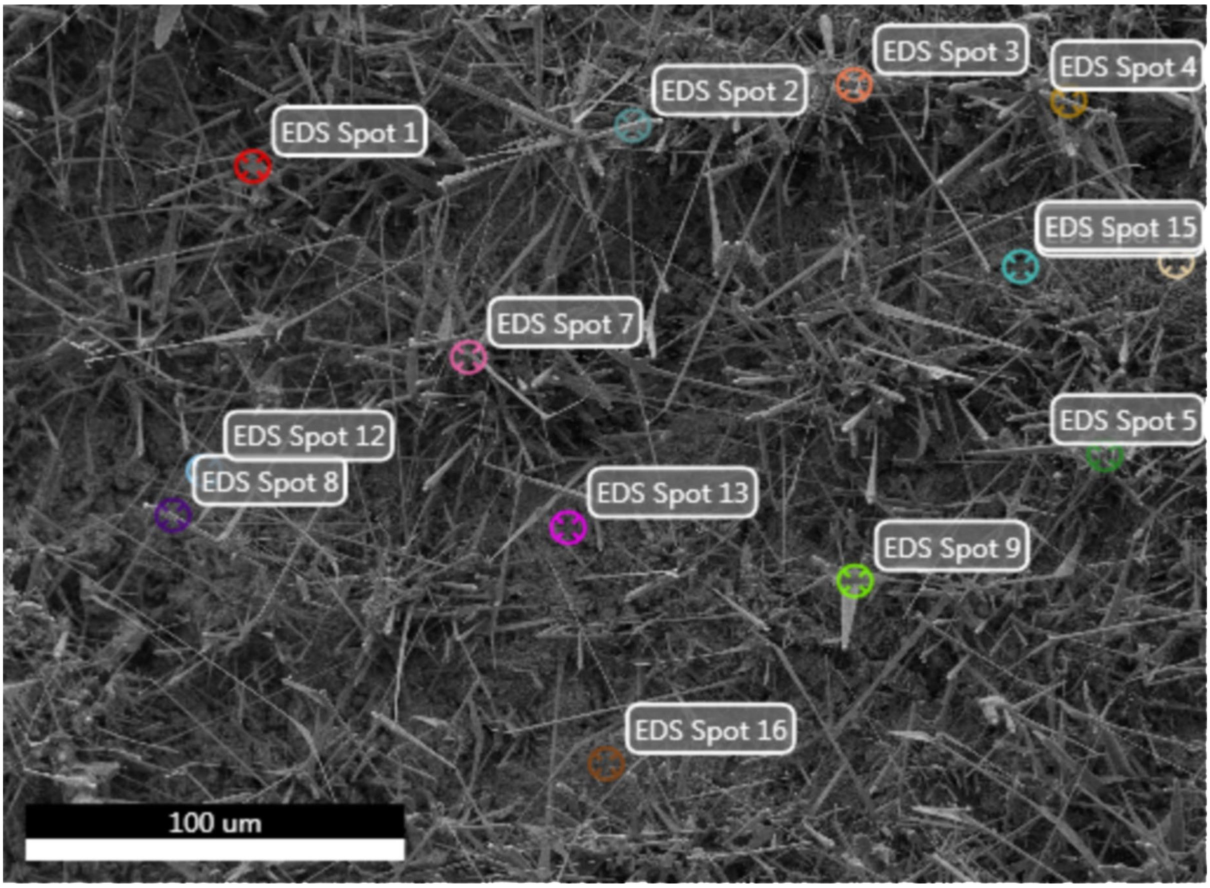
EDS Spot 1
EDS Spot 2
EDS Spot 3
EDS Spot 4
EDS Spot 15
EDS Spot 7
EDS Spot 5
EDS Spot 12
EDS Spot 8
EDS Spot 13
EDS Spot 9
EDS Spot 16
100 um

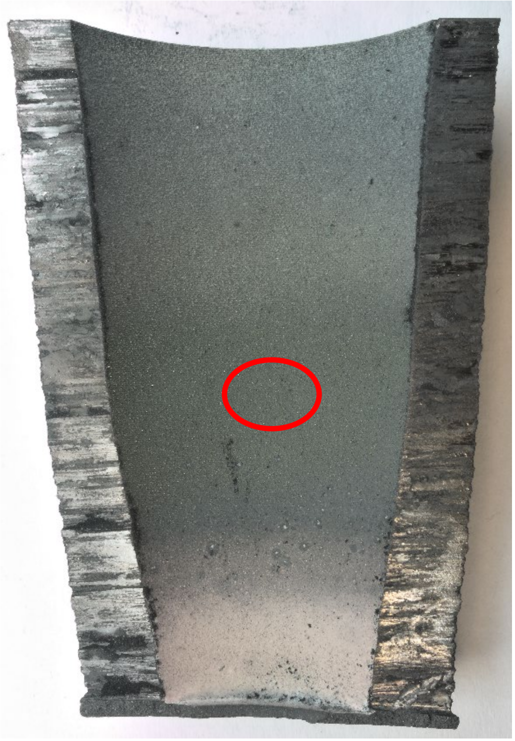

EDS Spot 1

kV: 10	Mag: 346	Takeoff: 34.3	Live Time(s): 29.8	Amp Time(μs): 1.92	Resolution:(eV) 127.4

EDS Spot 1 - Det 1

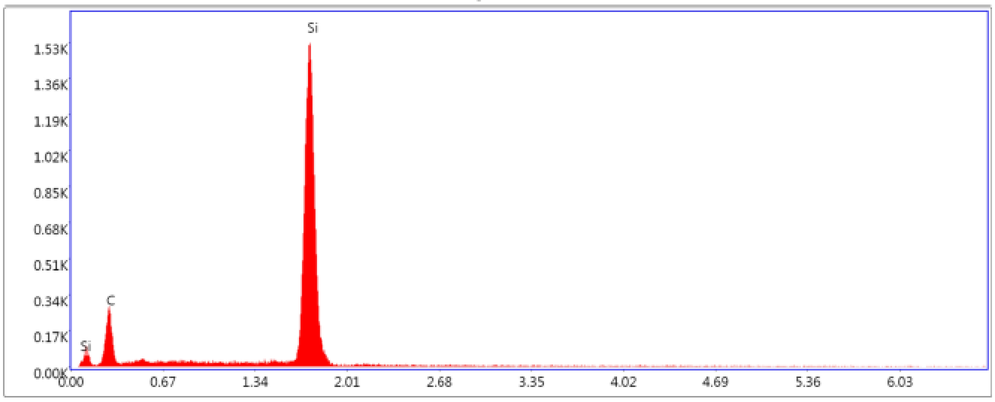

Lsec: 29.8 0 Cnts 0.000 keV Det: Octane Pro A

eZAF Smart Quant Results

Element	Weight %	Atomic %	Net Int.	Error %	Kratio	Z	A	F
C K	40.16	61.08	96.45	12.52	0.0786	1.1037	0.1773	1.0000
SiK	59.84	38.92	858.04	2.83	0.5516	0.9266	0.9942	1.0004

EDS Spot 2

kV: 10	Mag: 346	Takeoff: 34.3	Live Time(s): 29.8	Amp Time(μs): 1.92	Resolution:(eV) 127.4

EDS Spot 2 - Det 1

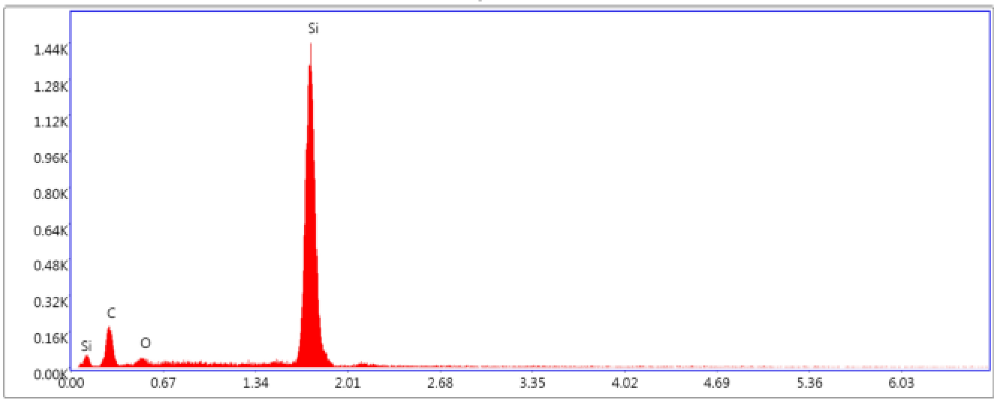

Lsec: 29.8 0 Cnts 0.000 keV Det: Octane Pro A

eZAF Smart Quant Results

Element	Weight %	Atomic %	Net Int.	Error %	Kratio	Z	A	F
C K	34.73	54.94	66.79	13.27	0.0645	1.1119	0.1670	1.0000
O K	1.78	2.12	11.33	24.47	0.0068	1.0515	0.3619	1.0000
SiK	63.48	42.94	773.32	2.87	0.5888	0.9339	0.9931	1.0003

EDS Spot 3

kV: 10 Mag: 346 Takeoff: 34.3 Live Time(s): 29.8 Amp Time(µs): 1.92 Resolution:(eV) 127.4

EDS Spot 3 - Det 1

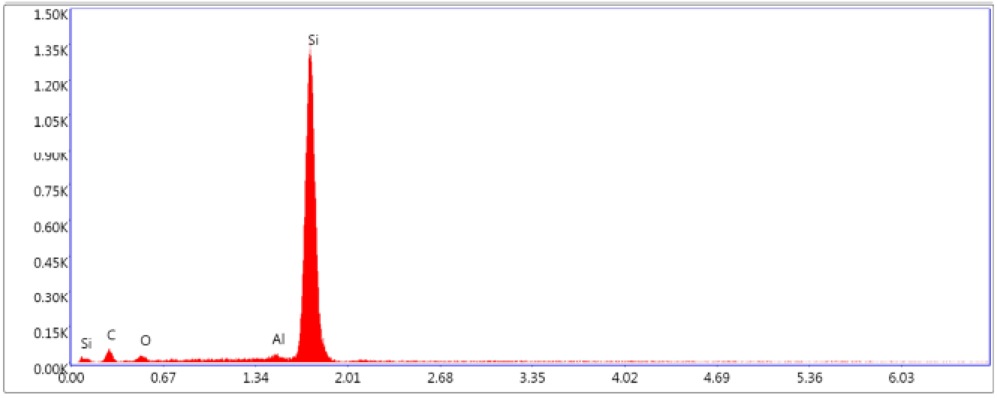

Lsec: 29.8 0 Cnts 0.000 keV Det: Octane Pro A

eZAF Smart Quant Results

Element	Weight %	Atomic %	Net Int.	Error %	Kratio	Z	A	F
C K	13.84	27.04	15.73	18.65	0.0202	1.1553	0.1260	1.0000
O K	1.42	2.08	7.88	25.28	0.0063	1.0934	0.4033	1.0000
AlK	2.30	2.00	23.47	10.81	0.0217	0.9543	0.9751	1.0154
SiK	82.44	68.88	782.37	2.93	0.7908	0.9723	0.9865	1.0001

EDS Spot 4

kV: 10 Mag: 346 Takeoff: 34.3 Live Time(s): 29.8 Amp Time(µs): 1.92 Resolution:(eV) 127.4

EDS Spot 4 - Det 1

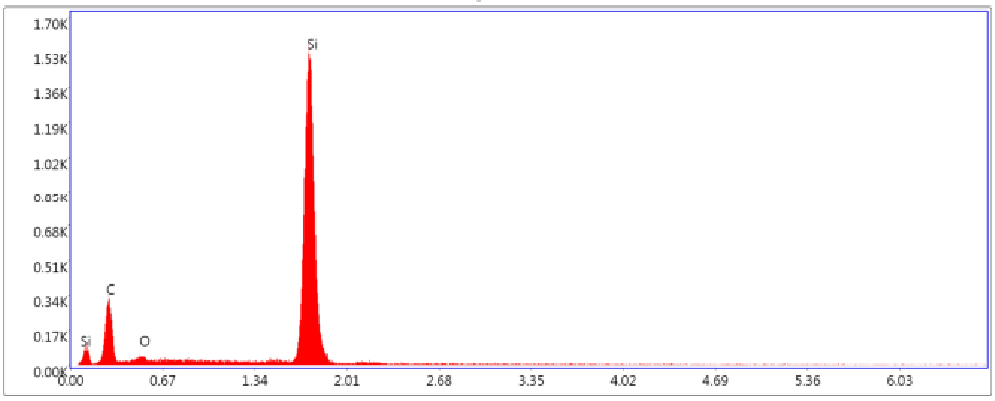

Lsec: 29.8 0 Cnts 0.000 keV Det: Octane Pro A

eZAF Smart Quant Results

Element	Weight %	Atomic %	Net Int.	Error %	Kratio	Z	A	F
C K	43.08	63.51	118.96	11.99	0.0896	1.0966	0.1896	1.0000
O K	1.24	1.37	9.54	30.14	0.0045	1.0367	0.3461	1.0000
SiK	55.68	35.11	856.68	2.87	0.5090	0.9203	0.9925	1.0005

EDS Spot 5

kV: 10	Mag: 346	Takeoff: 34.3	Live Time(s): 29.8	Amp Time(µs): 1.92	Resolution:(eV) 127.4

EDS Spot 5 - Det 1

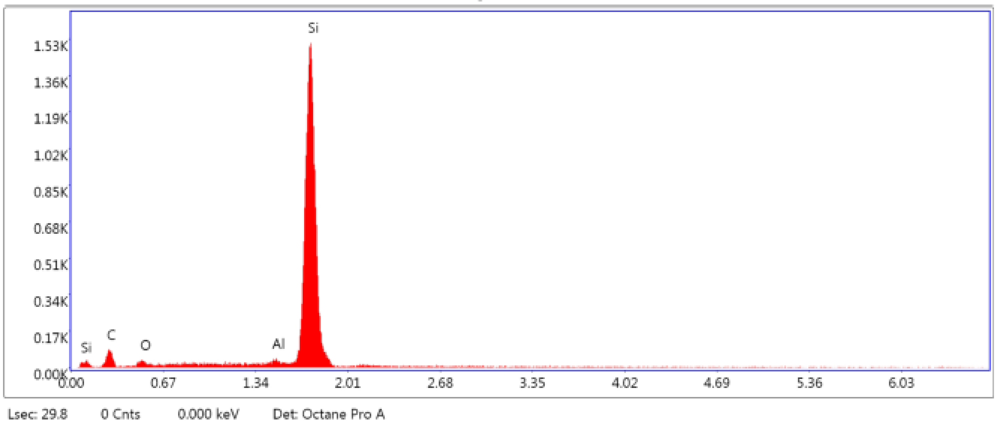

eZAF Smart Quant Results

Element	Weight %	Atomic %	Net Int.	Error %	Kratio	Z	A	F
C K	17.71	33.29	22.89	17.75	0.0266	1.1478	0.1310	1.0000
O K	0.83	1.17	4.91	54.46	0.0035	1.0862	0.3933	1.0000
AlK	1.42	1.19	15.90	15.32	0.0133	0.9479	0.9749	1.0157
SiK	80.04	64.35	834.87	2.87	0.7657	0.9657	0.9903	1.0001

EDS Spot 7

kV: 10	Mag: 346	Takeoff: 34.3	Live Time(s): 29.8	Amp Time(µs): 1.92	Resolution:(eV) 127.4

EDS Spot 7 - Det 1

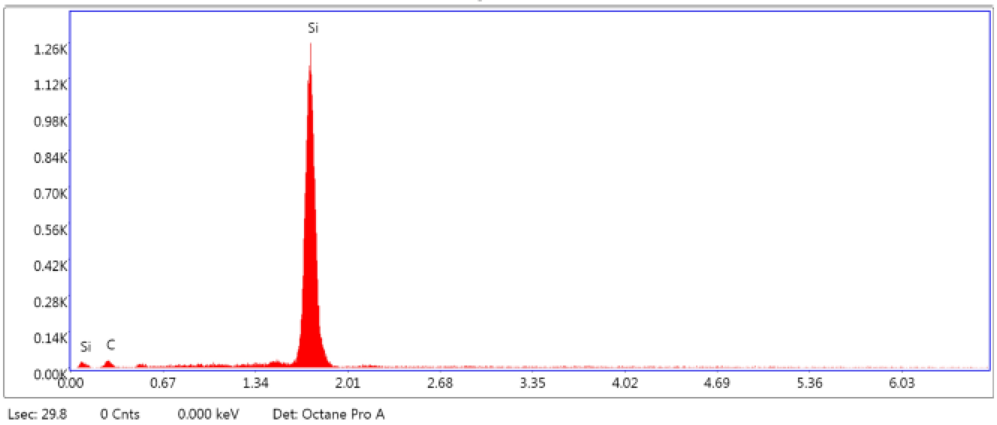

eZAF Smart Quant Results

Element	Weight %	Atomic %	Net Int.	Error %	Kratio	Z	A	F
C K	8.69	18.20	6.97	25.67	0.0118	1.1676	0.1166	1.0000
SiK	91.31	81.80	669.90	2.85	0.8967	0.9833	0.9987	1.0001

EDS Spot 8

kV: 10	Mag: 346	Takeoff: 34.3	Live Time(s): 29.8	Amp Time(µs): 1.92	Resolution:(eV) 127.4

EDS Spot 8 - Det 1

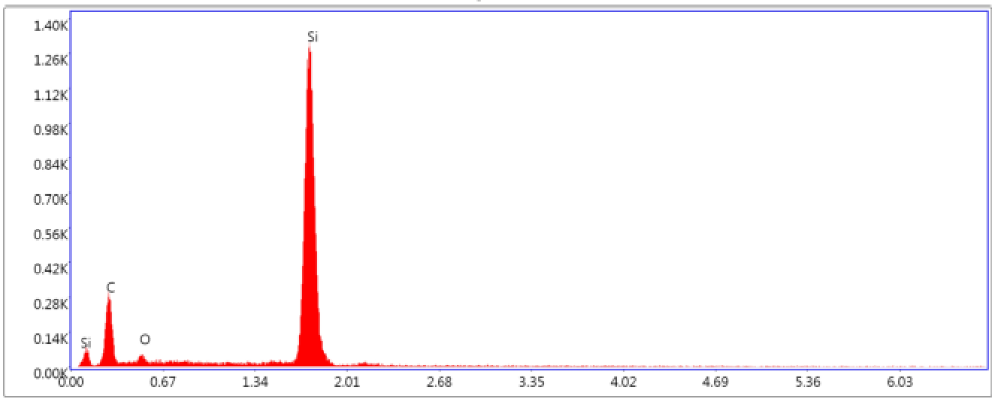

Lsec: 29.8 0 Cnts 0.000 keV Det: Octane Pro A

eZAF Smart Quant Results

Element	Weight %	Atomic %	Net Int.	Error %	Kratio	Z	A	F
C K	43.52	63.62	107.88	12.02	0.0925	1.0944	0.1943	1.0000
O K	2.25	2.47	15.22	20.20	0.0081	1.0346	0.3473	1.0000
SiK	54.23	33.90	730.03	2.94	0.4938	0.9183	0.9914	1.0005

EDS Spot 9

kV: 10	Mag: 346	Takeoff: 34.3	Live Time(s): 29.9	Amp Time(µs): 1.92	Resolution:(eV) 127.4

EDS Spot 9 - Det 1

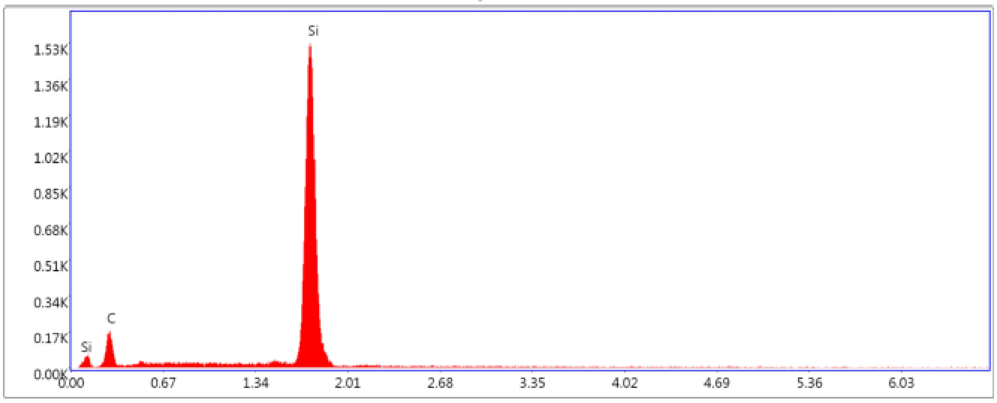

Lsec: 29.9 0 Cnts 0.000 keV Det: Octane Pro A

eZAF Smart Quant Results

Element	Weight %	Atomic %	Net Int.	Error %	Kratio	Z	A	F
C K	31.24	51.51	56.70	13.61	0.0542	1.1211	0.1547	1.0000
SiK	68.76	48.49	855.70	2.81	0.6452	0.9421	0.9954	1.0003

EDS Spot 12

kV: 10	Mag: 346	Takeoff: 34.3	Live Time(s): 29.8	Amp Time(μs): 1.92	Resolution:(eV) 127.4

EDS Spot 12 - Det 1

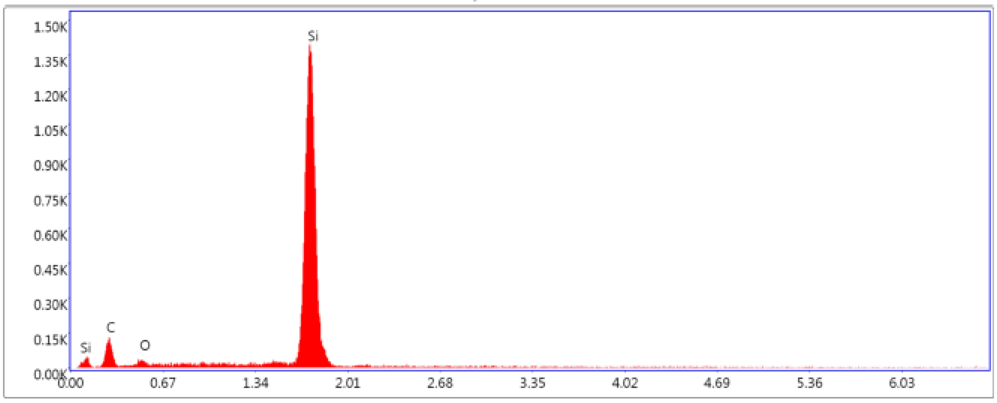

Lsec: 29.8 0 Cnts 0.000 keV Det: Octane Pro A

eZAF Smart Quant Results

Element	Weight %	Atomic %	Net Int.	Error %	Kratio	Z	A	F
C K	26.51	45.51	39.98	15.04	0.0439	1.1293	0.1465	1.0000
O K	0.94	1.21	5.56	45.40	0.0038	1.0684	0.3755	1.0000
SiK	72.55	53.27	792.16	2.84	0.6856	0.9494	0.9951	1.0002

EDS Spot 13

kV: 10	Mag: 346	Takeoff: 34.3	Live Time(s): 29.8	Amp Time(μs): 1.92	Resolution:(eV) 127.4

EDS Spot 13 - Det 1

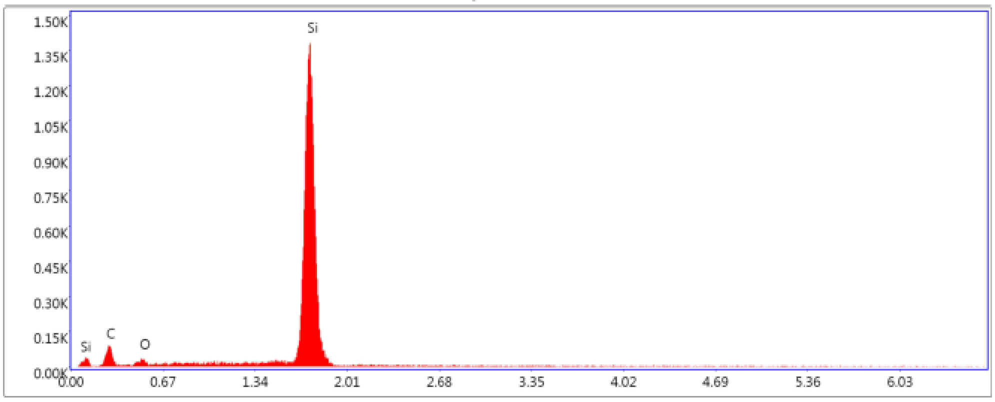

Lsec: 29.8 0 Cnts 0.000 keV Det: Octane Pro A

eZAF Smart Quant Results

Element	Weight %	Atomic %	Net Int.	Error %	Kratio	Z	A	F
C K	20.73	37.78	25.75	16.67	0.0321	1.1413	0.1355	1.0000
O K	0.73	1.00	3.94	69.76	0.0030	1.0800	0.3862	1.0000
SiK	78.54	61.22	765.03	2.84	0.7512	0.9600	0.9962	1.0002

EDS Spot 14

kV: 10	Mag: 346	Takeoff: 34.3	Live Time(s): 29.9	Amp Time(µs): 1.92	Resolution:(eV) 127.4

EDS Spot 14 - Det 1

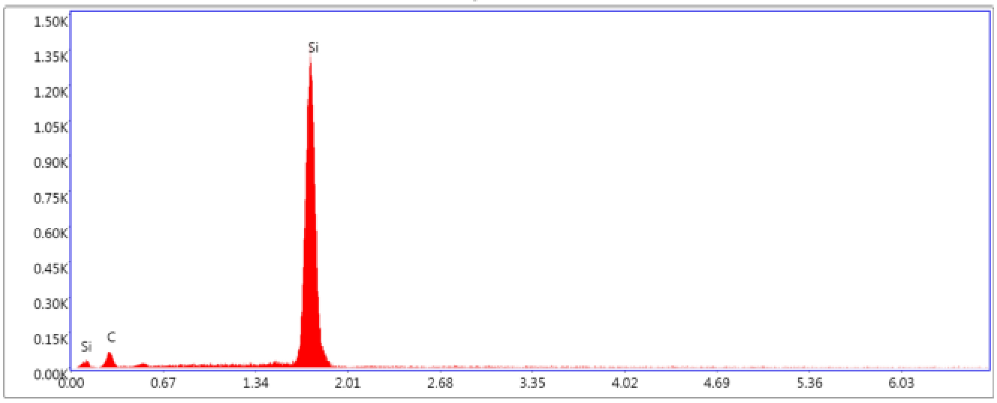

Lsec: 29.9 0 Cnts 0.000 keV Det: Octane Pro A

eZAF Smart Quant Results

Element	Weight %	Atomic %	Net Int.	Error %	Kratio	Z	A	F
C K	18.42	34.55	20.35	17.91	0.0276	1.1471	0.1305	1.0000
SiK	81.58	65.45	734.40	2.84	0.7853	0.9651	0.9973	1.0001

EDS Spot 15

kV: 10	Mag: 346	Takeoff: 34.3	Live Time(s): 29.8	Amp Time(µs): 1.92	Resolution:(eV) 127.4

EDS Spot 15 - Det 1

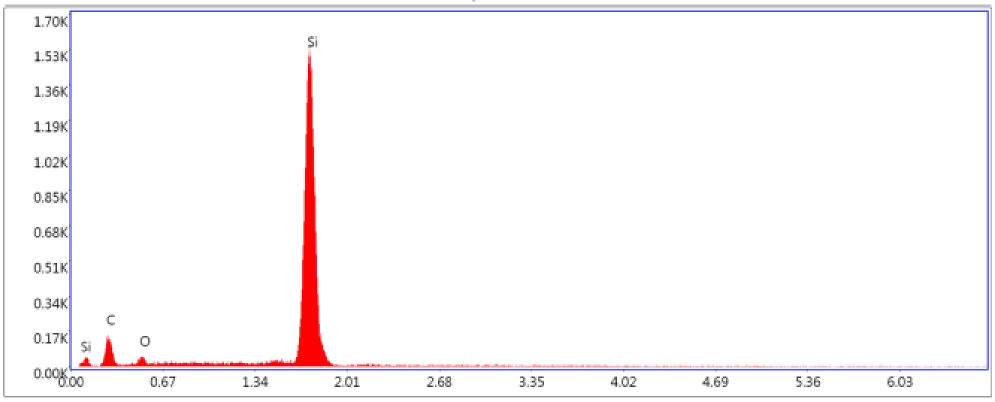

Lsec: 29.8 0 Cnts 0.000 keV Det: Octane Pro A

eZAF Smart Quant Results

Element	Weight %	Atomic %	Net Int.	Error %	Kratio	Z	A	F
C K	27.50	46.65	46.83	14.30	0.0463	1.1268	0.1494	1.0000
O K	1.39	1.77	9.04	25.41	0.0055	1.0659	0.3746	1.0000
SiK	71.11	51.58	859.24	2.82	0.6700	0.9471	0.9945	1.0002

EDS Spot 16

kV: 10	Mag: 346	Takeoff: 34.3	Live Time(s): 29.8	Amp Time(µs): 1.92	Resolution:(eV) 127.4

EDS Spot 16 - Det 1

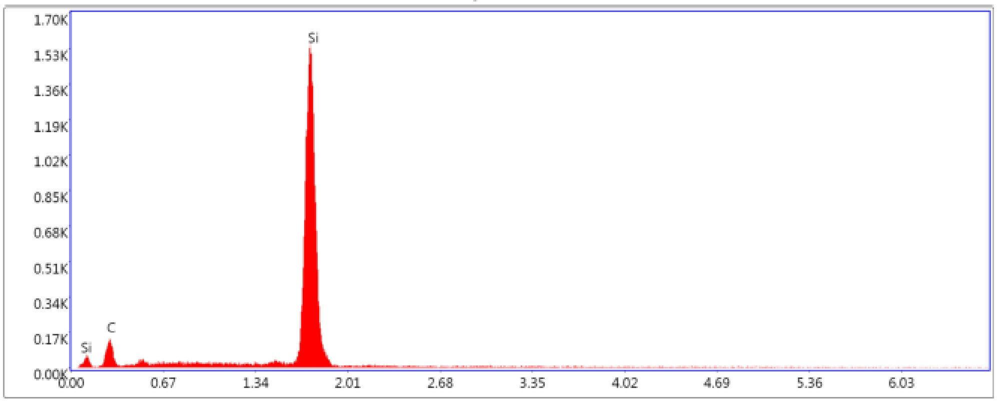

Lsec: 29.8 0 Cnts 0.000 keV Det: Octane Pro A

eZAF Smart Quant Results

Element	Weight %	Atomic %	Net Int.	Error %	Kratio	Z	A	F
C K	26.72	46.02	43.10	14.93	0.0439	1.1301	0.1453	1.0000
SiK	73.28	53.98	864.16	2.80	0.6938	0.9501	0.9961	1.0002

Area 3

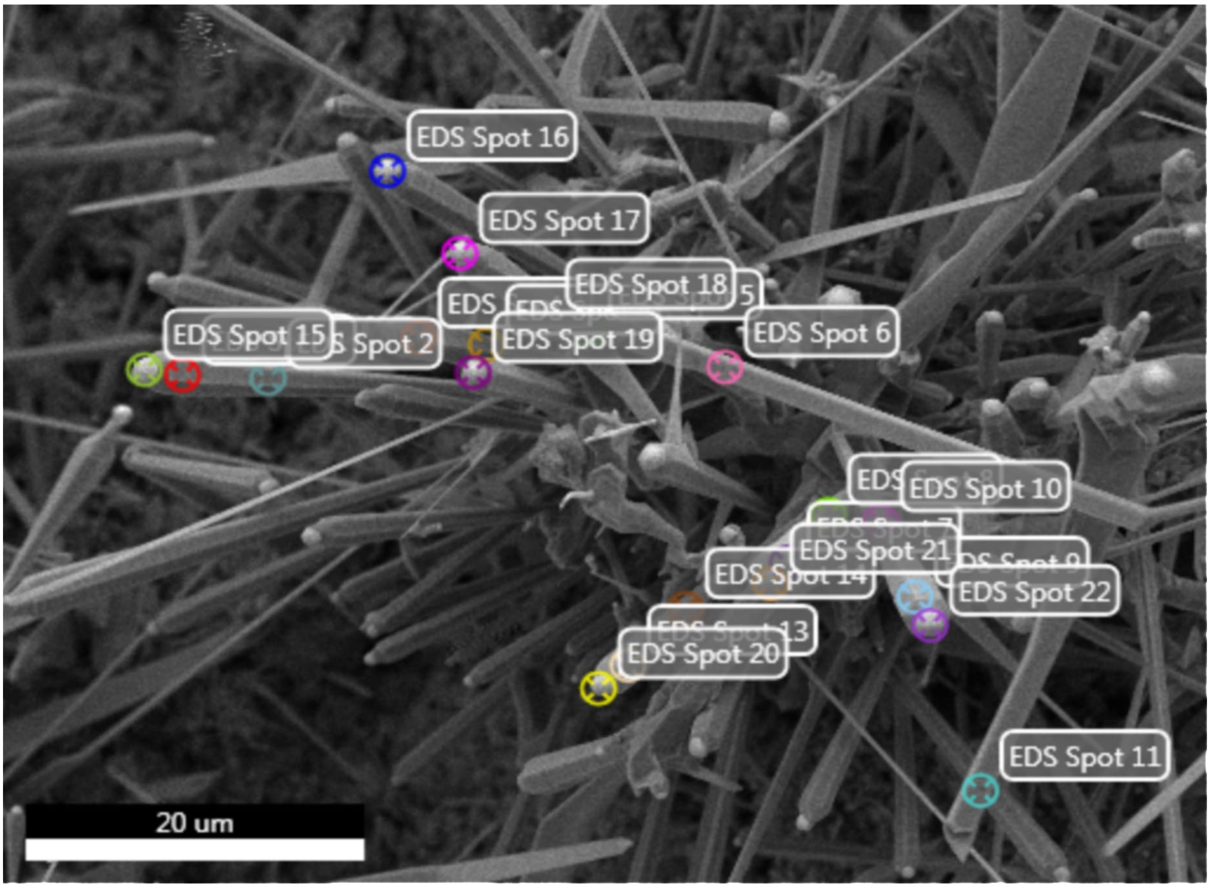
EDS Spot 16
EDS Spot 17
EDS Spot 18
EDS Spot 15
EDS Spot 19
EDS Spot 6
EDS Spot 10
EDS Spot 21
EDS Spot 22
EDS Spot 20
EDS Spot 11
20 um

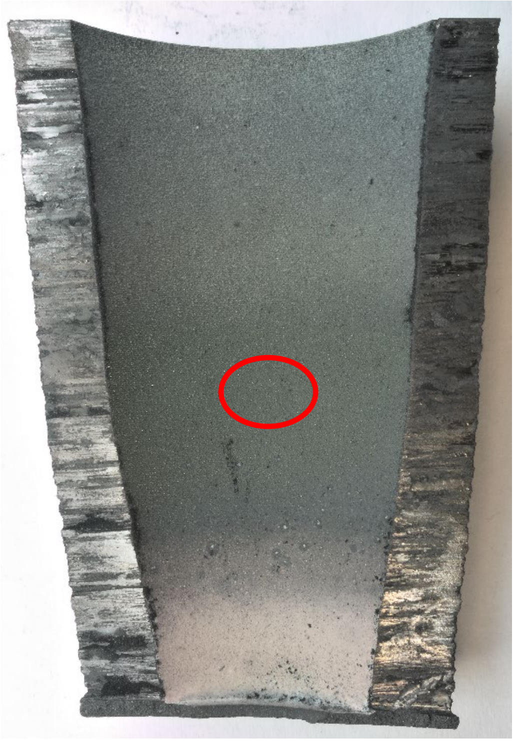

EDS Spot 1

kV: 10	Mag: 1546	Takeoff: 34	Live Time(s): 29.8	Amp Time(µs): 1.92	Resolution:(eV) 127.4

EDS Spot 1 - Det 1

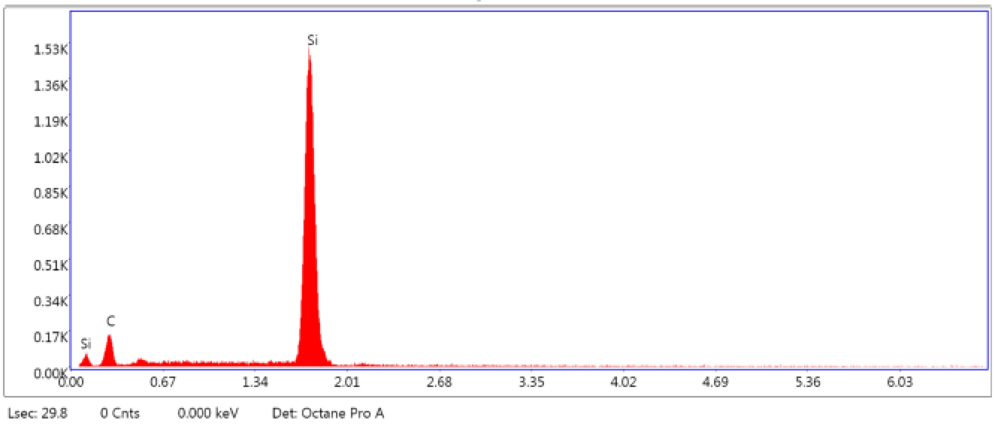

eZAF Smart Quant Results

Element	Weight %	Atomic %	Net Int.	Error %	Kratio	Z	A	F
C K	29.99	50.04	51.36	14.19	0.0509	1.1236	0.1510	1.0000
SiK	70.01	49.96	844.95	2.84	0.6585	0.9443	0.9956	1.0003

EDS Spot 2

kV: 10	Mag: 1546	Takeoff: 34	Live Time(s): 29.8	Amp Time(µs): 1.92	Resolution:(eV) 127.4

EDS Spot 2 - Det 1

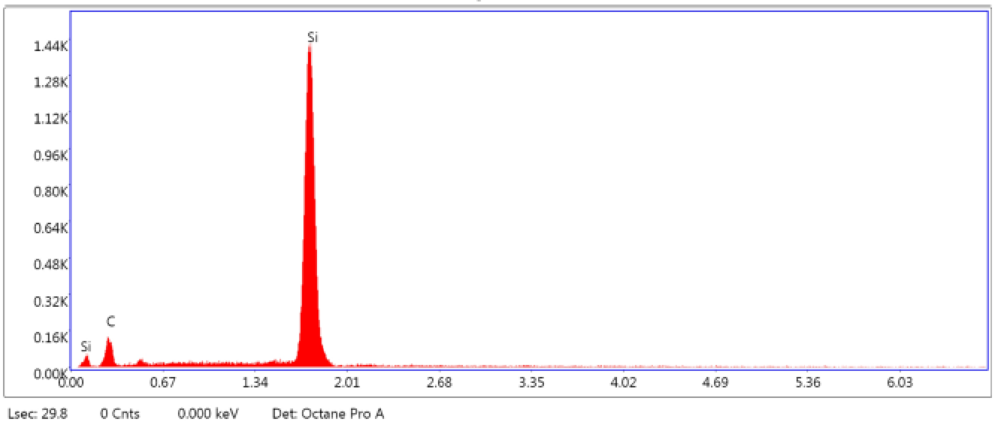

eZAF Smart Quant Results

Element	Weight %	Atomic %	Net Int.	Error %	Kratio	Z	A	F
C K	27.29	46.74	41.62	14.95	0.0448	1.1290	0.1455	1.0000
SiK	72.71	53.26	811.83	2.82	0.6875	0.9491	0.9960	1.0002

EDS Spot 3

kV: 10	Mag: 1546	Takeoff: 34	Live Time(s): 29.8	Amp Time(μs): 1.92	Resolution:(eV) 127.4

EDS Spot 3 - Det 1

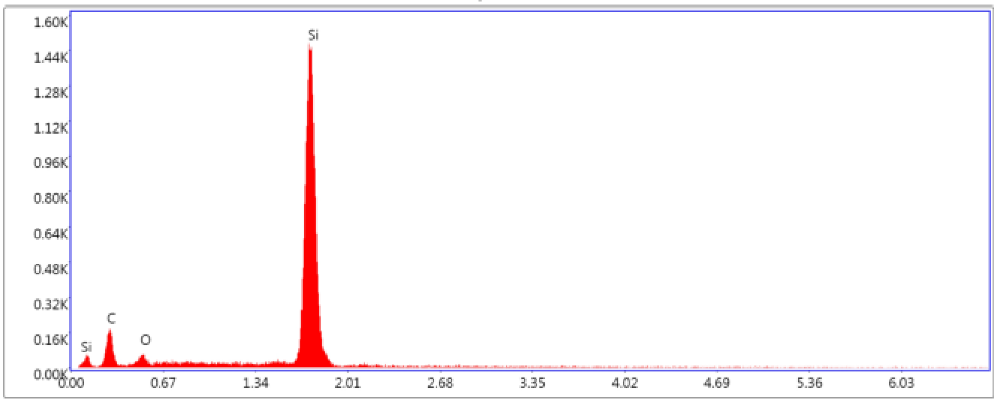

Lsec: 29.8 0 Cnts 0.000 keV Det: Octane Pro A

eZAF Smart Quant Results

Element	Weight %	Atomic %	Net Int.	Error %	Kratio	Z	A	F
C K	30.93	50.59	56.66	13.60	0.0544	1.1191	0.1572	1.0000
O K	2.06	2.53	13.51	20.38	0.0080	1.0585	0.3676	1.0000
SiK	67.01	46.87	828.87	2.87	0.6261	0.9403	0.9933	1.0003

EDS Spot 4

kV: 10	Mag: 1546	Takeoff: 34	Live Time(s): 29.9	Amp Time(μs): 1.92	Resolution:(eV) 127.4

EDS Spot 4 - Det 1

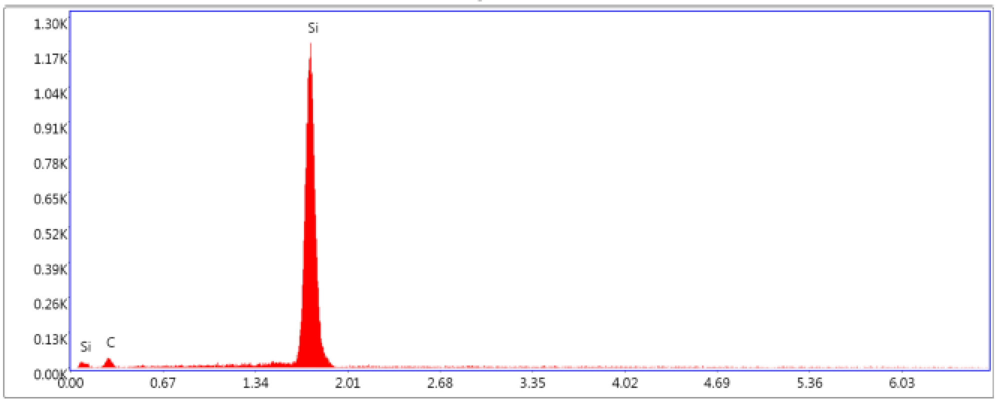

Lsec: 29.9 0 Cnts 0.000 keV Det: Octane Pro A

eZAF Smart Quant Results

Element	Weight %	Atomic %	Net Int.	Error %	Kratio	Z	A	F
C K	11.82	23.86	10.25	22.27	0.0165	1.1609	0.1200	1.0000
SiK	88.18	76.14	681.14	2.85	0.8604	0.9774	0.9982	1.0001

EDS Spot 5

kV: 10	Mag: 1546	Takeoff: 34	Live Time(s): 29.7	Amp Time(μs): 1.92	Resolution:(eV) 127.4

EDS Spot 5 - Det 1

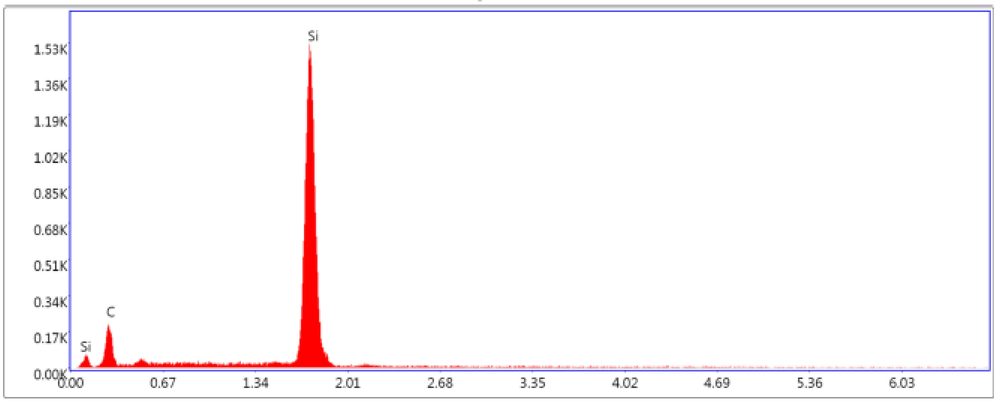

Lsec: 29.7 0 Cnts 0.000 keV Det: Octane Pro A

eZAF Smart Quant Results

Element	Weight %	Atomic %	Net Int.	Error %	Kratio	Z	A	F
C K	35.03	55.76	69.70	13.25	0.0634	1.1136	0.1627	1.0000
SiK	64.97	44.24	844.91	2.83	0.6047	0.9354	0.9948	1.0003

EDS Spot 6

kV: 10	Mag: 1546	Takeoff: 34	Live Time(s): 29.8	Amp Time(μs): 1.92	Resolution:(eV) 127.4

EDS Spot 6 - Det 1

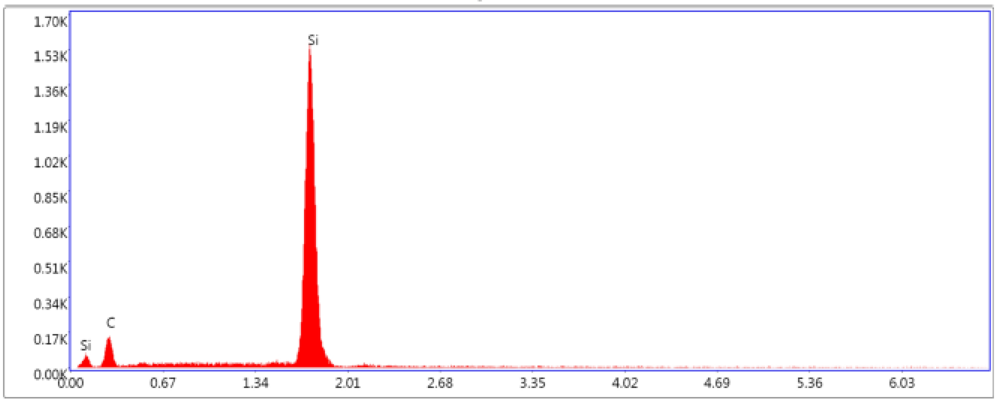

Lsec: 29.8 0 Cnts 0.000 keV Det: Octane Pro A

eZAF Smart Quant Results

Element	Weight %	Atomic %	Net Int.	Error %	Kratio	Z	A	F
C K	29.55	49.51	50.26	14.21	0.0499	1.1245	0.1501	1.0000
SiK	70.45	50.49	849.84	2.83	0.6632	0.9451	0.9956	1.0002

EDS Spot 7

kV: 10	Mag: 1546	Takeoff: 34	Live Time(s): 29.9	Amp Time(μs): 1.92	Resolution:(eV) 127.4

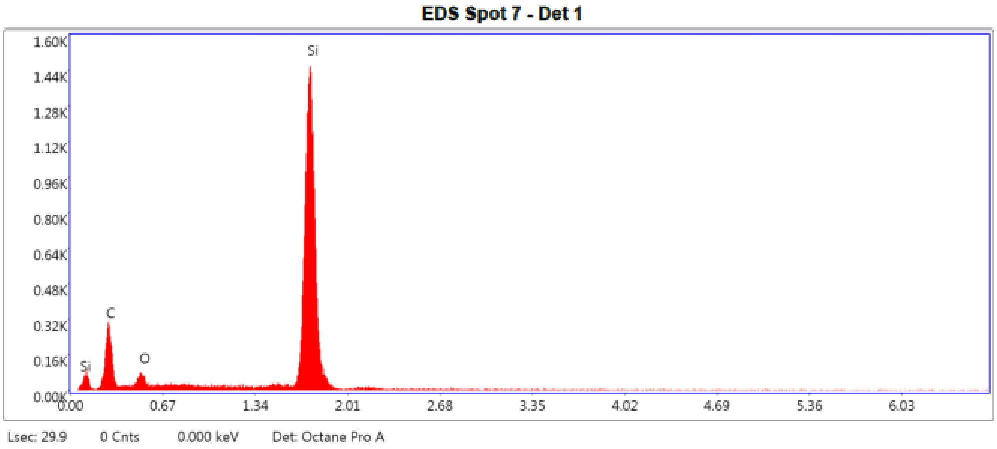

eZAF Smart Quant Results

Element	Weight %	Atomic %	Net Int.	Error %	Kratio	Z	A	F
C K	41.26	61.20	107.93	12.09	0.0853	1.0976	0.1883	1.0000
O K	3.21	3.58	23.93	16.48	0.0117	1.0377	0.3512	1.0000
SiK	55.53	35.23	816.07	2.90	0.5071	0.9212	0.9906	1.0005

EDS Spot 8

kV: 10	Mag: 1546	Takeoff: 34	Live Time(s): 29.8	Amp Time(μs): 1.92	Resolution:(eV) 127.4

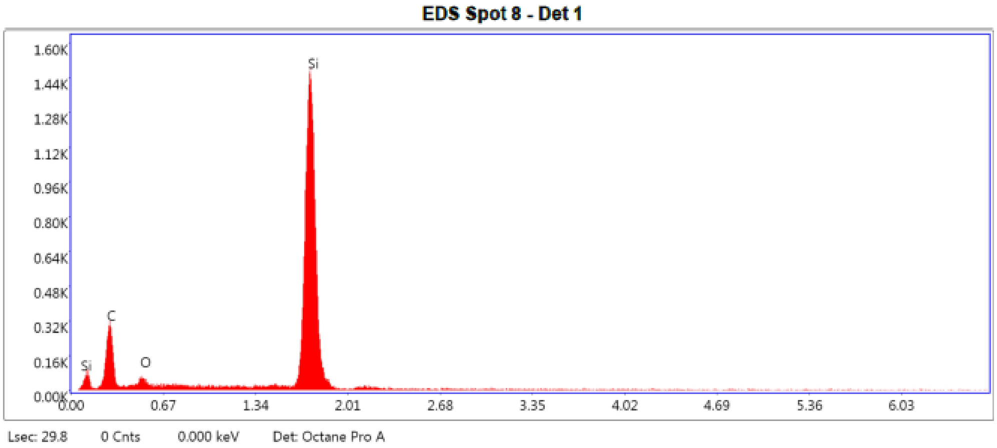

eZAF Smart Quant Results

Element	Weight %	Atomic %	Net Int.	Error %	Kratio	Z	A	F
C K	42.71	62.72	118.50	11.98	0.0897	1.0954	0.1917	1.0000
O K	2.75	3.04	21.16	16.83	0.0099	1.0355	0.3477	1.0000
SiK	54.53	34.25	834.92	2.89	0.4971	0.9192	0.9909	1.0005

EDS Spot 9

kV: 10	Mag: 1546	Takeoff: 34	Live Time(s): 29.9	Amp Time(µs): 1.92	Resolution:(eV) 127.4

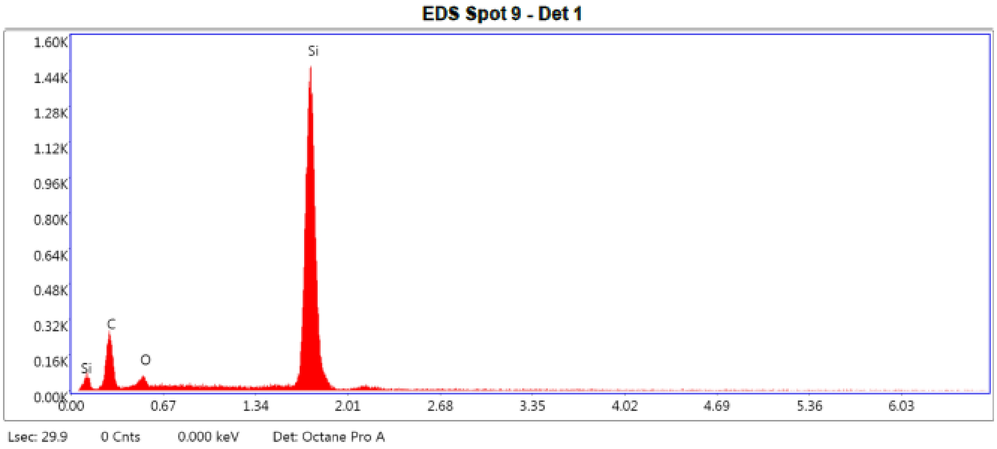

eZAF Smart Quant Results

Element	Weight %	Atomic %	Net Int.	Error %	Kratio	Z	A	F
C K	39.84	59.94	95.46	12.47	0.0801	1.1009	0.1826	1.0000
O K	2.77	3.13	19.60	16.91	0.0102	1.0408	0.3528	1.0000
SiK	57.39	36.93	797.15	2.88	0.5260	0.9240	0.9913	1.0004

EDS Spot 10

kV: 10	Mag: 1546	Takeoff: 34	Live Time(s): 29.9	Amp Time(µs): 1.92	Resolution:(eV) 127.4

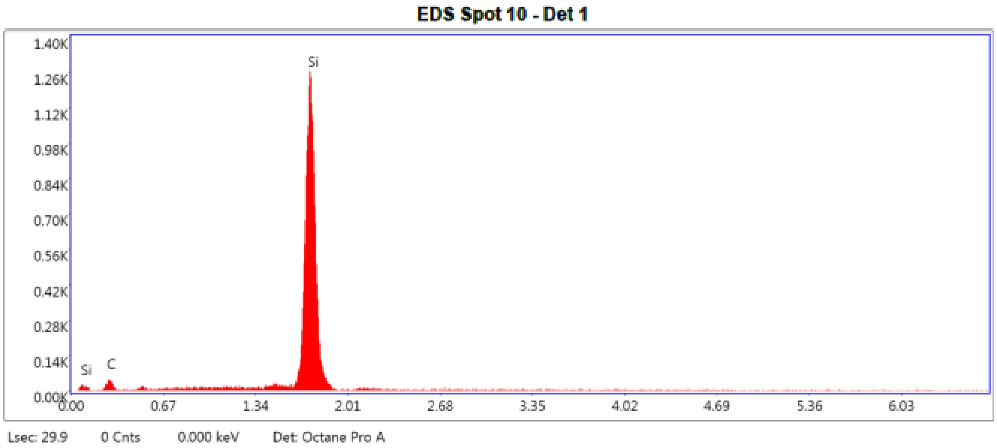

eZAF Smart Quant Results

Element	Weight %	Atomic %	Net Int.	Error %	Kratio	Z	A	F
C K	12.35	24.78	11.17	22.17	0.0173	1.1598	0.1207	1.0000
SiK	87.65	75.22	701.95	2.84	0.8543	0.9764	0.9982	1.0001

EDS Spot 11

kV: 10	Mag: 1546	Takeoff: 34	Live Time(s): 29.8	Amp Time(µs): 1.92	Resolution:(eV) 127.4

EDS Spot 11 - Det 1

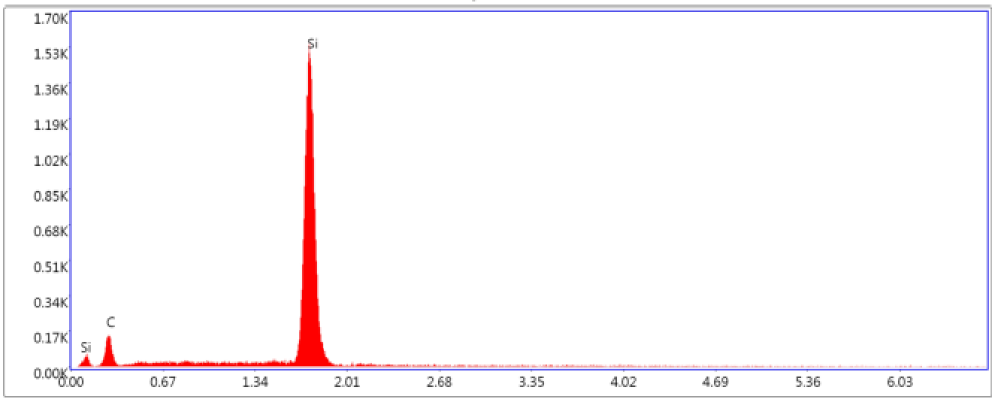

Lsec: 29.8   0 Cnts   0.000 keV   Det: Octane Pro A

eZAF Smart Quant Results

Element	Weight %	Atomic %	Net Int.	Error %	Kratio	Z	A	F
C K	29.17	49.06	48.87	14.26	0.0490	1.1252	0.1493	1.0000
SiK	70.83	50.94	845.91	2.81	0.6672	0.9457	0.9957	1.0002

EDS Spot 13

kV: 10	Mag: 1546	Takeoff: 34	Live Time(s): 29.8	Amp Time(µs): 1.92	Resolution:(eV) 127.4

EDS Spot 13 - Det 1

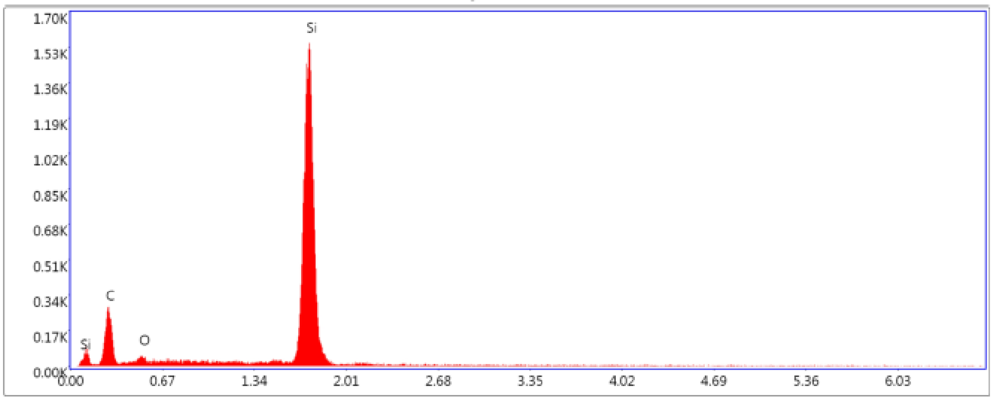

Lsec: 29.8   0 Cnts   0.000 keV   Det: Octane Pro A

eZAF Smart Quant Results

Element	Weight %	Atomic %	Net Int.	Error %	Kratio	Z	A	F
C K	40.11	60.64	99.78	12.46	0.0794	1.1021	0.1795	1.0000
O K	1.31	1.48	9.67	29.98	0.0048	1.0421	0.3492	1.0000
SiK	58.58	37.87	860.78	2.86	0.5384	0.9252	0.9928	1.0004

EDS Spot 14

kV: 10	Mag: 1546	Takeoff: 34	Live Time(s): 29.8	Amp Time(µs): 1.92	Resolution:(eV) 127.4

EDS Spot 14 - Det 1

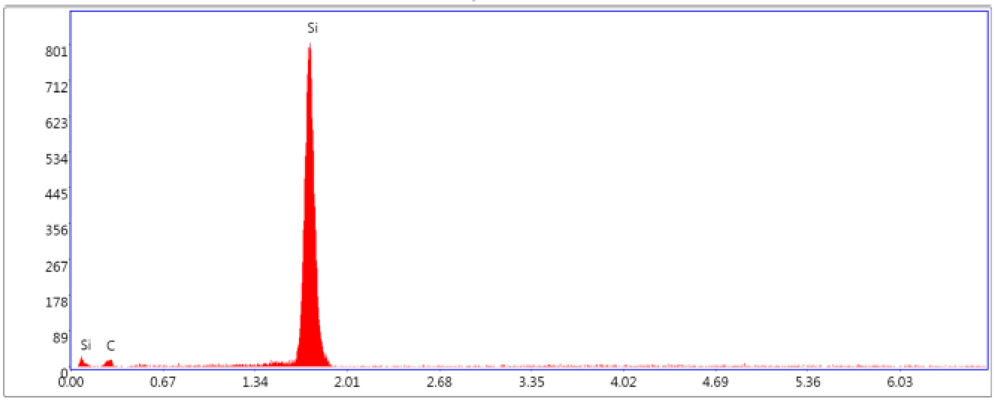

Lsec: 29.8 0 Cnts 0.000 keV Det: Octane Pro A

eZAF Smart Quant Results

Element	Weight %	Atomic %	Net Int.	Error %	Kratio	Z	A	F
C K	10.34	21.24	5.79	24.27	0.0142	1.1641	0.1180	1.0000
SiK	89.66	78.76	454.92	2.99	0.8775	0.9802	0.9985	1.0001

EDS Spot 15

kV: 10	Mag: 1546	Takeoff: 34	Live Time(s): 29.9	Amp Time(µs): 1.92	Resolution:(eV) 127.4

EDS Spot 15 - Det 1

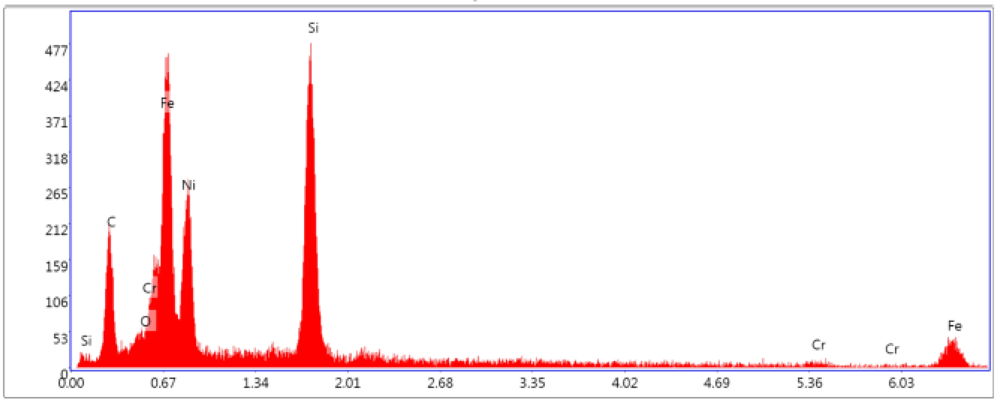

Lsec: 29.9 0 Cnts 0.000 keV Det: Octane Pro A

eZAF Smart Quant Results

Element	Weight %	Atomic %	Net Int.	Error %	Kratio	Z	A	F
C K	22.68	51.40	77.84	12.14	0.0734	1.2372	0.2615	1.0000
O K	0.38	0.64	3.58	99.99	0.0021	1.1739	0.4742	1.0000
FeL	30.44	14.84	143.82	4.91	0.2293	0.8759	0.8600	1.0000
NiL	20.48	9.50	87.31	8.08	0.1240	0.8870	0.6824	1.0000
SiK	22.43	21.74	264.12	5.46	0.1957	1.0499	0.8296	1.0018
CrK	3.60	1.88	6.02	44.97	0.0357	0.8661	0.9982	1.1471

EDS Spot 16

kV: 10	Mag: 1546	Takeoff: 34	Live Time(s): 29.8	Amp Time(µs): 1.92	Resolution:(eV) 127.4

EDS Spot 16 - Det 1

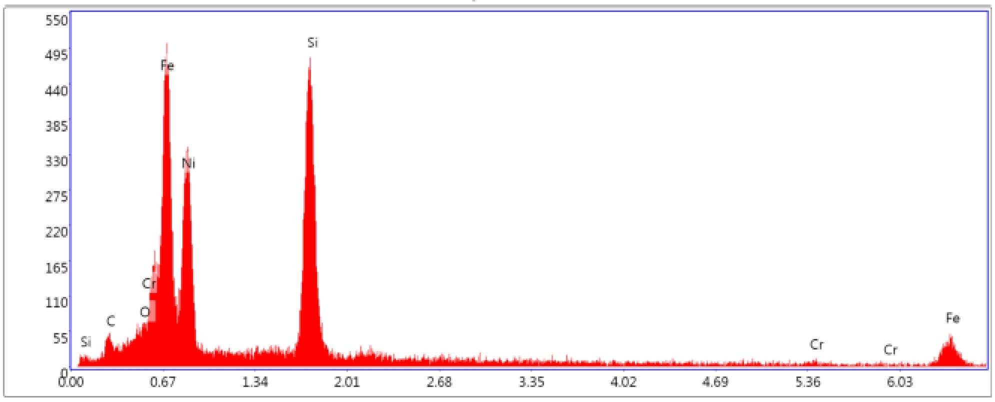

Lsec: 29.8 0 Cnts 0.000 keV Det: Octane Pro A

eZAF Smart Quant Results

Element	Weight %	Atomic %	Net Int.	Error %	Kratio	Z	A	F
C K	4.12	13.48	10.27	25.50	0.0116	1.3114	0.2136	1.0000
O K	0.56	1.38	5.55	68.51	0.0039	1.2454	0.5516	1.0000
FeL	32.91	23.13	150.03	4.36	0.2855	0.9296	0.9332	1.0000
NiL	32.04	21.42	119.81	7.40	0.2030	0.9417	0.6728	1.0000
SiK	27.49	38.42	274.76	5.77	0.2430	1.1161	0.7906	1.0016
CrK	2.87	2.17	4.30	64.65	0.0304	0.9277	0.9946	1.1477

EDS Spot 17

kV: 10	Mag: 1546	Takeoff: 34	Live Time(s): 29.9	Amp Time(µs): 1.92	Resolution:(eV) 127.4

EDS Spot 17 - Det 1

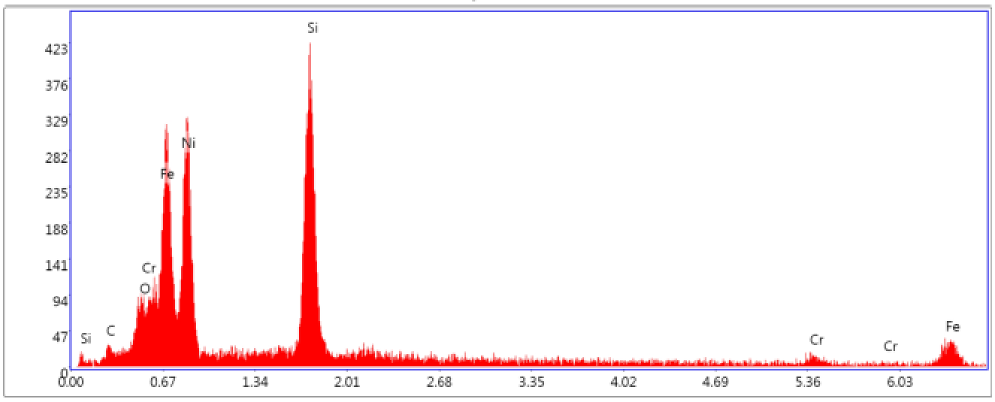

Lsec: 29.9 0 Cnts 0.000 keV Det: Octane Pro A

eZAF Smart Quant Results

Element	Weight %	Atomic %	Net Int.	Error %	Kratio	Z	A	F
C K	1.50	5.05	2.92	75.26	0.0042	1.3155	0.2116	1.0000
O K	2.52	6.37	20.27	15.41	0.0179	1.2494	0.5699	1.0000
FeL	25.34	18.38	84.71	5.81	0.2046	0.9326	0.8659	1.0000
NiL	35.97	24.82	110.61	7.23	0.2379	0.9447	0.7001	1.0000
SiK	27.65	39.90	217.70	6.20	0.2444	1.1197	0.7879	1.0015
CrK	7.03	5.48	8.07	38.23	0.0724	0.9310	0.9943	1.1132

EDS Spot 18

kV: 10	Mag: 1546	Takeoff: 34	Live Time(s): 29.8	Amp Time(μs): 1.92	Resolution:(eV) 127.4

EDS Spot 18 - Det 1

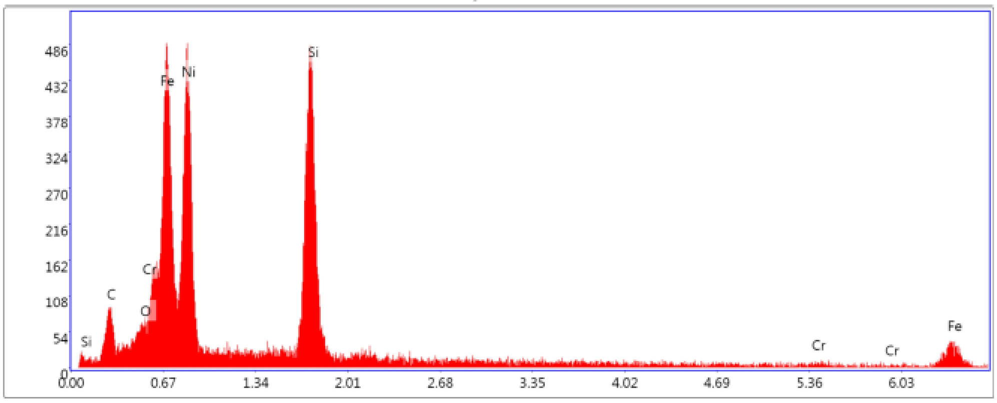

Lsec: 29.8 0 Cnts 0.000 keV Det: Octane Pro A

eZAF Smart Quant Results

Element	Weight %	Atomic %	Net Int.	Error %	Kratio	Z	A	F
C K	9.59	28.09	29.83	16.26	0.0286	1.2920	0.2308	1.0000
O K	0.78	1.72	8.57	36.30	0.0051	1.2269	0.5295	1.0000
FeL	26.80	16.89	139.07	4.57	0.2256	0.9157	0.9190	1.0000
NiL	36.19	21.69	165.70	6.63	0.2393	0.9276	0.7129	1.0000
SiK	23.56	29.52	272.43	5.84	0.2053	1.0991	0.7918	1.0016
CrK	3.08	2.08	5.29	54.28	0.0319	0.9123	0.9956	1.1404

EDS Spot 19

kV: 10	Mag: 1546	Takeoff: 34	Live Time(s): 29.9	Amp Time(μs): 1.92	Resolution:(eV) 127.4

EDS Spot 19 - Det 1

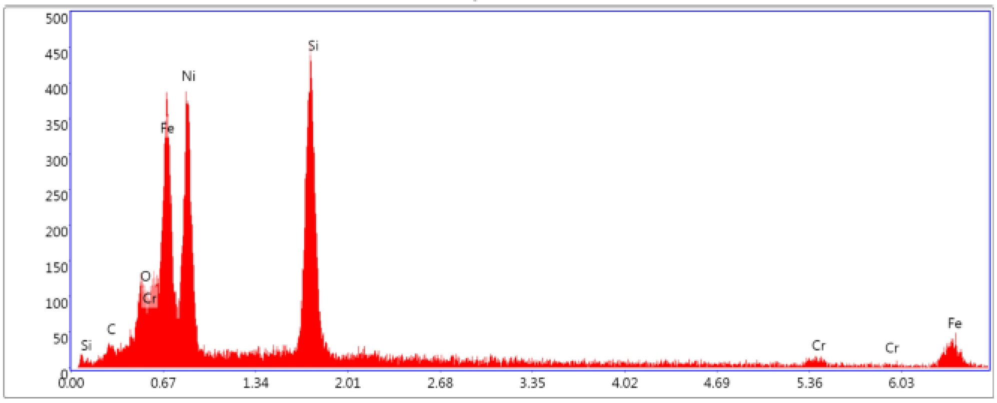

Lsec: 29.9 0 Cnts 0.000 keV Det: Octane Pro A

eZAF Smart Quant Results

Element	Weight %	Atomic %	Net Int.	Error %	Kratio	Z	A	F
C K	2.75	9.08	6.83	32.42	0.0079	1.3131	0.2198	1.0000
O K	2.91	7.21	28.72	14.56	0.0207	1.2471	0.5693	1.0000
FeL	26.40	18.72	107.98	5.51	0.2124	0.9309	0.8642	1.0000
NiL	35.94	24.24	133.59	7.05	0.2340	0.9429	0.6905	1.0000
SiK	25.30	35.66	243.37	5.97	0.2225	1.1176	0.7857	1.0016
CrK	6.70	5.10	9.46	33.07	0.0692	0.9292	0.9947	1.1180

EDS Spot 20

kV: 10	Mag: 1546	Takeoff: 34	Live Time(s): 29.8	Amp Time(μs): 1.92	Resolution:(eV) 127.4

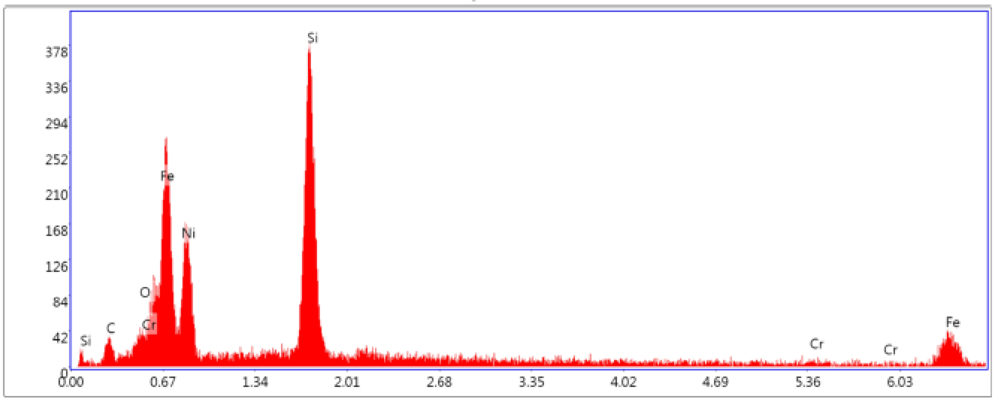

eZAF Smart Quant Results

Element	Weight %	Atomic %	Net Int.	Error %	Kratio	Z	A	F
C K	5.51	16.44	7.56	27.00	0.0143	1.2884	0.2010	1.0000
O K	1.05	2.36	5.89	38.22	0.0069	1.2232	0.5328	1.0000
FeL	31.24	20.06	80.23	5.48	0.2560	0.9129	0.8977	1.0000
NiL	24.01	14.66	53.51	8.52	0.1521	0.9246	0.6850	1.0000
SiK	34.32	43.81	207.36	5.91	0.3075	1.0951	0.8170	1.0015
CrK	3.87	2.67	3.33	65.00	0.0395	0.9073	0.9947	1.1329

EDS Spot 21

kV: 10	Mag: 1546	Takeoff: 34	Live Time(s): 29.9	Amp Time(μs): 1.92	Resolution:(eV) 127.4

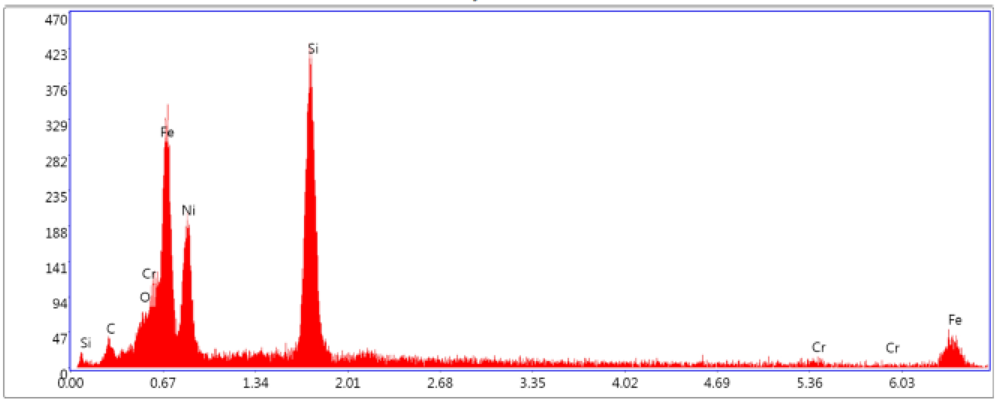

eZAF Smart Quant Results

Element	Weight %	Atomic %	Net Int.	Error %	Kratio	Z	A	F
C K	4.12	12.87	7.75	26.90	0.0113	1.3010	0.2110	1.0000
O K	1.73	4.07	13.13	19.61	0.0119	1.2353	0.5537	1.0000
FeL	32.66	21.94	102.72	5.55	0.2538	0.9220	0.8426	1.0000
NiL	23.47	14.99	64.75	8.79	0.1424	0.9339	0.6500	1.0000
SiK	30.44	40.66	237.37	5.67	0.2725	1.1064	0.8078	1.0016
CrK	7.57	5.46	8.43	37.13	0.0774	0.9178	0.9950	1.1185

EDS Spot 22

kV: 10	Mag: 1546	Takeoff: 34	Live Time(s): 29.8	Amp Time(µs): 1.92	Resolution:(eV) 127.4

EDS Spot 22 - Det 1

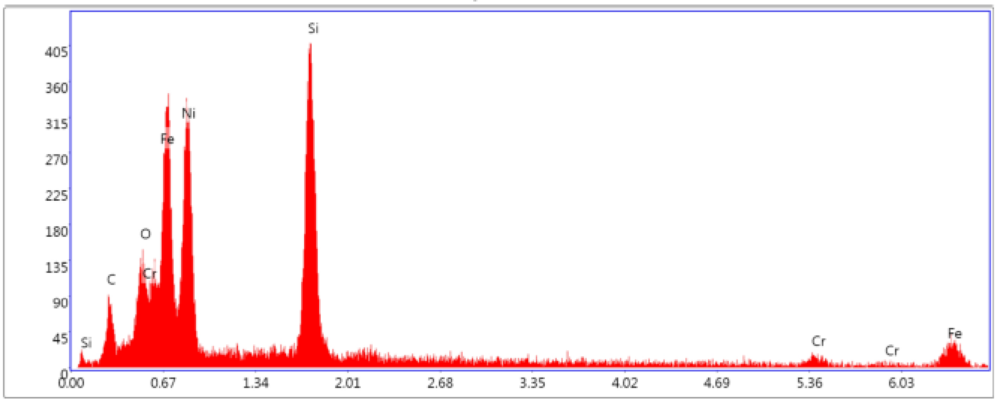

Lsec: 29.8 0 Cnts 0.000 keV Det: Octane Pro A

eZAF Smart Quant Results

Element	Weight %	Atomic %	Net Int.	Error %	Kratio	Z	A	F
C K	9.71	27.01	26.38	16.41	0.0297	1.2807	0.2391	1.0000
O K	3.93	8.20	36.96	12.53	0.0258	1.2159	0.5403	1.0000
FeL	24.61	14.72	96.12	6.04	0.1834	0.9075	0.8211	1.0000
NiL	30.98	17.63	116.28	7.22	0.1975	0.9191	0.6937	1.0000
SiK	23.14	27.52	228.24	5.89	0.2023	1.0888	0.8020	1.0016
CrK	7.64	4.91	10.77	31.43	0.0764	0.9021	0.9963	1.1119

Area 4

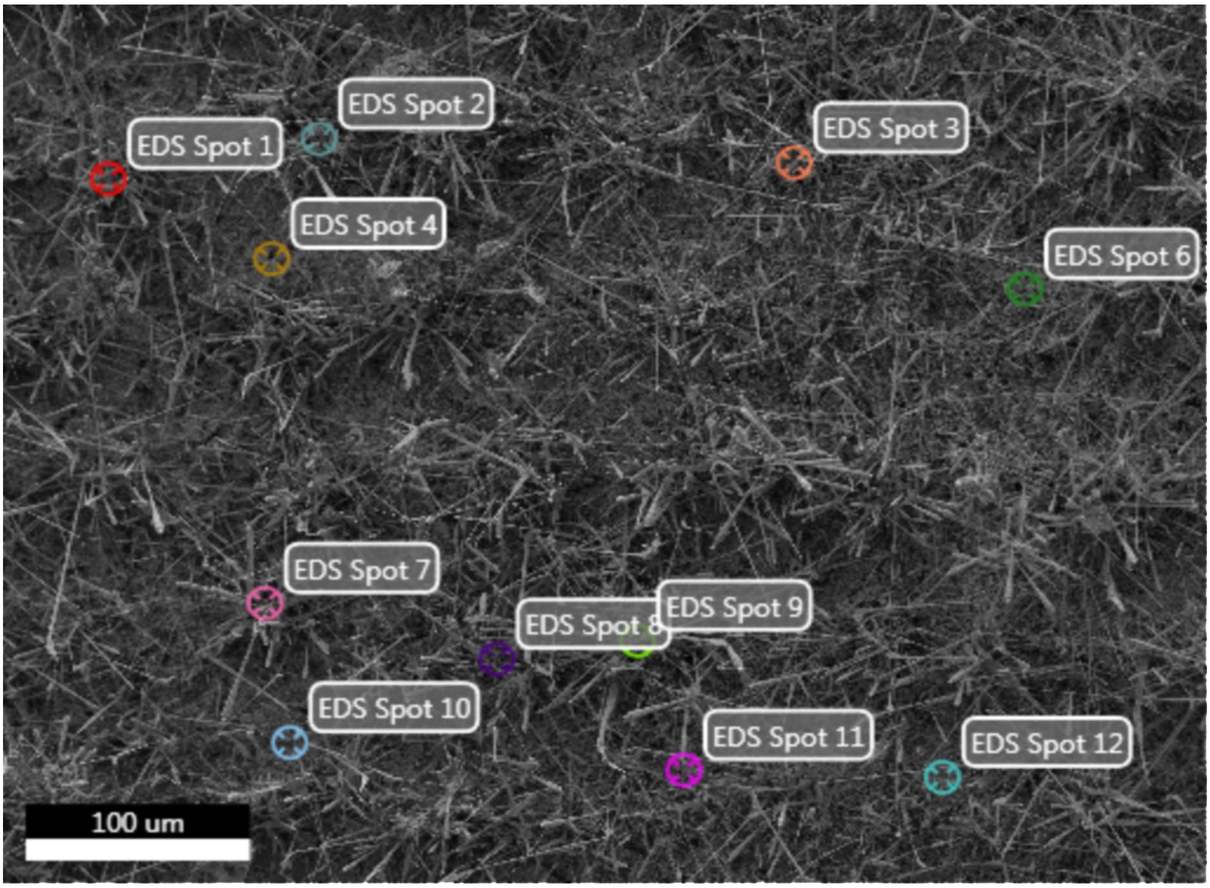
EDS Spot 1
EDS Spot 2
EDS Spot 3
EDS Spot 4
EDS Spot 6
EDS Spot 7
EDS Spot 8
EDS Spot 9
EDS Spot 10
EDS Spot 11
EDS Spot 12
100 um

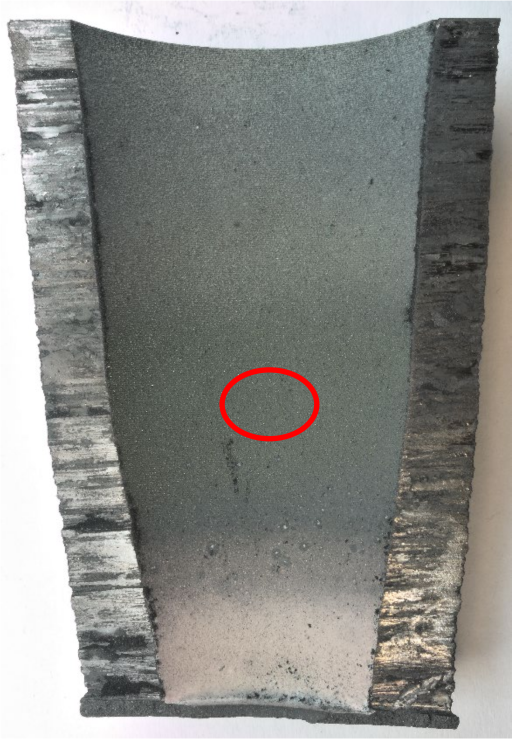

EDS Spot 1

kV: 10	Mag: 205	Takeoff: 34.4	Live Time(s): 29.9	Amp Time(μs): 1.92	Resolution:(eV) 127.4

EDS Spot 1 - Det 1

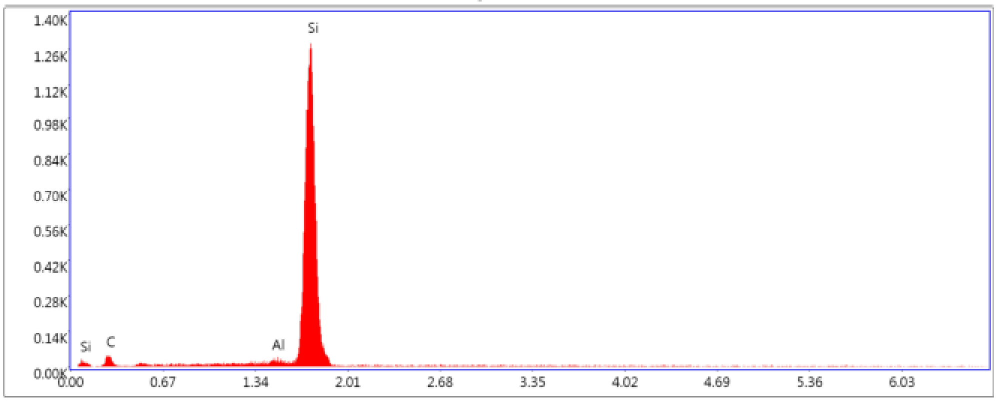

Lsec: 29.9 0 Cnts 0.000 keV Det: Octane Pro A

eZAF Smart Quant Results

Element	Weight %	Atomic %	Net Int.	Error %	Kratio	Z	A	F
C K	12.94	25.78	13.50	19.09	0.0185	1.1591	0.1231	1.0000
AlK	2.22	1.97	21.37	11.05	0.0211	0.9576	0.9777	1.0156
SiK	84.84	72.25	757.60	2.92	0.8183	0.9757	0.9884	1.0001

EDS Spot 2

kV: 10	Mag: 205	Takeoff: 34.4	Live Time(s): 29.8	Amp Time(μs): 1.92	Resolution:(eV) 127.4

EDS Spot 2 - Det 1

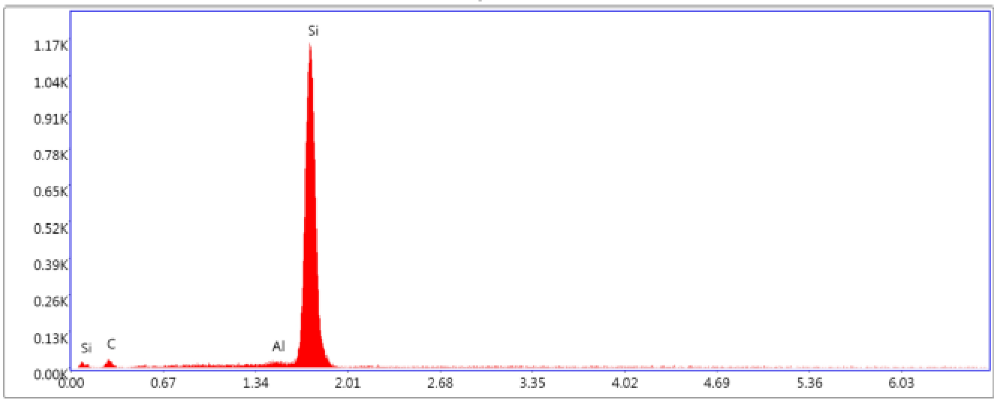

Lsec: 29.8 0 Cnts 0.000 keV Det: Octane Pro A

eZAF Smart Quant Results

Element	Weight %	Atomic %	Net Int.	Error %	Kratio	Z	A	F
C K	7.03	15.02	5.85	28.48	0.0095	1.1715	0.1150	1.0000
AlK	1.59	1.51	13.11	15.60	0.0153	0.9684	0.9792	1.0161
SiK	91.38	83.47	699.73	2.91	0.8945	0.9867	0.9920	1.0000

EDS Spot 3

kV: 10	Mag: 205	Takeoff: 34.4	Live Time(s): 29.9	Amp Time(µs): 1.92	Resolution:(eV) 127.4

EDS Spot 3 - Det 1

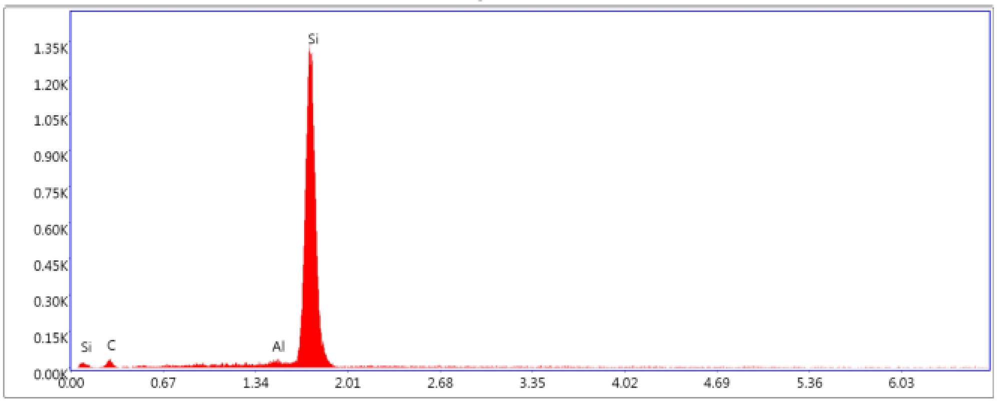

Lsec: 29.9 0 Cnts 0.000 keV Det: Octane Pro A

eZAF Smart Quant Results

Element	Weight %	Atomic %	Net Int.	Error %	Kratio	Z	A	F
C K	7.27	15.47	6.61	28.07	0.0098	1.1712	0.1155	1.0000
AlK	2.25	2.14	20.21	12.78	0.0217	0.9681	0.9792	1.0157
SiK	90.48	82.39	752.46	2.91	0.8828	0.9864	0.9891	1.0001

EDS Spot 4

kV: 10	Mag: 205	Takeoff: 34.4	Live Time(s): 29.9	Amp Time(µs): 1.92	Resolution:(eV) 127.4

EDS Spot 4 - Det 1

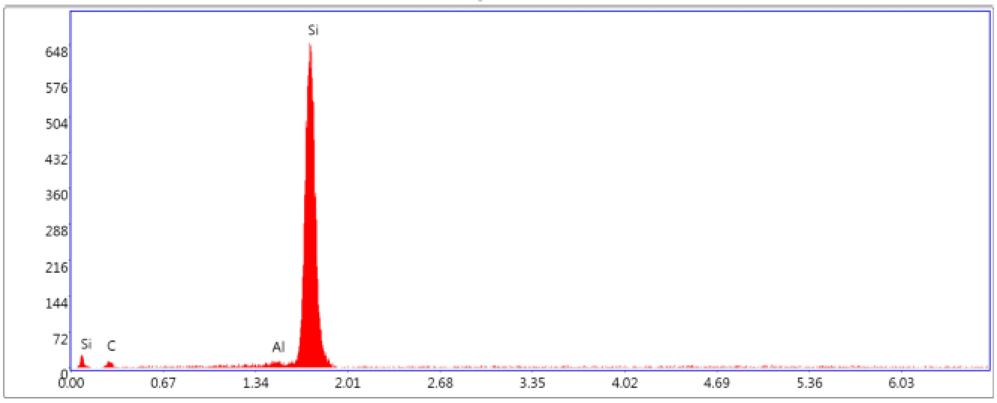

Lsec: 29.9 0 Cnts 0.000 keV Det: Octane Pro A

eZAF Smart Quant Results

Element	Weight %	Atomic %	Net Int.	Error %	Kratio	Z	A	F
C K	7.73	16.37	3.92	28.10	0.0105	1.1702	0.1160	1.0000
AlK	2.10	1.98	10.42	12.69	0.0202	0.9672	0.9791	1.0158
SiK	90.17	81.65	415.62	3.12	0.8795	0.9855	0.9897	1.0001

EDS Spot 6

kV: 10	Mag: 205	Takeoff: 34.4	Live Time(s): 29.8	Amp Time(μs): 1.92	Resolution:(eV) 127.4

EDS Spot 6 - Det 1

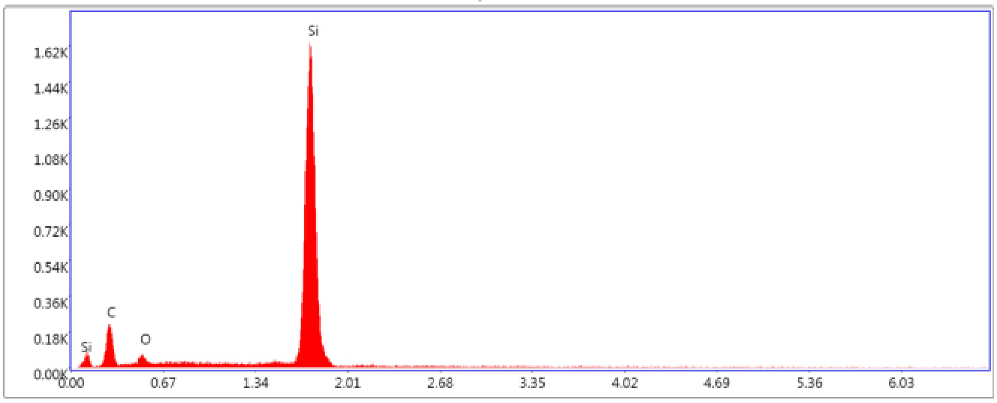

Lsec: 29.8 0 Cnts 0.000 keV Det: Octane Pro A

eZAF Smart Quant Results

Element	Weight %	Atomic %	Net Int.	Error %	Kratio	Z	A	F
C K	34.10	54.14	76.21	13.11	0.0632	1.1127	0.1665	1.0000
O K	2.18	2.59	16.22	19.80	0.0083	1.0522	0.3645	1.0000
SiK	63.72	43.27	903.55	2.83	0.5913	0.9345	0.9928	1.0003

EDS Spot 7

kV: 10	Mag: 205	Takeoff: 34.4	Live Time(s): 29.8	Amp Time(μs): 1.92	Resolution:(eV) 127.4

EDS Spot 7 - Det 1

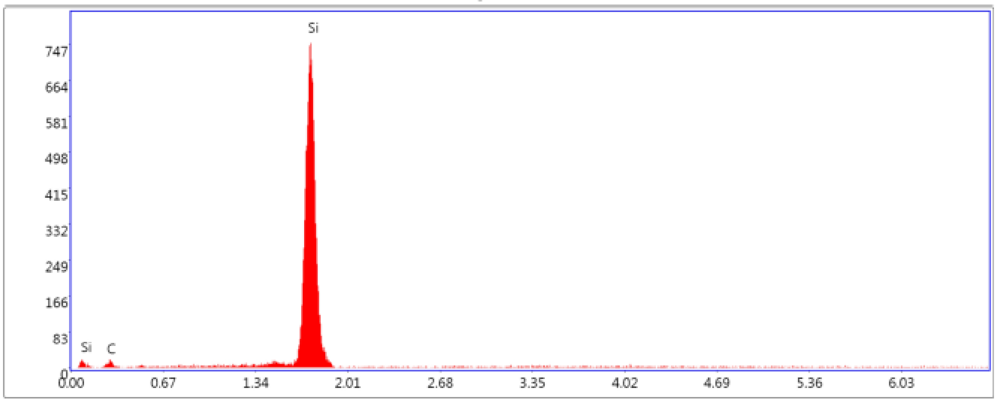

Lsec: 29.8 0 Cnts 0.000 keV Det: Octane Pro A

eZAF Smart Quant Results

Element	Weight %	Atomic %	Net Int.	Error %	Kratio	Z	A	F
C K	8.94	18.66	5.04	25.16	0.0122	1.1671	0.1171	1.0000
SiK	91.06	81.34	467.05	2.98	0.8938	0.9828	0.9987	1.0001

EDS Spot 8

kV: 10	Mag: 205	Takeoff: 34.4	Live Time(s): 29.8	Amp Time(µs): 1.92	Resolution:(eV) 127.4

EDS Spot 8 - Det 1

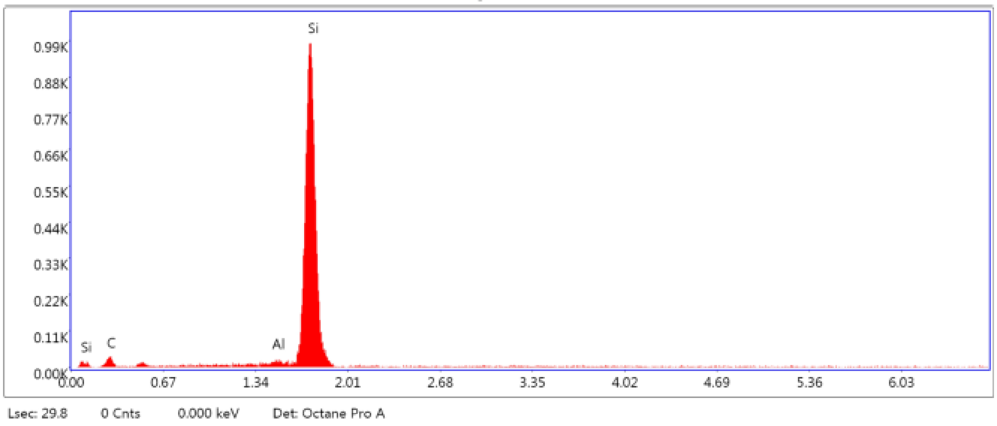

eZAF Smart Quant Results

Element	Weight %	Atomic %	Net Int.	Error %	Kratio	Z	A	F
C K	12.47	24.98	9.33	21.34	0.0177	1.1600	0.1223	1.0000
AlK	1.87	1.67	13.00	13.80	0.0178	0.9584	0.9778	1.0158
SiK	85.66	73.36	553.15	3.01	0.8282	0.9765	0.9900	1.0001

EDS Spot 9

kV: 10	Mag: 205	Takeoff: 34.4	Live Time(s): 29.9	Amp Time(µs): 1.92	Resolution:(eV) 127.4

EDS Spot 9 - Det 1

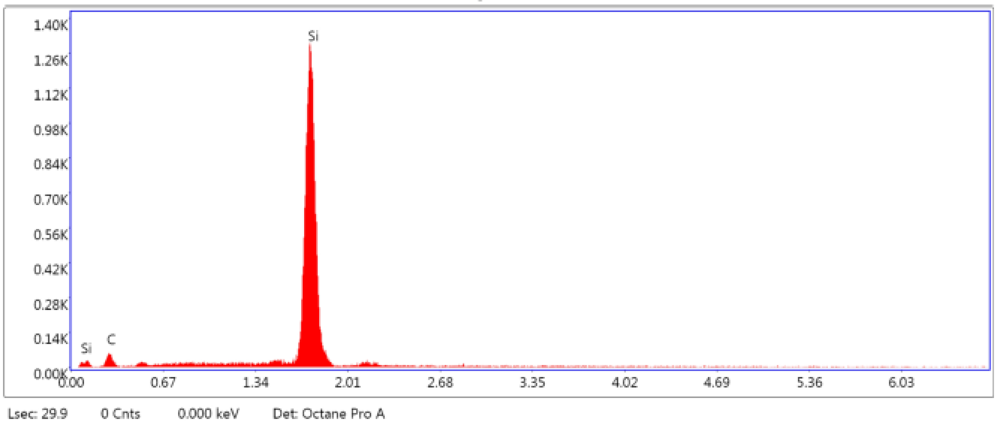

eZAF Smart Quant Results

Element	Weight %	Atomic %	Net Int.	Error %	Kratio	Z	A	F
C K	13.76	27.17	13.41	21.69	0.0197	1.1568	0.1237	1.0000
SiK	86.24	72.83	723.29	2.86	0.8382	0.9738	0.9980	1.0001

EDS Spot 10

kV: 10	Mag: 205	Takeoff: 34.4	Live Time(s): 29.8	Amp Time(µs): 1.92	Resolution:(eV) 127.4

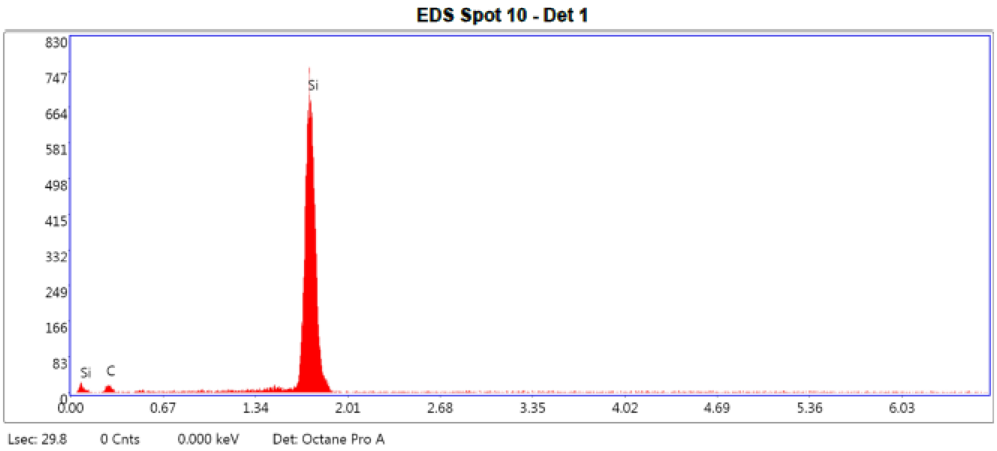

eZAF Smart Quant Results

Element	Weight %	Atomic %	Net Int.	Error %	Kratio	Z	A	F
C K	9.93	20.49	4.95	28.83	0.0137	1.1649	0.1184	1.0000
SiK	90.07	79.51	403.68	3.06	0.8622	0.9809	0.9985	1.0001

EDS Spot 11

kV: 10	Mag: 205	Takeoff: 34.4	Live Time(s): 29.9	Amp Time(µs): 1.92	Resolution:(eV) 127.4

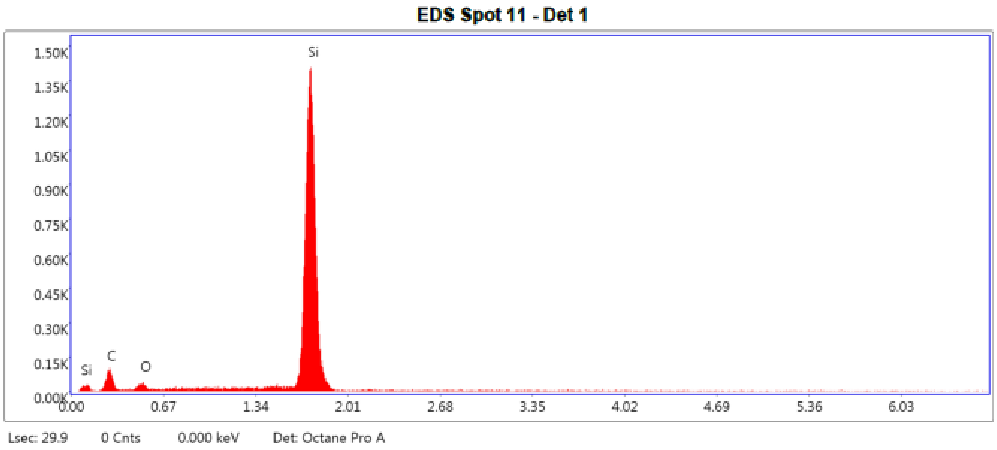

eZAF Smart Quant Results

Element	Weight %	Atomic %	Net Int.	Error %	Kratio	Z	A	F
C K	23.12	40.93	32.59	15.39	0.0371	1.1354	0.1413	1.0000
O K	1.52	2.02	8.86	25.11	0.0063	1.0742	0.3840	1.0000
SiK	75.36	57.05	796.80	2.84	0.7159	0.9547	0.9950	1.0002

EDS Spot 12

kV: 10	Mag: 205	Takeoff: 34.4	Live Time(s): 29.8	Amp Time(µs): 1.92	Resolution:(eV) 127.4

EDS Spot 12 - Det 1

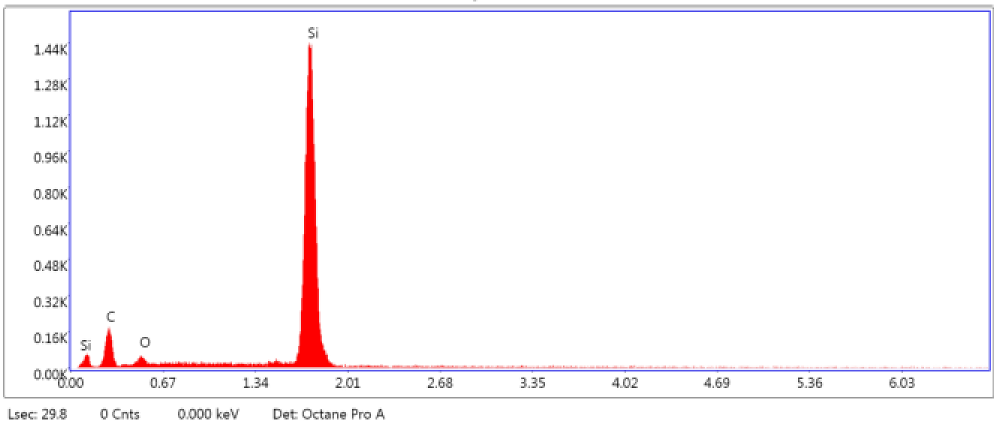

eZAF Smart Quant Results

Element	Weight %	Atomic %	Net Int.	Error %	Kratio	Z	A	F
C K	32.17	52.09	61.18	13.43	0.0578	1.1170	0.1608	1.0000
O K	1.79	2.18	11.86	24.22	0.0070	1.0564	0.3673	1.0000
SiK	66.04	45.73	826.01	2.85	0.6161	0.9384	0.9934	1.0003

Area 5

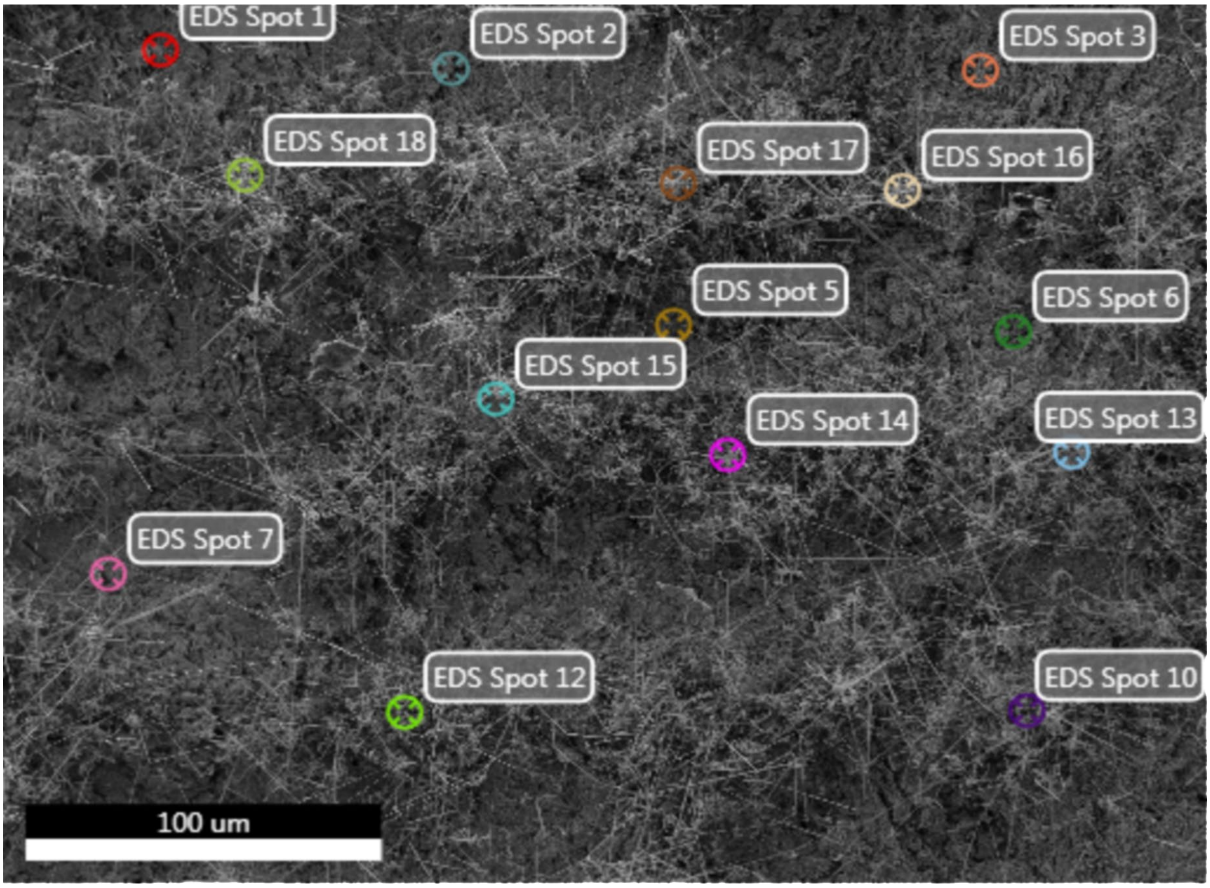
EDS Spot 1
EDS Spot 2
EDS Spot 3
EDS Spot 18
EDS Spot 17
EDS Spot 16
EDS Spot 5
EDS Spot 6
EDS Spot 15
EDS Spot 14
EDS Spot 13
EDS Spot 7
EDS Spot 12
EDS Spot 10
100 um

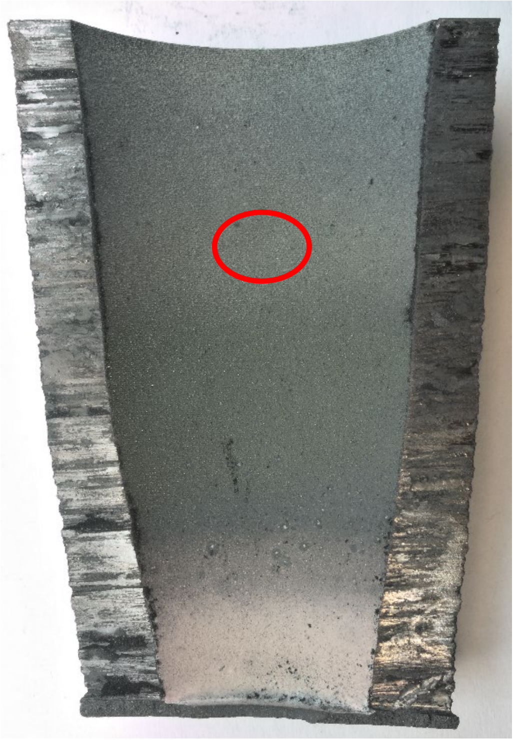

EDS Spot 1

kV: 10	Mag: 325	Takeoff: 34.5	Live Time(s): 29.8	Amp Time(µs): 1.92	Resolution:(eV) 127.4

EDS Spot 1 - Det 1

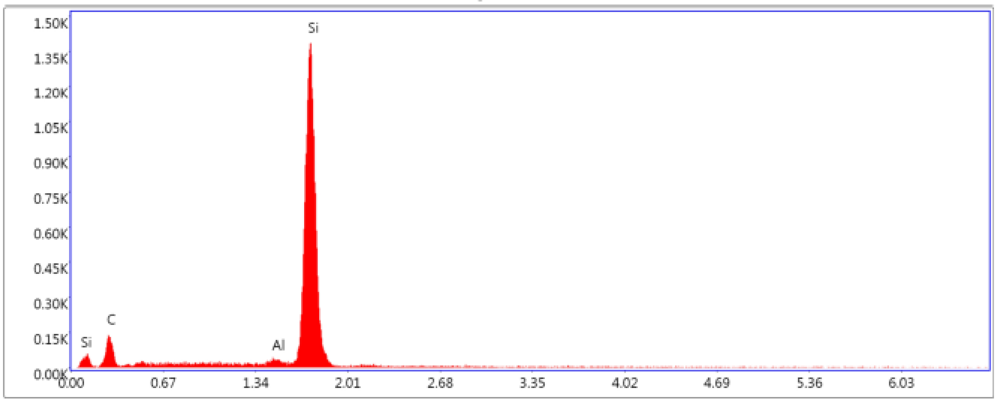

Lsec: 29.8 0 Cnts 0.000 keV Det: Octane Pro A

eZAF Smart Quant Results

Element	Weight %	Atomic %	Net Int.	Error %	Kratio	Z	A	F
C K	29.27	49.15	47.62	14.24	0.0500	1.1253	0.1517	1.0000
AlK	1.39	1.04	16.86	14.86	0.0128	0.9284	0.9733	1.0154
SiK	69.34	49.80	783.01	2.92	0.6493	0.9458	0.9897	1.0002

EDS Spot 2

kV: 10	Mag: 325	Takeoff: 34.5	Live Time(s): 29.9	Amp Time(µs): 1.92	Resolution:(eV) 127.4

EDS Spot 2 - Det 1

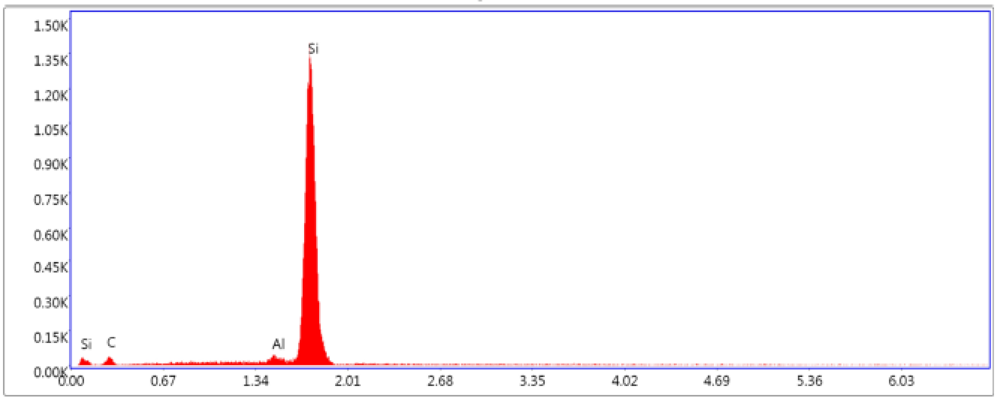

Lsec: 29.9 0 Cnts 0.000 keV Det: Octane Pro A

eZAF Smart Quant Results

Element	Weight %	Atomic %	Net Int.	Error %	Kratio	Z	A	F
C K	8.09	17.05	7.62	27.47	0.0110	1.1694	0.1168	1.0000
AlK	2.24	2.10	20.53	12.23	0.0215	0.9666	0.9791	1.0157
SiK	89.68	80.85	762.66	2.91	0.8735	0.9848	0.9891	1.0001

EDS Spot 3

kV: 10	Mag: 325	Takeoff: 34.5	Live Time(s): 29.8	Amp Time(μs): 1.92	Resolution:(eV) 127.4

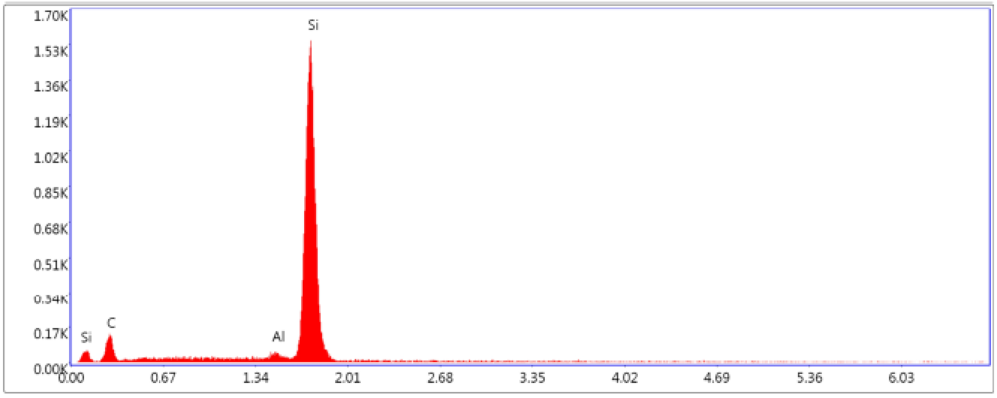

eZAF Smart Quant Results

Element	Weight %	Atomic %	Net Int.	Error %	Kratio	Z	A	F
C K	26.81	46.11	44.42	14.86	0.0444	1.1303	0.1467	1.0000
AlK	1.55	1.19	19.74	13.40	0.0143	0.9327	0.9740	1.0154
SiK	71.64	52.70	851.67	2.88	0.6737	0.9502	0.9894	1.0002

EDS Spot 5

kV: 10	Mag: 325	Takeoff: 34.5	Live Time(s): 29.8	Amp Time(μs): 1.92	Resolution:(eV) 127.4

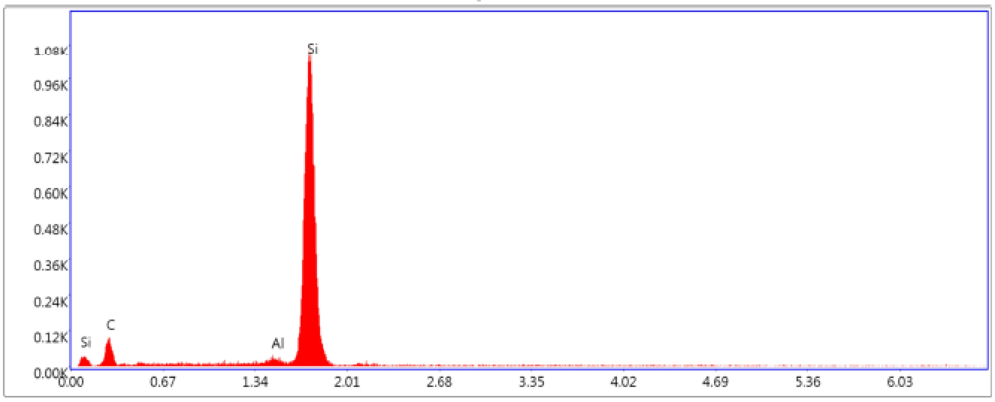

eZAF Smart Quant Results

Element	Weight %	Atomic %	Net Int.	Error %	Kratio	Z	A	F
C K	27.54	47.03	33.68	14.83	0.0461	1.1289	0.1483	1.0000
AlK	1.88	1.43	17.51	11.58	0.0173	0.9315	0.9739	1.0152
SiK	70.57	51.54	611.56	3.00	0.6618	0.9489	0.9879	1.0002

EDS Spot 6

kV: 10	Mag: 325	Takeoff: 34.5	Live Time(s): 29.8	Amp Time(µs): 1.92	Resolution:(eV) 127.4

EDS Spot 6 - Det 1

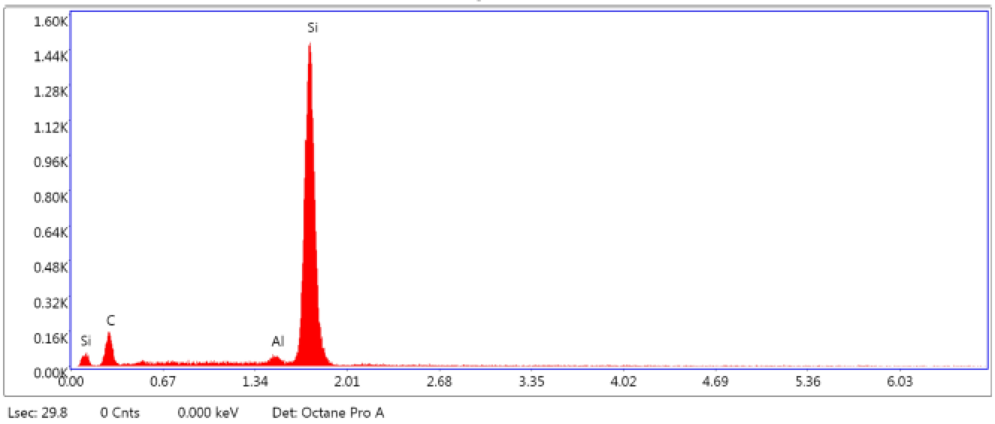

eZAF Smart Quant Results

Element	Weight %	Atomic %	Net Int.	Error %	Kratio	Z	A	F
C K	30.17	50.23	54.17	13.68	0.0522	1.1236	0.1538	1.0000
AlK	1.90	1.41	25.05	10.77	0.0174	0.9269	0.9732	1.0151
SiK	67.92	48.36	832.36	2.91	0.6336	0.9443	0.9874	1.0003

EDS Spot 7

kV: 10	Mag: 325	Takeoff: 34.5	Live Time(s): 29.8	Amp Time(µs): 1.92	Resolution:(eV) 127.4

EDS Spot 7 - Det 1

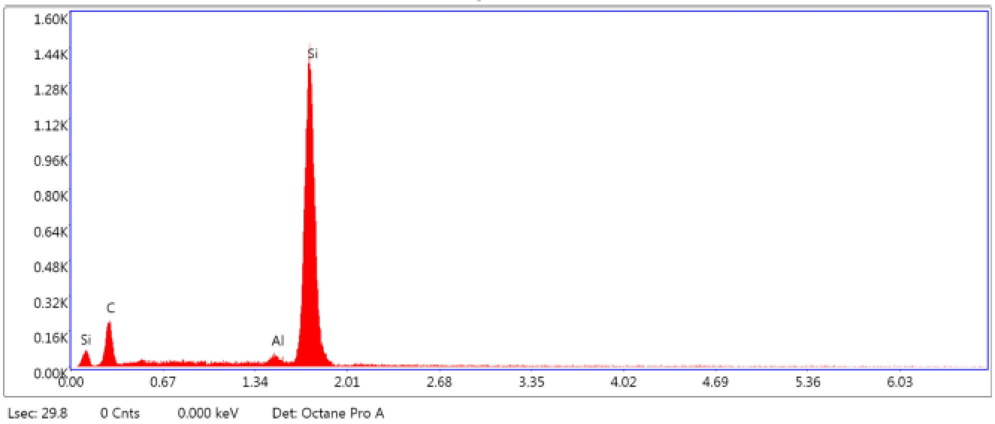

eZAF Smart Quant Results

Element	Weight %	Atomic %	Net Int.	Error %	Kratio	Z	A	F
C K	36.10	56.89	74.23	13.13	0.0674	1.1118	0.1680	1.0000
AlK	1.72	1.21	23.66	12.03	0.0155	0.9166	0.9716	1.0149
SiK	62.18	41.90	798.68	2.92	0.5733	0.9338	0.9874	1.0003

EDS Spot 10

kV: 10	Mag: 325	Takeoff: 34.5	Live Time(s): 29.8	Amp Time(µs): 1.92	Resolution:(eV) 127.4

EDS Spot 10 - Det 1

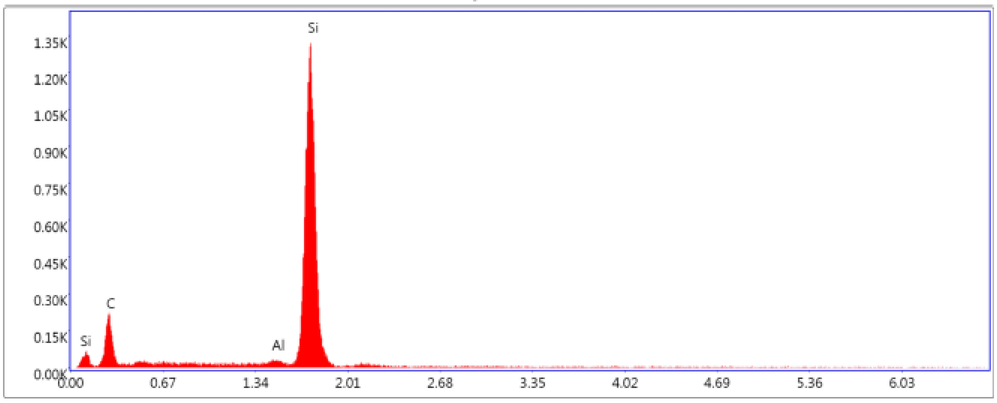

Lsec: 29.8 0 Cnts 0.000 keV Det: Octane Pro A

eZAF Smart Quant Results

Element	Weight %	Atomic %	Net Int.	Error %	Kratio	Z	A	F
C K	39.46	60.37	79.94	12.70	0.0771	1.1052	0.1766	1.0000
AlK	1.04	0.71	13.39	18.02	0.0093	0.9109	0.9706	1.0152
SiK	59.50	38.92	717.46	2.97	0.5468	0.9279	0.9898	1.0004

EDS Spot 12

kV: 10	Mag: 325	Takeoff: 34.5	Live Time(s): 29.8	Amp Time(µs): 1.92	Resolution:(eV) 127.4

EDS Spot 12 - Det 1

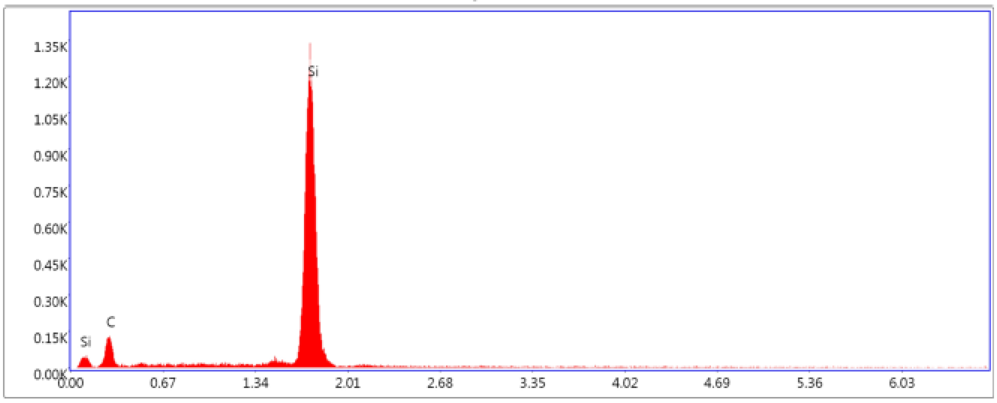

Lsec: 29.8 0 Cnts 0.000 keV Det: Octane Pro A

eZAF Smart Quant Results

Element	Weight %	Atomic %	Net Int.	Error %	Kratio	Z	A	F
C K	30.78	50.97	46.95	13.87	0.0533	1.1220	0.1544	1.0000
SiK	69.22	49.03	724.03	2.87	0.6501	0.9429	0.9955	1.0003

EDS Spot 13

kV: 10	Mag: 325	Takeoff: 34.5	Live Time(s): 29.8	Amp Time(µs): 1.92	Resolution:(eV) 127.4

EDS Spot 13 - Det 1

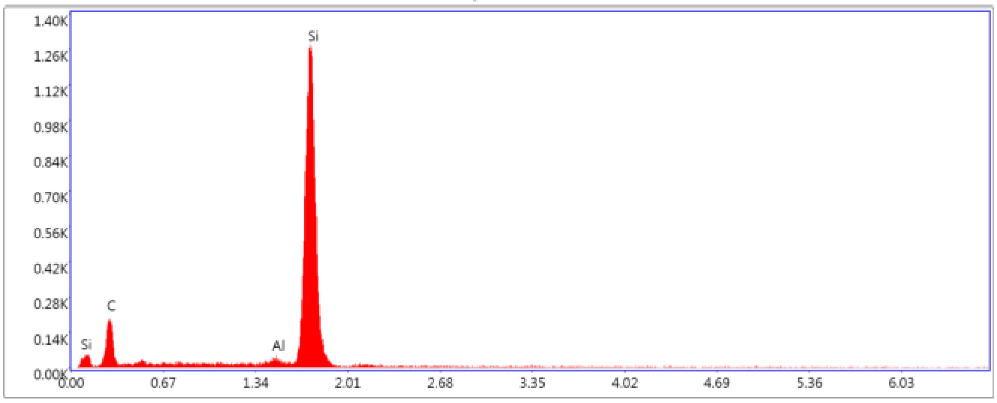

Lsec: 29.8 0 Cnts 0.000 keV Det: Octane Pro A

eZAF Smart Quant Results

Element	Weight %	Atomic %	Net Int.	Error %	Kratio	Z	A	F
C K	37.02	57.86	71.06	13.16	0.0699	1.1099	0.1702	1.0000
AlK	1.28	0.89	16.22	15.13	0.0115	0.9150	0.9713	1.0151
SiK	61.70	41.25	731.60	2.95	0.5689	0.9321	0.9891	1.0003

EDS Spot 14

kV: 10	Mag: 325	Takeoff: 34.5	Live Time(s): 29.8	Amp Time(µs): 1.92	Resolution:(eV) 127.4

EDS Spot 14 - Det 1

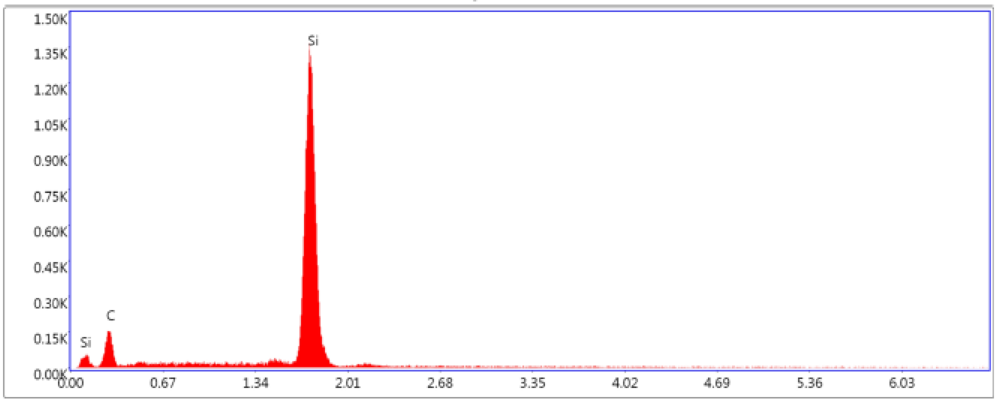

Lsec: 29.8 0 Cnts 0.000 keV Det: Octane Pro A

eZAF Smart Quant Results

Element	Weight %	Atomic %	Net Int.	Error %	Kratio	Z	A	F
C K	33.54	54.13	58.37	13.50	0.0602	1.1165	0.1608	1.0000
SiK	66.46	45.87	760.84	2.86	0.6204	0.9380	0.9951	1.0003

EDS Spot 15

kV: 10	Mag: 325	Takeoff: 34.5	Live Time(s): 29.8	Amp Time(μs): 1.92	Resolution:(eV) 127.4

EDS Spot 15 - Det 1

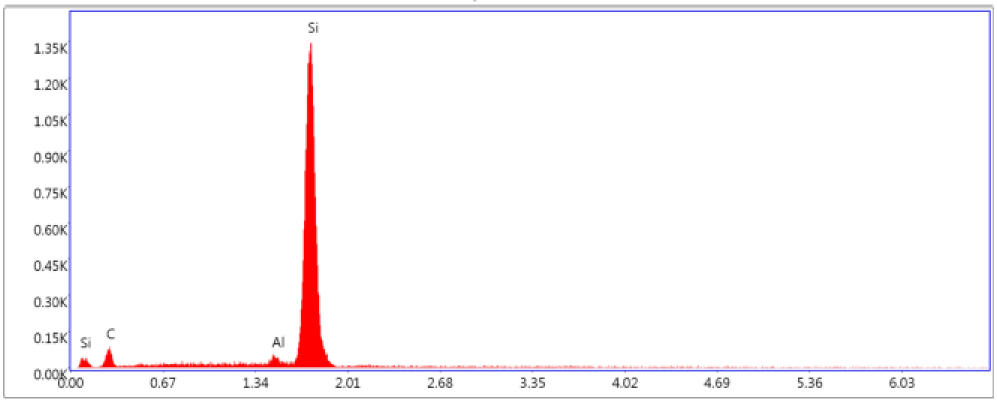

Lsec: 29.8 0 Cnts 0.000 keV Det: Octane Pro A

eZAF Smart Quant Results

Element	Weight %	Atomic %	Net Int.	Error %	Kratio	Z	A	F
C K	18.12	34.08	22.11	17.81	0.0273	1.1481	0.1312	1.0000
AlK	2.05	1.72	21.59	12.58	0.0193	0.9482	0.9764	1.0155
SiK	79.83	64.20	780.73	2.91	0.7623	0.9660	0.9885	1.0001

EDS Spot 16

kV: 10	Mag: 325	Takeoff: 34.5	Live Time(s): 29.8	Amp Time(μs): 1.92	Resolution:(eV) 127.4

EDS Spot 16 - Det 1

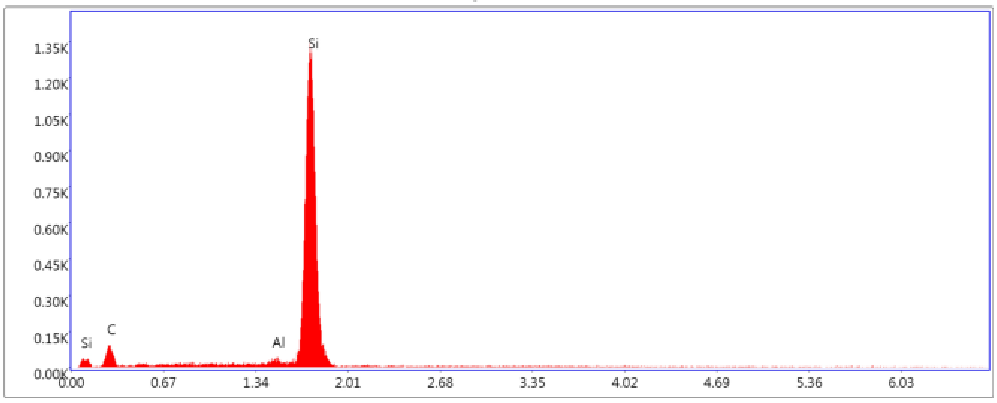

Lsec: 29.8 0 Cnts 0.000 keV Det: Octane Pro A

eZAF Smart Quant Results

Element	Weight %	Atomic %	Net Int.	Error %	Kratio	Z	A	F
C K	23.81	42.20	32.65	15.38	0.0381	1.1364	0.1410	1.0000
AlK	1.79	1.41	19.69	12.88	0.0166	0.9380	0.9748	1.0154
SiK	74.40	56.39	761.06	2.92	0.7030	0.9556	0.9888	1.0002

EDS Spot 17

kV: 10 Mag: 325 Takeoff: 34.5 Live Time(s): 29.9 Amp Time(µs): 1.92 Resolution:(eV) 127.4

EDS Spot 17 - Det 1

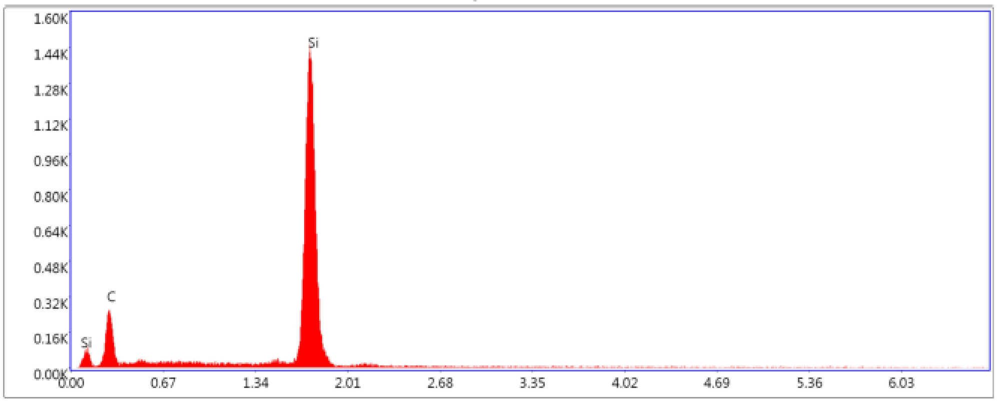

Lsec: 29.9 0 Cnts 0.000 keV Det: Octane Pro A

eZAF Smart Quant Results

Element	Weight %	Atomic %	Net Int.	Error %	Kratio	Z	A	F
C K	41.60	62.49	101.45	12.21	0.0835	1.1009	0.1823	1.0000
SiK	58.40	37.51	824.76	2.85	0.5368	0.9241	0.9940	1.0004

EDS Spot 18

kV: 10 Mag: 325 Takeoff: 34.5 Live Time(s): 29.8 Amp Time(µs): 1.92 Resolution:(eV) 127.4

EDS Spot 18 - Det 1

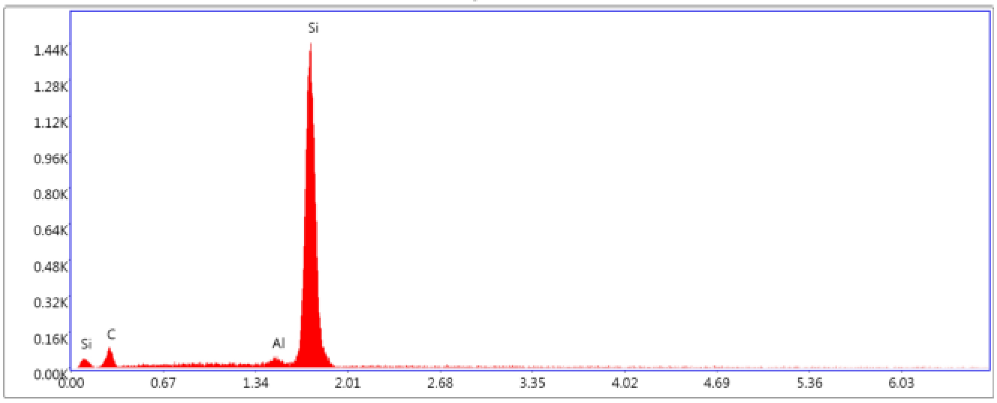

Lsec: 29.8 0 Cnts 0.000 keV Det: Octane Pro A

eZAF Smart Quant Results

Element	Weight %	Atomic %	Net Int.	Error %	Kratio	Z	A	F
C K	20.70	37.88	27.70	15.80	0.0321	1.1428	0.1355	1.0000
AlK	1.94	1.58	21.66	12.62	0.0181	0.9435	0.9757	1.0154
SiK	77.36	60.54	803.53	2.90	0.7352	0.9612	0.9886	1.0002

5% CH_4 in H_2 at 1650°C

Area 1

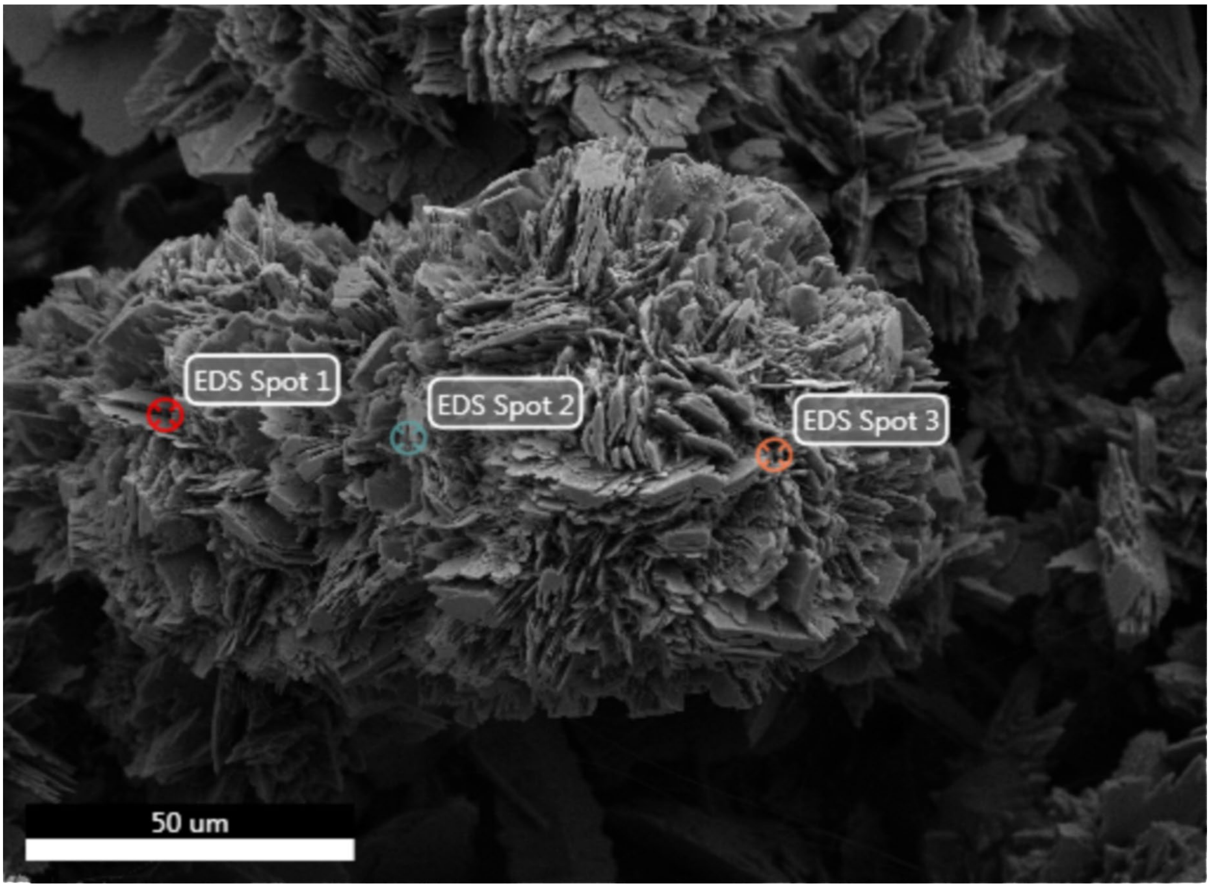

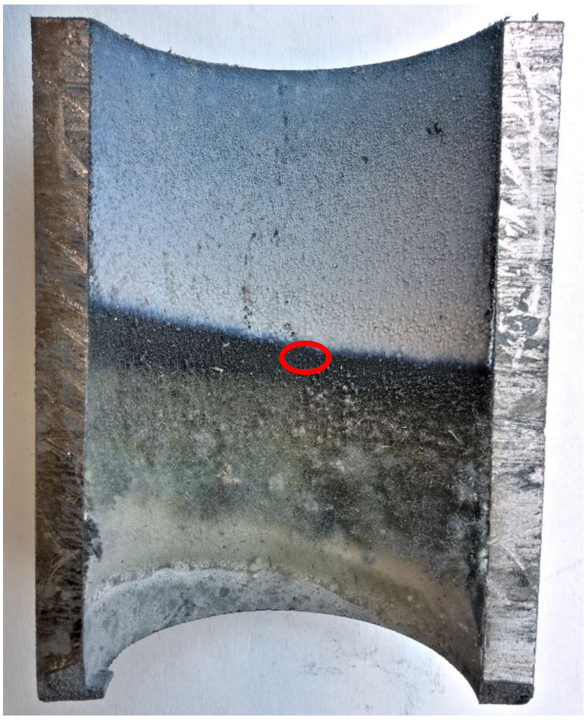

EDS Spot 1

kV: 10	Mag: 600	Takeoff: 30.4	Live Time(s): 30	Amp Time(µs): 1.92	Resolution:(eV) 127.9

EDS Spot 1 - Det 1

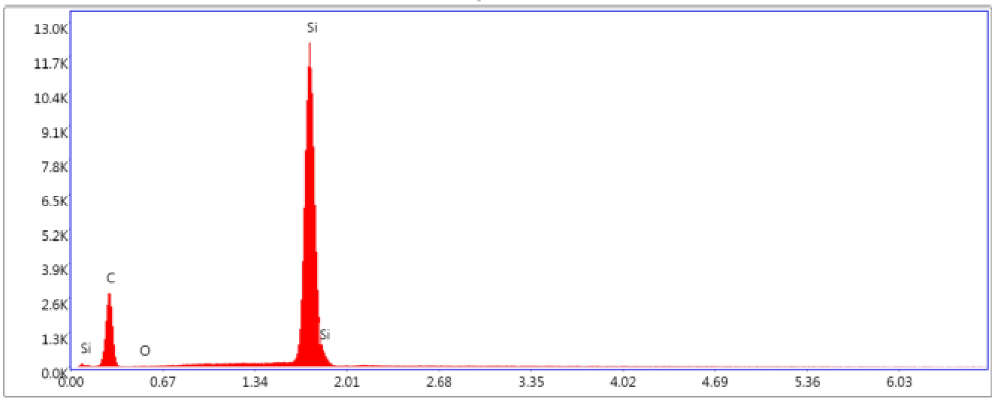

Lsec: 30.0 0 Cnts 0.000 keV Det: Octane Pro A

eZAF Smart Quant Results

Element	Weight %	Atomic %	Net Int.	Error %	Kratio	Z	A	F
C K	47.58	67.97	528.66	10.82	0.0968	1.0890	0.1870	1.0000
O K	0.00	0.00	0.00	99.99	0.0000	1.0294	0.3104	1.0000
SiK	52.42	32.03	3436.74	2.48	0.4754	0.9137	0.9925	1.0005

EDS Spot 2

kV: 10	Mag: 600	Takeoff: 30.4	Live Time(s): 30	Amp Time(µs): 1.92	Resolution:(eV) 127.9

EDS Spot 2 - Det 1

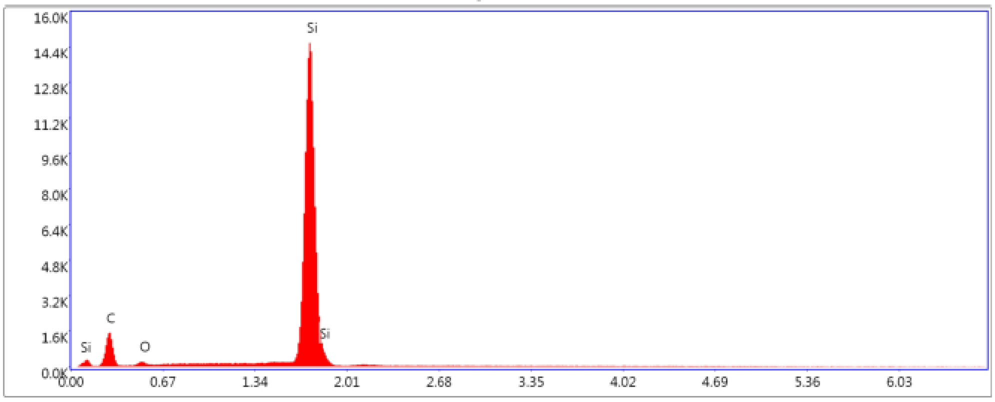

Lsec: 30.0 0 Cnts 0.000 keV Det: Octane Pro A

eZAF Smart Quant Results

Element	Weight %	Atomic %	Net Int.	Error %	Kratio	Z	A	F
C K	33.00	53.47	268.57	11.71	0.0544	1.1167	0.1475	1.0000
O K	0.21	0.26	6.17	69.60	0.0008	1.0562	0.3353	1.0000
SiK	66.79	46.27	4077.66	2.43	0.6233	0.9384	0.9945	1.0003

EDS Spot 3

kV: 10	Mag: 600	Takeoff: 30.4	Live Time(s): 30	Amp Time(µs): 1.92	Resolution:(eV) 127.9

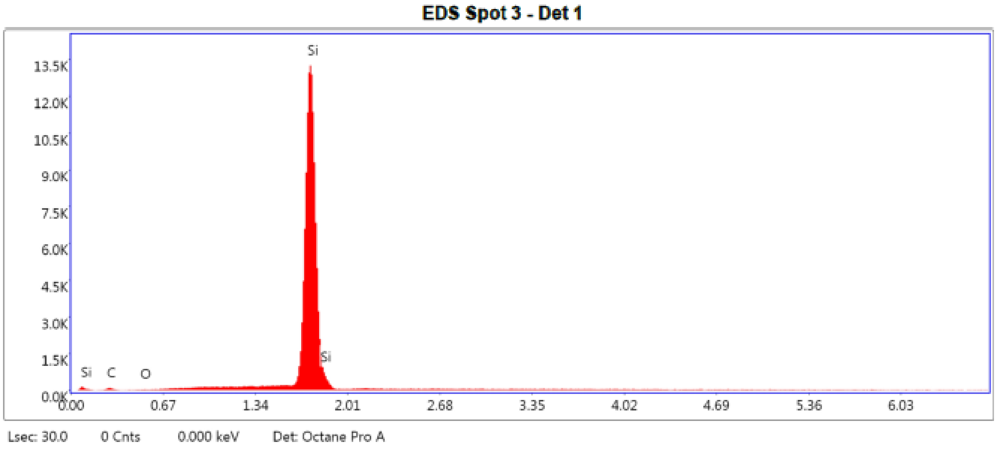

eZAF Smart Quant Results

Element	Weight %	Atomic %	Net Int.	Error %	Kratio	Z	A	F
C K	0.00	0.00	0.00	99.99	0.0000	1.1859	0.0986	1.0000
O K	0.00	0.00	0.00	99.99	0.0000	1.1230	0.4008	1.0000
SiK	100.00	100.00	3998.05	2.37	0.9997	0.9996	1.0001	1.0000

Area 2

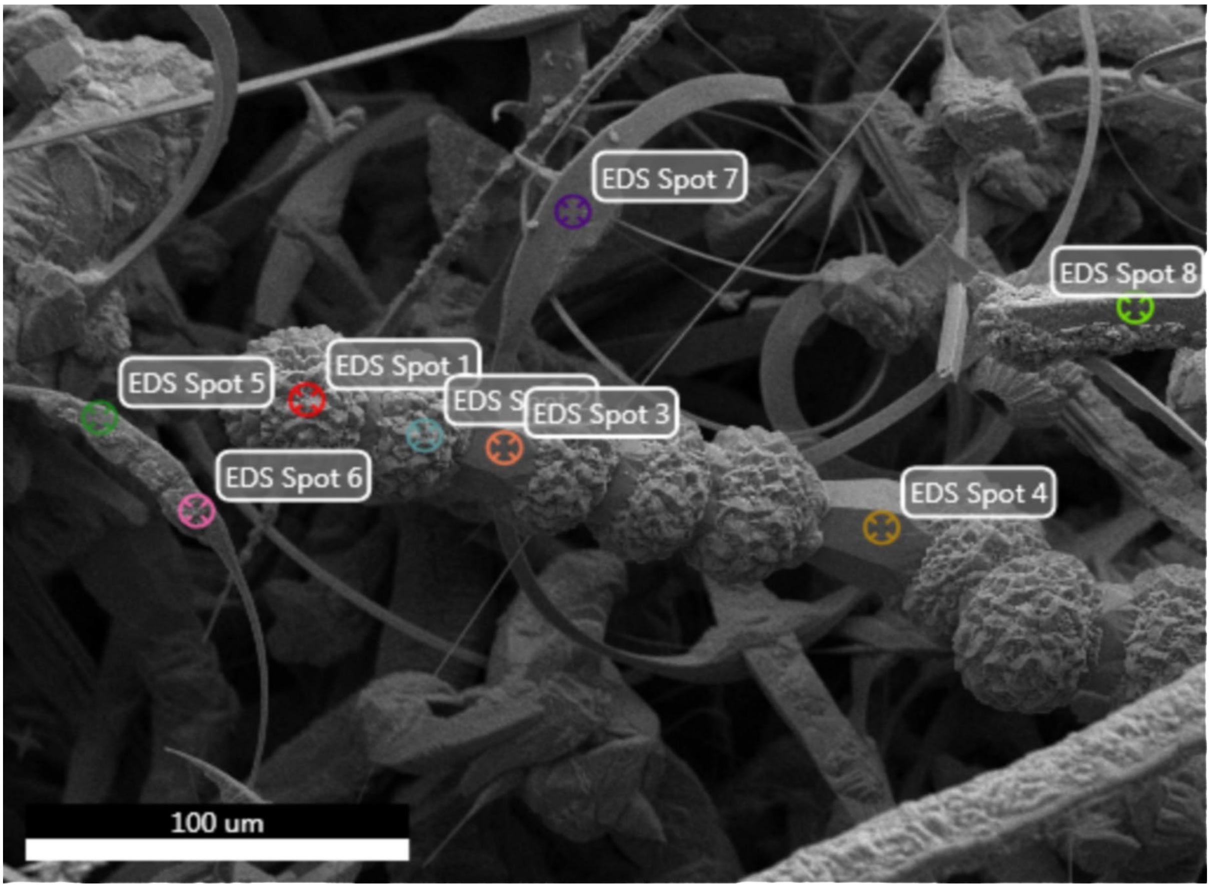
EDS Spot 7
EDS Spot 8
EDS Spot 1
EDS Spot 5
EDS Spot 3
EDS Spot 6
EDS Spot 4
100 um

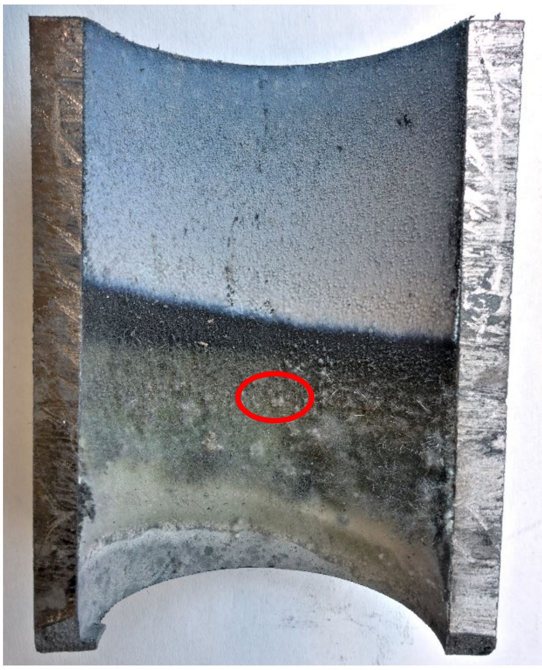

EDS Spot 1

kV: 10	Mag: 350	Takeoff: 29.2	Live Time(s): 30	Amp Time(μs): 1.92	Resolution:(eV) 127.9

EDS Spot 1 - Det 1

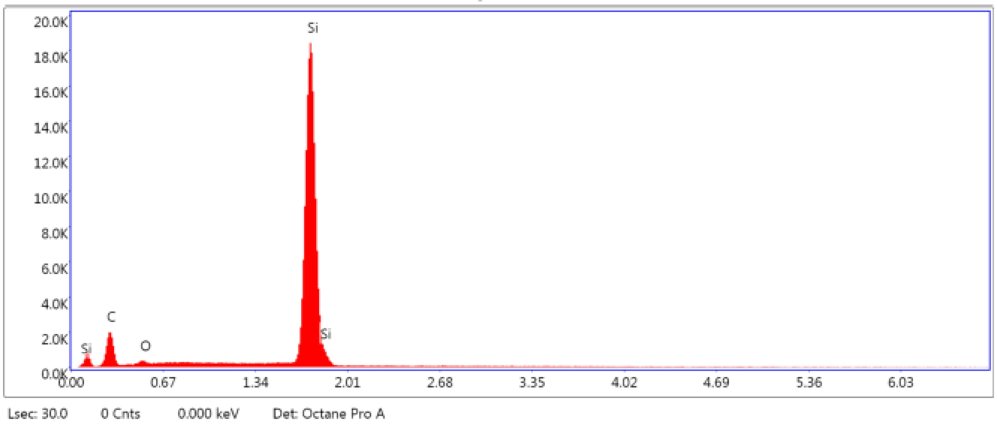

eZAF Smart Quant Results

Element	Weight %	Atomic %	Net Int.	Error %	Kratio	Z	A	F
C K	33.78	54.25	350.36	11.57	0.0550	1.1148	0.1460	1.0000
O K	0.50	0.61	18.33	22.92	0.0017	1.0544	0.3258	1.0000
SiK	65.72	45.14	5244.64	2.41	0.6119	0.9367	0.9938	1.0003

EDS Spot 2

kV: 10	Mag: 350	Takeoff: 29.2	Live Time(s): 30	Amp Time(μs): 1.92	Resolution:(eV) 127.9

EDS Spot 2 - Det 1

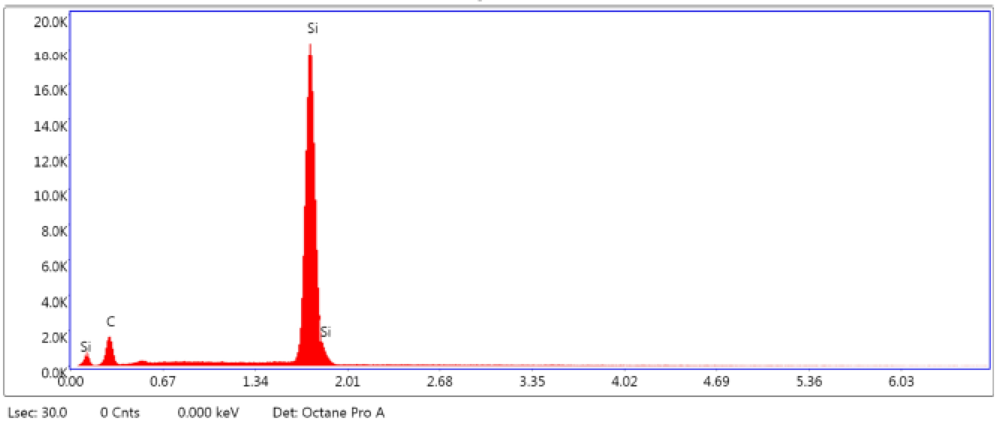

eZAF Smart Quant Results

Element	Weight %	Atomic %	Net Int.	Error %	Kratio	Z	A	F
C K	30.57	50.73	281.24	11.78	0.0475	1.1219	0.1383	1.0000
SiK	69.43	49.27	5193.82	2.40	0.6517	0.9429	0.9950	1.0003

EDS Spot 4

kV: 10	Mag: 350	Takeoff: 29.2	Live Time(s): 30	Amp Time(µs): 1.92	Resolution:(eV) 127.9

EDS Spot 4 - Det 1

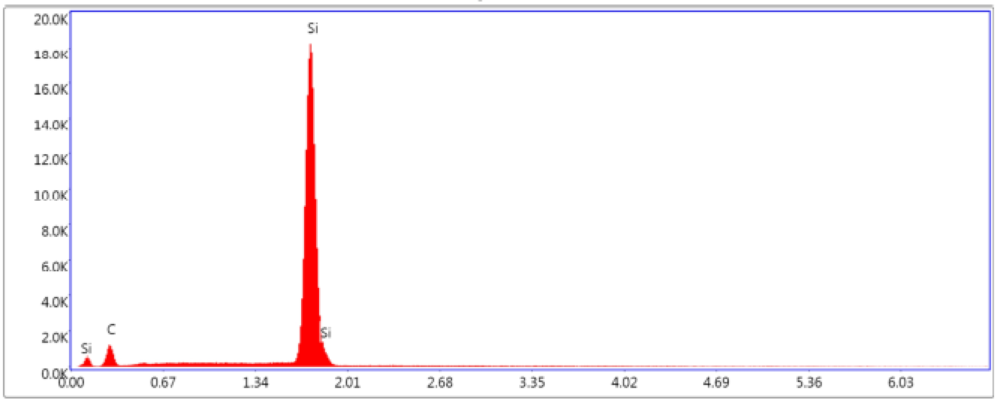

Lsec: 30.0 0 Cnts 0.000 keV Det: Octane Pro A

eZAF Smart Quant Results

Element	Weight %	Atomic %	Net Int.	Error %	Kratio	Z	A	F
C K	23.74	42.13	180.40	12.46	0.0340	1.1355	0.1261	1.0000
SiK	76.26	57.87	5179.83	2.38	0.7255	0.9551	0.9961	1.0002

EDS Spot 5

kV: 10	Mag: 350	Takeoff: 29.2	Live Time(s): 30	Amp Time(µs): 1.92	Resolution:(eV) 127.9

EDS Spot 5 - Det 1

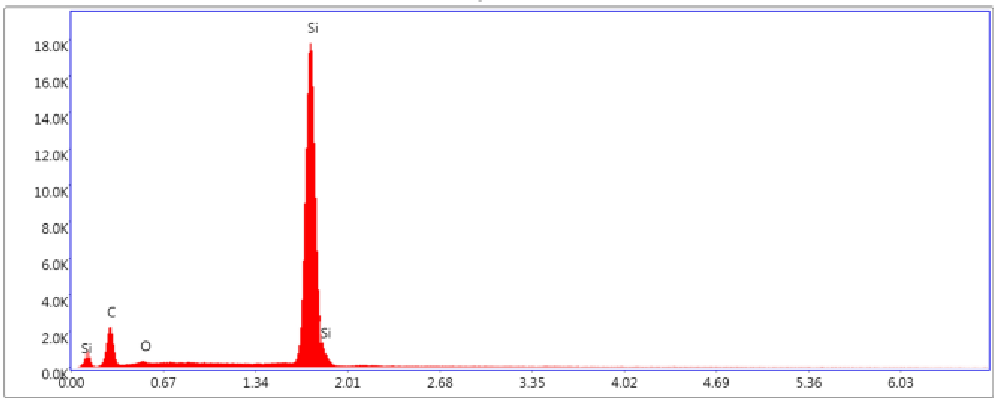

Lsec: 30.0 0 Cnts 0.000 keV Det: Octane Pro A

eZAF Smart Quant Results

Element	Weight %	Atomic %	Net Int.	Error %	Kratio	Z	A	F
C K	36.53	57.24	400.89	11.44	0.0617	1.1095	0.1522	1.0000
O K	0.47	0.56	17.27	23.10	0.0016	1.0492	0.3210	1.0000
SiK	62.99	42.21	5096.39	2.42	0.5832	0.9319	0.9934	1.0003

EDS Spot 6

kV: 10	Mag: 350	Takeoff: 29.2	Live Time(s): 30	Amp Time(μs): 1.92	Resolution:(eV) 127.9

EDS Spot 6 - Det 1

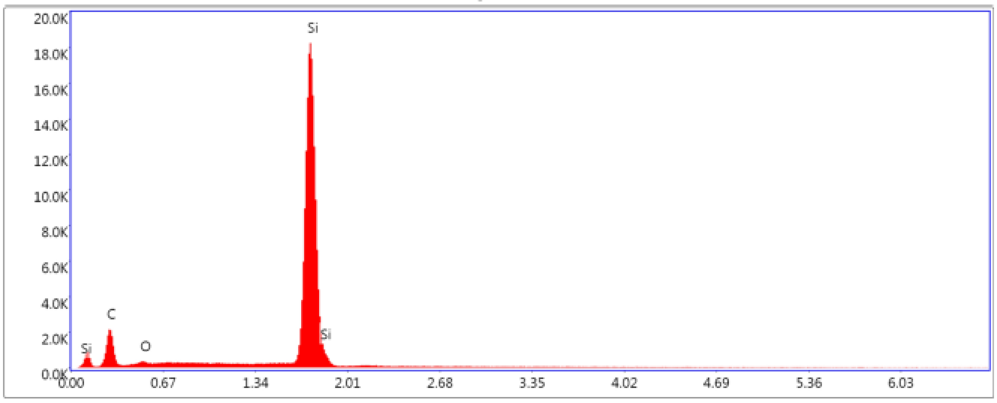

Lsec: 30.0 0 Cnts 0.000 keV Det: Octane Pro A

eZAF Smart Quant Results

Element	Weight %	Atomic %	Net Int.	Error %	Kratio	Z	A	F
C K	35.43	56.05	379.73	11.49	0.0590	1.1115	0.1498	1.0000
O K	0.55	0.65	20.03	22.51	0.0019	1.0512	0.3230	1.0000
SiK	64.02	43.30	5140.43	2.41	0.5939	0.9337	0.9935	1.0003

5% CH_4 in H_2 at 1750°C

Area 1

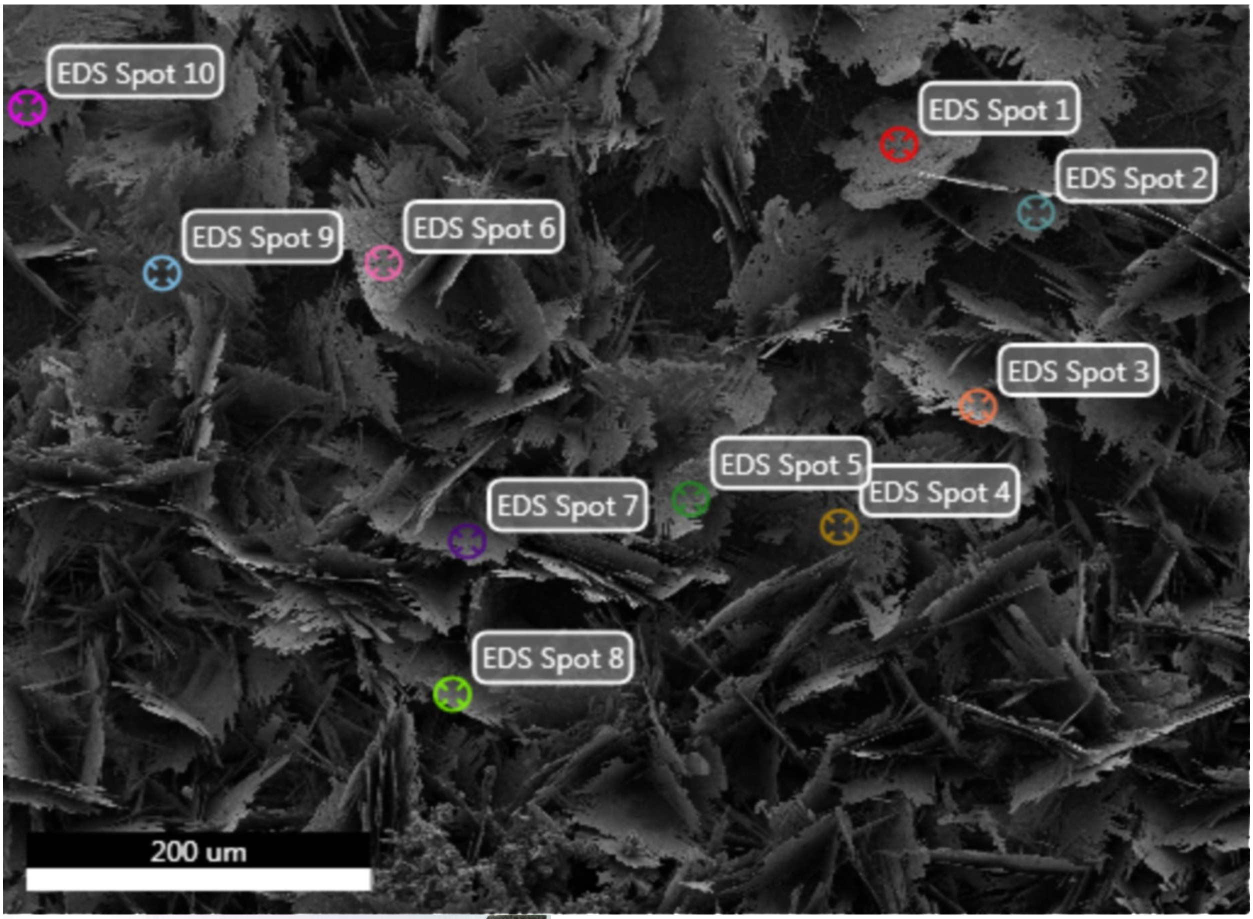

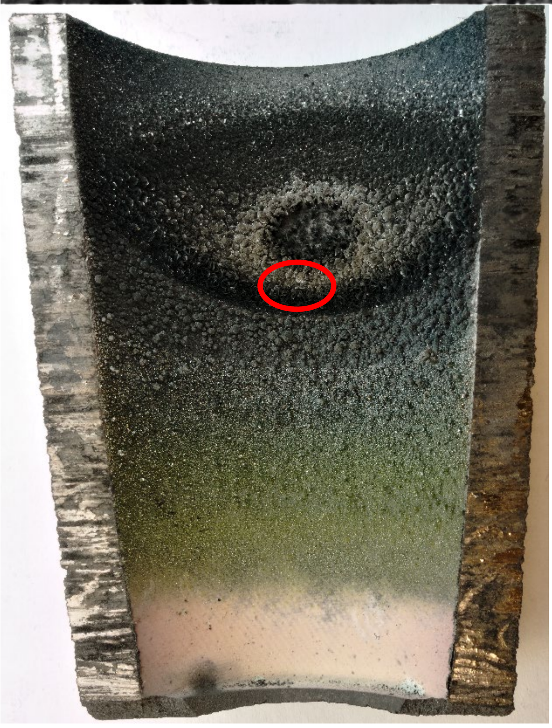

EDS Spot 1

kV: 10	Mag: 152	Takeoff: 40.8	Live Time(s): 29.9	Amp Time(µs): 1.92	Resolution:(eV) 127.4

EDS Spot 1 - Det 1

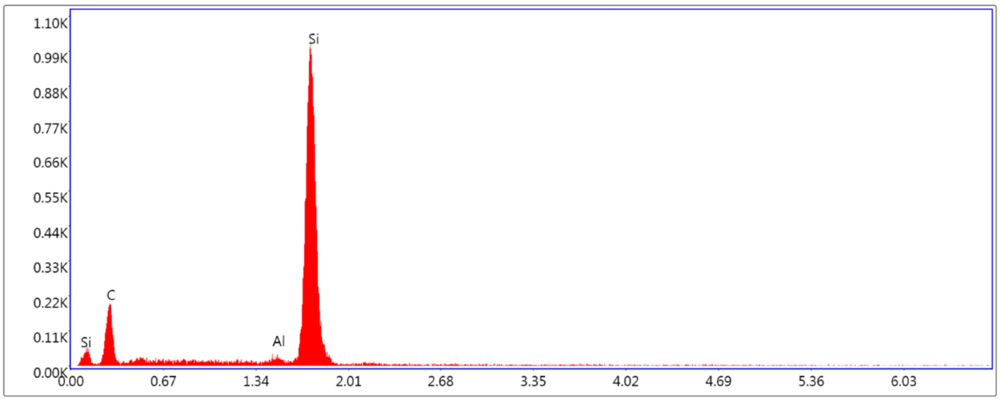

Lsec: 29.9 0 Cnts 0.000 keV Det: Octane Pro A

eZAF Smart Quant Results

Element	Weight %	Atomic %	Net Int.	Error %	Kratio	Z	A	F
C K	39.02	59.92	70.32	12.91	0.0845	1.1060	0.1958	1.0000
AlK	1.03	0.70	10.19	21.03	0.0093	0.9116	0.9744	1.0152
SiK	59.96	39.38	554.37	3.06	0.5519	0.9286	0.9912	1.0004

EDS Spot 2

kV: 10	Mag: 152	Takeoff: 40.8	Live Time(s): 29.8	Amp Time(µs): 1.92	Resolution:(eV) 127.4

EDS Spot 2 - Det 1

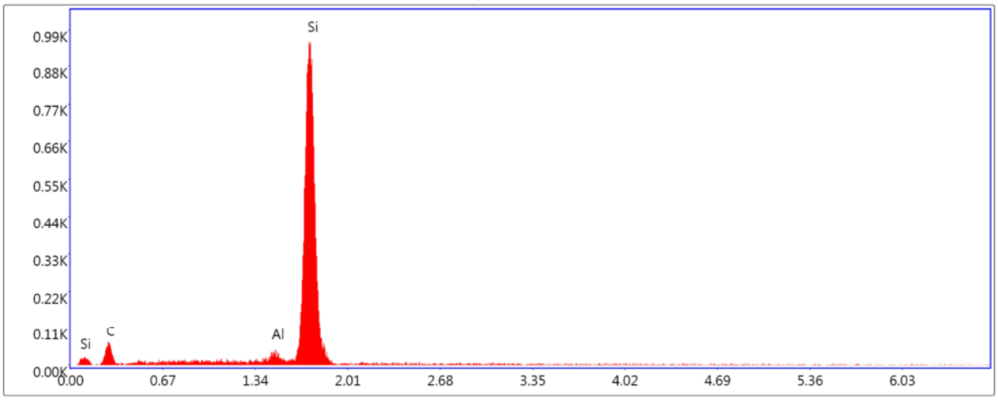

Lsec: 29.8 0 Cnts 0.000 keV Det: Octane Pro A

eZAF Smart Quant Results

Element	Weight %	Atomic %	Net Int.	Error %	Kratio	Z	A	F
C K	19.19	35.67	19.76	17.80	0.0327	1.1461	0.1489	1.0000
AlK	2.67	2.21	19.99	12.49	0.0251	0.9463	0.9792	1.0151
SiK	78.14	62.12	541.65	3.09	0.7441	0.9641	0.9875	1.0001

EDS Spot 3

kV: 10	Mag: 152	Takeoff: 40.8	Live Time(s): 29.9	Amp Time(μs): 1.92	Resolution:(eV) 127.4

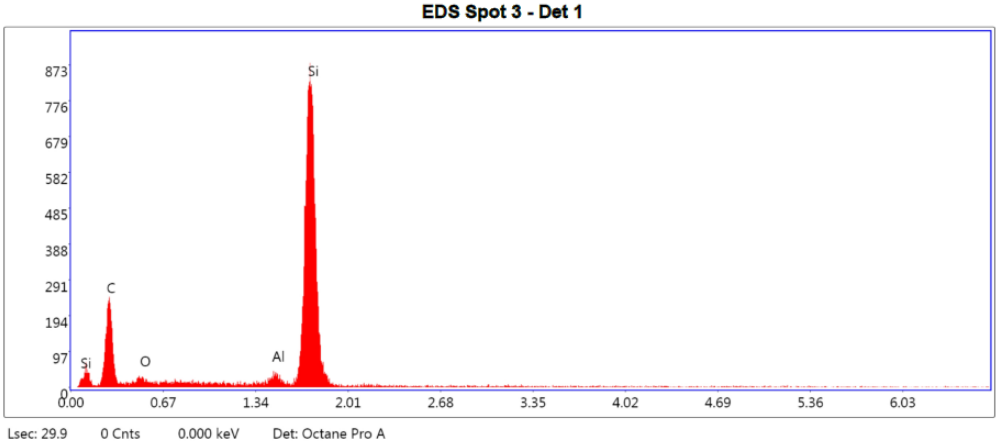

eZAF Smart Quant Results

Element	Weight %	Atomic %	Net Int.	Error %	Kratio	Z	A	F
C K	44.37	64.67	88.99	11.96	0.1060	1.0944	0.2183	1.0000
O K	1.29	1.41	6.74	30.80	0.0051	1.0345	0.3828	1.0000
AlK	1.67	1.08	16.46	12.94	0.0148	0.9015	0.9716	1.0144
SiK	52.67	32.83	483.88	3.14	0.4778	0.9183	0.9870	1.0005

EDS Spot 4

kV: 10	Mag: 152	Takeoff: 40.8	Live Time(s): 29.8	Amp Time(μs): 1.92	Resolution:(eV) 127.4

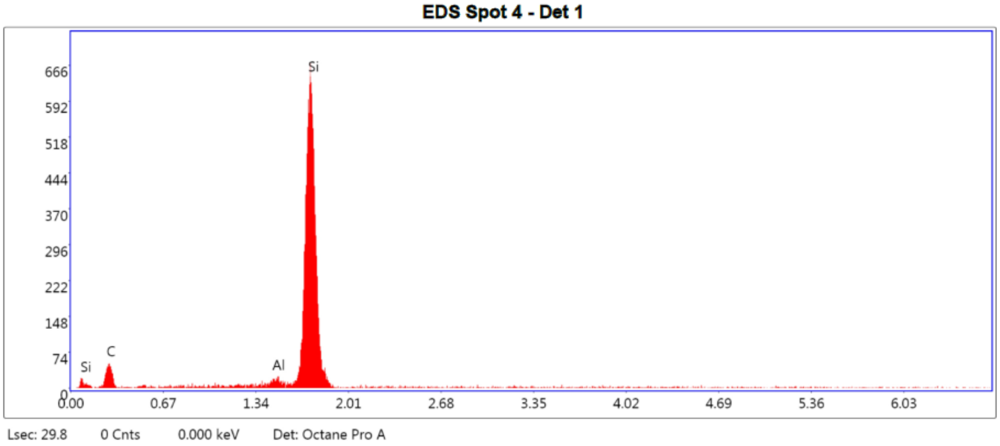

eZAF Smart Quant Results

Element	Weight %	Atomic %	Net Int.	Error %	Kratio	Z	A	F
C K	23.08	41.20	17.40	16.45	0.0411	1.1380	0.1564	1.0000
AlK	2.47	1.96	12.88	13.40	0.0230	0.9394	0.9783	1.0151
SiK	74.45	56.84	359.83	3.26	0.7038	0.9570	0.9878	1.0002

EDS Spot 5

kV: 10	Mag: 152	Takeoff: 40.8	Live Time(s): 29.9	Amp Time(μs): 1.92	Resolution:(eV) 127.4

EDS Spot 5 - Det 1

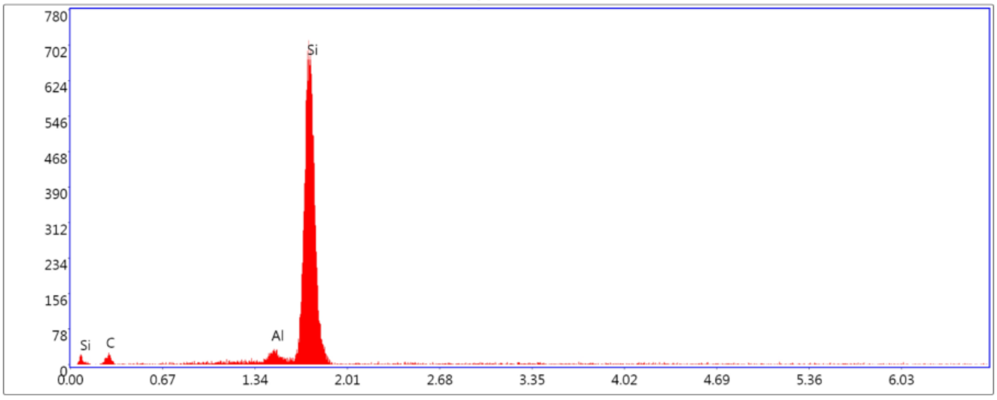

Lsec: 29.9 0 Cnts 0.000 keV Det: Octane Pro A

eZAF Smart Quant Results

Element	Weight %	Atomic %	Net Int.	Error %	Kratio	Z	A	F
C K	9.76	20.15	6.11	24.19	0.0152	1.1663	0.1336	1.0000
AlK	4.28	3.93	21.80	9.35	0.0411	0.9638	0.9817	1.0147
SiK	85.96	75.91	402.19	3.25	0.8295	0.9820	0.9827	1.0001

EDS Spot 6

kV: 10	Mag: 152	Takeoff: 40.8	Live Time(s): 29.9	Amp Time(μs): 1.92	Resolution:(eV) 127.4

EDS Spot 6 - Det 1

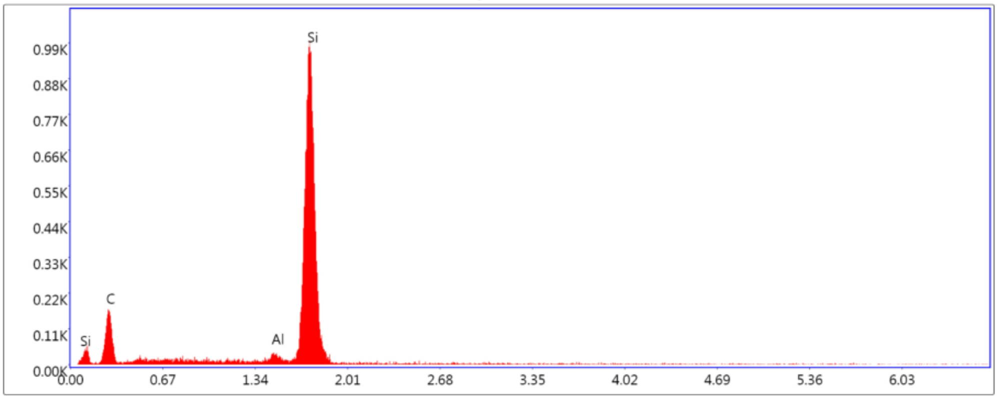

Lsec: 29.9 0 Cnts 0.000 keV Det: Octane Pro A

eZAF Smart Quant Results

Element	Weight %	Atomic %	Net Int.	Error %	Kratio	Z	A	F
C K	36.21	57.01	59.17	13.19	0.0756	1.1116	0.1878	1.0000
AlK	1.68	1.17	15.73	13.68	0.0152	0.9164	0.9752	1.0149
SiK	62.11	41.82	541.94	3.08	0.5736	0.9336	0.9891	1.0003

EDS Spot 7

kV: 10	Mag: 152	Takeoff: 40.8	Live Time(s): 29.8	Amp Time(μs): 1.92	Resolution:(eV) 127.4

EDS Spot 7 - Det 1

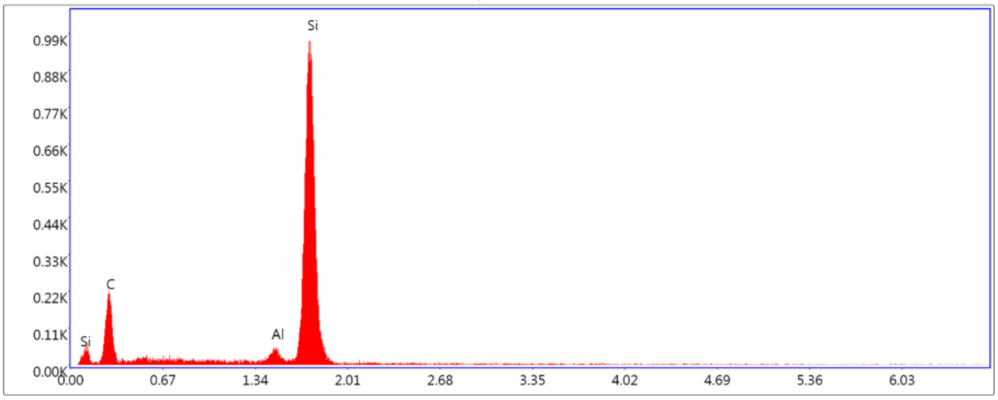

Lsec: 29.8 0 Cnts 0.000 keV Det: Octane Pro A

eZAF Smart Quant Results

Element	Weight %	Atomic %	Net Int.	Error %	Kratio	Z	A	F
C K	41.49	62.34	81.52	12.21	0.0934	1.1016	0.2043	1.0000
AlK	2.31	1.55	23.91	10.48	0.0207	0.9077	0.9741	1.0144
SiK	56.20	36.12	540.03	3.12	0.5128	0.9247	0.9861	1.0004

EDS Spot 8

kV: 10	Mag: 152	Takeoff: 40.8	Live Time(s): 29.8	Amp Time(μs): 1.92	Resolution:(eV) 127.4

EDS Spot 8 - Det 1

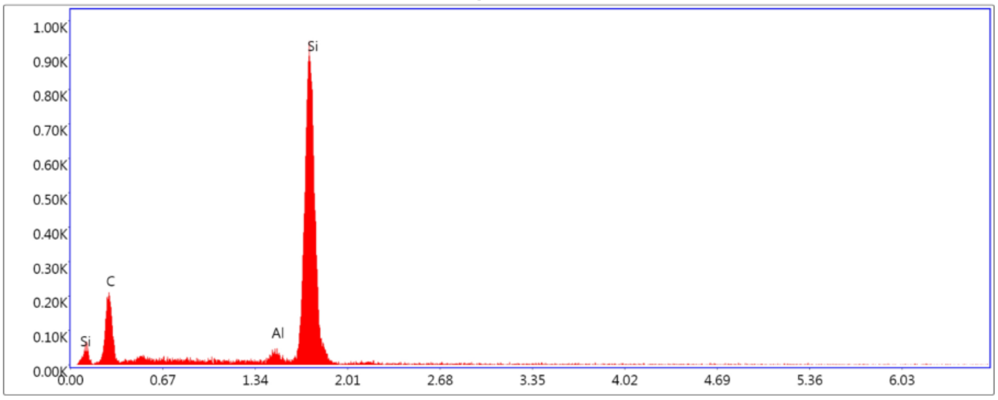

Lsec: 29.8 0 Cnts 0.000 keV Det: Octane Pro A

eZAF Smart Quant Results

Element	Weight %	Atomic %	Net Int.	Error %	Kratio	Z	A	F
C K	41.57	62.43	76.69	12.47	0.0936	1.1013	0.2043	1.0000
AlK	1.73	1.16	16.82	13.40	0.0155	0.9075	0.9740	1.0147
SiK	56.69	36.41	512.71	3.14	0.5183	0.9244	0.9883	1.0004

EDS Spot 9

kV: 10	Mag: 152	Takeoff: 40.8	Live Time(s): 30	Amp Time(μs): 1.92	Resolution:(eV) 127.4

EDS Spot 9 - Det 1

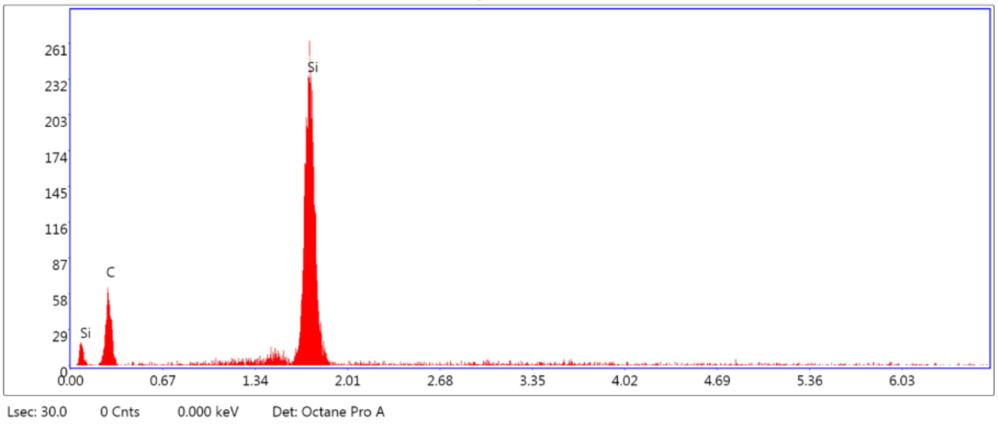

eZAF Smart Quant Results

Element	Weight %	Atomic %	Net Int.	Error %	Kratio	Z	A	F
C K	41.59	62.48	20.08	14.81	0.0931	1.1009	0.2033	1.0000
SiK	58.41	37.52	139.78	3.90	0.5373	0.9241	0.9948	1.0004

EDS Spot 10

kV: 10	Mag: 152	Takeoff: 40.8	Live Time(s): 29.9	Amp Time(μs): 1.92	Resolution:(eV) 127.4

EDS Spot 10 - Det 1

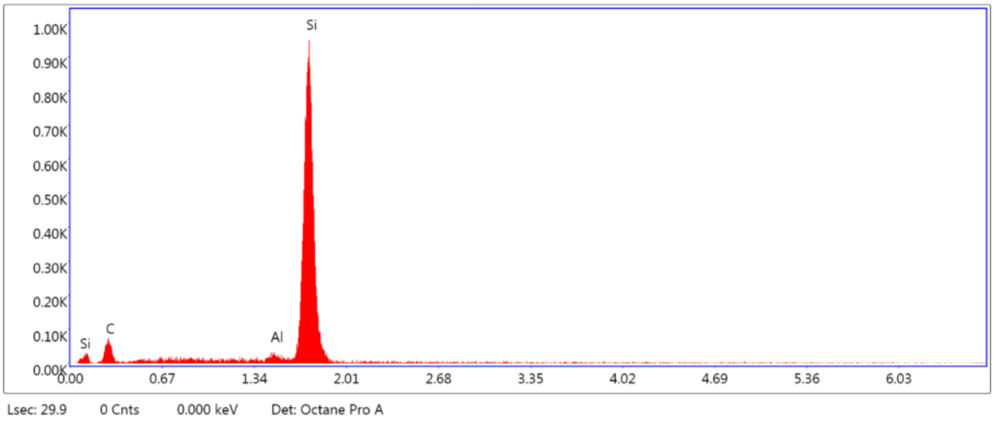

eZAF Smart Quant Results

Element	Weight %	Atomic %	Net Int.	Error %	Kratio	Z	A	F
C K	22.02	39.75	22.05	16.72	0.0387	1.1400	0.1540	1.0000
AlK	1.75	1.40	12.30	17.01	0.0163	0.9411	0.9784	1.0155
SiK	76.23	58.85	498.36	3.09	0.7241	0.9588	0.9906	1.0002

Area 2

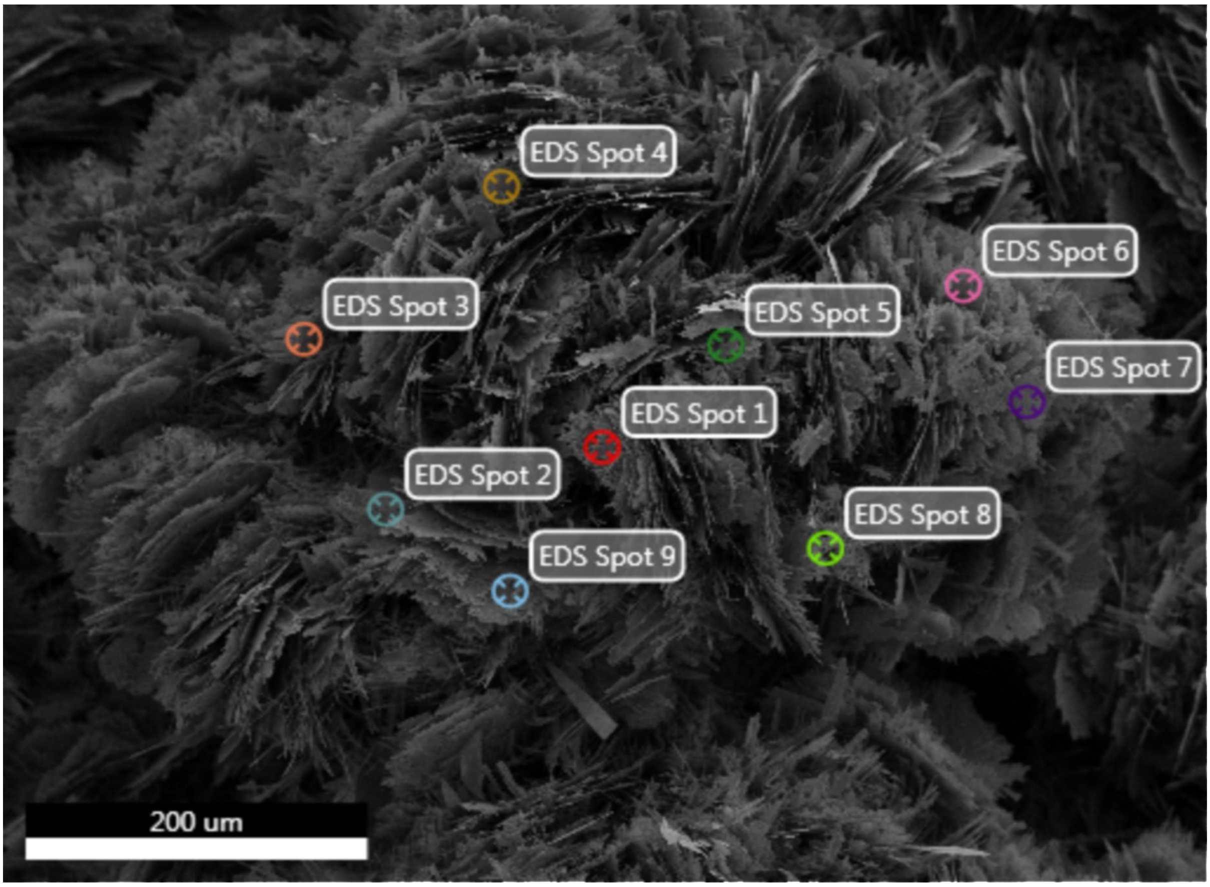
EDS Spot 4
EDS Spot 6
EDS Spot 3
EDS Spot 5
EDS Spot 7
EDS Spot 1
EDS Spot 2
EDS Spot 8
EDS Spot 9
200 um

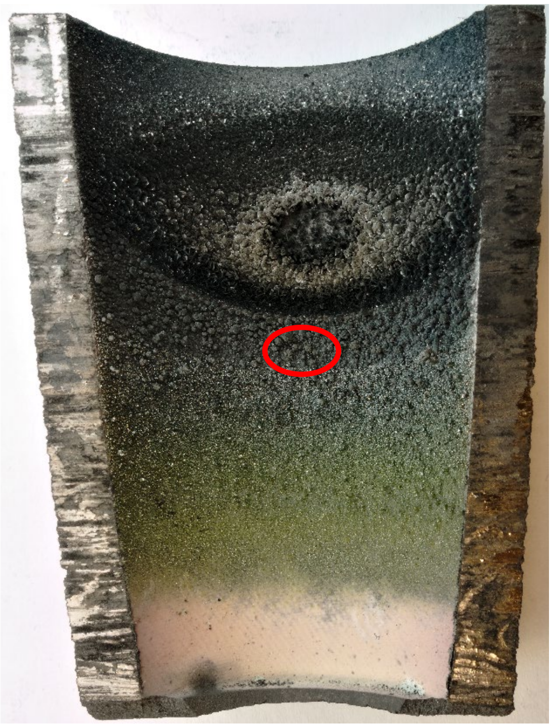

EDS Spot 1

kV: 10	Mag: 156	Takeoff: 39.5	Live Time(s): 29.9	Amp Time(μs): 1.92	Resolution:(eV) 127.4

EDS Spot 1 - Det 1

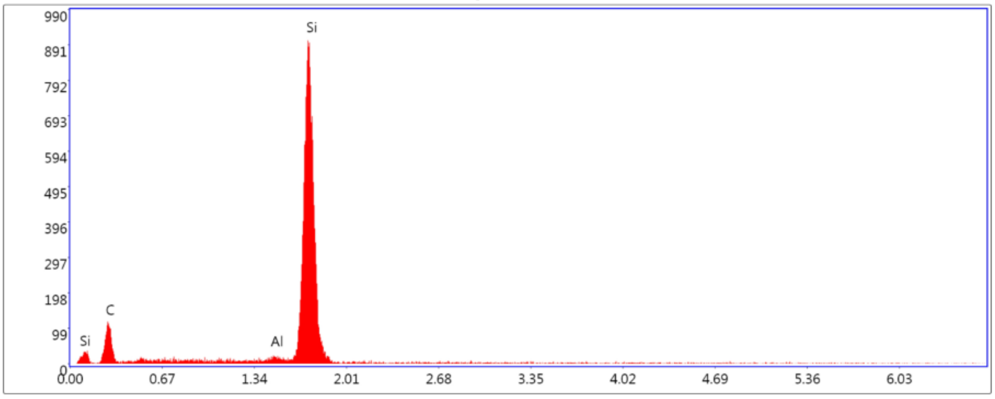

Lsec: 29.9 1 Cnts 5.390 keV Det: Octane Pro A

eZAF Smart Quant Results

Element	Weight %	Atomic %	Net Int.	Error %	Kratio	Z	A	F
C K	31.78	52.12	40.07	13.90	0.0612	1.1203	0.1719	1.0000
AlK	1.33	0.97	10.64	16.73	0.0122	0.9241	0.9755	1.0153
SiK	66.89	46.91	496.65	3.08	0.6241	0.9414	0.9907	1.0003

EDS Spot 2

kV: 10	Mag: 156	Takeoff: 39.5	Live Time(s): 29.9	Amp Time(μs): 1.92	Resolution:(eV) 127.4

EDS Spot 2 - Det 1

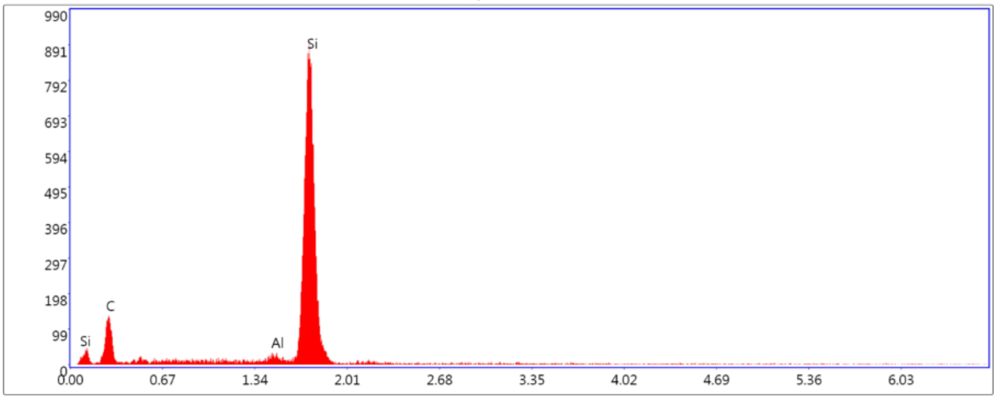

Lsec: 29.9 0 Cnts 5.390 keV Det: Octane Pro A

eZAF Smart Quant Results

Element	Weight %	Atomic %	Net Int.	Error %	Kratio	Z	A	F
C K	34.46	55.12	46.77	13.60	0.0688	1.1150	0.1790	1.0000
AlK	1.53	1.09	12.56	16.43	0.0139	0.9194	0.9749	1.0151
SiK	64.01	43.79	490.72	3.12	0.5934	0.9366	0.9896	1.0003

EDS Spot 3

kV: 10	Mag: 156	Takeoff: 39.5	Live Time(s): 29.9	Amp Time(μs): 1.92	Resolution:(eV) 127.4

EDS Spot 3 - Det 1

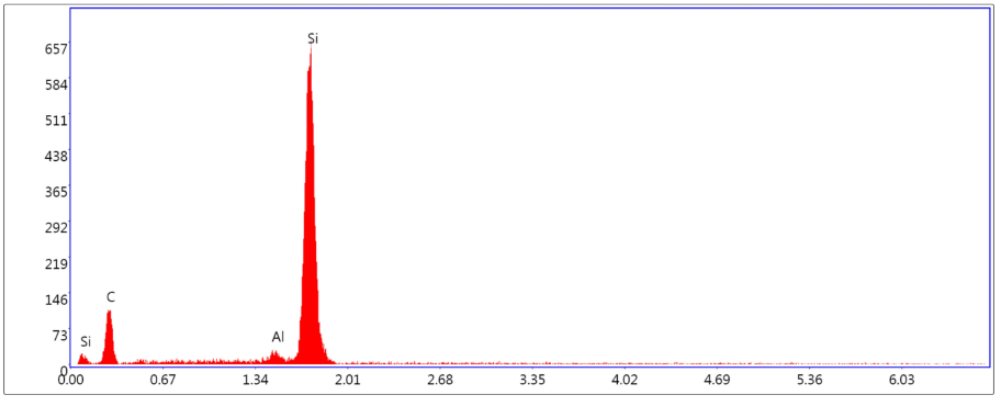

Lsec: 29.9 0 Cnts 5.390 keV Det: Octane Pro A

eZAF Smart Quant Results

Element	Weight %	Atomic %	Net Int.	Error %	Kratio	Z	A	F
C K	38.06	58.93	42.77	13.60	0.0799	1.1081	0.1896	1.0000
AlK	2.36	1.62	15.13	12.23	0.0213	0.9134	0.9742	1.0145
SiK	59.58	39.45	355.59	3.31	0.5467	0.9305	0.9860	1.0004

EDS Spot 4

kV: 10	Mag: 156	Takeoff: 39.5	Live Time(s): 29.8	Amp Time(μs): 1.92	Resolution:(eV) 127.4

EDS Spot 4 - Det 1

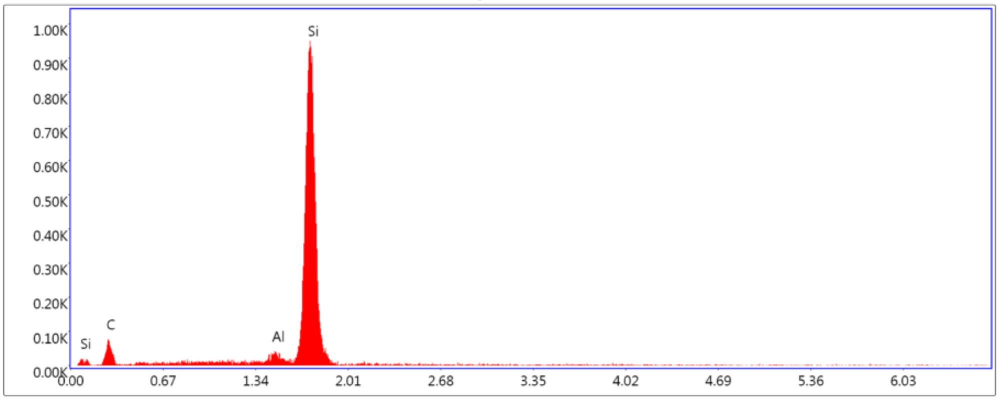

Lsec: 29.8 0 Cnts 5.390 keV Det: Octane Pro A

eZAF Smart Quant Results

Element	Weight %	Atomic %	Net Int.	Error %	Kratio	Z	A	F
C K	21.18	38.55	21.76	16.13	0.0362	1.1420	0.1495	1.0000
AlK	2.81	2.28	21.10	11.14	0.0263	0.9428	0.9782	1.0150
SiK	76.01	59.17	526.98	3.11	0.7202	0.9605	0.9864	1.0002

EDS Spot 5

kV: 10	Mag: 156	Takeoff: 39.5	Live Time(s): 29.9	Amp Time(μs): 1.92	Resolution:(eV) 127.4

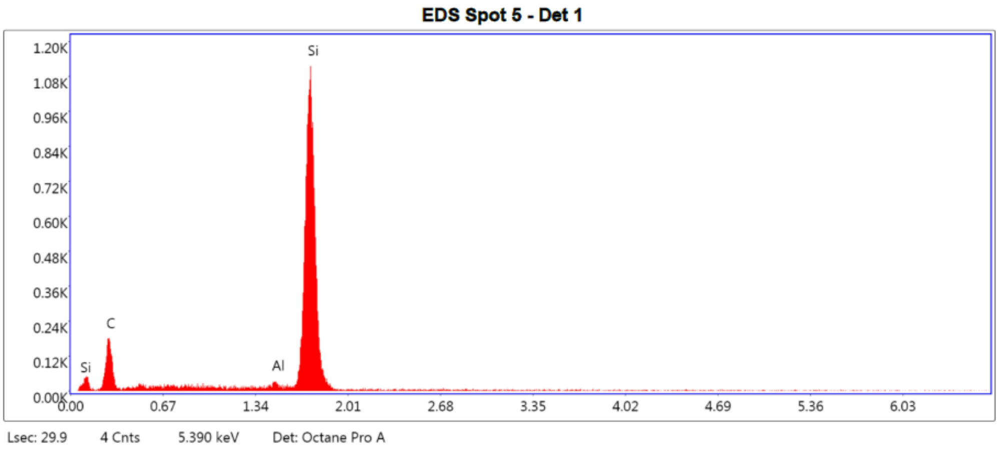

eZAF Smart Quant Results

Element	Weight %	Atomic %	Net Int.	Error %	Kratio	Z	A	F
C K	36.10	56.89	62.45	13.15	0.0735	1.1117	0.1833	1.0000
AlK	1.18	0.83	12.13	18.49	0.0107	0.9166	0.9745	1.0152
SiK	62.72	42.28	599.00	3.01	0.5802	0.9337	0.9908	1.0003

EDS Spot 6

kV: 10	Mag: 156	Takeoff: 39.5	Live Time(s): 29.9	Amp Time(μs): 1.92	Resolution:(eV) 127.4

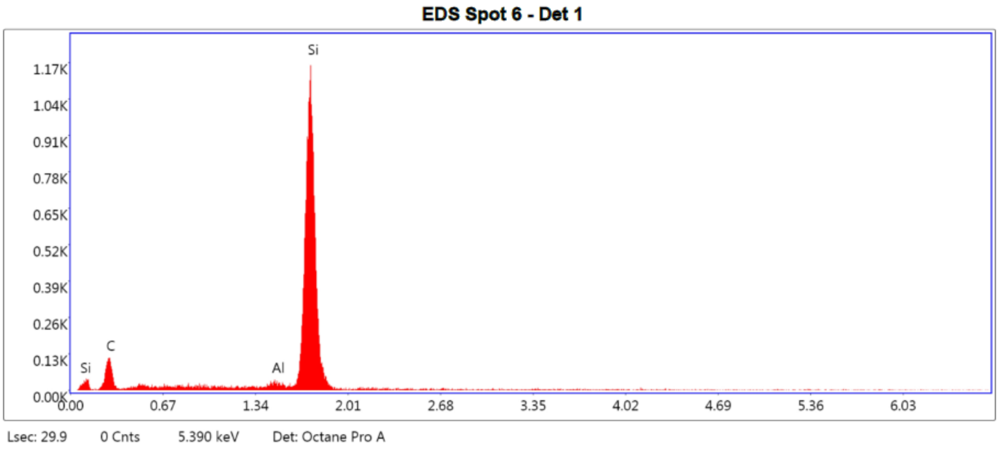

eZAF Smart Quant Results

Element	Weight %	Atomic %	Net Int.	Error %	Kratio	Z	A	F
C K	28.22	47.88	39.21	14.43	0.0520	1.1274	0.1636	1.0000
AlK	1.42	1.07	13.12	16.90	0.0131	0.9302	0.9764	1.0154
SiK	70.36	51.05	605.37	3.02	0.6609	0.9476	0.9908	1.0002

EDS Spot 7

kV: 10	Mag: 156	Takeoff: 39.5	Live Time(s): 29.8	Amp Time(µs): 1.92	Resolution:(eV) 127.4

EDS Spot 7 - Det 1

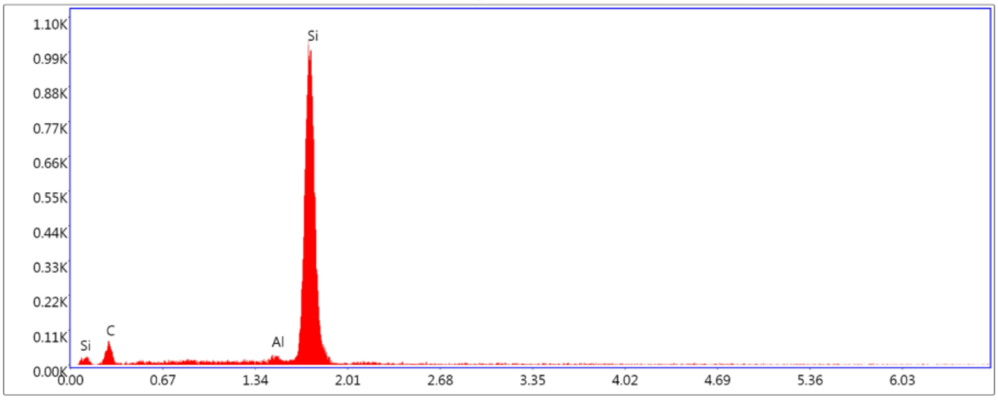

Lsec: 29.8 0 Cnts 5.390 keV Det: Octane Pro A

eZAF Smart Quant Results

Element	Weight %	Atomic %	Net Int.	Error %	Kratio	Z	A	F
C K	20.35	37.38	21.86	16.21	0.0343	1.1434	0.1476	1.0000
AlK	1.73	1.42	13.76	15.81	0.0162	0.9441	0.9783	1.0156
SiK	77.91	61.20	574.45	3.02	0.7425	0.9618	0.9906	1.0002

EDS Spot 8

kV: 10	Mag: 156	Takeoff: 39.5	Live Time(s): 29.8	Amp Time(µs): 1.92	Resolution:(eV) 127.4

EDS Spot 8 - Det 1

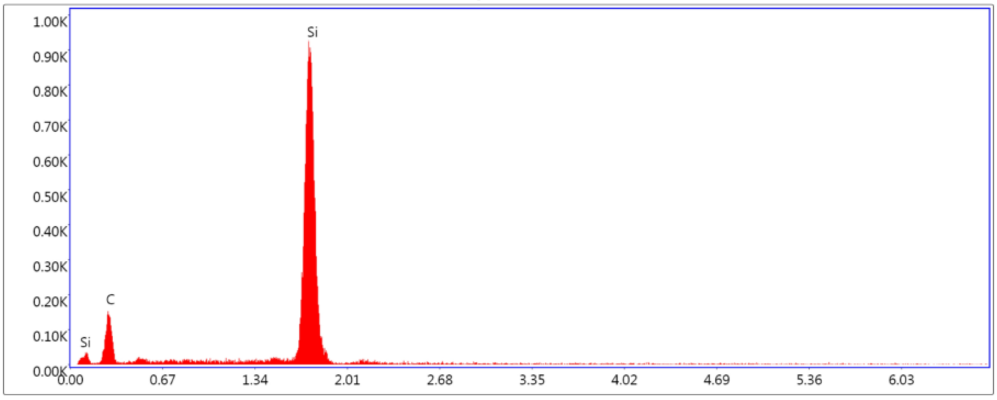

Lsec: 29.8 1 Cnts 5.390 keV Det: Octane Pro A

eZAF Smart Quant Results

Element	Weight %	Atomic %	Net Int.	Error %	Kratio	Z	A	F
C K	36.06	56.88	52.17	13.42	0.0732	1.1115	0.1826	1.0000
SiK	63.94	43.12	514.87	3.04	0.5941	0.9336	0.9953	1.0003

EDS Spot 9

kV: 10	Mag: 156	Takeoff: 39.5	Live Time(s): 29.9	Amp Time(μs): 1.92	Resolution:(eV) 127.4

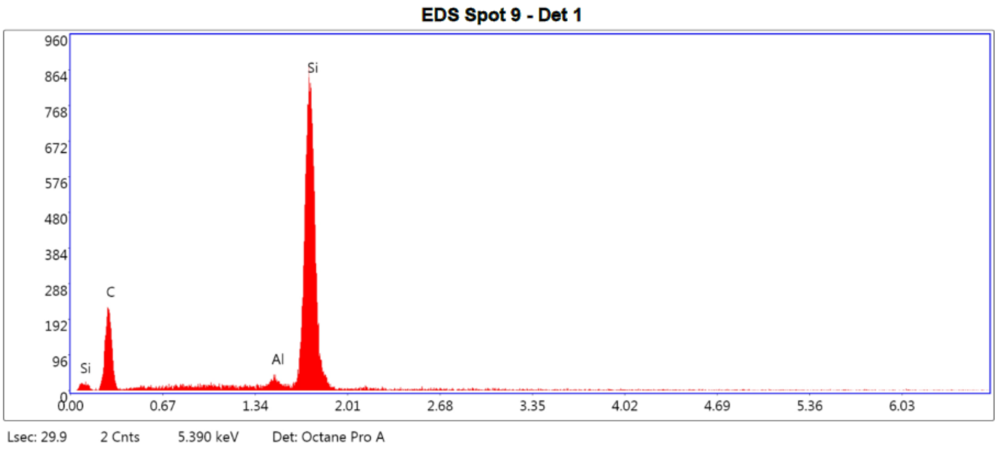

eZAF Smart Quant Results

Element	Weight %	Atomic %	Net Int.	Error %	Kratio	Z	A	F
C K	44.59	65.27	82.26	12.32	0.1028	1.0956	0.2104	1.0000
AlK	1.64	1.07	15.57	13.84	0.0146	0.9025	0.9726	1.0146
SiK	53.77	33.66	475.44	3.15	0.4888	0.9193	0.9880	1.0005

Area 3

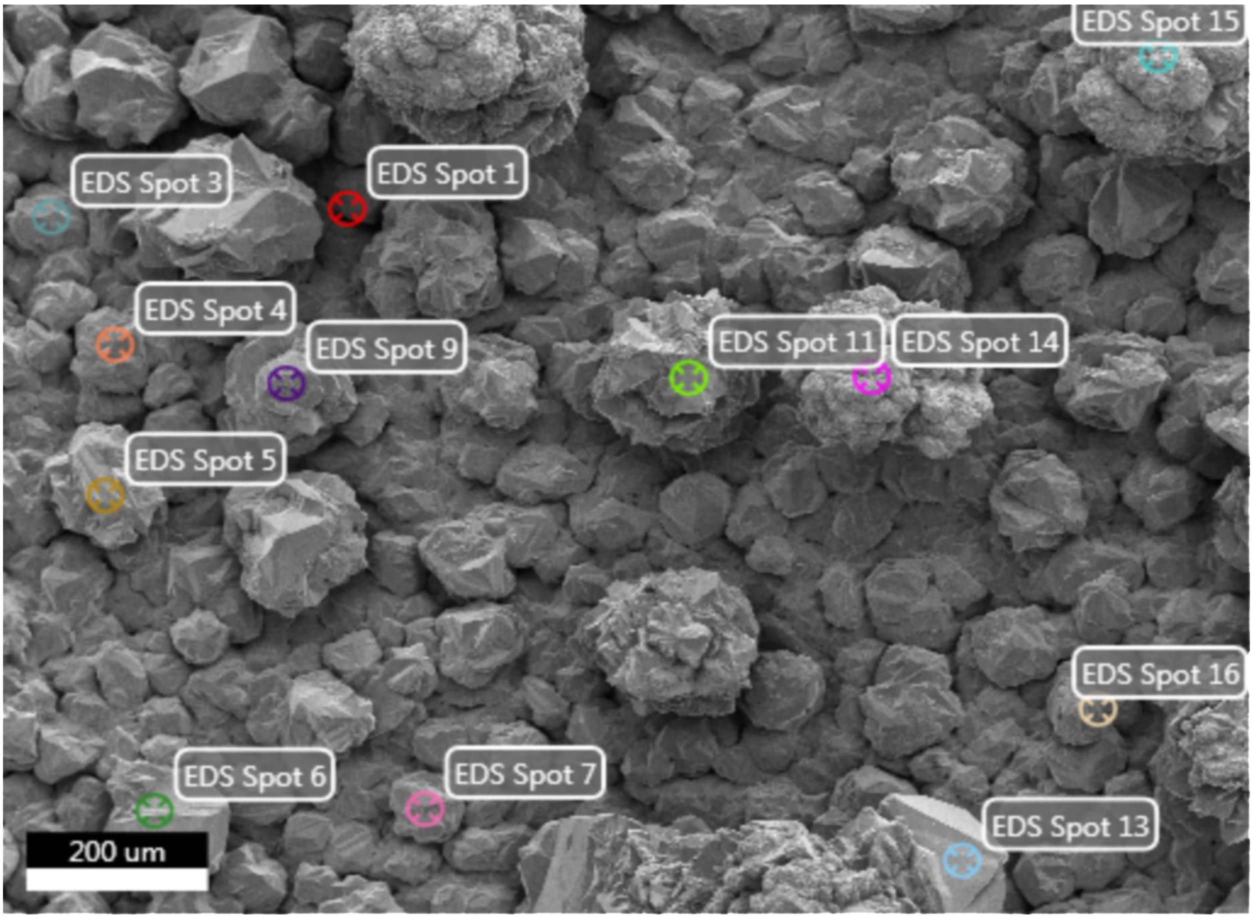
EDS Spot 15
EDS Spot 3
EDS Spot 1
EDS Spot 4
EDS Spot 9
EDS Spot 11
EDS Spot 14
EDS Spot 5
EDS Spot 16
EDS Spot 6
EDS Spot 7
EDS Spot 13
200 um

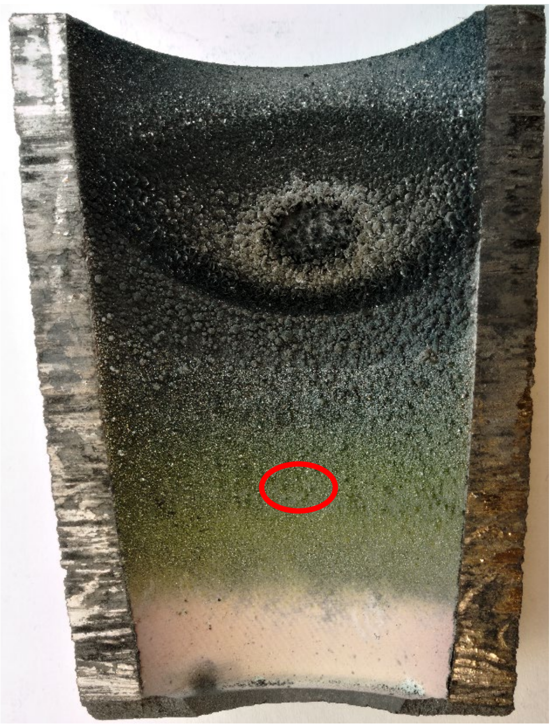

EDS Spot 1

kV: 10	Mag: 80	Takeoff: 40.3	Live Time(s): 30	Amp Time(µs): 1.92	Resolution:(eV) 127.4

EDS Spot 1 - Det 1

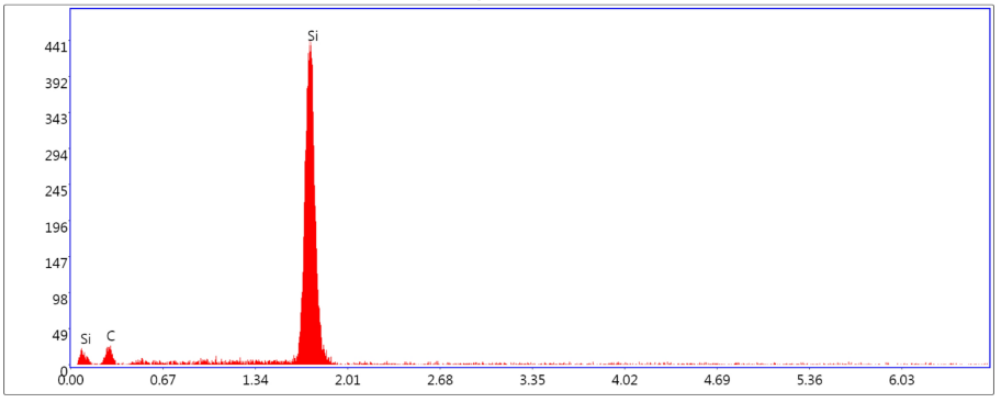

Lsec: 30.0 1 Cnts 5.390 keV Det: Octane Pro A

eZAF Smart Quant Results

Element	Weight %	Atomic %	Net Int.	Error %	Kratio	Z	A	F
C K	19.15	35.65	8.53	20.09	0.0322	1.1456	0.1468	1.0000
SiK	80.85	64.35	249.05	3.39	0.7773	0.9638	0.9975	1.0001

EDS Spot 3

kV: 10	Mag: 80	Takeoff: 40.3	Live Time(s): 29.8	Amp Time(µs): 1.92	Resolution:(eV) 127.4

EDS Spot 3 - Det 1

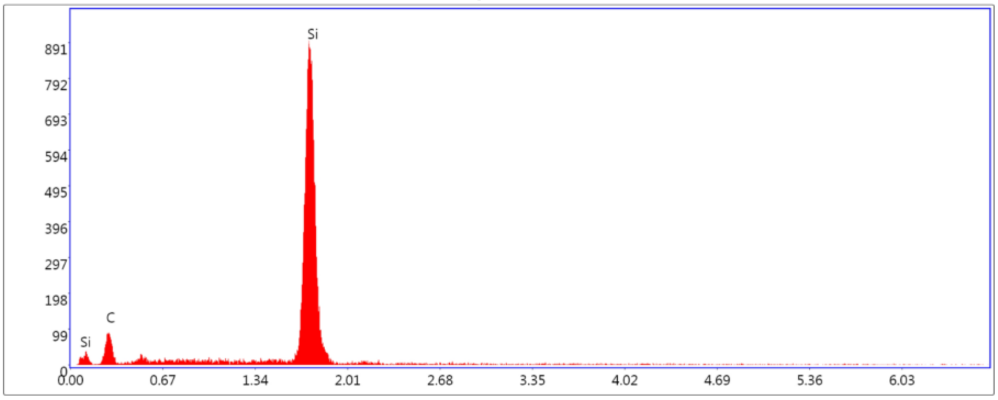

Lsec: 29.8 0 Cnts 5.390 keV Det: Octane Pro A

eZAF Smart Quant Results

Element	Weight %	Atomic %	Net Int.	Error %	Kratio	Z	A	F
C K	27.33	46.79	30.31	15.32	0.0503	1.1289	0.1631	1.0000
SiK	72.67	53.21	500.87	3.04	0.6875	0.9490	0.9965	1.0002

EDS Spot 4

kV: 10 | Mag: 80 | Takeoff: 40.3 | Live Time(s): 29.9 | Amp Time(μs): 1.92 | Resolution:(eV) 127.4

EDS Spot 4 - Det 1

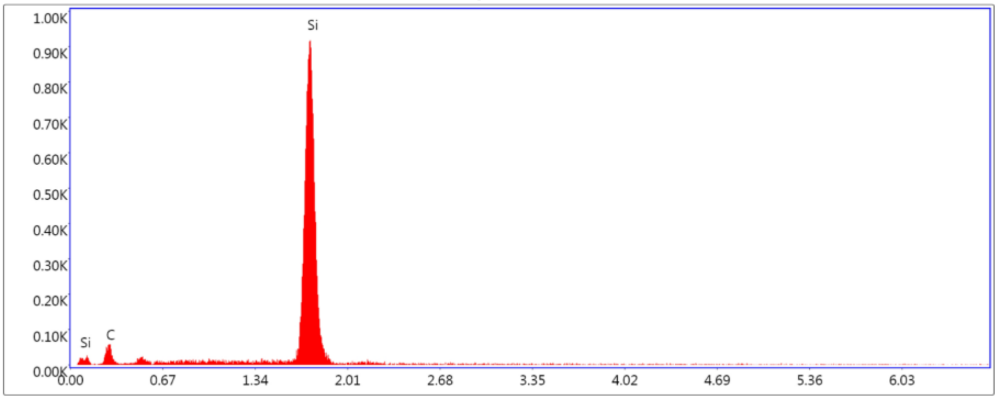

Lsec: 29.9 0 Cnts 5.390 keV Det: Octane Pro A

eZAF Smart Quant Results

Element	Weight %	Atomic %	Net Int.	Error %	Kratio	Z	A	F
C K	17.65	33.39	15.47	18.63	0.0292	1.1487	0.1441	1.0000
SiK	82.35	66.61	508.74	3.01	0.7944	0.9666	0.9977	1.0001

EDS Spot 5

kV: 10 | Mag: 80 | Takeoff: 40.3 | Live Time(s): 29.8 | Amp Time(μs): 1.92 | Resolution:(eV) 127.4

EDS Spot 5 - Det 1

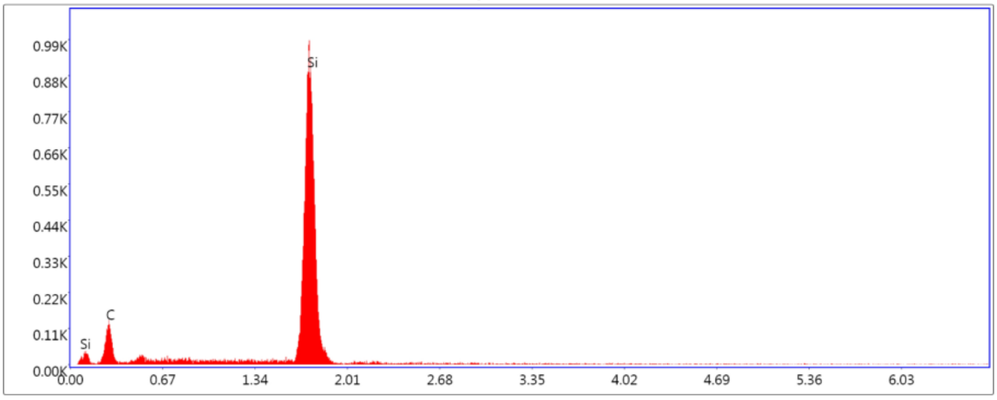

Lsec: 29.8 0 Cnts 5.390 keV Det: Octane Pro A

eZAF Smart Quant Results

Element	Weight %	Atomic %	Net Int.	Error %	Kratio	Z	A	F
C K	31.73	52.08	42.10	14.23	0.0617	1.1201	0.1735	1.0000
SiK	68.27	47.92	528.91	3.05	0.6403	0.9412	0.9959	1.0003

EDS Spot 6

kV: 10	Mag: 80	Takeoff: 40.3	Live Time(s): 29.9	Amp Time(µs): 1.92	Resolution:(eV) 127.4

EDS Spot 6 - Det 1

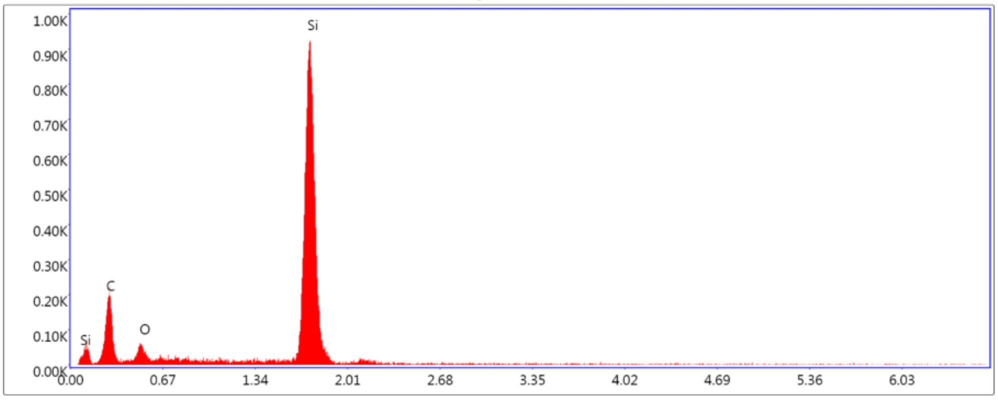

Lsec: 29.9   3 Cnts   5.390 keV   Det: Octane Pro A

eZAF Smart Quant Results

Element	Weight %	Atomic %	Net Int.	Error %	Kratio	Z	A	F
C K	40.21	60.01	75.82	12.48	0.0922	1.0989	0.2087	1.0000
O K	3.82	4.27	20.09	16.45	0.0156	1.0389	0.3924	1.0000
SiK	55.97	35.72	509.36	3.10	0.5121	0.9223	0.9914	1.0004

EDS Spot 7

kV: 10	Mag: 80	Takeoff: 40.3	Live Time(s): 29.9	Amp Time(µs): 1.92	Resolution:(eV) 127.4

EDS Spot 7 - Det 1

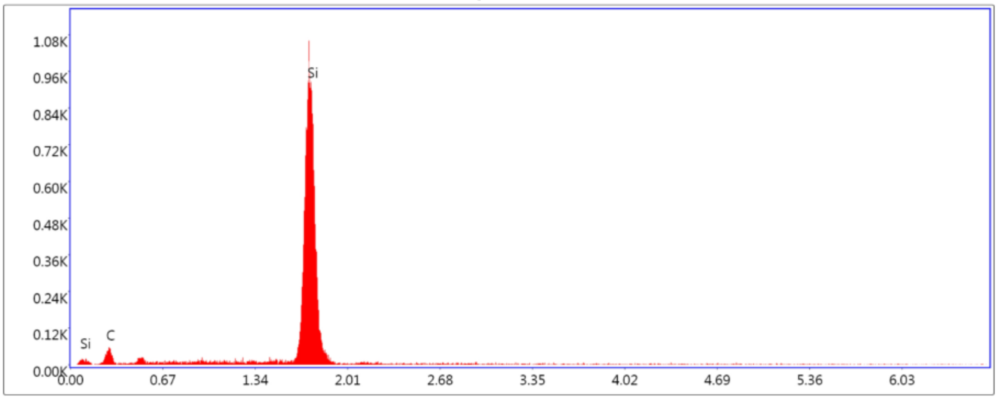

Lsec: 29.9   1 Cnts   5.390 keV   Det: Octane Pro A

eZAF Smart Quant Results

Element	Weight %	Atomic %	Net Int.	Error %	Kratio	Z	A	F
C K	16.48	31.57	15.18	18.65	0.0270	1.1511	0.1421	1.0000
SiK	83.52	68.43	550.24	2.99	0.8075	0.9687	0.9979	1.0001

EDS Spot 9

kV: 10	Mag: 80	Takeoff: 40.3	Live Time(s): 29.9	Amp Time(µs): 1.92	Resolution:(eV) 127.4

EDS Spot 9 - Det 1

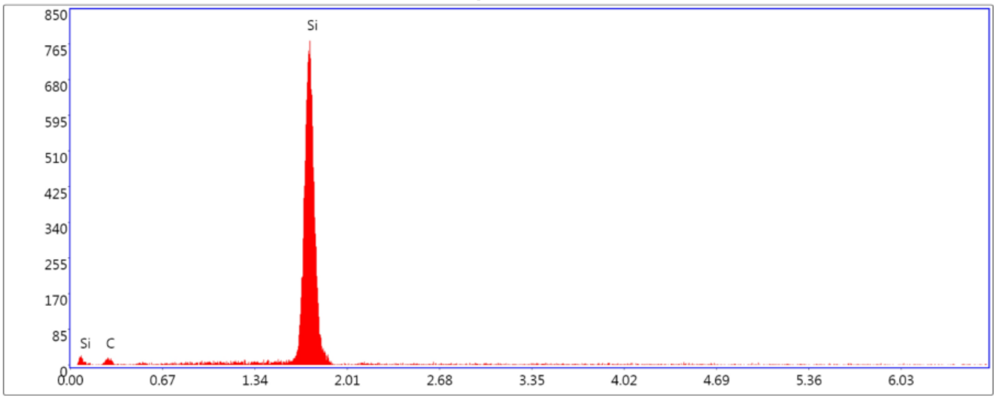

Lsec: 29.9 0 Cnts 5.390 keV Det: Octane Pro A

eZAF Smart Quant Results

Element	Weight %	Atomic %	Net Int.	Error %	Kratio	Z	A	F
C K	7.04	15.05	4.05	35.00	0.0105	1.1712	0.1276	1.0000
SiK	92.96	84.95	426.59	3.07	0.9161	0.9864	0.9991	1.0000

EDS Spot 11

kV: 10	Mag: 80	Takeoff: 40.3	Live Time(s): 29.8	Amp Time(µs): 1.92	Resolution:(eV) 127.4

EDS Spot 11 - Det 1

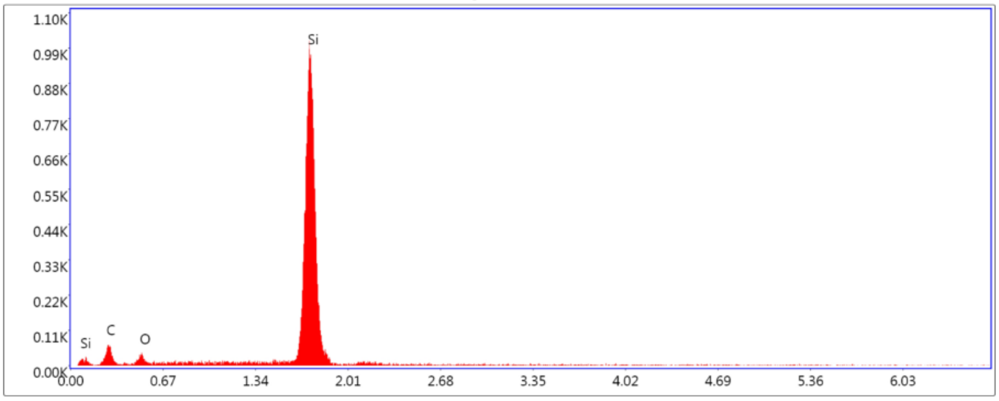

Lsec: 29.8 0 Cnts 5.390 keV Det: Octane Pro A

eZAF Smart Quant Results

Element	Weight %	Atomic %	Net Int.	Error %	Kratio	Z	A	F
C K	19.05	35.15	19.01	17.93	0.0325	1.1436	0.1493	1.0000
O K	1.62	2.25	6.91	30.70	0.0075	1.0822	0.4285	1.0000
SiK	79.33	62.60	537.63	3.01	0.7602	0.9620	0.9960	1.0002

EDS Spot 13

kV: 10	Mag: 80	Takeoff: 40.3	Live Time(s): 29.9	Amp Time(µs): 1.92	Resolution:(eV) 127.4

EDS Spot 13 - Det 1

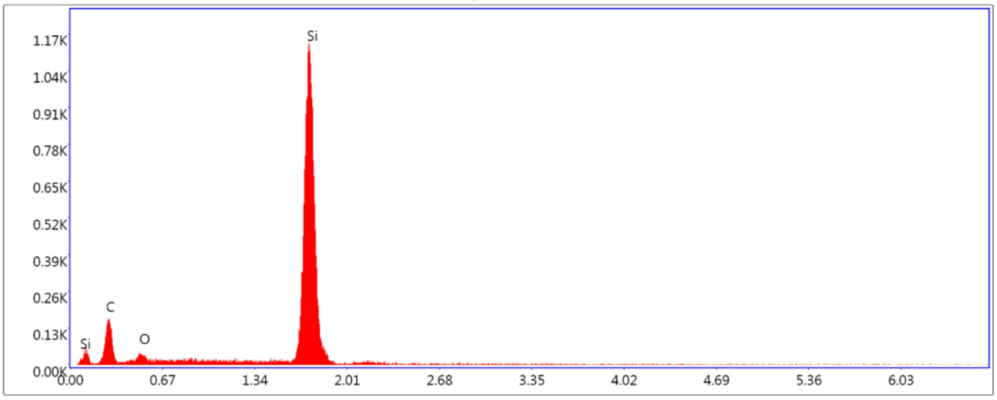

Lsec: 29.9 2 Cnts 5.390 keV Det: Octane Pro A

eZAF Smart Quant Results

Element	Weight %	Atomic %	Net Int.	Error %	Kratio	Z	A	F
C K	33.14	53.11	58.57	13.27	0.0672	1.1147	0.1820	1.0000
O K	2.07	2.49	11.98	20.28	0.0088	1.0542	0.4016	1.0000
SiK	64.79	44.40	635.63	2.96	0.6031	0.9363	0.9939	1.0003

EDS Spot 14

kV: 10	Mag: 80	Takeoff: 40.3	Live Time(s): 29.8	Amp Time(µs): 1.92	Resolution:(eV) 127.4

EDS Spot 14 - Det 1

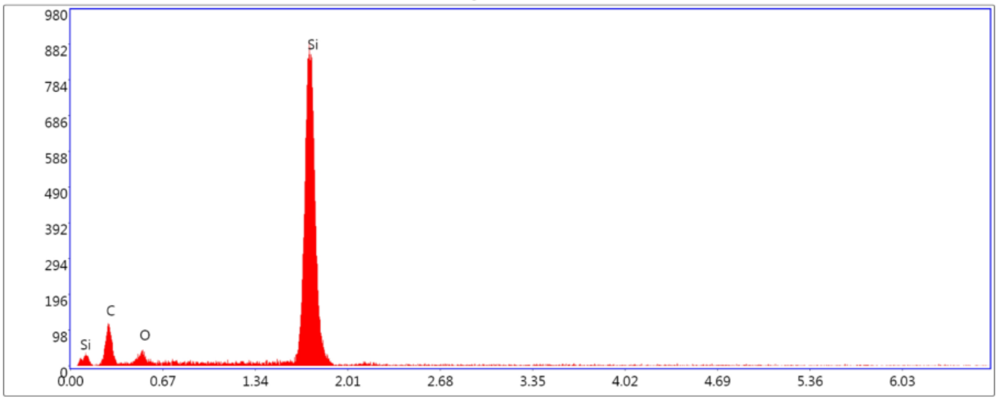

Lsec: 29.8 0 Cnts 5.390 keV Det: Octane Pro A

eZAF Smart Quant Results

Element	Weight %	Atomic %	Net Int.	Error %	Kratio	Z	A	F
C K	30.37	49.70	40.85	13.80	0.0601	1.1191	0.1767	1.0000
O K	2.95	3.62	13.65	17.13	0.0128	1.0584	0.4091	1.0000
SiK	66.69	46.68	512.87	3.02	0.6232	0.9402	0.9934	1.0003

EDS Spot 15

kV: 10 Mag: 80 Takeoff: 40.3 Live Time(s): 29.9 Amp Time(μs): 1.92 Resolution:(eV) 127.4

EDS Spot 15 - Det 1

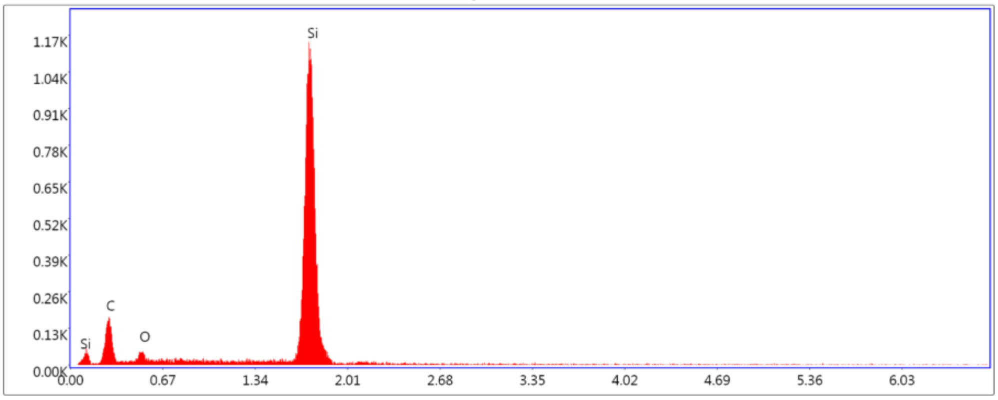

Lsec: 29.9 0 Cnts 5.390 keV Det: Octane Pro A

eZAF Smart Quant Results

Element	Weight %	Atomic %	Net Int.	Error %	Kratio	Z	A	F
C K	33.37	53.40	59.11	13.26	0.0678	1.1144	0.1823	1.0000
O K	1.93	2.31	11.15	24.11	0.0081	1.0539	0.4009	1.0000
SiK	64.70	44.28	635.20	2.98	0.6022	0.9361	0.9940	1.0003

EDS Spot 16

kV: 10 Mag: 80 Takeoff: 40.3 Live Time(s): 29.9 Amp Time(μs): 1.92 Resolution:(eV) 127.4

EDS Spot 16 - Det 1

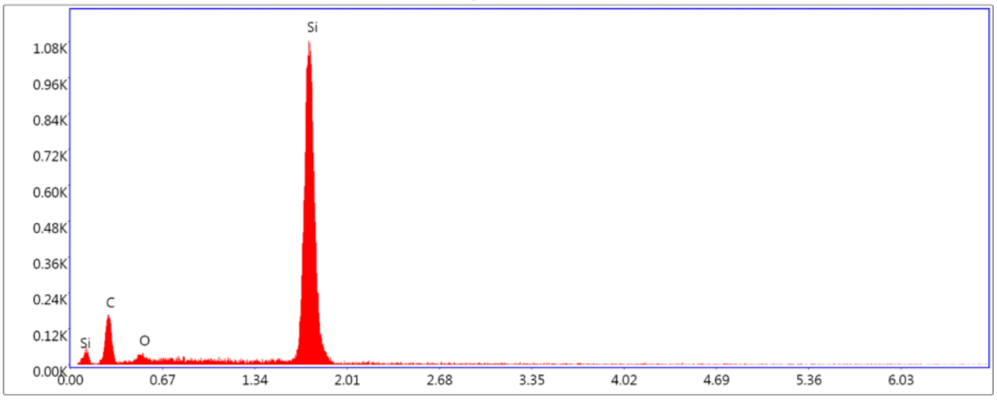

Lsec: 29.9 4 Cnts 5.390 keV Det: Octane Pro A

eZAF Smart Quant Results

Element	Weight %	Atomic %	Net Int.	Error %	Kratio	Z	A	F
C K	33.42	53.65	56.65	13.33	0.0673	1.1152	0.1807	1.0000
O K	1.21	1.46	6.75	36.85	0.0051	1.0547	0.3992	1.0000
SiK	65.37	44.89	620.07	2.98	0.6092	0.9368	0.9946	1.0003

Area 4

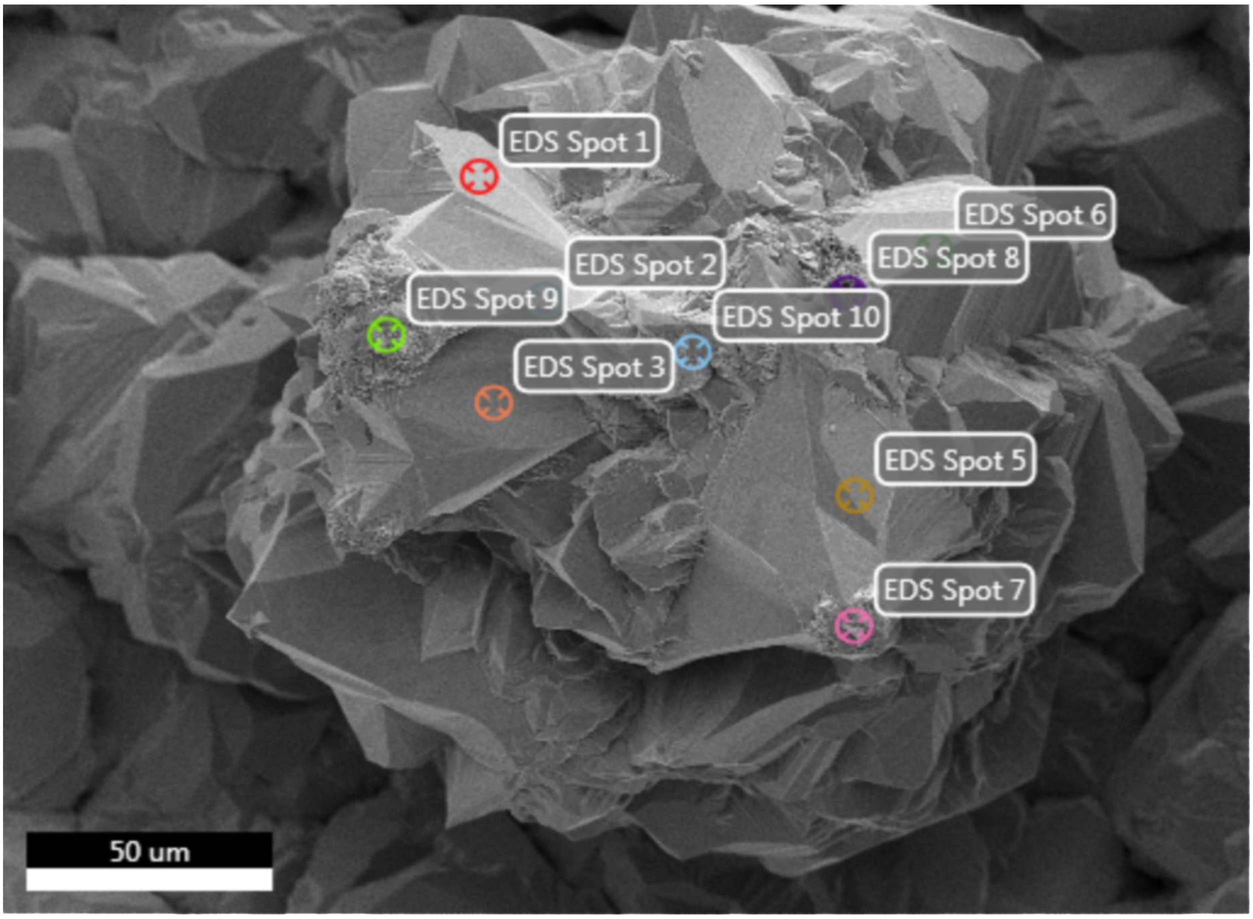
EDS Spot 1
EDS Spot 6
EDS Spot 8
EDS Spot 2
EDS Spot 9
EDS Spot 10
EDS Spot 3
EDS Spot 5
EDS Spot 7
50 um

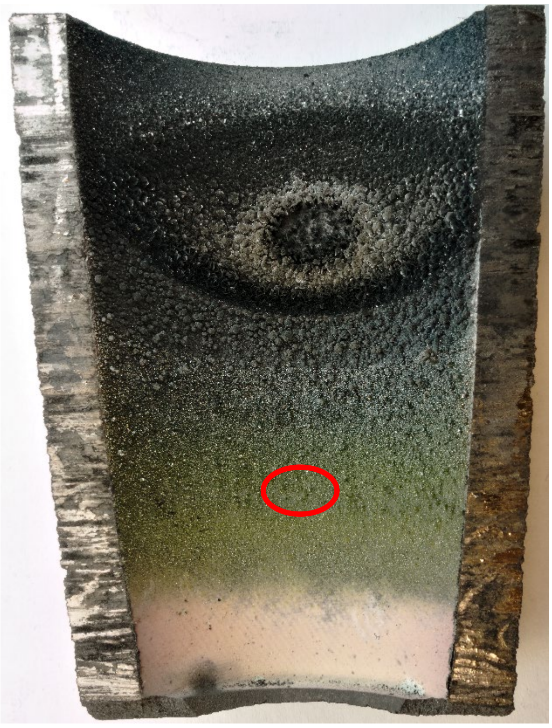

EDS Spot 1

kV: 10	Mag: 434	Takeoff: 40.2	Live Time(s): 29.9	Amp Time(μs): 1.92	Resolution:(eV) 127.4

EDS Spot 1 - Det 1

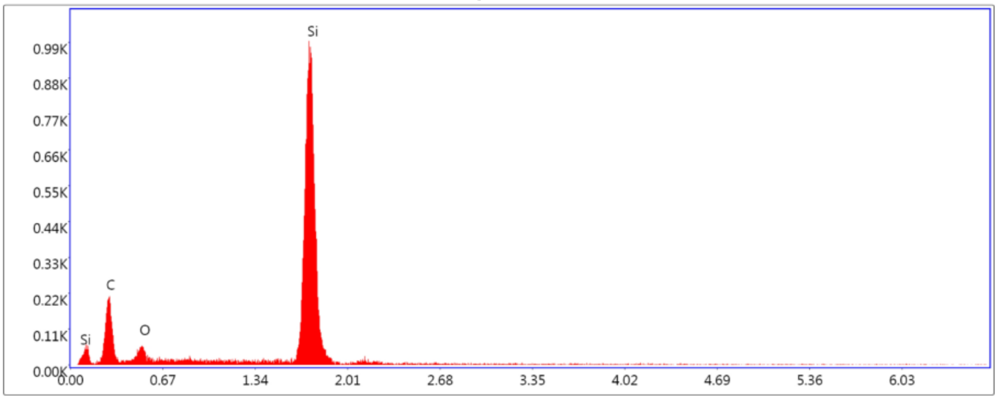

Lsec: 29.9   0 Cnts   5.390 keV   Det: Octane Pro A

eZAF Smart Quant Results

Element	Weight %	Atomic %	Net Int.	Error %	Kratio	Z	A	F
C K	39.85	59.67	79.15	12.23	0.0906	1.0996	0.2067	1.0000
O K	3.75	4.22	21.00	14.66	0.0153	1.0396	0.3921	1.0000
SiK	56.40	36.12	546.27	3.07	0.5164	0.9229	0.9915	1.0004

EDS Spot 2

kV: 10	Mag: 434	Takeoff: 40.2	Live Time(s): 29.8	Amp Time(μs): 1.92	Resolution:(eV) 127.4

EDS Spot 2 - Det 1

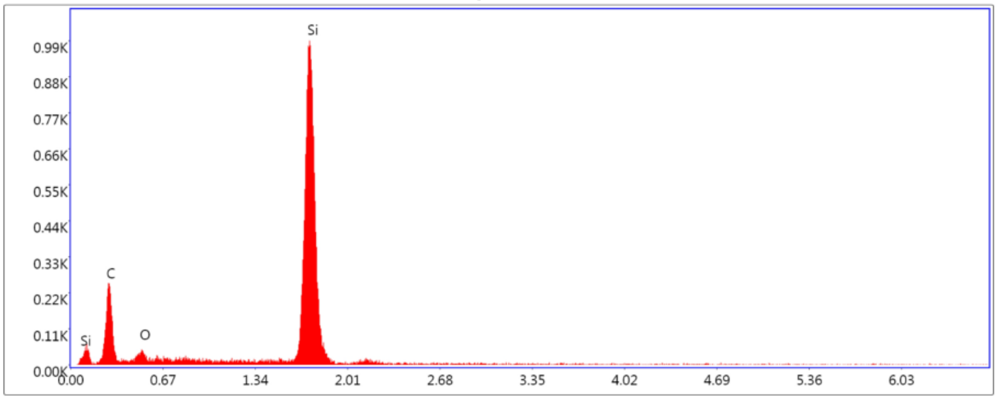

Lsec: 29.8   2 Cnts   5.390 keV   Det: Octane Pro A

eZAF Smart Quant Results

Element	Weight %	Atomic %	Net Int.	Error %	Kratio	Z	A	F
C K	42.20	62.32	88.51	12.06	0.0976	1.0967	0.2109	1.0000
O K	2.47	2.74	14.06	20.05	0.0099	1.0368	0.3851	1.0000
SiK	55.33	34.94	555.09	3.05	0.5057	0.9204	0.9924	1.0005

EDS Spot 3

kV: 10	Mag: 434	Takeoff: 40.2	Live Time(s): 29.9	Amp Time(μs): 1.92	Resolution:(eV) 127.4

EDS Spot 3 - Det 1

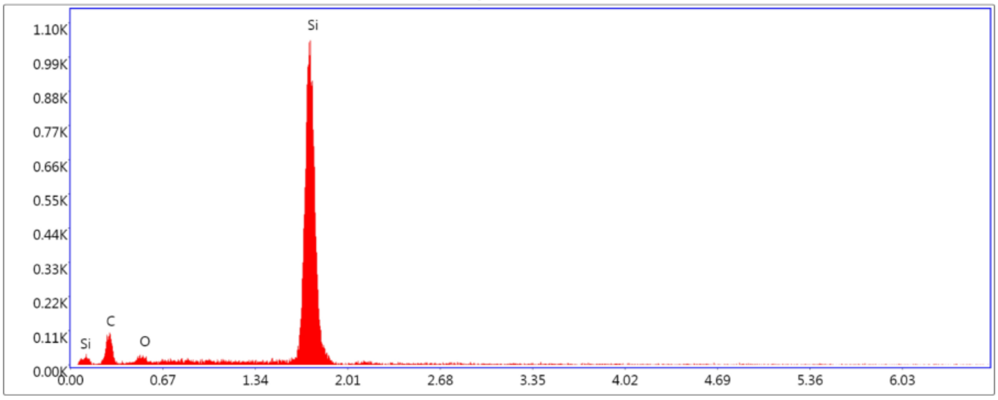

Lsec: 29.9 3 Cnts 5.390 keV Det: Octane Pro A

eZAF Smart Quant Results

Element	Weight %	Atomic %	Net Int.	Error %	Kratio	Z	A	F
C K	25.88	44.60	32.81	15.20	0.0475	1.1300	0.1623	1.0000
O K	1.38	1.79	6.64	30.74	0.0061	1.0690	0.4134	1.0000
SiK	72.74	53.61	575.82	2.97	0.6880	0.9500	0.9954	1.0002

EDS Spot 5

kV: 10	Mag: 434	Takeoff: 40.2	Live Time(s): 29.9	Amp Time(μs): 1.92	Resolution:(eV) 127.4

EDS Spot 5 - Det 1

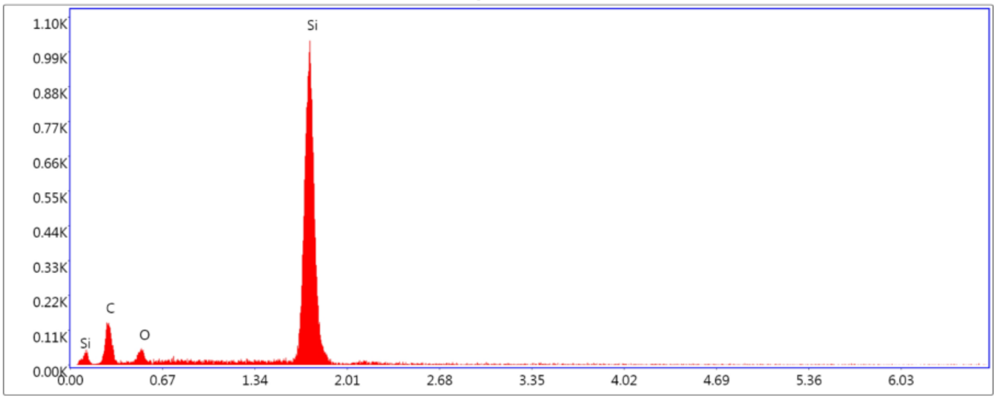

Lsec: 29.9 1 Cnts 5.390 keV Det: Octane Pro A

eZAF Smart Quant Results

Element	Weight %	Atomic %	Net Int.	Error %	Kratio	Z	A	F
C K	31.67	51.34	46.34	13.62	0.0632	1.1171	0.1786	1.0000
O K	2.46	3.00	12.14	20.37	0.0105	1.0565	0.4047	1.0000
SiK	65.86	45.66	545.72	3.03	0.6146	0.9385	0.9937	1.0003

EDS Spot 6

kV: 10	Mag: 434	Takeoff: 40.2	Live Time(s): 29.9	Amp Time(μs): 1.92	Resolution:(eV) 127.4

EDS Spot 6 - Det 1

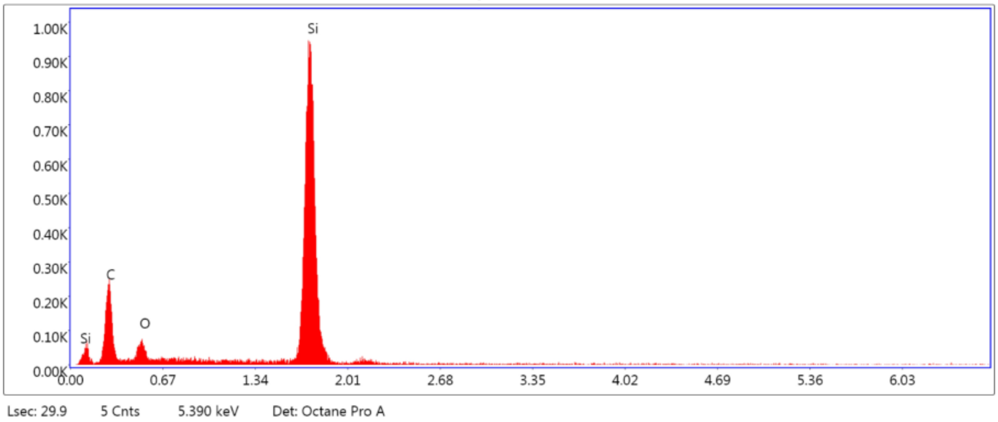

eZAF Smart Quant Results

Element	Weight %	Atomic %	Net Int.	Error %	Kratio	Z	A	F
C K	42.30	61.75	91.30	11.96	0.1012	1.0938	0.2188	1.0000
O K	4.73	5.18	27.02	14.15	0.0191	1.0340	0.3898	1.0000
SiK	52.98	33.07	526.13	3.12	0.4818	0.9177	0.9903	1.0005

EDS Spot 7

kV: 10	Mag: 434	Takeoff: 40.2	Live Time(s): 29.8	Amp Time(μs): 1.92	Resolution:(eV) 127.4

EDS Spot 7 - Det 1

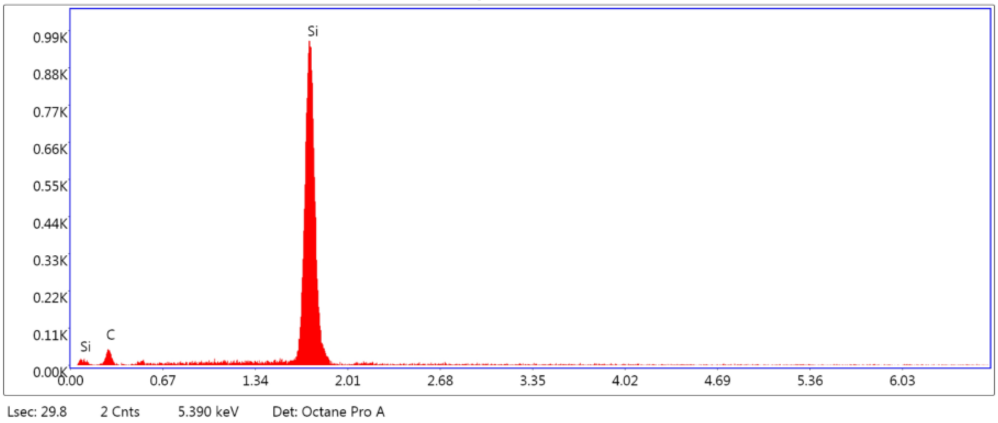

eZAF Smart Quant Results

Element	Weight %	Atomic %	Net Int.	Error %	Kratio	Z	A	F
C K	14.06	27.66	12.13	21.74	0.0224	1.1562	0.1378	1.0000
SiK	85.94	72.34	548.04	2.98	0.8350	0.9732	0.9982	1.0001

EDS Spot 8

kV: 10	Mag: 434	Takeoff: 40.2	Live Time(s): 30	Amp Time(μs): 1.92	Resolution:(eV) 127.4

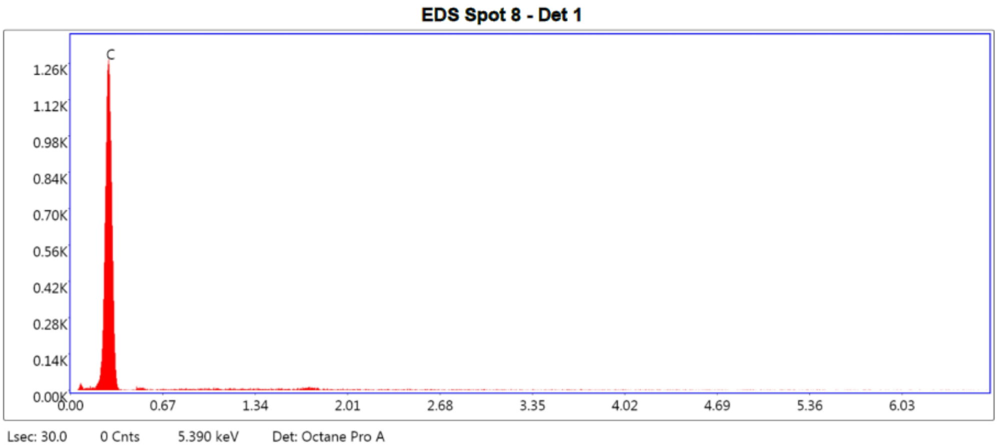

eZAF Smart Quant Results

Element	Weight %	Atomic %	Net Int.	Error %	Kratio	Z	A	F
C K	100.00	100.00	498.00	2.64	1.0000	1.0000	1.0000	1.0000

EDS Spot 9

kV: 10	Mag: 434	Takeoff: 40.2	Live Time(s): 29.9	Amp Time(μs): 1.92	Resolution:(eV) 127.4

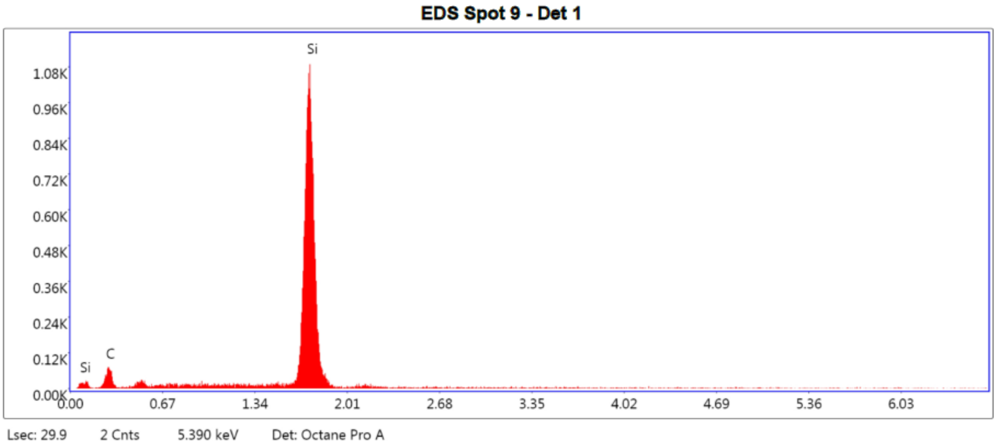

eZAF Smart Quant Results

Element	Weight %	Atomic %	Net Int.	Error %	Kratio	Z	A	F
C K	19.18	35.69	19.50	17.95	0.0322	1.1455	0.1465	1.0000
SiK	80.82	64.31	570.12	2.97	0.7770	0.9637	0.9975	1.0001

EDS Spot 10

kV: 10	Mag: 434	Takeoff: 40.2	Live Time(s): 29.8	Amp Time(µs): 1.92	Resolution:(eV) 127.4

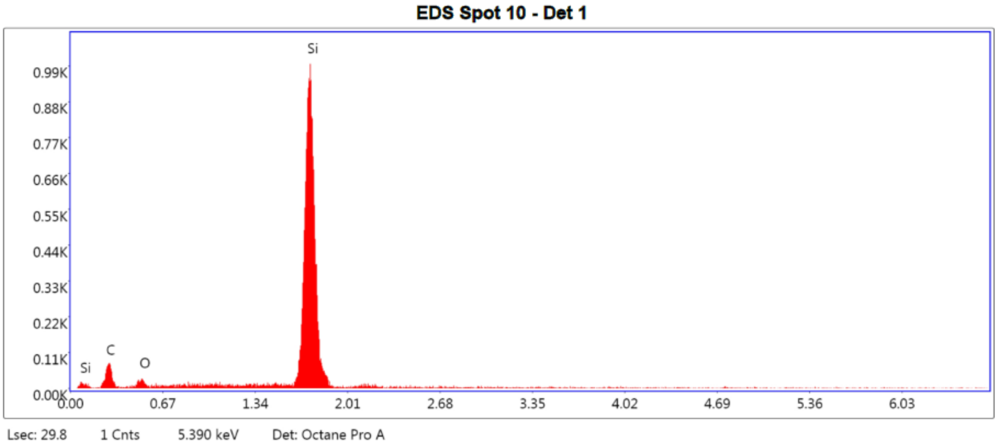

eZAF Smart Quant Results

Element	Weight %	Atomic %	Net Int.	Error %	Kratio	Z	A	F
C K	22.43	40.05	24.46	15.88	0.0395	1.1372	0.1548	1.0000
O K	1.22	1.63	5.35	37.91	0.0055	1.0760	0.4198	1.0000
SiK	76.36	58.32	545.77	3.00	0.7273	0.9563	0.9960	1.0002

5% CH_4 in H_2 at 1750°C, SiC from the alumina gas lance

Area 1

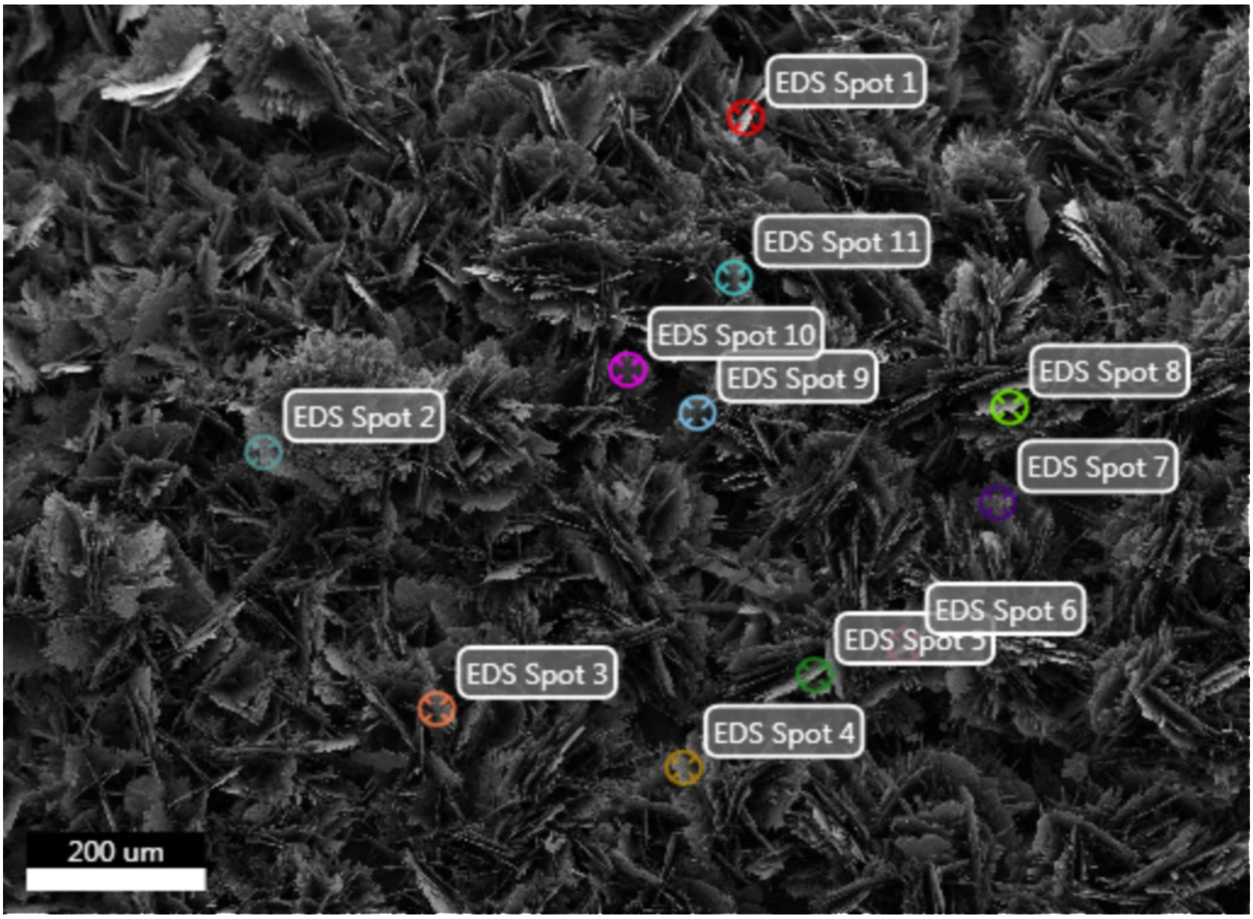

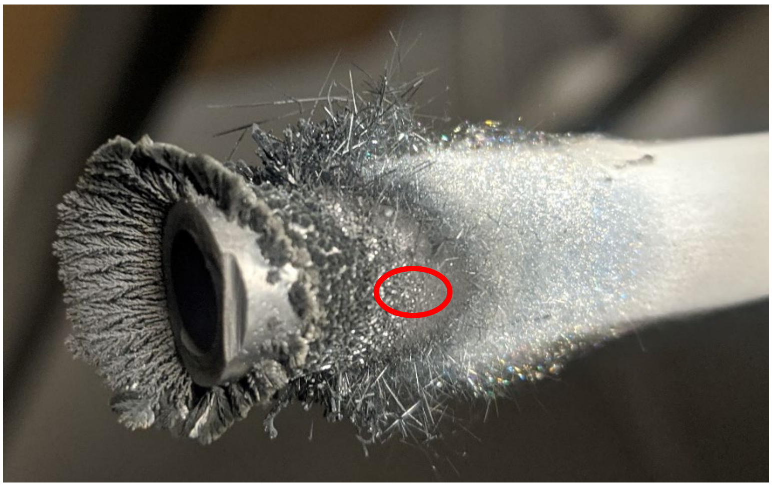

EDS Spot 1

kV: 10	Mag: 79	Takeoff: 30.8	Live Time(s): 29.8	Amp Time(μs): 1.92	Resolution:(eV) 127.4

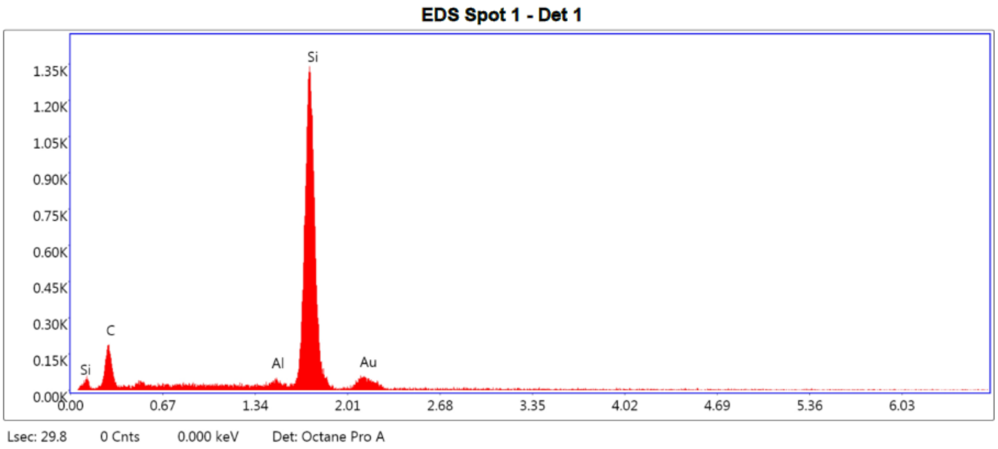

eZAF Smart Quant Results

Element	Weight %	Atomic %	Net Int.	Error %	Kratio	Z	A	F
C K	33.00	56.36	65.60	13.32	0.0637	1.1535	0.1673	1.0000
AlK	1.20	0.91	16.11	17.85	0.0109	0.9550	0.9464	1.0030
SiK	57.29	41.84	736.08	3.15	0.5418	0.9733	0.9715	1.0004
AuM	8.51	0.89	26.44	16.44	0.0511	0.5747	1.0489	0.9962

EDS Spot 2

kV: 10	Mag: 79	Takeoff: 30.8	Live Time(s): 29.8	Amp Time(μs): 1.92	Resolution:(eV) 127.4

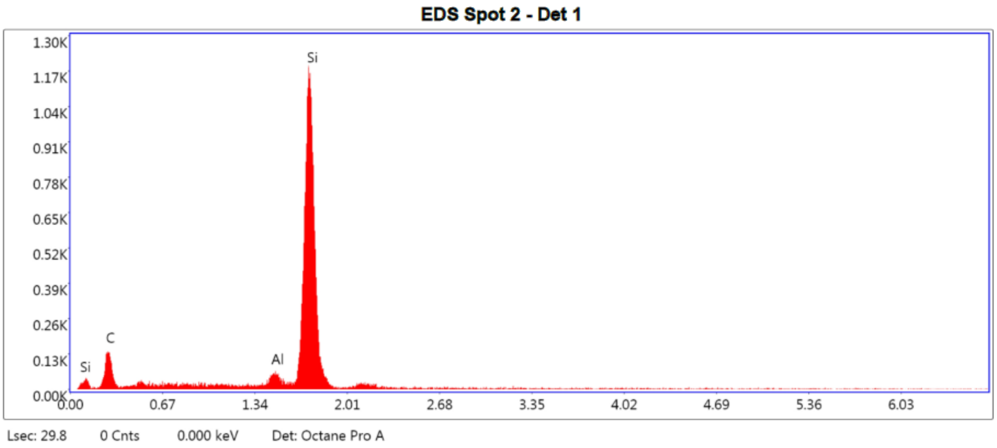

eZAF Smart Quant Results

Element	Weight %	Atomic %	Net Int.	Error %	Kratio	Z	A	F
C K	34.54	55.19	50.39	13.80	0.0587	1.1151	0.1525	1.0000
AlK	2.71	1.93	30.23	10.20	0.0245	0.9195	0.9693	1.0145
SiK	62.75	42.88	652.99	3.09	0.5770	0.9366	0.9817	1.0003

EDS Spot 3

kV: 10	Mag: 79	Takeoff: 30.8	Live Time(s): 30	Amp Time(µs): 1.92	Resolution:(eV) 127.4

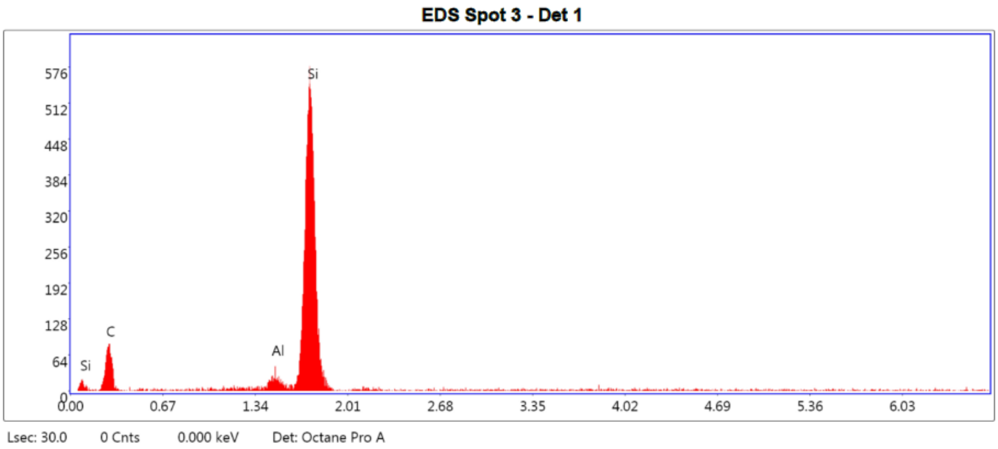

eZAF Smart Quant Results

Element	Weight %	Atomic %	Net Int.	Error %	Kratio	Z	A	F
C K	39.00	59.87	31.58	14.33	0.0707	1.1065	0.1637	1.0000
AlK	3.20	2.19	18.47	9.70	0.0287	0.9120	0.9682	1.0140
SiK	57.80	37.95	310.00	3.44	0.5258	0.9291	0.9787	1.0004

EDS Spot 4

kV: 10	Mag: 79	Takeoff: 30.8	Live Time(s): 29.8	Amp Time(µs): 1.92	Resolution:(eV) 127.4

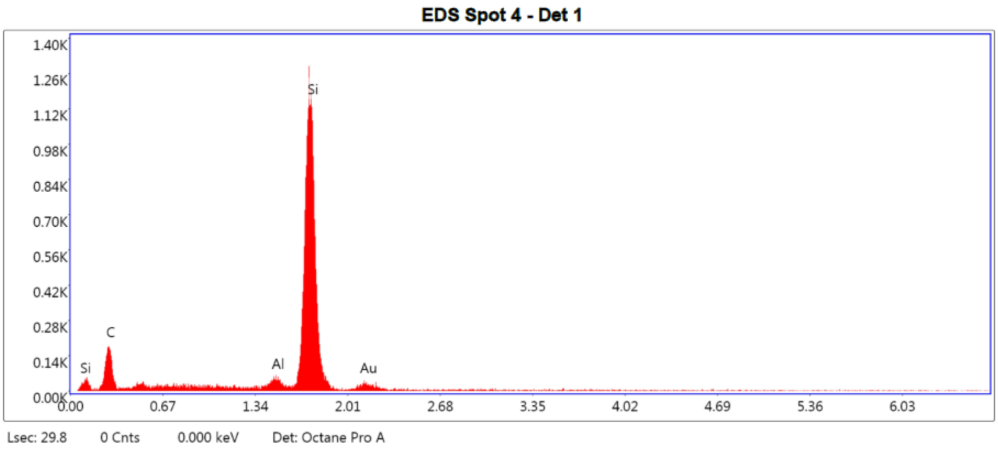

eZAF Smart Quant Results

Element	Weight %	Atomic %	Net Int.	Error %	Kratio	Z	A	F
C K	36.85	58.85	66.45	13.29	0.0687	1.1244	0.1660	1.0000
AlK	1.86	1.32	23.16	10.92	0.0166	0.9285	0.9590	1.0056
SiK	57.82	39.49	682.35	3.07	0.5351	0.9460	0.9781	1.0004
AuM	3.47	0.34	9.91	22.67	0.0204	0.5583	1.0573	0.9959

EDS Spot 5

kV: 10	Mag: 79	Takeoff: 30.8	Live Time(s): 29.9	Amp Time(μs): 1.92	Resolution:(eV) 127.4

EDS Spot 5 - Det 1

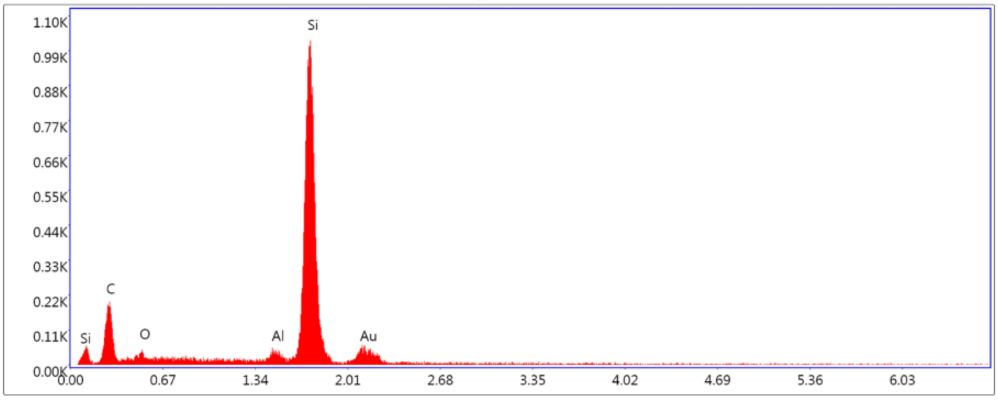

Lsec: 29.9 0 Cnts 0.000 keV Det: Octane Pro A

eZAF Smart Quant Results

Element	Weight %	Atomic %	Net Int.	Error %	Kratio	Z	A	F
C K	37.22	60.38	76.18	12.69	0.0808	1.1437	0.1898	1.0000
O K	2.76	3.37	15.55	17.89	0.0100	1.0831	0.3339	1.0000
AlK	1.71	1.23	20.67	12.31	0.0152	0.9467	0.9389	1.0025
SiK	49.18	34.12	569.44	3.33	0.4576	0.9648	0.9642	1.0005
AuM	9.13	0.90	26.27	15.72	0.0554	0.5696	1.0702	0.9964

EDS Spot 6

kV: 10	Mag: 79	Takeoff: 30.8	Live Time(s): 29.8	Amp Time(μs): 1.92	Resolution:(eV) 127.4

EDS Spot 6 - Det 1

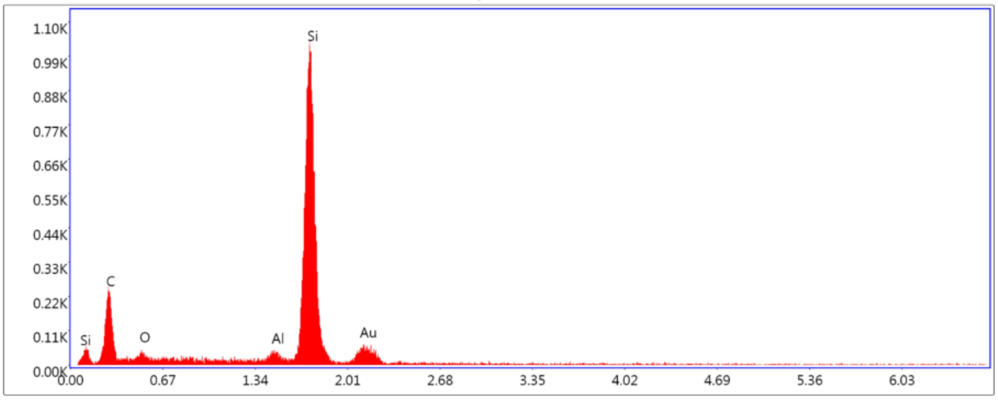

Lsec: 29.8 0 Cnts 0.000 keV Det: Octane Pro A

eZAF Smart Quant Results

Element	Weight %	Atomic %	Net Int.	Error %	Kratio	Z	A	F
C K	40.33	63.97	91.72	12.37	0.0925	1.1405	0.2011	1.0000
O K	2.28	2.72	13.20	21.04	0.0081	1.0801	0.3275	1.0000
AlK	1.54	1.09	19.53	13.06	0.0137	0.9441	0.9373	1.0023
SiK	46.11	31.28	559.50	3.33	0.4279	0.9621	0.9638	1.0005
AuM	9.73	0.94	29.61	16.74	0.0595	0.5681	1.0792	0.9965

EDS Spot 7

kV: 10	Mag: 79	Takeoff: 30.8	Live Time(s): 29.8	Amp Time(μs): 1.92	Resolution:(eV) 127.4

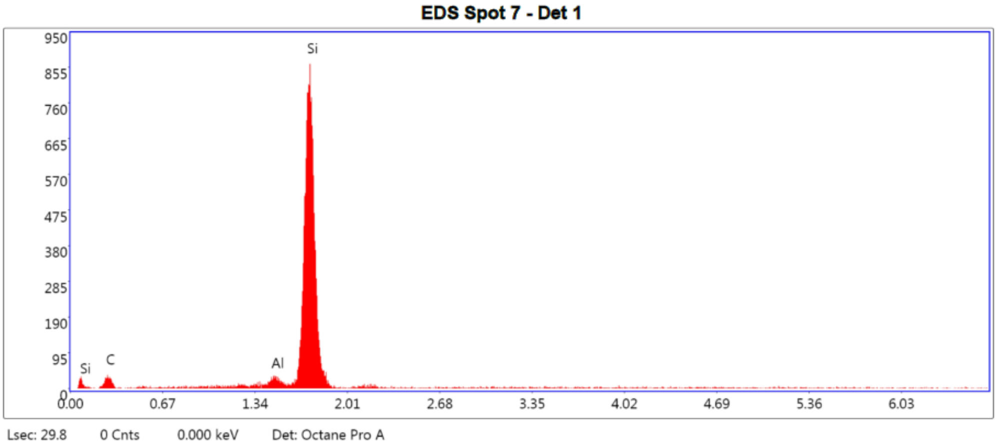

eZAF Smart Quant Results

Element	Weight %	Atomic %	Net Int.	Error %	Kratio	Z	A	F
C K	16.14	31.01	10.38	22.25	0.0222	1.1526	0.1191	1.0000
AlK	3.20	2.74	20.32	11.18	0.0301	0.9520	0.9748	1.0150
SiK	80.66	66.25	474.61	3.20	0.7685	0.9699	0.9822	1.0001

EDS Spot 8

kV: 10	Mag: 79	Takeoff: 30.8	Live Time(s): 29.8	Amp Time(μs): 1.92	Resolution:(eV) 127.4

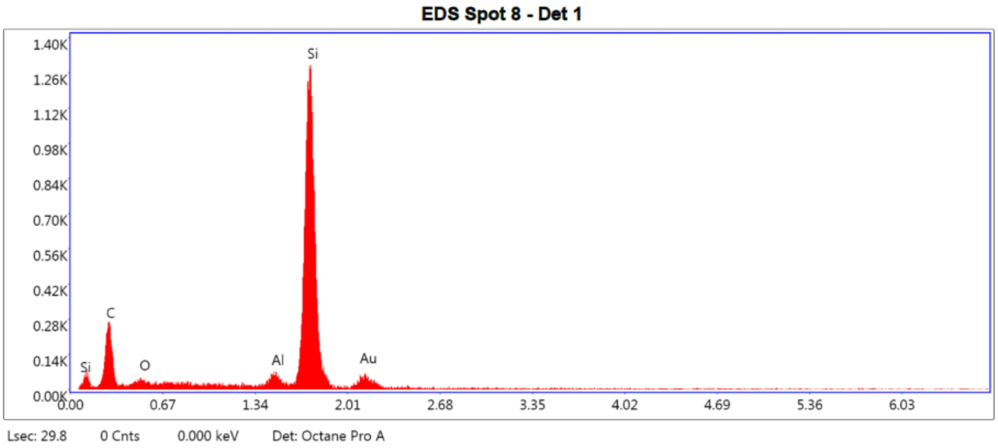

eZAF Smart Quant Results

Element	Weight %	Atomic %	Net Int.	Error %	Kratio	Z	A	F
C K	40.04	62.73	102.26	12.37	0.0855	1.1291	0.1892	1.0000
O K	1.76	2.07	12.17	24.75	0.0062	1.0688	0.3279	1.0000
AlK	1.96	1.37	29.86	10.60	0.0173	0.9334	0.9464	1.0032
SiK	49.54	33.19	720.17	3.19	0.4568	0.9511	0.9686	1.0005
AuM	6.70	0.64	24.16	19.55	0.0402	0.5614	1.0745	0.9962

EDS Spot 9

kV: 10	Mag: 79	Takeoff: 30.8	Live Time(s): 29.9	Amp Time(μs): 1.92	Resolution:(eV) 127.4

EDS Spot 9 - Det 1

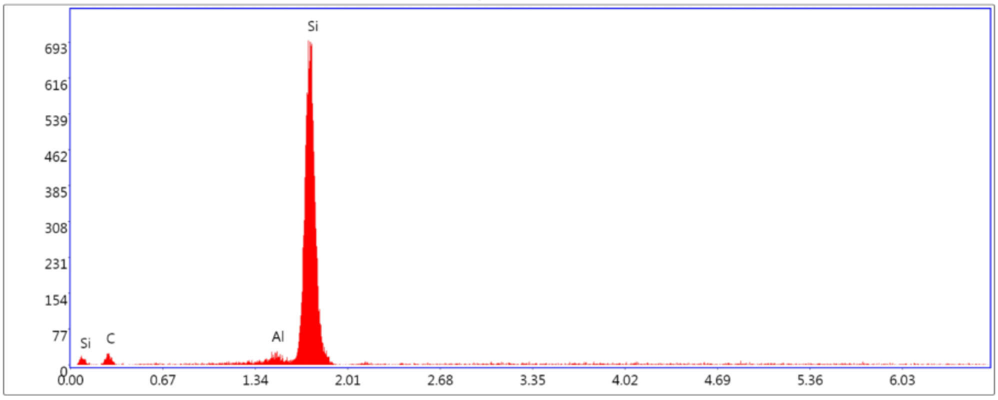

Lsec: 29.9 0 Cnts 0.000 keV Det: Octane Pro A

eZAF Smart Quant Results

Element	Weight %	Atomic %	Net Int.	Error %	Kratio	Z	A	F
C K	14.27	27.99	7.30	23.32	0.0192	1.1565	0.1165	1.0000
AlK	3.31	2.89	17.10	11.38	0.0313	0.9554	0.9753	1.0150
SiK	82.42	69.13	394.58	3.26	0.7879	0.9734	0.9820	1.0001

EDS Spot 10

kV: 10	Mag: 79	Takeoff: 30.8	Live Time(s): 29.9	Amp Time(μs): 1.92	Resolution:(eV) 127.4

EDS Spot 10 - Det 1

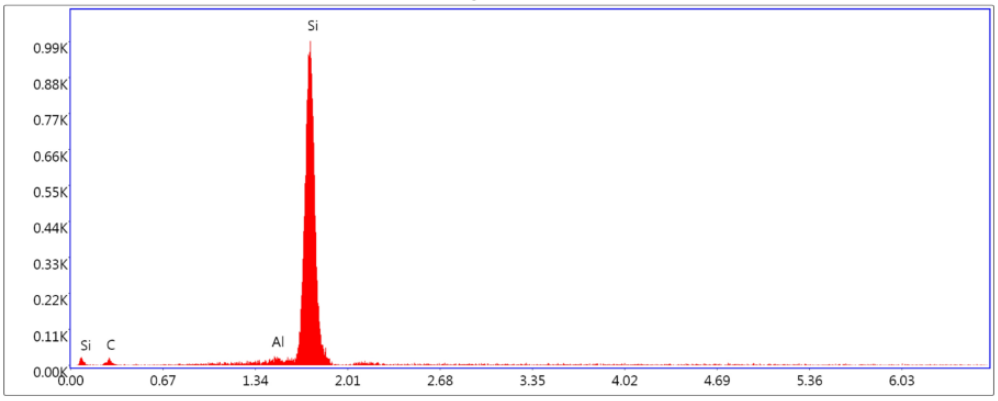

Lsec: 29.9 0 Cnts 0.000 keV Det: Octane Pro A

eZAF Smart Quant Results

Element	Weight %	Atomic %	Net Int.	Error %	Kratio	Z	A	F
C K	5.25	11.46	3.01	63.39	0.0065	1.1757	0.1052	1.0000
AlK	2.71	2.63	17.44	13.79	0.0261	0.9720	0.9779	1.0156
SiK	92.04	85.91	549.78	3.09	0.8990	0.9903	0.9862	1.0000

EDS Spot 11

kV: 10	Mag: 79	Takeoff: 30.8	Live Time(s): 29.9	Amp Time(μs): 1.92	Resolution:(eV) 127.4

EDS Spot 11 - Det 1

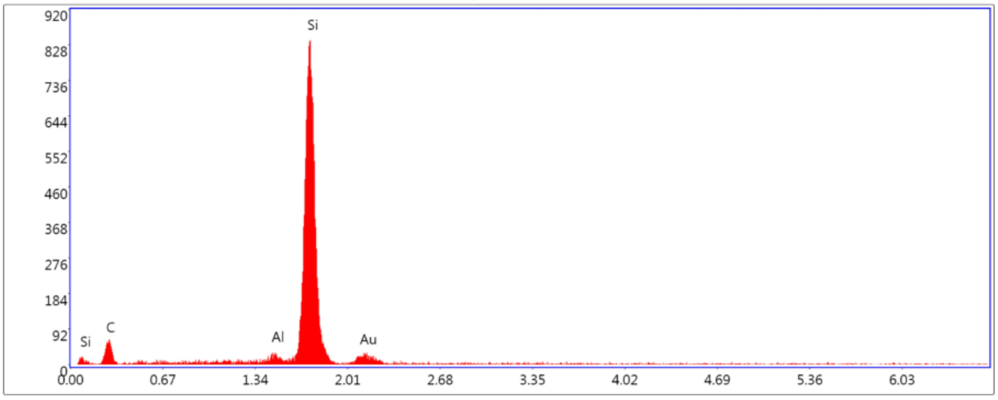

Lsec: 29.9 0 Cnts 0.000 keV Det: Octane Pro A

eZAF Smart Quant Results

Element	Weight %	Atomic %	Net Int.	Error %	Kratio	Z	A	F
C K	23.36	43.75	21.35	16.36	0.0390	1.1700	0.1429	1.0000
AlK	1.77	1.48	12.92	15.30	0.0164	0.9690	0.9518	1.0036
SiK	67.32	53.92	466.33	3.31	0.6465	0.9876	0.9723	1.0003
AuM	7.54	0.86	12.26	20.60	0.0446	0.5831	1.0195	0.9961
